# Nitrile Hydroboration by Cooperative Iron Catalysis: An Experimental and Computational Study

**DOI:** 10.1002/chem.202501782

**Published:** 2025-07-02

**Authors:** Laura A. Grose, Yi Zhang, Samuel Oultram, Ryan J. Schwamm, Sam. P. de Visser, Darren Willcox

**Affiliations:** ^1^ Department of Chemistry The University of Manchester Oxford Road Manchester M13 9PL UK; ^2^ Department of Chemical Engineering The University of Manchester Oxford Road Manchester M13 9PL UK; ^3^ Manchester Institute of Biotechnology The University of Manchester 131 Princess Street Manchester M1 7DN UK

**Keywords:** cooperative catalysis, DFT calculations, hydroboration, iron catalysis, kinetic measurements

## Abstract

Reductive amination is a challenging reaction in catalysis that often gives poor yield and selectivity. We present an iron‐catalyzed approach of synthesizing amines through reduction of nitriles through hydroboration with good yield under ambient conditions. Our detailed mechanistic study establishes the factors that influence the selectivity and turnover. The kinetics and mechanism of the iron‐catalyzed hydroboration of benzonitrile to bis(boryl)benzylamine have been investigated by initial rates, temperature dependence, kinetic isotope effects, and computational studies. In contrast to other iron‐catalyzed nitrile hydroboration, this study reveals that B─H bond activation is not rate‐determining. Moreover, the rate‐determining step was revealed to be C─H bond reductive elimination with an equilibrium isotope effect in operation. Through this combined approached, an Fe(0)/(II) catalytic manifold proceeding via metal‐ligand cooperativity has been determined.

## Graphical abstract

We describe the Fe‐catalyzed double hydroboration of nitriles using HBpin as the terminal reductant. Mechanistic studies, both experimental and computational, reveal a unique and unreported mechanistic pathway for iron‐catalyzed hydroboration where reductive elimination, rather than B─H bond activation is the rate‐ limiting step.

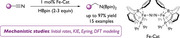

## 1 Introduction

Amines are one of the most important functional groups in organic chemistry and are ubiquitous in material science, dyes, agrochemicals, and pharmaceutical agents.^[^ ^1^, ^2^, ^3^ ^]^ To date, the most widespread synthetic approaches to generate amines are via *N*‐alkylation, reductive amination of a carbonyl compound as well as stoichiometric reduction amide, imines, and nitriles using metal hydride species.^[^ ^4^, ^5^, ^6^, ^7^, ^8^, ^9^, ^10^, ^11^, ^12^ ^]^ Although these approaches are routinely used, they do suffer from deleterious onward reactivity, functional group tolerance, and copious quantities of undesired byproducts.^[^ ^13^, ^14^ ^]^ Promising alternatives to these stoichiometric reductions include catalytic hydrogenation,^[^ ^15^ ^]^ hydrosilylation,^[^ ^16^ ^]^ and hydroboration^[^ ^17^ ^]^ of readily available nitriles.

Of these transformations, hydrogenation of nitriles to amines is the most atom‐economical approach, however precious metal catalysts and harsh reaction conditions are often required which can lead to poor selectivity.^[^ ^18^ ^]^ Conversely, there have been numerous well‐defined first‐row transition metal systems developed for catalytic nitrile hydrosilylation, whereas examples of double hydroboration using such systems remain scarce and typically require elevated temperatures.^[^ ^15^, ^16^, ^19^ ^]^ The catalytic double hydroboration of nitriles selectively furnishes the bis(boryl)amino moieties which due to labile B─N bonds have been readily converted to the corresponding primary amine via hydrolysis, to amides via coupling with carboxylic acids and to aldimines through reactions with aldehydes.^[^ ^20^, ^29^ ^]^

**Figure 1:**
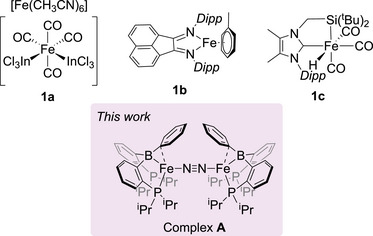
Selected iron complexes effective for hydroboration of nitriles and *N*‐heteroarenes and the precatalyst, complex A used for this work.

As one of the most abundant metals on earth, iron offers a level of sustainable and longstanding availability, accompanied by low toxicity that is unorthodox compared to other transition metals. The use of iron complexes in the hydroboration of unsaturated C─N bonds has been overshadowed by work with precious metals, however, in recent years a range of structurally diverse catalytically active iron complexes has emerged (Figure 1). The first example of iron‐catalyzed nitrile hydroboration was reported by Nakazawa using a cooperative iron‐indium heterobimetallic system, **1a**.^[^ ^21^ ^]^ Findlater and coworkers reported an iron complex bearing the redox active bis(imino)acenaphthene (BIAN) ligand for nitrile hydroboration without the use of an exogenous activator, **1b**.^[^ ^22^ ^]^ Recently, Hashimoto and coworkers reported the use of an iron silyl‐NHC complex **1c** with activation via UV irradiation (> 300 nm) to promote nitrile reduction with HBpin.^[^ ^23^ ^]^ Despite these examples, there are very few reports that provide mechanistic analysis of the reactions either experimentally or computationally.

We previously demonstrated that an iron(0) complex containing a diphosphinoborane ligand scaffold (complex **A**) was a suitable precatalyst for the hydroboration of terminal olefins.^[^ ^24^ ^]^ Given the paucity of reports utilizing complex **A** in catalysis, we wondered if the hydroboration chemistry using **A** could be extended to substrates other than olefins. Herein we disclose that **A** is a suitable precatalyst for nitrile hydroboration and provide a combined experimental and computational mechanistic insight into nitrile hydroboration.

## 2 Results and Discussion

### 2.1 Catalytic Reaction Scope

We initiated our research with the hydroboration of nitriles. Optimization of reaction parameters was conducted using benzonitrile as the model substrate. Several reaction variables were screened to optimize product yield, namely the catalyst loading, the temperature, and the solvent. Ultimately, employing benzene as the reaction solvent with 1 mol% catalyst loading at 50 °C afforded the cleanly converted amine product (**3d**, 95%) within 3 hours.

**Scheme 1:**
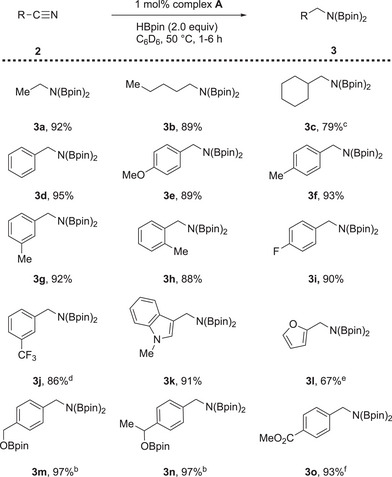
Substrate scope for the iron(0)‐ catalyzed hydroboration of aromatic and aliphatic nitriles.^[a]^ ^[a]^ Reactions were performed with substrate (0.205 mmol), HBpin (0.410 mmol, 2.0 equiv.), Complex **A** (1.0 mol%), in 0.6 mL of benzene‐[d]_6_ for 1–6 hours at 50 °C. ^[b]^ 3.0 equivalents of HBpin were used. ^[c]^ 5 hours. ^[d]^ 4 hours. ^[e]^ 6 hours. ^[f]^ 9 hours.

Encouraged by the successful use of **A** as a suitable precatalyst for benzonitrile reduction, we explored a range of nitrile‐containing substrates with electron‐donating and electron‐withdrawing groups, as well as those with heteroatoms and sterically hindered groups (Scheme 1). Simple aliphatic nitriles were reduced in high isolated yields within 3 hours (**3a**, **3b**), whereas the slightly more sterically hindered cyclohexanecarbonitrile required longer reaction times (5 hours) to give **3c** in 79% yield. Benzonitrile and derivatives containing electron‐donating and ‐withdrawing groups were reduced to the corresponding bis(boryl)benzylamine product in good to excellent yields within 3 hours, except for the *meta*‐trifluoromethylphenyl derivative (**3j**), which required a reaction time of 4 hours to achieve 86% yield. The isolated yields are comparable to those previously reported in other iron‐catalyzed systems.^[^ ^21^, ^22^, ^23^ ^]^

To demonstrate the power of this work, the hydroboration of nitriles bearing heterocyclic aromatic substrates was also explored. These substrates typically prove challenging or are unreported for iron‐catalyzed nitrile hydroboration.^[^ ^21^, ^22^, ^23^ ^]^ Indole **3k** was reduced readily in 3 hours giving good isolatable yields, that is, 91%; however, 2‐furonitrile took 6 hours to produce a moderate yield of 67% **3l**.

In line with our previous studies, the only halogen compound tolerated for this catalytic system was fluorine (**3i**).^[^ ^24^ ^]^ The use of other halogenated compounds resulted in single electron oxidation of the iron species by halide abstraction. With the double hydroboration established, we wanted to test the chemoselectivity of the hydroboration to ascertain if we could generate the *N*‐borylimine. Unfortunately, under the same conditions developed above, using 1 equivalent of HBpin, only the double hydroboration product was observed using ^1^H NMR spectroscopy with remaining benzonitrile the only other species present. When nitriles containing other reducible functional groups were explored with 2 equivalents of HBpin, for example *p*‐cyanobenzaldehyde, a mixture of the fully reduced borylated amino‐alcohol and the aldehyde reduction product was observed (see Supporting Information). However, the corresponding fully reduced borylated products (**3m** and **3n**) could be obtained when using 3 equivalents of HBpin in good yields. Interestingly, it was found that when the nitrile contained an ester substituent, only the nitrile was reduced to the corresponding diborylamine and the ester remained untouched (**3o**).

### 2.2 Stoichiometric Studies

To gain insight into these successful catalytic reactions, a series of stoichiometric reactions was conducted. Monitoring of NMR scale reactions performed using PhCN was particularly informative. We previously reported that **A** reacts with an excess of HBpin to generate an iron(II) dihydride species (**B**), along with B_2_pin_2_ and PhBpin within 6 hours at 50 °C.^[^ ^24^ ^]^ When **B** was reacted with an equimolar quantity of PhCN, no reaction was observed at either room temperature or 50 °C after 6 hours. These results suggest that, unlike some transition metal ‐catalyzed hydroboration reactions,^[^ ^25^ ^]^ autocatalytic hidden‐borane catalysis^[^ ^26^ ^]^ is not in operation during the course of the reaction as no borane/borohydride products were observed by ^11^B NMR spectroscopy.

**Figure 2:**
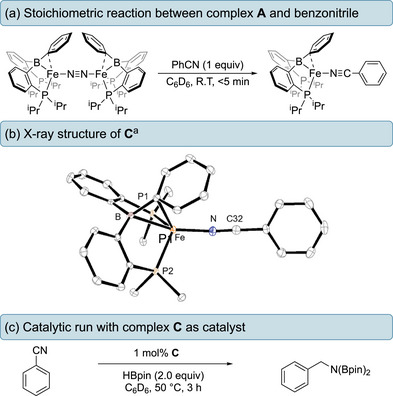
a) synthesis of complex C, b) Solid‐state structure of complex C, c) utilizing complex C as a catalyst in hydroboration. ^[a]^ Ellipsoids are drawn at the 50% probability level, methyl groups and hydrogen atoms on isopropyl groups have been omitted for clarity. Selected distances of **C** (Å): Fe─P1 2.3121(6), Fe─P2 2.2792(6), Fe─N1 1.9376(19), Fe─B1 2.318(2), N1─C32 1.154(3) C32─C33 1.440(3).

Treatment of complex **A** with an equimolar quantity of benzonitrile resulted in a rapid color change from dark red to black at ambient temperature coinciding with the formation of a new paramagnetic complex (**C**) as observed by ^1^H NMR spectroscopy. Complex **C** was isolated as a black solid and was characterized by X‐ray crystallographic analysis revealing complex **C** to exist as a mononuclear iron species [{(^iPr^DPB^Ph^)Fe(NCPh)}] in the solid state with a highly distorted trigonal bipyramidal geometry (*τ*_5_ = 0.67). From the solid‐state structure, it is apparent that the benzonitrile is coordinated to the iron in an end‐on manner through the nitrogen‐lone pair (Figure 2). Analysis of the crystal structure reveals an Fe─N bond length of 1.9376(19) Å, a C≡N bond length of 1.154(3) Å and a NC─C bond length of 1.440(3) Å. A significant deviation in the Fe─N─C32 bond angle (166.88(19)°) from linearity was also observed. In addition, an absorbance centered at 2149 cm^−1^ was observed in the IR spectrum (ATR‐FTIR), which is shifted to a lower stretching frequency by 79 cm^−1^ compared to free PhCN (2228 cm^−1^). These data suggest there is a degree of π‐back‐bonding from the electron rich metal center to the nitrile π*‐orbital. This is in accordance with the iron(0) complex, [(^iPr^PDI)Fe(NCPh)] reported by Chirik.^[^ ^27^ ^]^ A solution phase magnetic moment of 2.84 μ_B_ (Evan's method, C_6_D_6_, 25 °C, run 1 = 2.84 μ_B_; run 2 = 2.84 μ_B_) was obtained indicating complex **C** has an S = 1 ground state. When the solid‐state structures of **C** and **A** are compared, it is evident that the η3‐hapticity of the B─C_(ipso)_─C fragment is retained in complex **C**, however, an increase in the P1─Fe─P2 bond angle is observed coupled with slightly longer P─Fe bonds for complex **C**.

Treatment of **C** with an equimolar quantity of HBpin at ambient temperature, furnished a mixture of the bis(boryl)amine **3d**, unreacted complex **C,** and regenerated complex **A**. Increasing the quantity of HBpin to two equivalents generates **3d** and complex **A** exclusively, as observed by ^1^H NMR spectroscopy. Finally, Isolated complex **C** was directly applied as a catalyst in place of complex **A** under identical conditions to that of the standard reaction conditions for the hydroboration of benzonitrile (**2d**). To our delight, the yield of **3d** was comparable in both systems. This result strongly suggests that complex **C** is an on‐cycle species in the catalytic nitrile hydroboration.

### 2.3 Kinetic Studies

**Figure 3:**
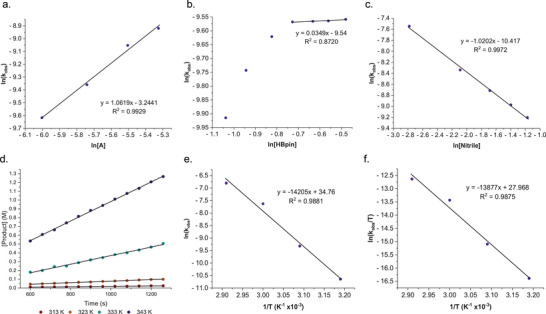
a) van ’t Hoff plot for the reaction rate law order in [complex A]; b) van ’t Hoff plot for the reaction rate law order in [HBpin]; c) van ’t Hoff plot for the reaction rate law order in [nitrile]; d) Initial rates of hydroboration at different temperatures. e) Eyring plot f) Arrhenius plot.

To further ascertain mechanistic insights into the double nitrile hydroboration, a kinetic investigation was undertaken. Benzonitrile was selected as the representative nitrile substrate as the product (**3d**) has clearly observable product resonances in the ^1^H NMR spectra. Initial reactions were monitored by ^1^H NMR spectroscopy following the growth of the hydroboration product, **3d** at 50 °C using the standard reaction conditions of 1 mol% Complex **A** and a 2:1 ratio of benzonitrile (0.33 M) to HBpin (0.66 M). (Figure 3). The reaction conformed to first order kinetic behavior, with a first order dependence on [**A**] being deduced through the variation of the catalyst loading whilst keeping the concentrations of PhCN and HBpin constant (Figure 3a). This first order dependency is suggestive of a rapid conversion of dimeric **A** into a monomeric species, presumably complex **C**, as observed in the stoichiometric reactions. Variation of the [benzonitrile] revealed an inverse‐first order dependency on the nitrile indicating inhibition is occurring (Figure 3b). To further probe this inhibition effects, **A** was subjected to 2 equivalents of benzonitrile, and the mixture was monitored by ^1^H NMR spectroscopy. From the ^1^H NMR spectrum, complex **C** can be clearly identified by comparing with the authentic sample, however, the formation of a new paramagnetic species is also observed. Unfortunately, attempts to isolate this new species proved unsuccessful. Finally, the order in [HBpin] was determined by variation of the [HBpin] under standard reaction conditions and indicated a major dependence on the reaction stoichiometry with maximum saturation HBpin concentration limits reminiscent of enzymatic kinetics (Figure 3c). This suppression of reaction rate at high HBpin concentrations could be attributed to the competing formation of the previously reported iron‐dihydride complex **B**.^[^ ^24^ ^]^ At Low HBpin concentrations a pseudo first order dependency is observed suggesting that HBpin is required up to and including in the RDS.

**Scheme 2:**
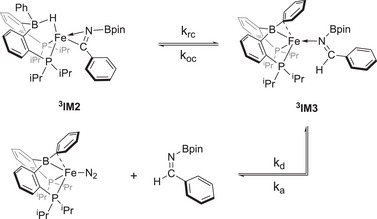
Potential mechanism for the equilibrium isotope effect (EIE).

A comparison of the pseudo‐first order rate constants for hydroboration of benzonitrile in which HBpin was exchanged for DBpin provided a kinetic isotope effect (KIE, *k*_H_/*k*_D_) of 0.3. The observation of the inverse KIE (i.e., KIE = *k*_H_/*k*_D_ < 1) for the hydroboration of benzonitrile using **A** implies the existence of an intermediate in a multistep reaction prior to the rate‐determining step (RDS). Specifically, the observed KIE suggests that reductive elimination could be the RDS for this process, see Scheme 2. The reductive elimination (*k*_re_) in this transformation can be considered as a composite of the effect of substituting deuterium for hydrogen on the individual rate constants for the reductive coupling (*k*_rc_) to form the boryl‐imine complex (**^3^IM3**), the oxidative addition (*k*_OA_) of the C─H bond to regenerate the borohydride‐ferratoimine complex (**^3^IM2**), and the dissociation (*k*_d_) of the boryl‐imine. If the dissociation of the boryl‐imine from the iron center is facile, the isotope effect on R.E will therefore be dominated by the ratio of the reductive coupling step to the oxidative addition and this ratio can be considered as being identical to the equilibrium isotope (EIE) for the interconversion of **^3^IM2** and **^3^IM3**. Based on the inverse EIE observed, this is suggestive that the deuterium prefers to be located on the iminyl carbon over the iron and that the *k*_OA_ of the C─D bond back to **^3^IM2** would be much slower compared to the hydrogen isotopologue (Scheme 2).^[^ ^28^ ^]^

Further insight into the nature of the mechanism was obtained from variable temperature kinetic studies. The activation parameter for the hydroboration of benzonitrile catalyzed by **A** was determined through Arrhenius and Eyring analyses for the reaction performed at four temperatures (313, 323, 333, and 343 K) under standard conditions for the hydroboration of benzonitrile (0.33 M) with HBpin (0.67 M) and **A** (0.003 M). Obtained values for ΔS^‡^, ΔH^‡^, and E_a_ are 8.36 (±0.64) cal K^−1^ mol^−1^, 27.57 (±0.2) kcal mol^−1^, and 28.22 (±0.2) kcal mol^−1^, respectively (Figure 3). Whilst the hydroboration is enthalpically disadvantaged, the catalysis benefits from a slightly positive (effectively zero) activation entropy. This small entropic term is suggestive of a dissociative process being in operation prior to the rate‐limiting step.

### 2.4 DFT Studies

**Figure 4:**
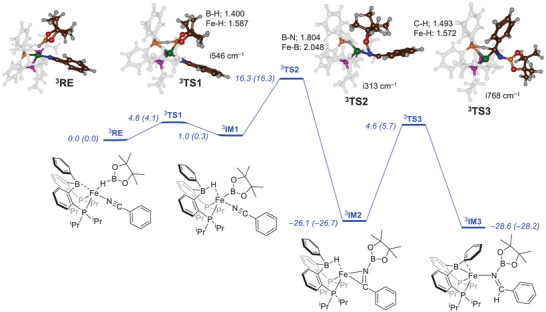
UB3LYP‐GD3BJ/BS2//UB3LYP‐GD3BJ/BS1 calculated potential energy profile for the first hydroboration step of benzonitrile by HBpin on complex C in the triplet spin state. The spin state is identified with superscript. Enthalpies and free energies (in parenthesis) are reported in kcal mol^−1^, obtained at 323 K, and contain zero‐point, solvent, entropic, and thermal corrections. Optimized geometries give bond lengths in Å, bond angles in degrees, and the imaginary frequency in cm^−1^.

To further probe the reaction mechanism, DFT calculations were undertaken on the catalytic cycle of the nitrile hydroboration at the unrestricted B3LYP‐GD3BJ/BS2//UB3LYP‐GD3BJ/BS1 level of theory with a self‐consistent reaction field included with a dielectric constant mimicking benzene. This level of theory was shown previously to reproduce experimentally determined free energies of activation to within a few kcal mol^−1^ and predict the correct selectivities.^[^ ^29^ ^]^ We started from the crystal structure coordinates of complex **C** and manually added HBpin to the structure to obtain **C**•HBpin, designated structure **RE**. A geometry optimization resulted in a triplet spin ground state with the singlet spin much higher in free energy at ΔG = 12.2 kcal mol^−1^. This is in agreement with the experimental data reported above of a triplet spin ground state of **C**. The optimized geometry (see Figure 4) binds HBpin to iron through the hydride with a Fe─H distance of 2.246 Å, while the H─B distance remains short at 1.196 Å. The optimized geometry of ^3^ **RE** compares well with the crystal structure coordinates displayed in Figure 2b with Fe─P distances of 2.323 and 2.301 Å as compared to 2.312 and 2.279 Å, whereas the Fe─B distance was 2.308 Å (DFT) versus 2.318 Å (crystal structure).

**Scheme 3:**
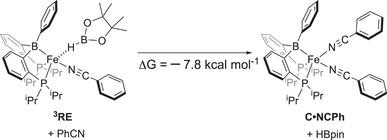
On‐ versus Off‐Cycle Resting State Formation with Calculated Reaction Free Energy.

Subsequently, we investigated the off‐cycle resting state that has two benzonitrile molecules bound to iron rather than one benzonitrile and one HBPin molecule as in **C**. The off‐cycle resting state is more stable than **C** by ΔG = −7.8 kcal mol^−1^, see Scheme 3. These values imply that under high concentrations of benzonitrile, the reaction will be slowed down as the equilibrium will be pushed to the right‐hand‐side. This result is in agreement with experimental observation of an inverse first‐order kinetics in substrate.

We tested several reaction pathways for nitrile hydroboration from complex **C**, namely starting with C─N bond formation and H‐transfer from HBpin as well as a pathway with an initial iron‐phosphine bond cleavage. The latter pathway and structures, however, were substantially higher in energy than complexes that retained the Fe─P bond. The C─N cleavage pathway was found to be high in energy and did not lead to a stable local minimum. We then investigated hydride transfer from HBpin to the metal or its ligands and attempted to form a bond with either iron or the boron atom of the diphosphinoborane ligand.

The lowest energy pathway led to hydride transfer from HBpin to the boron atom of the diphosphinoborane ligand via transition state **^3^TS1** with a free energy of activation of ΔG^‡^ = 4.1 kcal mol^−1^.

The optimized geometry is shown in Figure 4 and displays a large imaginary frequency of i546 cm^−1^ for the B─H─B stretch vibration, which should have resulted in a large kinetic isotope effect (KIE) if this barrier was rate‐determining. However, the barrier is small and the H─Bpin bond cleavage is not rate‐determining in the process. This computational result matches the experimental observation of an inverse isotope effect for the reaction of HBpin versus DBpin with **C**. Structurally, the hydride transfer is relatively central with long B─H distances toward its donor and acceptor atoms of 1.400 and 1.830 Å, respectively. The hydride is relatively close to iron in the transition state (at a distance of 1.587 Å), which shows that the iron atom guides the H─B cleavage step. After the transition state, the system relaxes to structure **^3^IM1**, which has the hydride bridged between the borane group and iron. Subsequently, the boron atom of the Bpin group attacks the nitrile group via transition state **^3^TS2** to form the N─B bond. This step has a significant barrier and a ΔG^‡^ = 16.6 kcal mol^−1^ with respect to **^3^IM1** but leads through a large exothermicity to structure **^3^IM2**. Consequently, the Bpin transfer to the nitrile will be irreversible. However, the final hydride transfer incurs a significant barrier of ΔG^‡^ = 32.4 kcal mol^−1^ with respect to **^3^IM2** for hydride transfer to the carbon atom of the nitrile. As such the highest barrier obtained from the DFT calculations is via **^3^TS3** and will be rate‐determining, which explains the inverse KIE observed experimentally. The barrier is in reasonable agreement with the experimentally determined free energy of activation of ΔG^‡^ = 28.2 kcal mol^−1^ reported above in Figure 4. Previously, for hydrogen atom abstraction barriers by metal‐peroxo complexes using the same methods and approaches we also overestimated experimental free energies of activation by 3–4 kcal mol^−1^, hence our results are in line with literature data.^[^ ^30^ ^]^

The **^3^TS2** and **^3^TS3** transition state structures are shown in Figure 4, and **^3^TS2** has a small imaginary frequency (i313 cm^−1^) for the C─N stretch vibration, whereas **^3^TS3** displays a somewhat larger imaginary frequency of i768 cm^−1^ for the final hydride transfer. The latter is a relatively small imaginary frequency for hydride transfer as previous studies on hydrogen transfer transition states showed typical values of > i1500 cm^−1^.^[^ ^31^ ^]^ A small imaginary frequency implicates a broad potential energy profile and generally indicates a low KIE for the replacement of H by D atoms. As such, the transition state geometries implicate that the reaction will not proceed with a large KIE in agreement with experimental observation. Indeed, when we replace the transferring hydride with deuteride, the free energy of activation goes up by 0.69 kcal mol^−1^ and the imaginary frequency in the transition state drops from i768 to i565 cm^−1^ resulting in an Eyring KIE of 3.2 and a Wigner KIE of 3.8. These KIE values are relatively small and much smaller than those found for typical hydrogen atom transfer reactions.^[^ ^32^ ^]^ The singlet spin state was also tested but found to be higher in free energy for all structures, see Supporting Information.

To test the effect of the metal catalyst on the reaction mechanism, we also explored the reaction of HBpin with benzonitrile in the absence of the iron complex. The subsequently calculated hydride transfer from HBpin to the nitrogen atom of benzonitrile has a barrier of ΔG^‡^ = 61.4 kcal mol^−1^ with respect to isolated reactants. This barrier is high in free energy and will not be able to proceed at 323 K, hence the metal complex provides a low‐energy transition state for the reaction between HBpin and benzonitrile.

**Figure 5:**
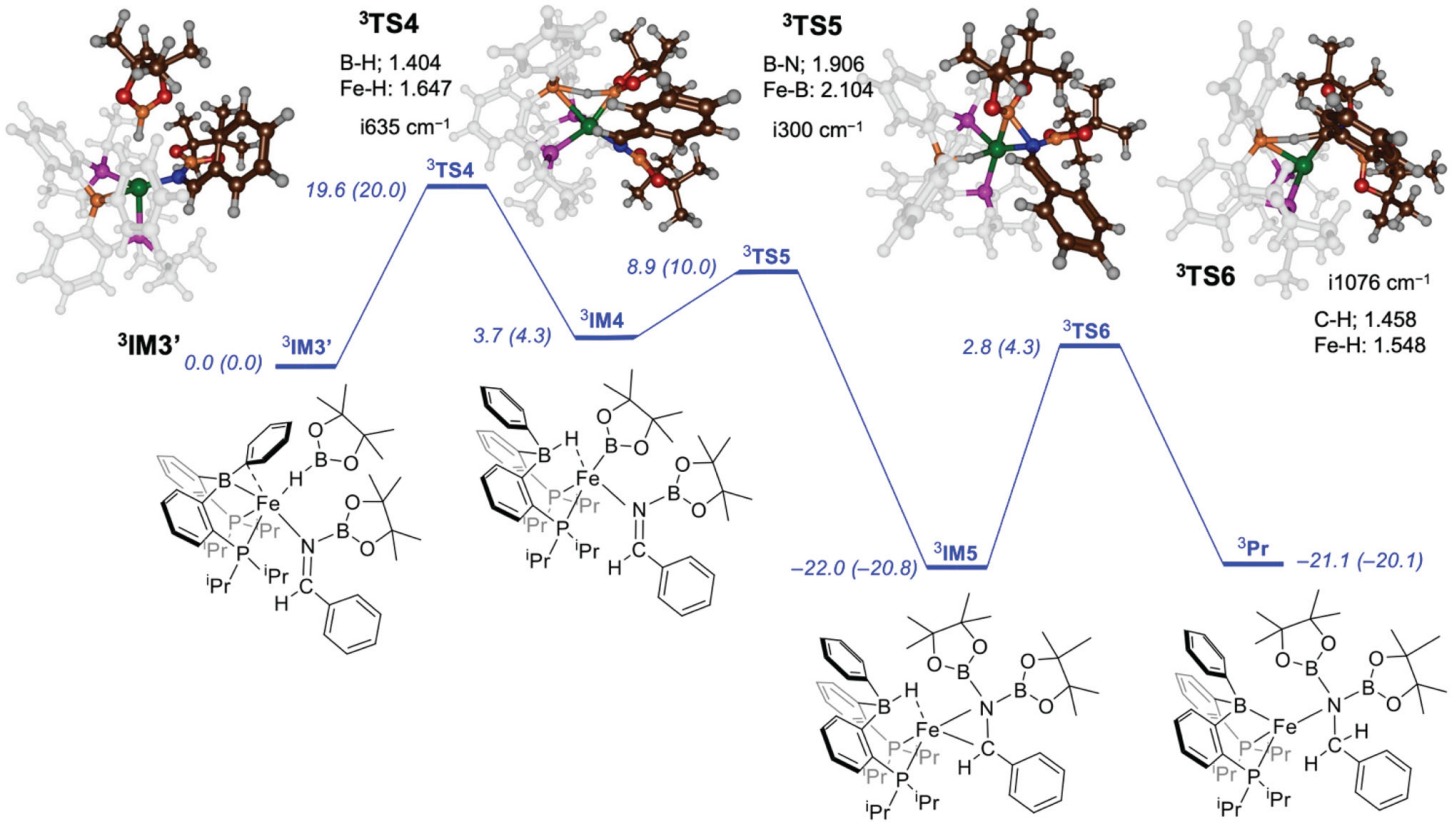
UB3LYP‐GD3BJ/BS2//UB3LYP‐GD3BJ/BS1 calculated potential energy profile for the second hydroboration step of benzonitrile by HBpin in the triplet spin state. The spin state is identified with superscript. Enthalpies and free energies (in parenthesis) are reported in kcal mol^−1^, obtained at 323 K, and contain zero‐point, solvent, entropic, and thermal corrections. Optimized geometries give bond lengths in Å, bond angles in degrees, and the imaginary frequency in cm^−1^.

Next, we took the **^3^IM3** optimized geometry and manually added another HBpin molecule, and optimized the structure as **^3^IM3’**, see Figure 5. The orientation of the second HBpin in the complex **^3^IM3’** is similar to that in **^3^RE**, with the hydride pointing toward iron. The potential energy profile for the second hydroboration step from ^3^ **IM3’** is shown in Figure 5. The first hydride transfer via **^3^TS4** has a free energy of activation of ΔG^‡^ = 20.0 kcal mol^−1^, which is significantly higher in free energy than that reported above for **^3^TS1**. This likely stems from the stereochemical interactions around the iron center that make the borane less accessible than in cycle 1 above. The transition state is highly central with long B─H and H─Bpin distances of 1.403 and 1.736 Å, respectively. The imaginary frequency for hydride transfer is small, i.e. i634 cm^−1^, which is of similar value as the one seen for **^3^TS1**.

After the hydride transfer, the system relaxes to **^3^IM4** which is slightly higher in free energy than ^3^ **IM3’** by ΔG = 4.3 kcal mol^−1^. The subsequent barrier for B─N bond formation has a barrier of ΔG^‡^ = 10.0 kcal mol^−1^ for **^3^TS5** with respect to **^3^IM3’**. The transition state has a small imaginary frequency of i300 cm^−1^ and has similar features as **^3^TS2**. The final stage leads to hydride transfer from the borane group to the carbon atom of the substrate to give the final product **^3^Pr**. This barrier is ΔG = 25.1 kcal mol^−1^ in free energy with respect to **^3^IM5**. Consequently, the hydride transfer is calculated to be rate‐determining.

**Scheme 4:**
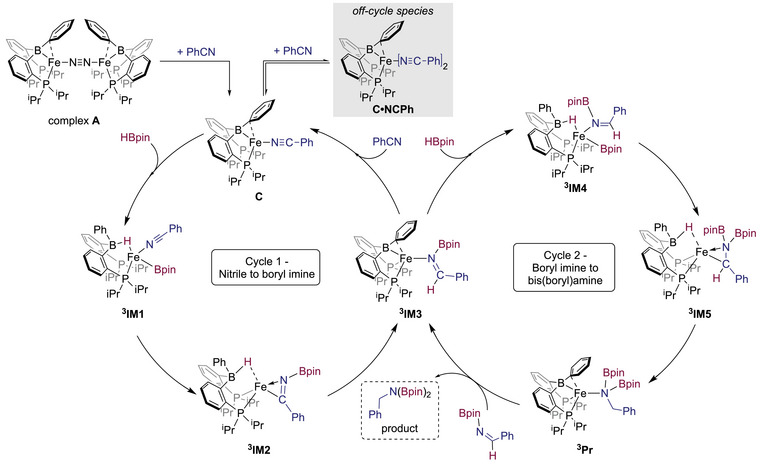
Proposed catalytic cycle based on the kinetic and computational data.

Based on the above experimental and computational results we propose a possible catalytic mechanism using complex **A**, as shown in Scheme 4. For cycle 1, initial coordination of the nitrile to complex **A** furnishes the paramagnetic complex **C,** which is in equilibrium with the off‐cycle species **7**. Addition of HBpin to **C**, results in a cooperative addition of the B─H across the Fe─B interaction, leading to complex **^3^IM1**. This process is a formal two electron oxidation of the iron center. Boryl migration from iron to the nitrile nitrogen affords the iron‐bound *N*‐borylated imine (**^3^IM3**), followed by a subsequent C─H reductive elimination and ligand substitution to regenerate complex **C** and the *N*‐borylimine to enter cycle 2. Cycle 2 proceeds from **^3^IM3** via B─H bond addition across the Fe─B interaction to generate **^3^IM4**. This species again undergoes a boryl migration to the nitrogen followed a final C─H bond reductive elimination to form the *bis*(boryl)amine adduct, which upon release of the product addition of another equivalent of *N*‐borylimine re‐enters the catalytic cycle. Upon consumption of all the substrate, Complex **A** is regenerated as observed experimentally. Thus, the stoichiometric, kinetic, and computational studies, propose a mechanistic scenario that differs from the related Fe─Si system reported by Hashimoto and Komuro. In their study, a low‐valent iron(0)─Si complex is reported to react with both the nitrile and the HBpin via a B─H oxidative addition and subsequent dissociation of the *N*‐silylimine fragment to deliver a corresponding terminal Fe(II)─hydride species. This species was then reported to undergo a hydride migration onto the ligated nitrile and a subsequent B─N coupling.^[^ ^23^ ^]^ Our study, however, implicates that a different mechanistic pathway is in operation here, where after initial nitrile binding, and the generation of a bridging hydride between the iron and boron in the ligand via a cooperative B─H bond oxidative addition occurs, which is nonrate limiting. Based on the computational study, a boryl migration proceeds prior to the rate determining C─H bond reductive elimination step.

## 3 Conclusion

In summary, complex **A** is demonstrated to be an efficient precatalyst for the hydroboration of nitriles using HBpin as the terminal reductant. The functional group tolerance is comparable to and in some cases surpasses the current state‐of‐the‐art in iron‐ catalyzed hydroboration chemistry. Stoichiometric studies of complex **A** with benzonitrile furnish a new paramagnetic complex **C**, which has been spectroscopically and crystallographically characterized. Combined computational, stoichiometric, and kinetic studies of the benzonitrile hydroboration revealed a first‐order dependency in complex **A**, saturation kinetics in HBpin and an inverse first‐order dependency in nitrile, suggesting inhibition by nitrile. An inverse KIE (*k*_H_/*k*_D_ = 0.3) reveals that B─H bond activation is not the rate‐limiting step and that this value can be attributed to an equilibrium isotope effect, as supported by DFT calculations, suggestive of the C─H bond in the boryl imine intermediate (**^3^IM3**) being more prone to undergoing the reversible C─H oxidative addition back to complex **^3^IM2** compared to the corresponding C─D addition.

## Supporting Information

Deposition Number(s) 2340635 contain(s) the supplementary crystallographic data for this paper. These data are provided free of charge by the joint Cambridge Crystallographic Data Centre and Fachinformationszentrum Karlsruhe “http://www.ccdc.cam.ac.uk/structures”> Access Structures service. The authors have cited additional references within the Supporting Information.^[^ ^33^, ^34^, ^35^, ^36^, ^37^, ^38^, ^39^, ^40^, ^41^, ^42^, ^43^ ^]^

## Conflict of Interest

The authors declare no conflict of interest.

## Supporting information



Supporting Information

## Data Availability

The data that support the findings of this study are available in the supplementary material of this article.
