# Supplementary material for: Nitrile Hydroboration by Cooperative Iron Catalysis: An Experimental and Computational Study
Source: Chemistry. 2025 Jul 2;31(41):e202501782. doi: 10.1002/chem.202501782 (PMC12284615; doi:10.1002/chem.202501782)
Supplement: Supplementary file 1 — Supporting Information [file CHEM-31-e202501782-s001.pdf]

Supporting Information

Nitrile Hydroboration by Cooperative Iron Catalysis: An Experimental and Computational Study

Laura A. Grose,[a] Yi Zhang,[b],[c] Samuel Oultram,[a] Ryan J. Schwamm,[a] Sam. P. de Visser*[b],[c] and Darren Willcox*[a]

[a] L. G. Grose, S. Oultram, R. J. Schwamm, Dr D. Willcox
Department of Chemistry
The University of Manchester
Oxford Road, Manchester, M13 9PL, United Kingdom

[b] Y. Zhang, Dr. S. P. de Visser
Department of Chemical Engineering
The University of Manchester
Oxford Road, Manchester, M13 9PL, United Kingdom

[c] Y. Zhang, Dr. S. P. de Visser
Manchester Institute of Biotechnology,
The University of Manchester
131 Princess Street, Manchester M1 7DN, United Kingdom

Corresponding authors - DW: darren.willcox@manchester.ac.uk; SPdV: sam.devisser@manchester.ac.uk

Table of Contents

Experimental procedures 3

Table of optimizations 3

General procedure 1: hydroboration of nitriles 4

Characterization data of nitrile hydroborated products 4

Synthesis of [{(^{iPr}DPB^{Ph})Fe(NCPh)}] (C) 7

Infrared spectroscopic data 7

General crystallographic methods 8

Solution magnetic susceptibility measurements 10

Kinetic studies on benzonitrile 11

Pre-catalyst rate order assessment 11

Benzonitrile rate order assessment 12

HBpin rate order assessment 13

Temperature dependance graphs 14

Kinetic isotope effect measurements 15

Data for investigating the inverse first-order in nitrile 15

NMR spectra 17

Computational Methods 40

Computational data 41

Cartesian Coordinates of Optimized Geometries 46

Raw Kinetic Data 72

References 93

Experimental procedures

All commercially purchased starting materials were used as received unless otherwise stated. All manipulations were performed using standard Schlenk techniques or in an MBraun glovebox, under an atmosphere of dry N_2. Dry solvents (THF, Et_2O, pentane, C_6H_6, CH_3CN, toluene and CH_2Cl_2) were obtained using Innovative Technologies anhydrous engineering solvent purification systems, subsequently degassed and left over 3 Å activated sieves before being transferred to a potassium mirror, except for CH_2Cl_2. All other solvents used were of HPLC grade, unless otherwise stated. Solvents removed under "reduced pressure" were by rotary evaporation and "*in vacuo*" under high vacuum via Schlenk line. THF-d_8, C_6D_6 and $CDCl_3$ were dried over activated 3 Å molecular sieves and degassed by sparging with dry N_2. All glassware and stirrer bars were flame dried with a blowtorch under a vacuum before use. Column chromatography: Flash column chromatography with silica gel 60. Mixtures of solvents used are noted in brackets.

^{1}H, ^{11}B, $^{13}C\{^{1}H\}$ and $^{31}P\{^{1}H\}$ NMR spectra were recorded on a Bruker Advance III HD 400 spectrometer (operating frequencies: 399.78 MHz, 128.25 MHz, 100.53 MHz and 161.83 ppm, respectively). ^{1}H and $^{13}C\{^{1}H\}$ NMR chemical shifts were internally referenced to the residual solvent resonances ($CDCl_3$ (chloroform-d): ^{1}H δ = 7.26 ppm, $^{13}C\{^{1}H\}$ δ = 77.16 ppm; C_6D_6 (benzene-d_6): ^{1}H δ = 7.16 ppm, $^{13}C\{^{1}H\}$ δ = 128.02 ppm), THF-d_8 (tetrahydrofuran-d_8): ^{1}H δ = 3.58, 1.73 ppm, $^{13}C\{^{1}H\}$ δ = 67.57, 25.37 ppm). NMR samples were prepared under an inert atmosphere in 5 mm J. Youngs NMR tubes. Data was analysed using MestReNova V14.0.0 software. ATR-IR spectra were recorded as microcrystalline powders using a Bruker Tensor 27 spectrometer.

Table of optimizations

Table S1: Optimized conditions

Entry	Fe (mol%)	HBpin Equiv	Solvent	Temp (°C)	Time (h)	Yield (%)
1	1	2.0	Neat	50	3	63
2	1	2.0	Diethyl ether	50	3	70
3	1	2.0	THF	50	3	80
4	1	2.0	Toluene	50	3	68
5	1	2.0	C_6D_6	50	3	95
6	2	2.0	C_6D_6	50	2	83
7	5	2.0	C_6D_6	50	2	90
8	1	2.0	C_6D_6	R.T	3	3
9	1	2.2	C_6D_6	50	3	95
10	1	2.4	C_6D_6	50	3	96
11	1	3.0	C_6D_6	50	3	95

General procedure 1: hydroboration of nitriles

In a nitrogen filled glovebox, an oven dried J-Youngs NMR tube was charged with [{(^{iPr}DPB^{Ph})Fe}$_2$(μ-1,2-N$_2$)] (2.05 x 10^{-3} mmol), C_6D_6 (0.6 mL), HBpin (0.410 mmol), substrate (0.205 mmol) and toluene (0.205 mmol) as internal standard for NMR quantification. The reaction mixture was added to an oil bath (50 °C) for 1–6 h, yield was calculated by ^{1}H NMR spectroscopy. Volatiles were removed *in vacuo*, the mixture was suspended in Et_2O and filtered through a short plug of Celite® in a glove box, and volatiles were removed *in vacuo* to reveal isolated product.

Characterization data of nitrile hydroborated products

Spectroscopic data is in accordance with the literature.[1]

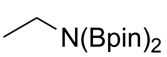

3a, *N*-ethyl-4,4,5,5-tetramethyl-*N*-(4,4,5,5-tetramethyl-1,3,2-dioxaborolan-2-yl)-1,3,2-dioxaborolan-2-amine was prepared according to general procedure 1 using acetonitrile (11 *μL*, 0.205 mmol) and HBpin (60 μL, 0.410 mmol) at 50 °C for 3 h to furnish product **3a** (56 mg, 0.188 mmol, 92%). **^{1}H NMR** (400 MHz, $CDCl_3$) δ 3.04 (q, *J* = 7.0 Hz, 2H), 1.22 (s, 24H), 1.01 (t, *J* = 7.1 Hz, 3^{2}H). **^{13}C{^{1}H} NMR** (101 MHz, $CDCl_3$) δ 82.1, 38.6, 24.6, 18.7 ppm. **^{11}B NMR** (128 MHz, $CDCl_3$) δ 25.8 ppm.

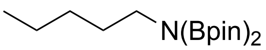

3b, *N*-butyl-4,4,5,5-tetramethyl-*N*-(4,4,5,5-tetramethyl-1,3,2-dioxaborolan-2-yl)-1,3,2-dioxaborolan-2-amine was prepared according to general procedure 1 using butyronitrile (14 *μL*, 0.205 mmol) and HBpin (60 μL, 0.410 mmol) at 50 °C for 3 h to furnish product **3b** (59 mg, 0.182 mmol, 89%).**^{1}H NMR** (400 MHz, $CDCl_3$) δ 3.00 (t, *J* = 6.8 Hz, 2H), 1.40–1.30 (m, 2H), 1.20 (s, 26H), 0.86 (t, *J* = 7.2 Hz, 3H). **^{13}C{^{1}H} NMR** (101 MHz, $CDCl_3$) δ 82.0, 43.3, 35.3, 24.5, 19.6, 14.1 ppm. **^{11}B NMR** (128 MHz, $CDCl_3$) δ 25.8 ppm.

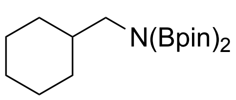

3c, *N*-(cyclohexylmethyl)-4,4,5,5-tetramethyl-*N*-(4,4,5,5-tetramethyl-1,3,2-dioxaborolan-2-yl)-1,3,2-dioxaborolan-2-amine was prepared according to general procedure 1 using cyclohexane carbonitrile (24 *μL*, 0.205 mmol) and HBpin (60 μL, 0.410 mmol) at 50 °C for 5 h to furnish product **3c** (59 mg, 0.162 mmol, 79%). **^{1}H NMR** (400 MHz, $CDCl_3$) δ 2.85 (d, *J* = 7.0 Hz, 2H), 1.67–1.63 (m, 4H), 1.33–1.10 (m, 29H), 0.88–0.79 (m, 2H). **^{13}C{^{1}H} NMR** (101 MHz, $CDCl_3$) δ 82.0, 49.7, 40.5, 30.6, 26.8, 26.2, 24.5 ppm. **^{11}B NMR** (128 MHz, $CDCl_3$) δ 25.8 ppm.

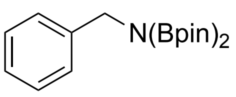

3d, *N*-benzyl-4,4,5,5-tetramethyl-*N*-(4,4,5,5-tetramethyl-1,3,2-dioxaborolan-2-yl)-1,3,2-dioxaborolan-2-amine was prepared according to general procedure 1 using benzonitrile (21 *μL*, 0.205 mmol) and HBpin (60 μL, 0.410 mmol) at 50 °C for 3 h to furnish product **3d** (70 mg, 0.195 mmol, 95%). **^{1}H NMR** (400 MHz, $CDCl_3$) δ 7.29–7.22 (m, 2H), 7.18 (td, *J* = 6.8, 1.9 Hz, 2H), 7.13–7.07 (m, 1H), 4.18 (s, 2H), 1.15 (s, 18H). **^{13}C{^{1}H} NMR** (101 MHz, $CDCl_3$) δ 143.1, 127.8, 127.5, 126.1, 82.3, 47.3, 24.5 ppm. **^{11}B NMR** (128 MHz, $CDCl_3$) δ 26.0 ppm.

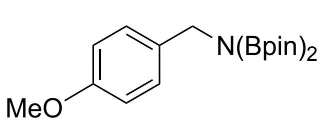

3e, *N*-(4-methoxybenzyl)-4,4,5,5-tetramethyl-*N*-(4,4,5,5-tetramethyl-1,3,2-dioxaborolan-2-yl)-1,3,2-dioxaborolan-2-amine was prepared according to general procedure 1 using 4-methoxybenzonitrile (27 *mg*, 0.205 mmol) and HBpin (60 µL, 0.410 mmol) at 50 °C for 3 h to furnish product **3e** (71 mg, 0.182 mmol, 89%). ^{1}**H NMR** (400 MHz, $CDCl_3$) δ 7.26 (d, *J* = 8.7 Hz, 2H), 6.80 (d, *J* = 8.7 Hz, 2H), 4.18 (s, 2H), 3.80 (s, 3H), 1.22 (s, 24H). ^{13}**C{^{1}H} NMR** (101 MHz, $CDCl_3$) δ 158.0, 135.5, 128.9, 113.2, 82.3, 55.3, 46.6, 24.6 ppm. ^{11}**B NMR** (128 MHz, $CDCl_3$) δ 26.0 ppm.

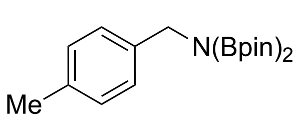

3f, 4,4,5,5-tetramethyl-*N*-(4-methylbenzyl)-*N*-(4,4,5,5-tetramethyl-1,3,2-dioxaborolan-2-yl)-1,3,2-dioxaborolan-2-amine was prepared according to general procedure 1 using *p*-tolunitrile (24 *mg*, 0.205 mmol) and HBpin (60 µL, 0.410 mmol) at 50 °C for 3 h to furnish product **3f** (71 mg, 0.190 mmol, 93%). ^{1}**H NMR** (400 MHz, $CDCl_3$) δ 7.20 (d, *J* = 8.0 Hz, 2H), 7.05 (d, *J* = 8.0 Hz, 2H), 4.20 (s, 2H), 2.31 (s, 3H), 1.21 (s, 24H). ^{13}**C{^{1}H} NMR** (101 MHz, $CDCl_3$) δ 140.1, 135.5, 128.5, 127.5, 82.3, 46.9, 24.6, 21.1 ppm. ^{11}**B NMR** (128 MHz, $CDCl_3$) δ 26.0 ppm.

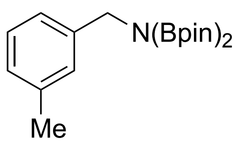

3g, 4,4,5,5-tetramethyl-*N*-(3-methylbenzyl)-*N*-(4,4,5,5-tetramethyl-1,3,2-dioxaborolan-2-yl)-1,3,2-dioxaborolan-2-amine was prepared according to general procedure 1 using *m*-tolunitrile (24 mg, 0.205 mmol) and HBpin (60 µL, 0.410 mmol) at 50 °C for 3 h to furnish product **3g** (70 mg, 0.189 mmol, 92%). ^{1}**H NMR** (400 MHz, $CDCl_3$) δ 7.18–7.06 (m, 3H), 7.02–6.90 (m, 1H), 4.21 (s, 2H), 2.31 (s, 3H), 1.21 (s, 24H). ^{13}**C{^{1}H} NMR** (101 MHz, $CDCl_3$) δ 143.0, 137.3, 128.4, 127.8, 126.8, 124.6, 82.4, 47.2, 24.6, 21.5. ^{11}**B NMR** (128 MHz, $CDCl_3$) δ 26.0.

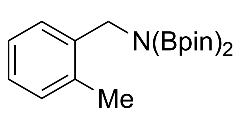

3h, 4,4,5,5-tetramethyl-*N*-(2-methylbenzyl)-*N*-(4,4,5,5-tetramethyl-1,3,2-dioxaborolan-2-yl)-1,3,2-dioxaborolan-2-amine was prepared according to general procedure 1 using *o*-tolunitrile (24 mg, 0.205 mmol) and HBpin (60 µL, 0.410 mmol) at 50 °C for 3 h to furnish product **3h** (67 mg,0.180 mmol, 88%). ^{1}**H NMR (**400 MHz, C_6D_6) δ 7.64 (d, *J* = 7.7 Hz, 1H), 7.24 (t, *J* = 7.5 Hz, 1H), 7.08 (t, *J* = 7.4 Hz, 1H), 6.99 (d, *J* = 7.5 Hz, 1H), 4.60 (s, 2H), 2.13 (s, 3H), 1.03 (s, 24H). ^{13}**C{^{1}H} NMR** (101 MHz, C_6D_6) δ 141.2, 135.4, 130.5, 126.2, 126.0, 125.7, 82.6, 45.43, 24.7, 19.1. ^{11}**B NMR** (128 MHz, C_6D_6) δ 26.5.

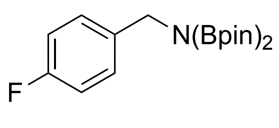

3i, *N*-(4-fluorobenzyl)-4,4,5,5-tetramethyl-*N*-(4,4,5,5-tetramethyl-1,3,2-dioxaborolan-2-yl)-1,3,2-dioxaborolan-2-amine was prepared according to general procedure 1 using 4-fluorobenzonitrile (26 *mg*, 0.205 mmol) and HBpin (60 µL, 0.410 mmol) at 50 °C for 3 h to furnish product **3i** (70 mg, 0.182 mmol, 90%). ^{1}**H NMR** (400 MHz, $CDCl_3$) δ 7.28–7.22 (m, 2H), 6.97–6.85 (m, 2H), 4.16 (s, 2H), 1.18 (s, 24H). ^{13}**C{^{1}H}** NMR (101 MHz, $CDCl_3$) δ 161.6 (d, *J* = 243.1 Hz), 138.9, 129.2 (d, *J* = 7.8 Hz), 114.5 (d, *J* = 21.0 Hz), 82.5, 46.6, 24.6 ppm. ^{11}**B NMR** (128 MHz, $CDCl_3$) δ 25.9 ppm.

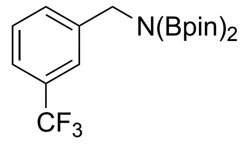

3j, 4,4,5,5-tetramethyl-*N*-(4,4,5,5-tetramethyl-1,3,2-dioxaborolan-2-yl)-*N*-(3-(trifluoromethyl)benzyl)-1,3,2-dioxaborolan-2-amine was prepared according to general procedure 1 using 3-(trifluoromethyl)benzonitrile (27 *μL*, 0.205 mmol) and HBpin (60 μL, 0.410 mmol) at 50 °C for 4 h to furnish product **3j** (75 mg, 0.176 mmol, 86%). **^{1}H NMR** (400 MHz, $CDCl_3$) δ 7.64 (s, 1H), 7.50 (d, *J* = 7.6 Hz, 1H), 7.43 (d, *J* = 8.1 Hz, 1H), 7.35 (t, *J* = 7.6 Hz, 1H), 4.26 (s, 2H), 1.20 (s, 19H). **^{13}C{^{1}H} NMR** (101 MHz, $CDCl_3$) δ 144.1, 130.2 (q, *J* = 32.0 Hz), 128.3, 125.9, 124.9 (q, *J* = 3.9 Hz), 123.1 (q, *J* = 3.9 Hz), 82.6, 47.1, 24.6 ppm.**^{11}B NMR** (128 MHz, $CDCl_3$) δ 25.6 ppm. **HRMS** (ESI) m/z cald [M] 427.08, found $[M+Na]^-$ 450.22.

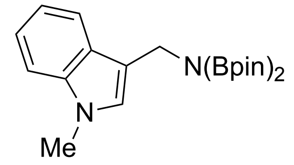

3k, 4,4,5,5-tetramethyl-*N*-((1-methyl-1H-indol-3-yl)methyl)-*N*-(4,4,5,5-tetramethyl-1,3,2-dioxaborolan-2-yl)-1,3,2-dioxaborolan-2-amine was prepared according to general procedure 1 using 1-methyl-1H-indole-3-carbonitrile (32 *μL*, 0.205 mmol) and HBpin (60 μL, 0.410 mmol) at 50 °C for 3 h to furnish product **3k** (77 mg, 0.187 mmol, 91%). **^{1}H NMR** (400 MHz, C_6D_6) δ 8.36–8.32 (m, 1H), 7.31–7.27 (m, 2H), 7.10–7.07 (m, 2H), 4.86 (s, 2H), 3.05 (s, 3H), 1.08 (s, 24H). **^{13}C NMR** (101 MHz, C_6D_6) δ 137.5, 121.7, 121.0, 118.9, 117.6, 109.2, 82.5, 38.9, 31.9, 24.9. **^{11}B NMR** (128 MHz, C_6D_6) δ 26.4. **HRMS** (ESI) m/z cald [M] 412.14, found [M] 412.27

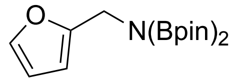

3l, *N*-(furan-2-ylmethyl)-4,4,5,5-tetramethyl-*N*-(4,4,5,5-tetramethyl-1,3,2-dioxaborolan-2-yl)-1,3,2-dioxaborolan-2-amine was prepared according to general procedure 1 furan-2-carbonitrile (19 *μL*, 0.205 mmol) and HBpin (60 μL, 0.410 mmol) at 50 °C for 6 h to furnish product **3l** (48 mg, 0.137 mmol, 67%). **^{1}H NMR** (400 MHz, C_6D_6) δ 7.52 (dd, *J* = 1.6, 0.8 Hz, 1H), 7.19 (t, *J* = 1.7 Hz, 1H), 6.59 (dd, *J* = 1.8, 0.8 Hz, 1H), 4.38 (s, 2H), 1.03 (s, 24H). **^{13}C{^{1}H} NMR** (101 MHz, C_6D_6) δ 142.7, 140.4, 127.6, 111.4, 82.6, 38.7, 24.8. **^{11}B NMR** (128 MHz, C_6D_6) δ 26.3.

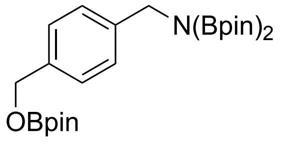

3m, 4,4,5,5-tetramethyl-N-(4,4,5,5-tetramethyl-1,3,2-dioxaborolan-2-yl)-N-(4-(((4,4,5,5-tetramethyl-1,3,2-dioxaborolan-2-yl)oxy)methyl)benzyl)-1,3,2-dioxaborolan-2-amine was prepared according to general procedure 1 using 4-formylbenzonitrile (26.9 mg, 0.205 mmol) and HBpin (89 μL, 0.615 mmol) at 50 °C for 3 h to furnish product **3m** (103 mg, 0.199 mmol, 97%). **^{1}H NMR** (400 MHz, $CDCl_3$) δ 7.24 (d, J = 9.3 Hz, 4H), 4.88 (s, 2H), 4.21 (s, 2H), 1.25 (s, 12H), 1.19 (s, 24H); **^{13}C{^{1}H} NMR** (101 MHz, $CDCl_3$) δ 142.3, 136.9, 127.4, 126.4, 82.9, 82.3, 66.7, 47.0, 24.6, 24.5; **^{11}B NMR** (128 MHz, $CDCl_3$) δ 26.30, 22.40.

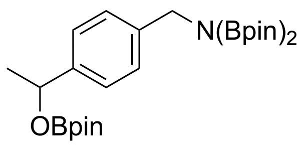

3n, 4,4,5,5-tetramethyl-N-(4,4,5,5-tetramethyl-1,3,2-dioxaborolan-2-yl)-N-(4-(1-((4,4,5,5-tetramethyl-1,3,2-dioxaborolan-2-yl)oxy)ethyl)benzyl)-1,3,2-dioxaborolan-2-amine was prepared according to general procedure 1 using 4-acetylbenzonitrile (29.7 mg, 0.205 mmol) and HBpin (89 μL, 0.615 mmol) at 50 °C for 3 h to furnish product **3n** (105 mg, 0.199 mmol, 97%). **^{1}H NMR** (400 MHz, $CDCl_3$) δ 7.24 (app. d, J = 2.7 Hz, 4H), 5.20 (q, J = 6.4 Hz, 1H), 4.20 (s, 2H), 1.46 (d, J = 6.4 Hz, 3H), 1.23 (s, 6H), 1.20 (s, 6H), 1.19 (s, 24H). **^{13}C{^{1}H} NMR** (101 MHz, $CDCl_3$) δ 142.6, 142.1, 127.5, 125.0, 82.8, 82.5, 72.7, 47.1, 25.6, 24.7, 24.7; ^{11}B NMR (128 MHz, $CDCl_3$) δ 26.36, 22.34.

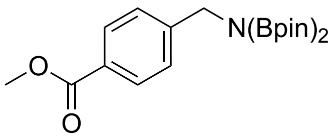

3o, methyl 4-((bis(4,4,5,5-tetramethyl-1,3,2-dioxaborolan-2-yl)amino)methyl)benzoate was prepared according to general procedure 1 using methyl 4-cyanobenzoate (33 mg, 0.205 mmol) and HBpin (60 µL, 0.410 mmol) at 50 °C for 9 h to furnish product **3o** (79.7 mg, 0.191 mmol, 93%). **^{1}H NMR** (400 MHz, $CDCl_3$) δ 7.93 (d, J = 8.4 Hz, 2H), 7.35 (d, J = 8.4 Hz, 2H), 4.27 (s, 2H), 3.89 (s, 3H), 1.19 (s, 24H); **^{13}C{^{1}H} NMR** (101 MHz, $CDCl_3$) δ 167.5, 148.6, 131.0, 129.4, 128.1, 127.4, 82.7, 52.1, 47.3, 24.6; **^{11}B NMR** (128 MHz, $CDCl_3$) δ 26.27; **IR (ATR)** ν_{max} / cm^{-1} 2984, 1713 (C=O), 1613, 1487, 1438, 1403, 1371, 1313, 1273, 1256, 1136, 1107, 1048, 1032.

Synthesis of [{(^{iPr}DPB^{Ph})Fe(NCPh)}] (C)

7 was prepared by dissolving complex **A** (30 mg, 0.058 mmol) and benzonitrile (6 µL, 0.058 mmol) in C_6D_6 (0.6 ml). Immedietly the colour changes from dark red to black at R.T, volatiles were removed *in vacuo* affording **C** as a dark solid. Slow evaporation from a concentrated solution of benzene gave black single crystals suitable for XRD (35 mg, 0.055 mmol, 95%). **^{1}H NMR** (400 MHz, C_6D_6) δ 78.56, 42.40, 21.90, 17.20, 12.95, 9.71, 3.79, 2.01, 0.30, −2.86, −3.30, −4.01, −25.09 ppm. **IR** (cm^{-1}) 2149 (C≡N). APCI m/z calcd for [M] 635.40, found [M] 635.34. Solution magnetic moment (25 °C, C_6D_6) 2.54 μ_B.

Infrared spectroscopic data

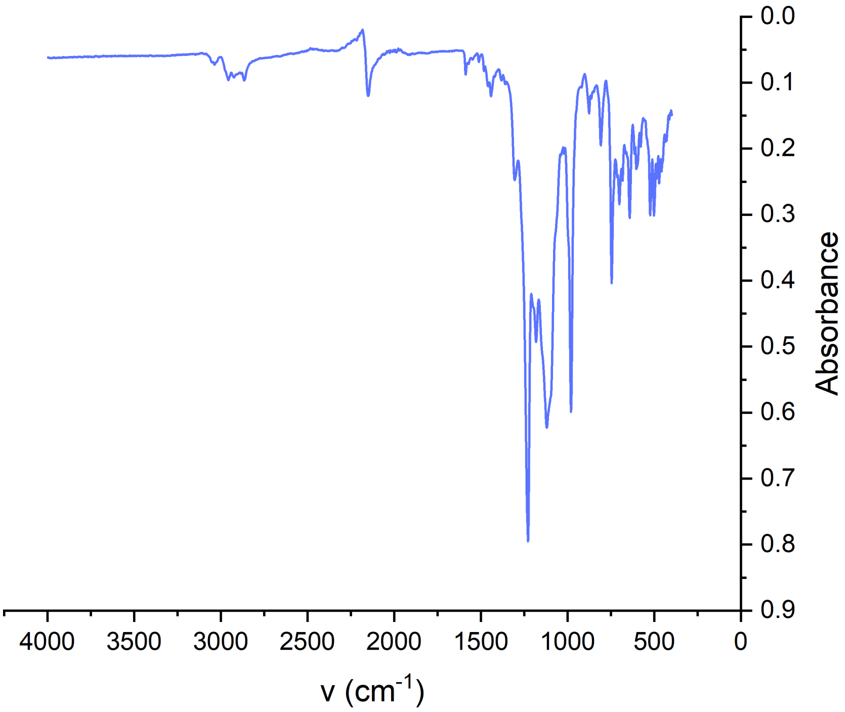

Figure S1: IR spectrum of complex **C**

General crystallographic methods

The crystal data for complex **C** is recorded in XRD experimental parameters. The crystals were obtained from slow evaporation of toluene and benzene, respectively. The crystals were examined using an Agilent Supernova diffractometer, equipped with an Eos CCD area detector and a microfocus source with Mo Kα radiation (λ = 0.71073 Å). Intensities were integrated from data recorded on 1° frames by ω rotation. Data was reduced and processed using CrysAlisPro.[2] The structure was solved using olex2.solve[3] structure solution program using Charge Flipping and refined with olex2.refine with anisotropic displacement parameters for all non-hydrogen atoms, and with constrained riding hydrogen geometries; U_{iso}(H) was set at 1.2 (1.5 for methyl groups) times U_{eq} of the parent atom employed through OLEX2 suit program.[4] For molecular graphics ORTEP-3[5] was employed. These data sets can be obtained free of charge from the Cambridge Crystallographic Data Centre via deposition number 2340633 and 2340635.

Table S2: X-ray crystallography experimental parameters of complex **C**

	Complex **C**
Empirical formula	$C_{43}H_{52}BFeNP_2$
Formula weight	711.516
Temperature/K	99.9(3)
Crystal system	triclinic
Space group	P-1
a/Å	10.2890(3)
b/Å	10.5071(4)
c/Å	18.5666(7)
α/°	100.106(3)
β/°	105.921(3)
γ/°	94.915(3)
Volume/Å^3	1881.17(12)
Z	2
ρ_{calc}g/cm^3	1.256
μ/mm^{-1}	0.517
F(000)	757.5
Crystal size/mm^3	0.472 × 0.307 × 0.234
Radiation	Mo Kα (λ = 0.71073)
2Θ range for data collection/°	3.98 to 58.1
Index ranges	-10 ≤ h ≤ 12, -13 ≤ k ≤ 11, -23 ≤ l ≤ 21
Reflections collected	14258
Independent reflections	8493 [R_{int} = 0.0337, R_{sigma} = 0.0657]
Data/restraints/parameters	8493/87/429
GOF	1.035
R, $_wR^2$ ($F^2 > 2\sigma(F^2)$)	(0.0453, 0.0966)
R, $_wR^2$ (all data)	(0.0622, 0.1067)
Max., min. diff map, e Å^{-3}	1.15/-0.60

[a] Conventional $R = \Sigma||F_o| - |F_c||/\Sigma|F_o|$; $R_w = [\Sigma w(F_o^2 - F_c^2)^2 / \Sigma w(F_o^2)^2]^{1/2}$; $S = [\Sigma w(F_o^2 - F_c^2)^2$ /no. data – no. params)]$^{1/2}$ for all data.

Table S3: Selected bond lengths for complex **C**

Atom	Length/Å
Fe1-P1	2.3121(6)
Fe1-P2	2.2792(6)
Fe1-N1	1.9376(19)
Fe1-C26	2.143(2)
Fe1-C27	2.402(2)
Fe1-B1	2.318(2)
N1-C32	1.154(3)
C33-C32	1.440(3)
C33-C34	1.394(3)
C33-C38	1.393(3)

Table S4: Selected bond angles for complex **C**

Atom	Angle/˚
P2-Fe1-P1	120.97(2)
N1-Fe1-P1	107.56(5)
N1-Fe1-P2	101.11(5)
C13-Fe1-P1	105.05(5)
C13-Fe1-P2	89.95(6)
C13-Fe1-N1	132.94(7)
C14-Fe1-P1	95.81(5)
C14-Fe1-P2	124.97(6)
C14-Fe1-N1	104.93(7)
C14-Fe1-C13	38.34(7)
B1-Fe1-P1	76.88(6)
B1-Fe1-P2	80.66(6)
B1-Fe1-N1	172.96(7)
B1-Fe1-C13	40.03(7)
B1-Fe1-C14	68.84(8)
C32-N1-Fe1	122.57(13)
C36-N1-Fe1	119.53(13)
C36-N1-C32	116.08(17)

Solution magnetic susceptibility measurements

Evans' method for solution-state magnetic susceptibility determination was modified and applied to complex **C**

Equation S1

$$X_M = \frac{3\Delta f}{4\pi F c}$$

Equation S2

$$\mu_{eff} = \sqrt{8(X_M T)}$$

$\Delta f(Hz)$ = change in chemical shift = 128.62 Hz

F = operating frequency of NMR machine = 400,000 Hz

c = concentration of sample = 0.024959281 mol/L,

T = operating temperature of NMR = 298 K

Kinetic studies on benzonitrile

In a nitrogen filled glovebox, an oven dried J-Youngs NMR tube was charged with [{(^{iPr}DPB^{Ph})Fe}$_2$(μ-1,2-N_2)] and benzonitrile dissolved in C_6D_6 (0.5 mL) followed by HBpin and toluene as internal standard for NMR quantification. The tube was immediately removed from the glovebox and placed into an acetonitrile/liquid nitrogen bath to freeze the solution, the mixture was thawed and shaken to mix the contents before being placed in the spectrometer. Kinetic analysis was performed by monitoring the reaction progress by ^{1}H NMR (700 MHz, C_6D_6) analysis at 60 s intervals over 20 minutes at 323.15 K measured against an internal standard toluene.

Pre-catalyst rate order assessment

Table S5: Rate constants measured for determination of complex A order.

Cat A (M)	HBpin (M)	Benzonitrile (M)	k_{obs}	R^2
0.002475	0.666	0.333	$6.66 \times 10^{-5} \pm 2.1 \times 10^{-6}$	0.9972
0.003211	0.666	0.333	$8.62 \times 10^{-5} \pm 3.4 \times 10^{-6}$	0.9981
0.004067	0.666	0.333	$1.17 \times 10^{-4} \pm 1.0 \times 10^{-5}$	0.9988
0.004864	0.666	0.333	$1.34 \times 10^{-4} \pm 2.2 \times 10^{-7}$	0.9964

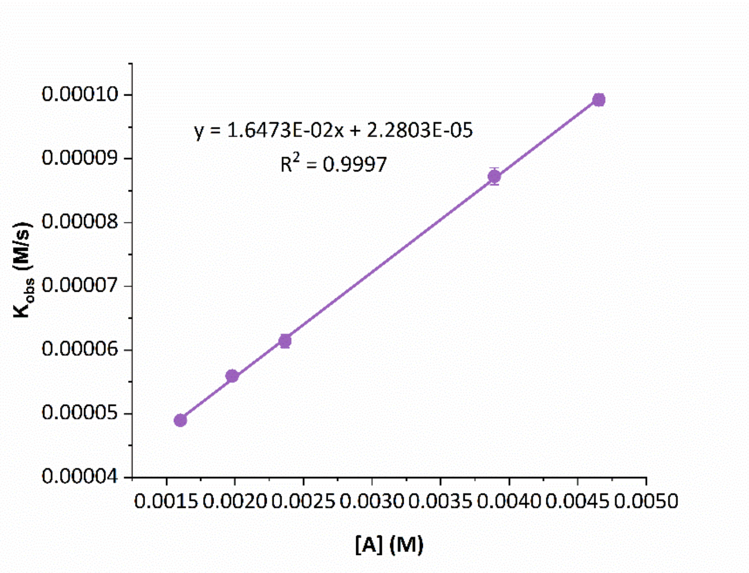

Figure S2: Graph to show concentration of complex A vs. observed rate equates to a first order dependence on the concentration of **A** over the investigated concentration range.

Benzonitrile rate order assessment

Table S6: Rate constants measured for determination of benzonitrile order.

Cat A (M)	HBpin (M)	Benzonitrile (M)	k_{obs}	R^2
0.00333	0.666	0.061882	$5.28 \times 10^{-4} \pm 3.70 \times 10^{-7}$	0.9986
0.00333	0.666	0.123764	$2.39 \times 10^{-4} \pm 3.22 \times 10^{-6}$	0.9981
0.00333	0.666	0.185646	$1.64 \times 10^{-4} \pm 1.93 \times 10^{-6}$	0.9952
0.00333	0.666	0.247209	$1.27 \times 10^{-4} \pm 6.24 \times 10^{-6}$	0.9926
0.00333	0.666	0.30941	$1.01 \times 10^{-4} \pm 8.46 \times 10^{-6}$	0.9988

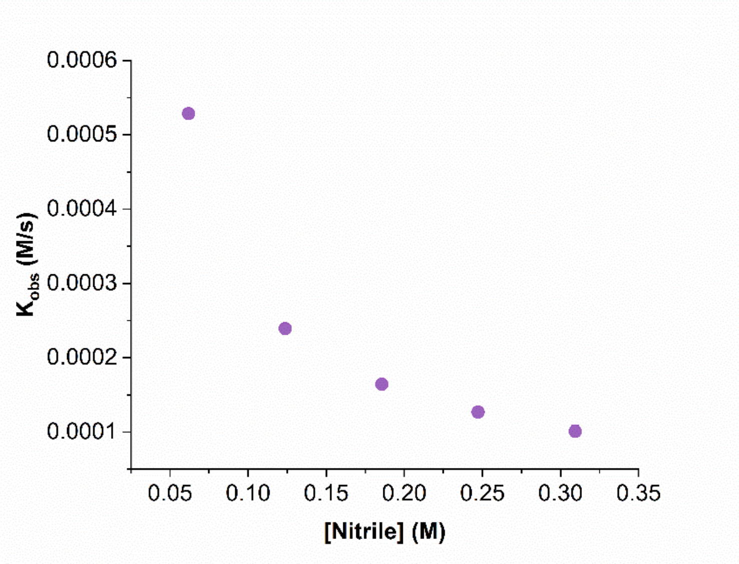

Figure S3: Graph to show concentration of cyclohexylphenyl ketone vs. observed rate equates to an inverse-first order dependence on the concentration of ketone over the investigated concentration range.

HBpin rate order assessment

Table S7: Rate constants measured for determination of HBpin order.

Cat A (M)	HBpin (M)	Benzonitrile (M)	k_{obs}	R^2
0.00333	0.354	0.333	$4.94x10^{-5} \pm 1.11x10^{-6}$	0.9974
0.00333	0.389	0.333	$5.87x10^{-5} \pm 3.57x10^{-6}$	0.9964
0.00333	0.439	0.333	$6.63x10^{-5} \pm 5.87x10^{-7}$	0.9976
0.00333	0.483	0.333	$6.99x10^{-5} \pm 1.38x10^{-6}$	0.9993
0.00333	0.531	0.333	$7.01x10^{-5} \pm 2.16x10^{-7}$	0.9987
0.00333	0.571	0.333	$7.02x10^{-5} \pm 5.86x10^{-6}$	0.9998
0.0033	0.612	0.333	$7.06x10^{-5} \pm 1.61x10^{-6}$	0.9966

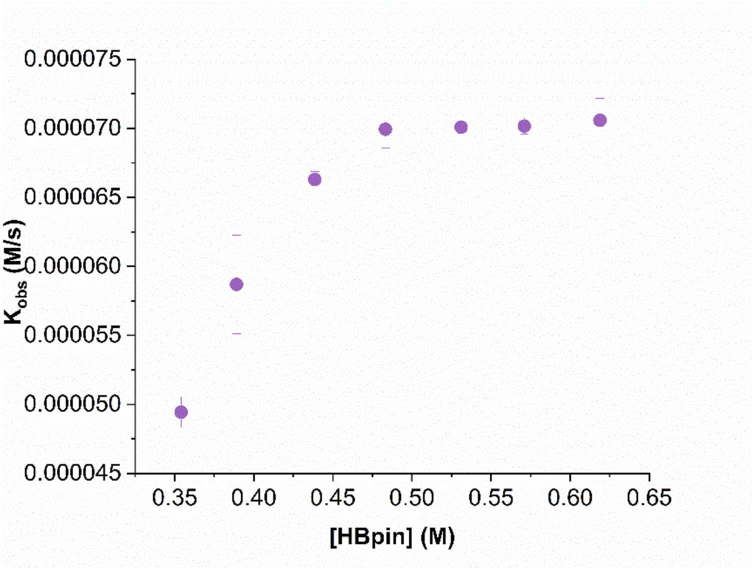

Figure S4: Graph to show concentration of HBpin *vs.* observed rate equates to saturation kinetics over investigated concentration range

Temperature dependance graphs

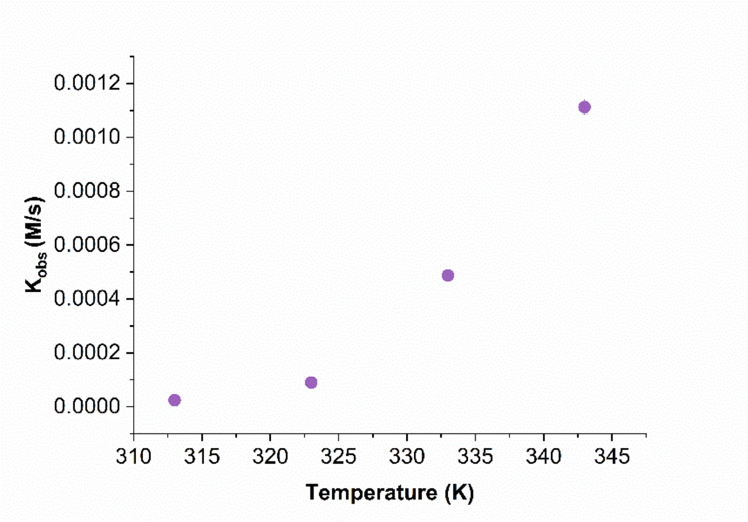

Figure S5: Graph to show dependence on temperature for hydroboration of benzonitrile by temperature *vs.* observed rate

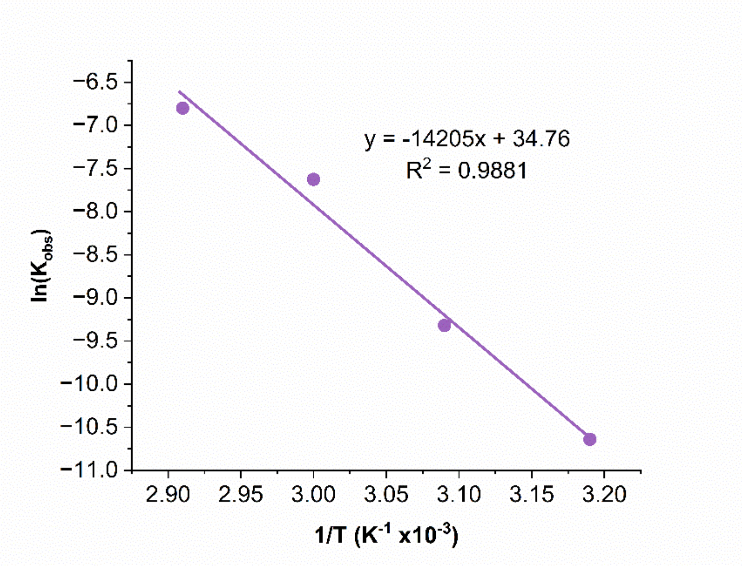

Figure S6: Effect of temperature on reaction rates used to determine both Eyring and Arrhenius parameters.

Kinetic isotope effect measurements

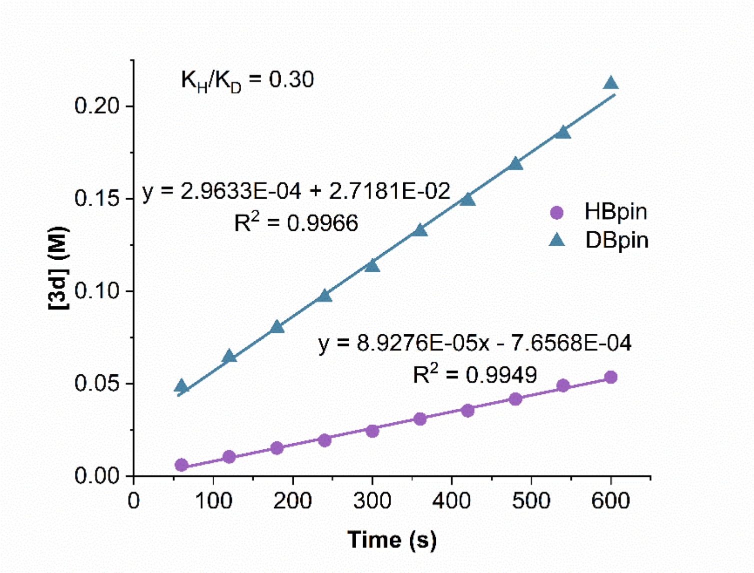

Figure S7: Kinetic isotope effect measurement time vs concentration of **3d**

Data for investigating the inverse first-order in nitrile.

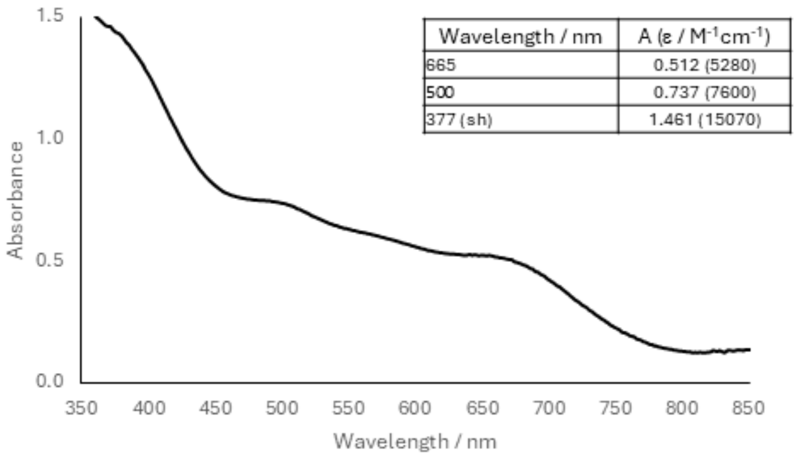

Wavelength / nm	A (ε / $M^{-1}cm^{-1}$)
665	0.512 (5280)
500	0.737 (7600)
377 (sh)	1.461 (15070)

Figure S8: UV-visible spectrum of **C** in toluene

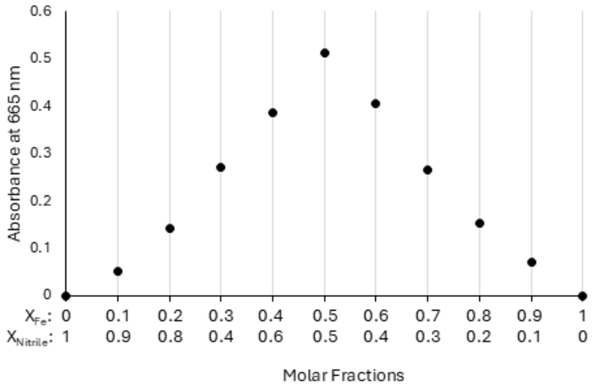

$X_{nitrile}$	X_{Fe}	Absorbance	Conc. 7 / mM
0	1	0	0.00
0.1	0.9	0.052	0.08
0.2	0.8	0.143	0.22
0.3	0.7	0.271	0.41
0.4	0.6	0.388	0.59
0.5	0.5	0.512	0.78
0.6	0.4	0.407	0.62
0.7	0.3	0.266	0.41
0.8	0.2	0.154	0.23
0.9	0.1	0.07	0.11
1	0	0	0.00

Figure S9: Job's Plot of molar fractions of Fe content (i.e. **A**/2) : benzonitrile determined by UV-visible spectroscopy

NMR spectra

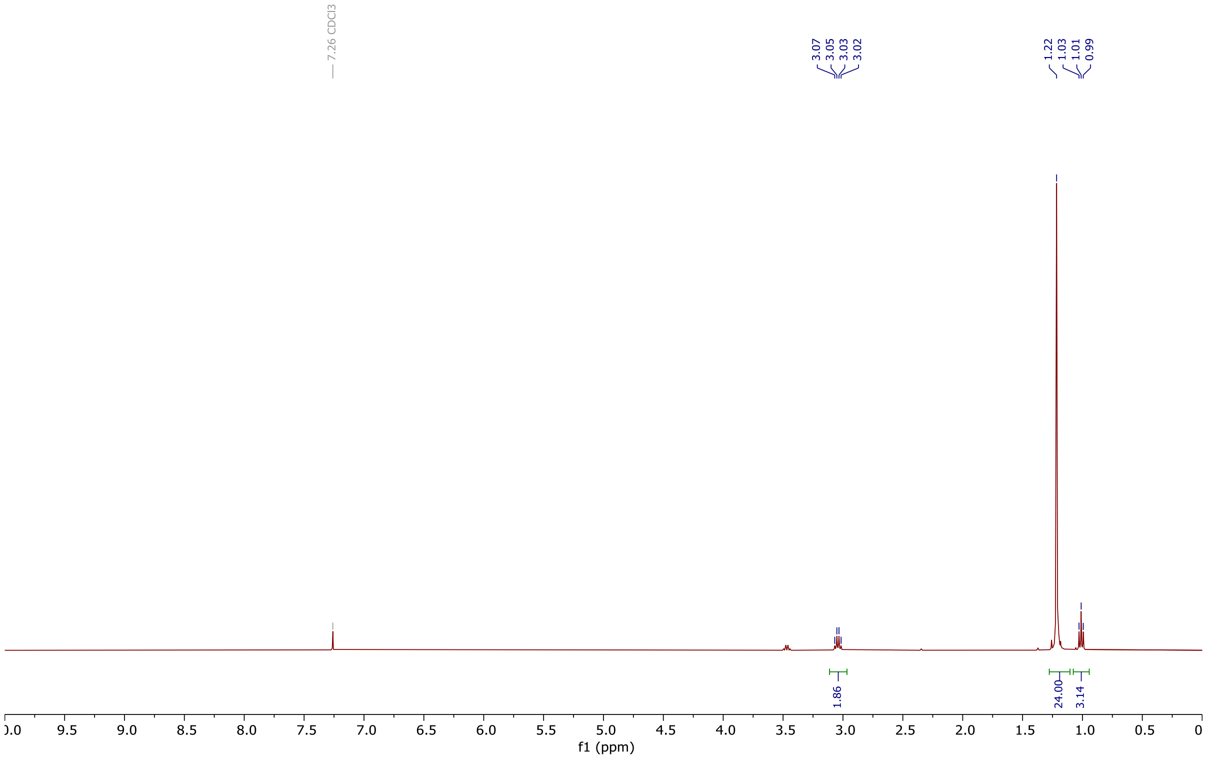

Figure S10: ^{1}H NMR spectrum ($CDCl_3$) of **3a**

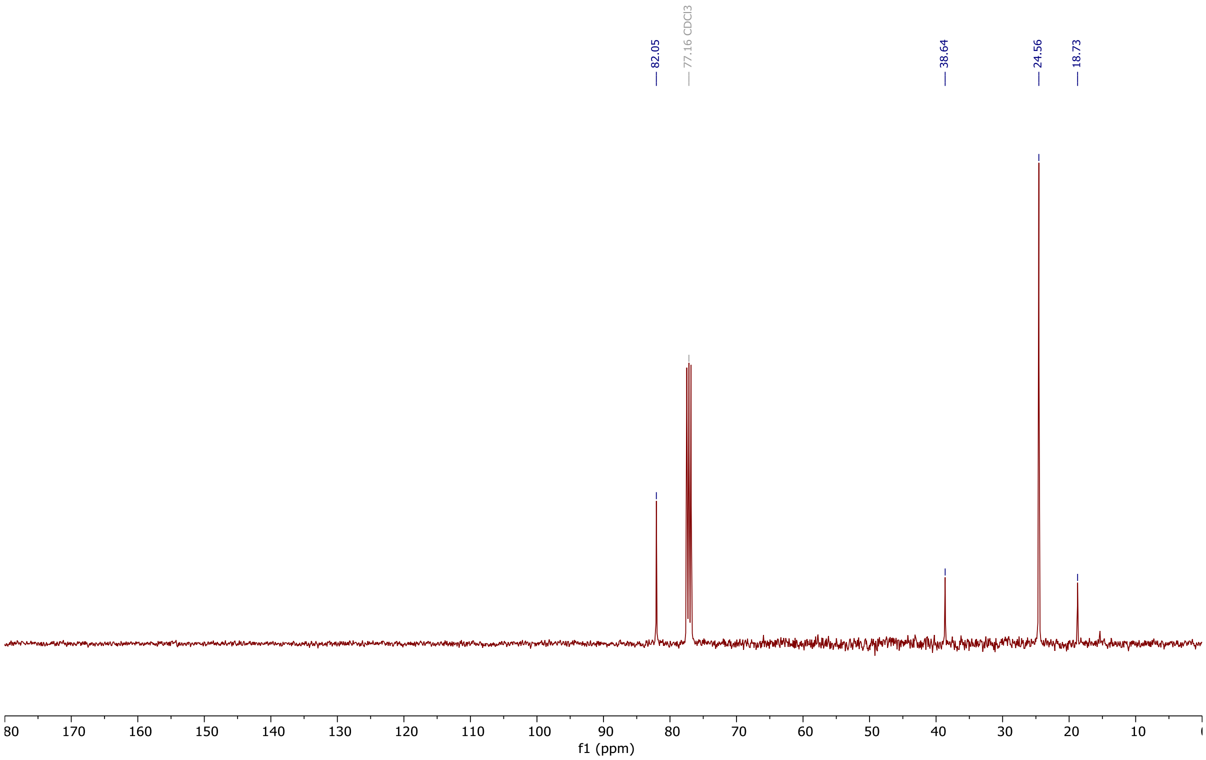

Figure S11: ^{13}C NMR spectrum ($CDCl_3$) of **3a**

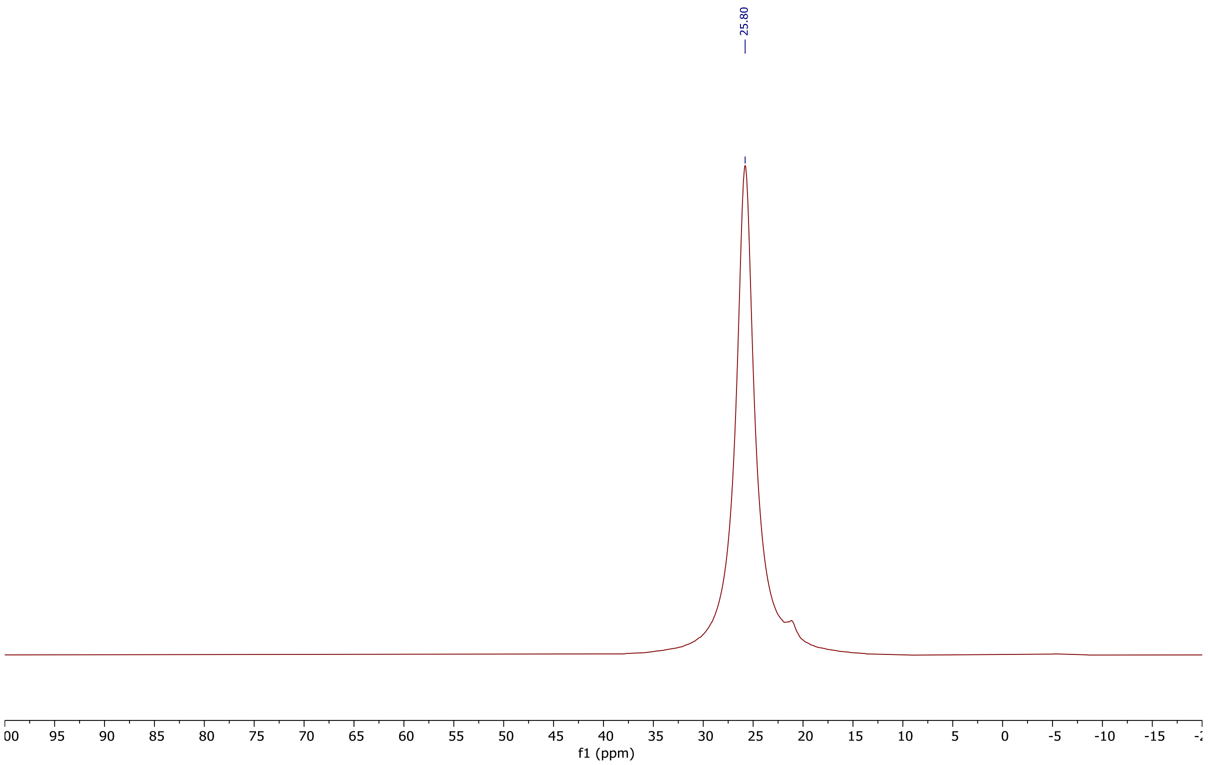

Figure S12: ^{11}B NMR spectrum ($CDCl_3$) of **3a**

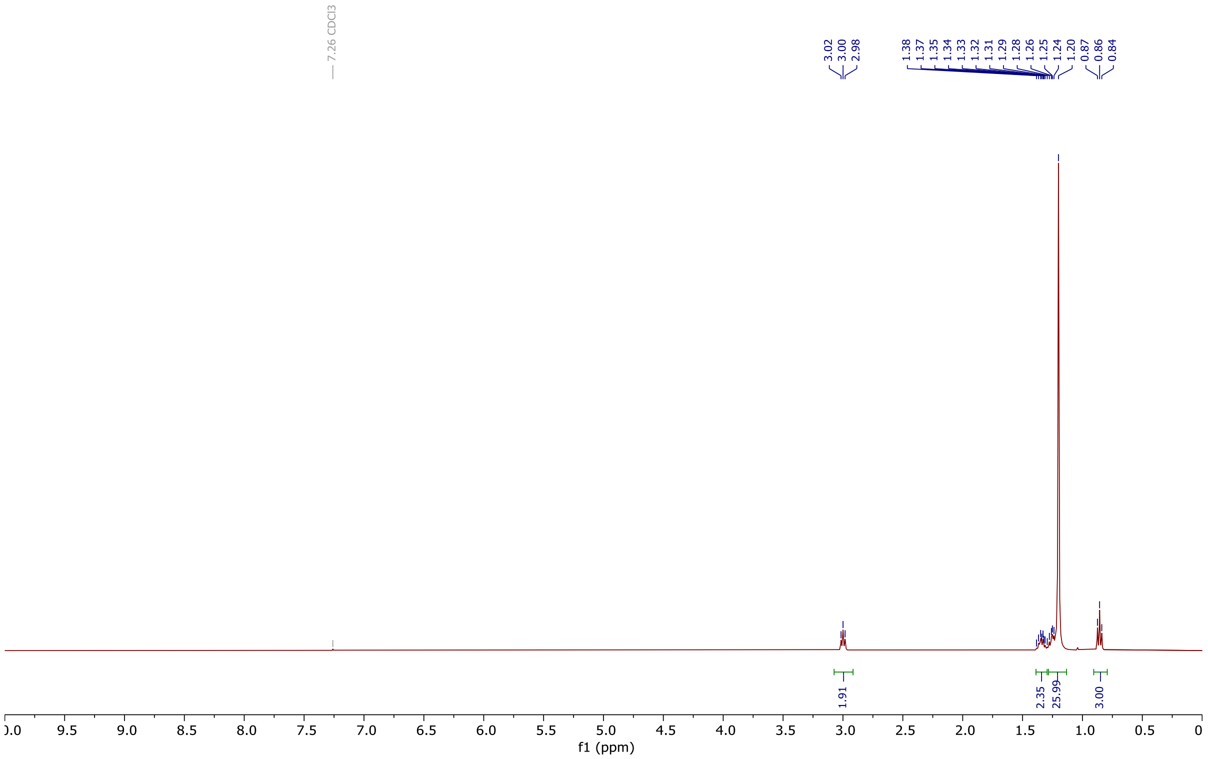

Figure S13: ^{1}H NMR spectrum ($CDCl_3$) of **3b**

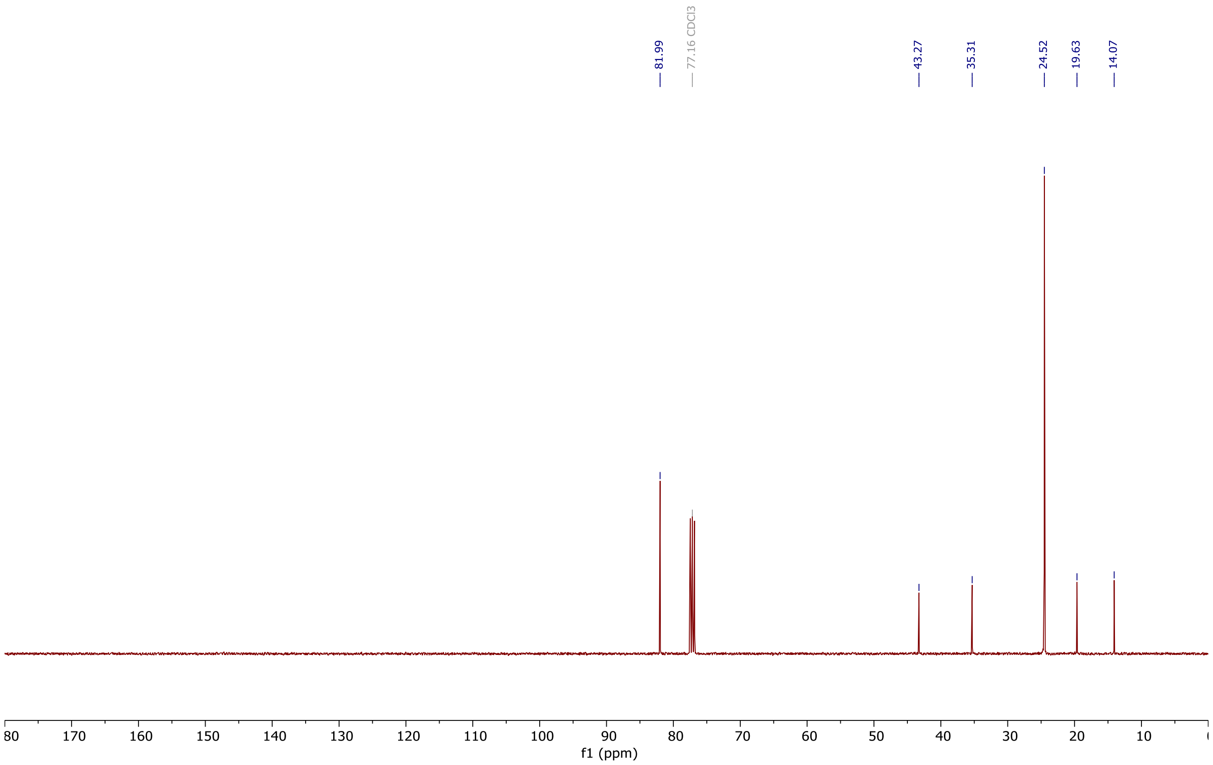

Figure S14: ^{13}C NMR spectrum ($CDCl_3$) of **3b**

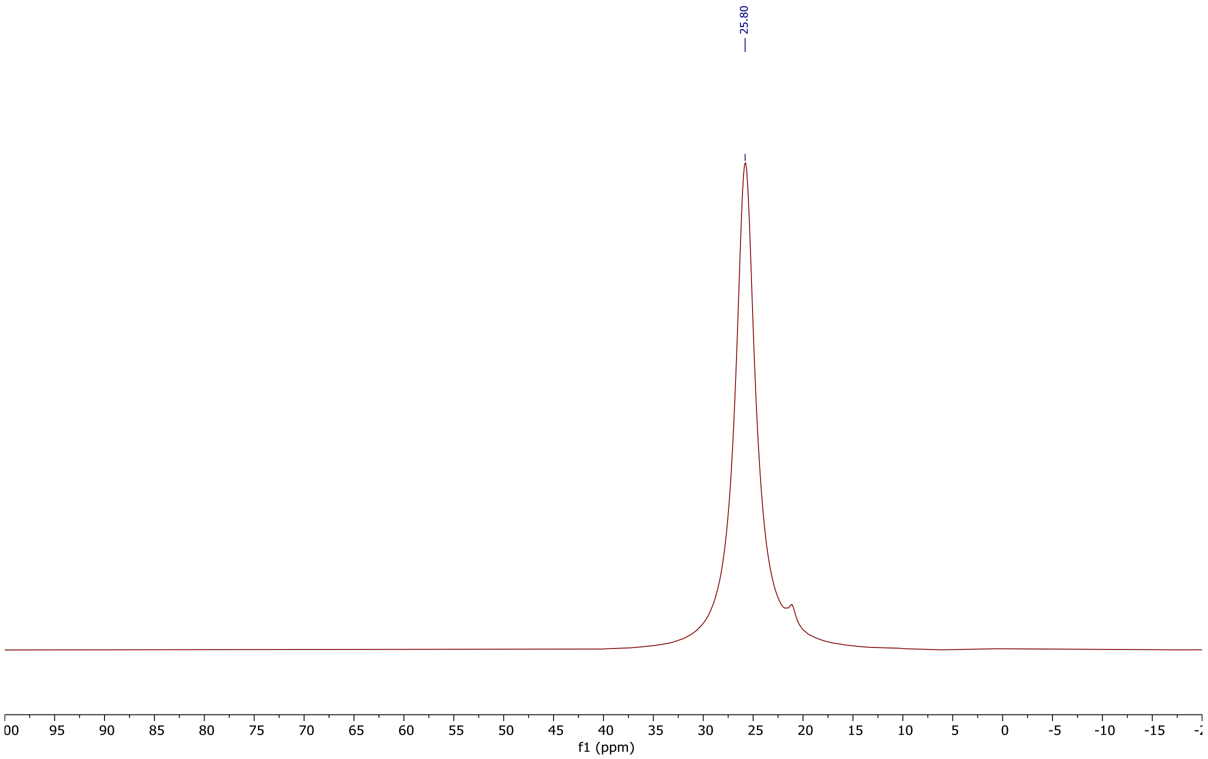

Figure S15: ^{11}B NMR spectrum ($CDCl_3$) of **3b**

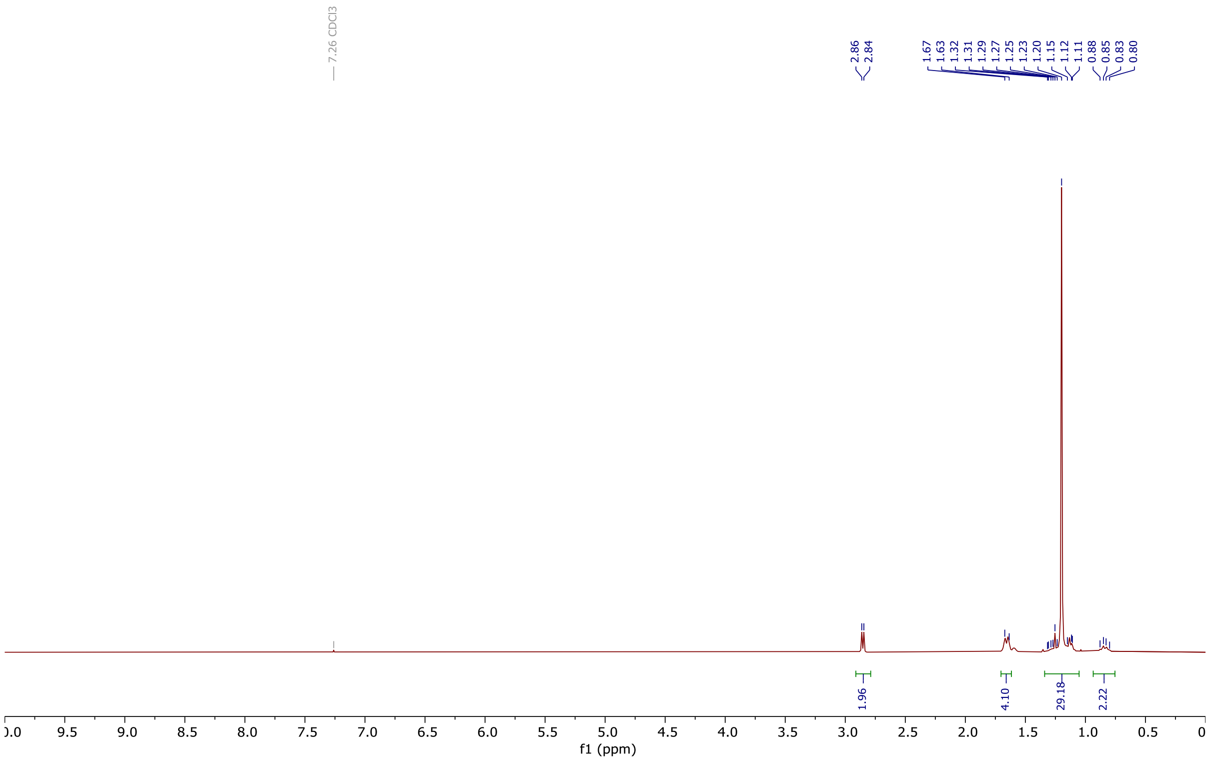

Figure S16: ^{1}H NMR spectrum ($CDCl_3$) of **3c**

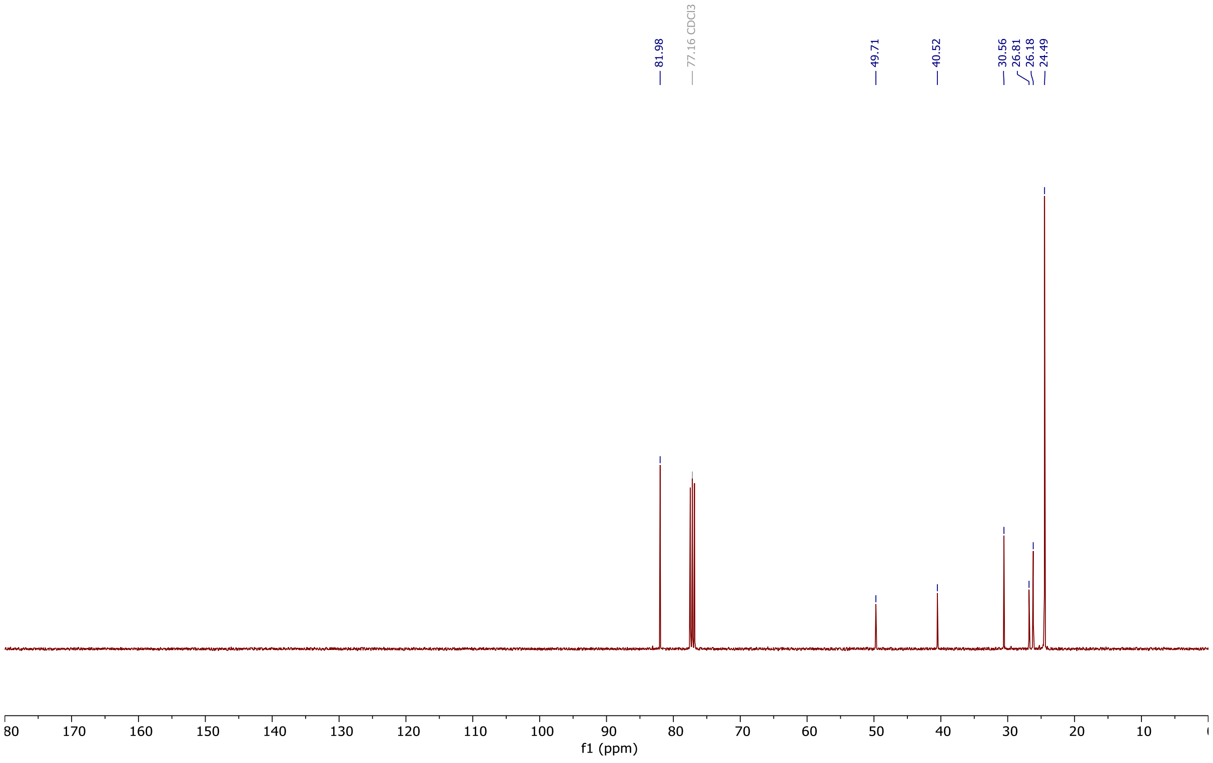

Figure S17: ^{13}C NMR spectrum ($CDCl_3$) of **3c**

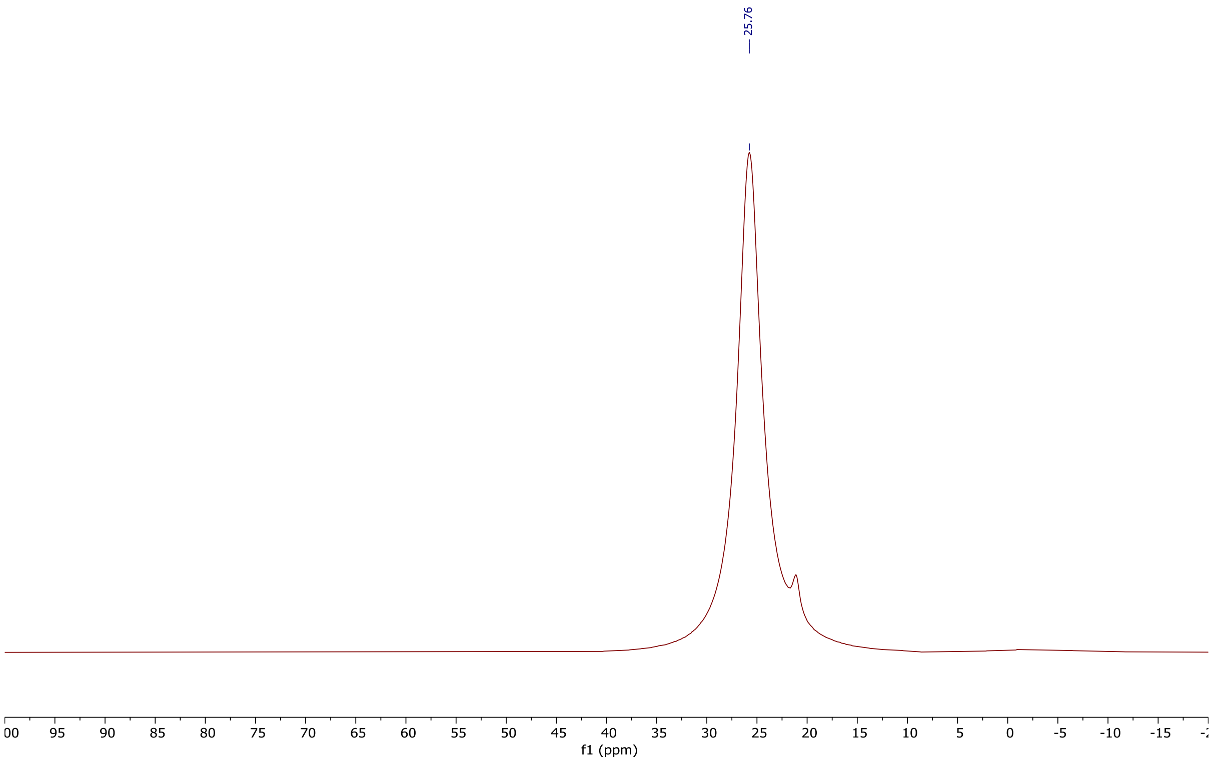

Figure S18: ^{11}B NMR spectrum ($CDCl_3$) of **3**

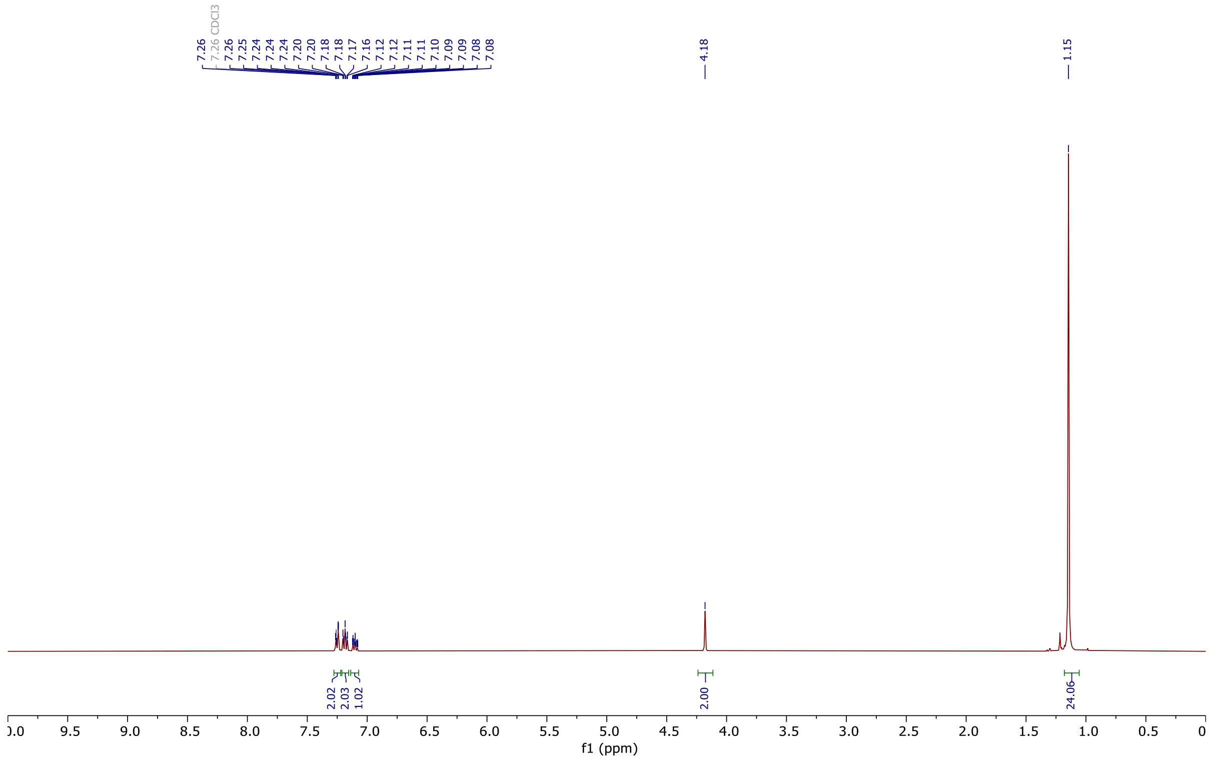

Figure S19: ^{1}H NMR spectrum ($CDCl_3$) of **3d**

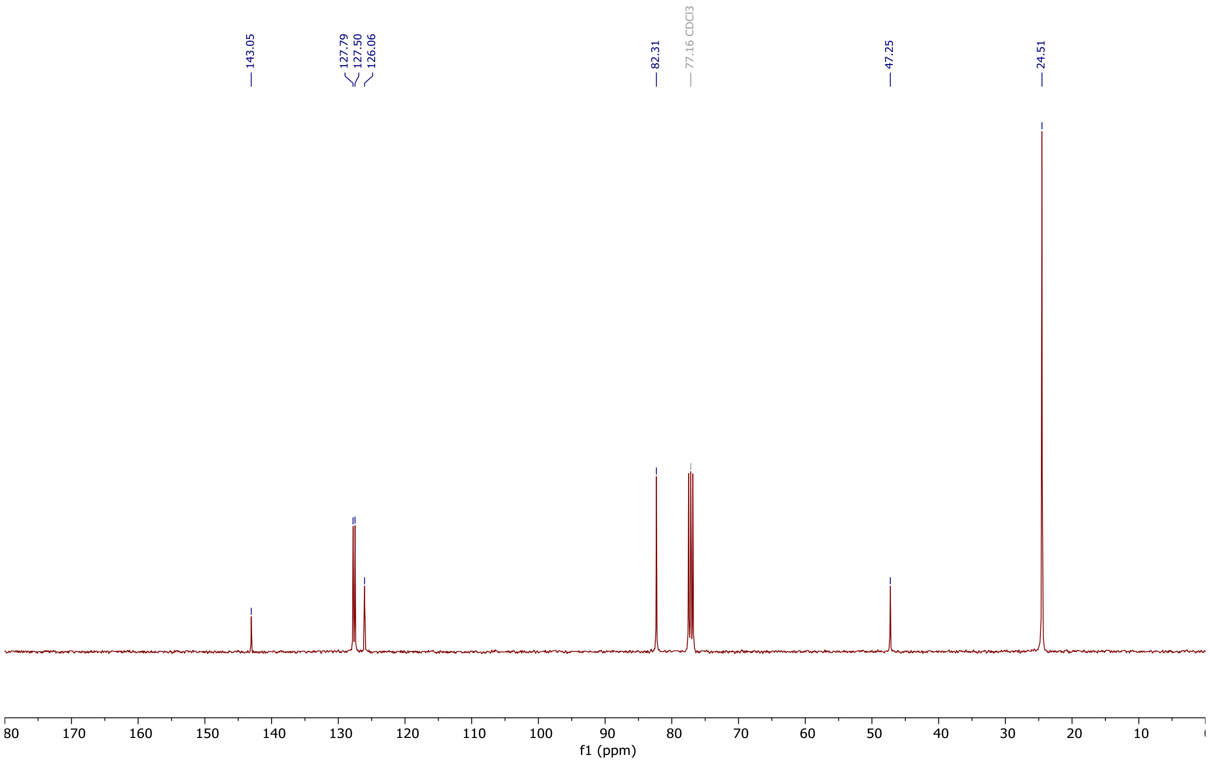

Figure S20: ^{13}C NMR spectrum ($CDCl_3$) of **3d**

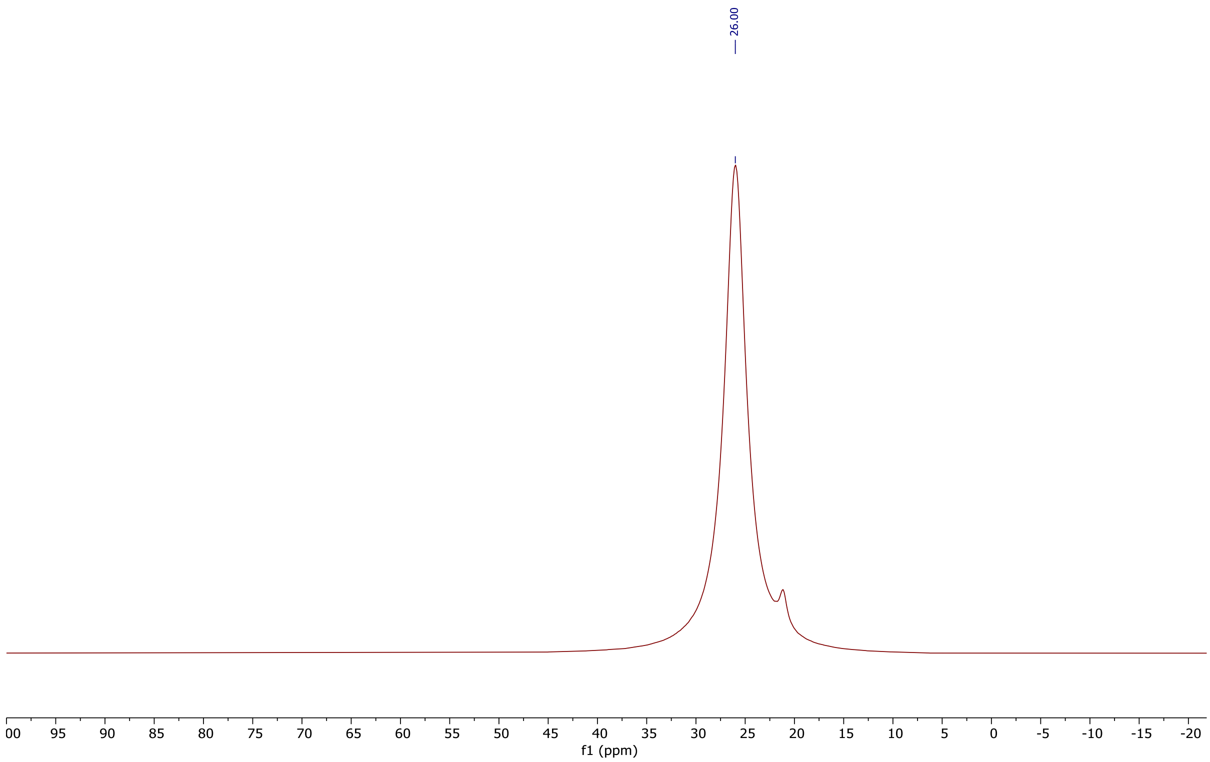

Figure S21: ^{11}B NMR spectrum ($CDCl_3$) of **3d**

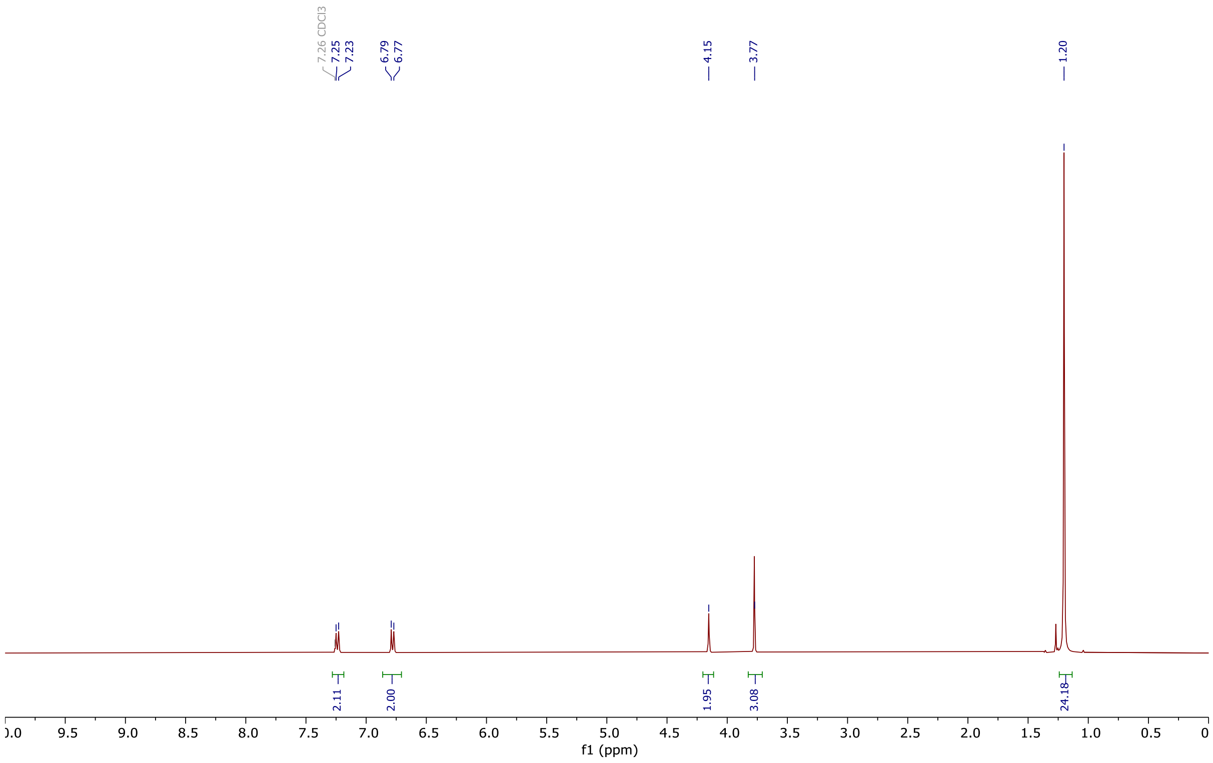

Figure S22: ^{1}H NMR spectrum ($CDCl_3$) of **3e**

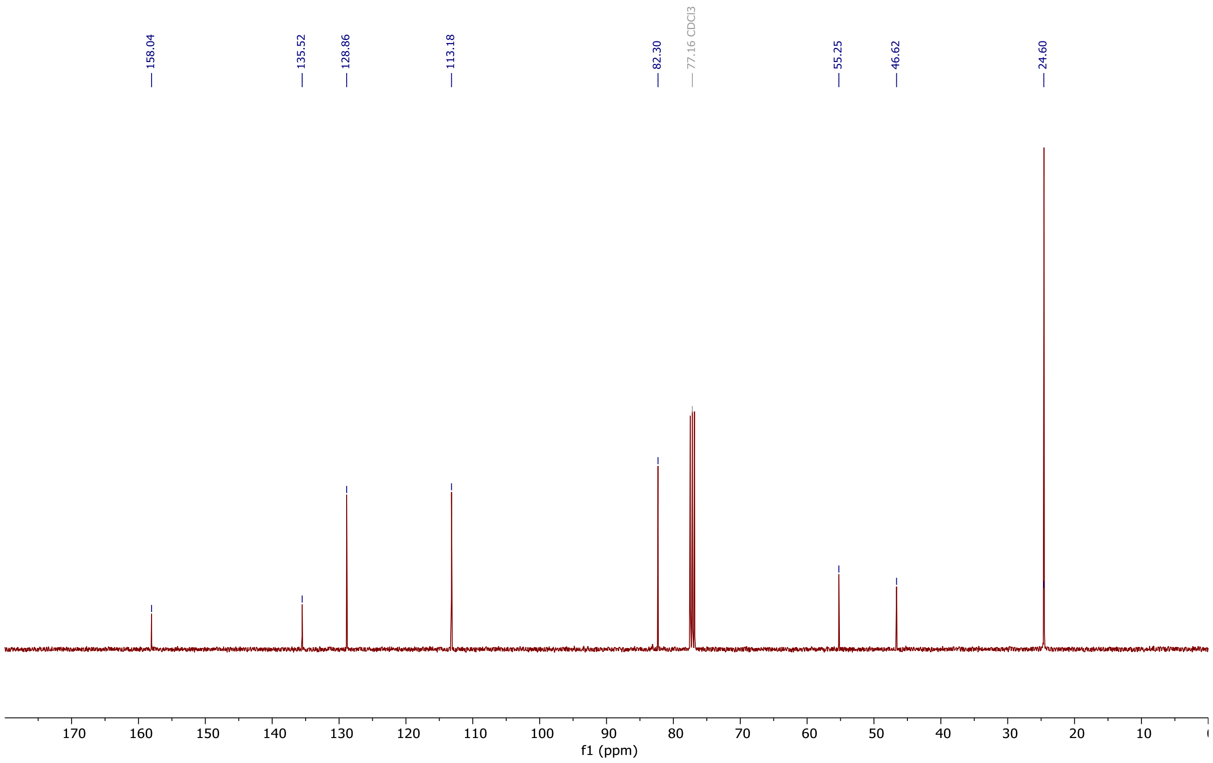

Figure S23: ^{13}C NMR spectrum ($CDCl_3$) of **3e**

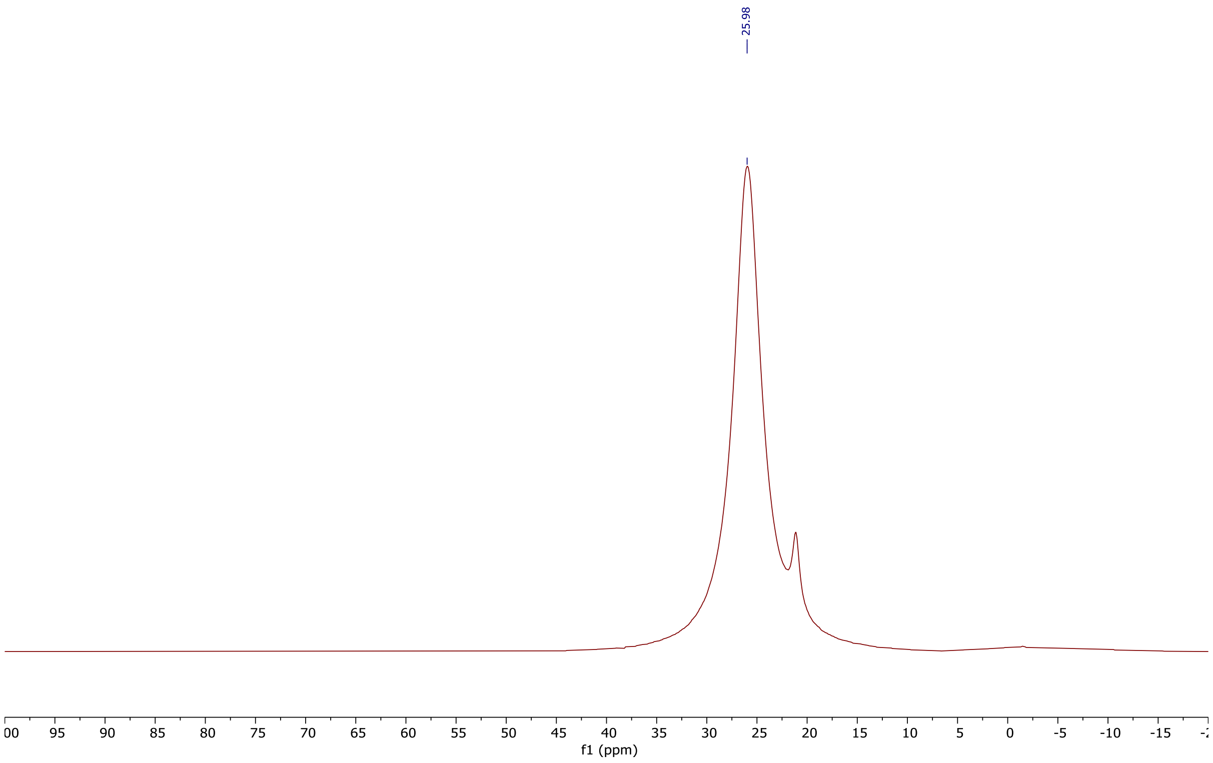

Figure S24: ^{11}B NMR spectrum ($CDCl_3$) of **3e**

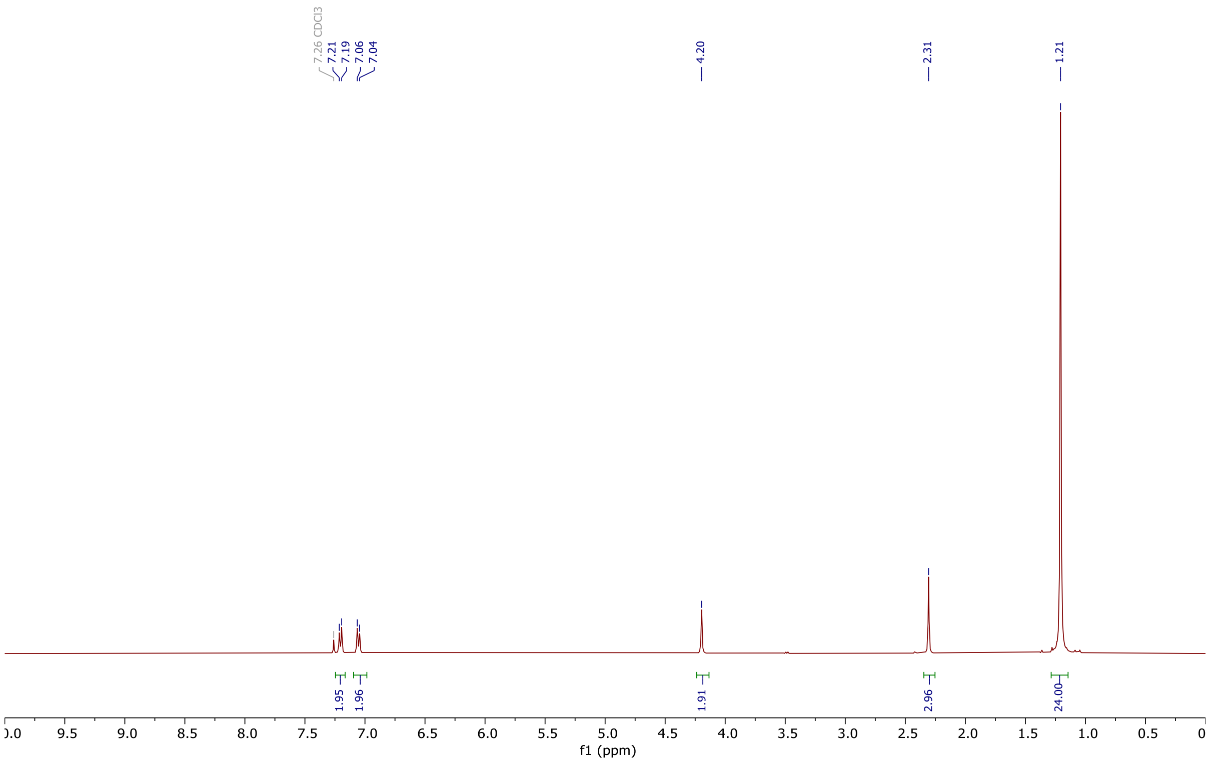

Figure S25: ^{1}H NMR spectrum ($CDCl_3$) of **3f**

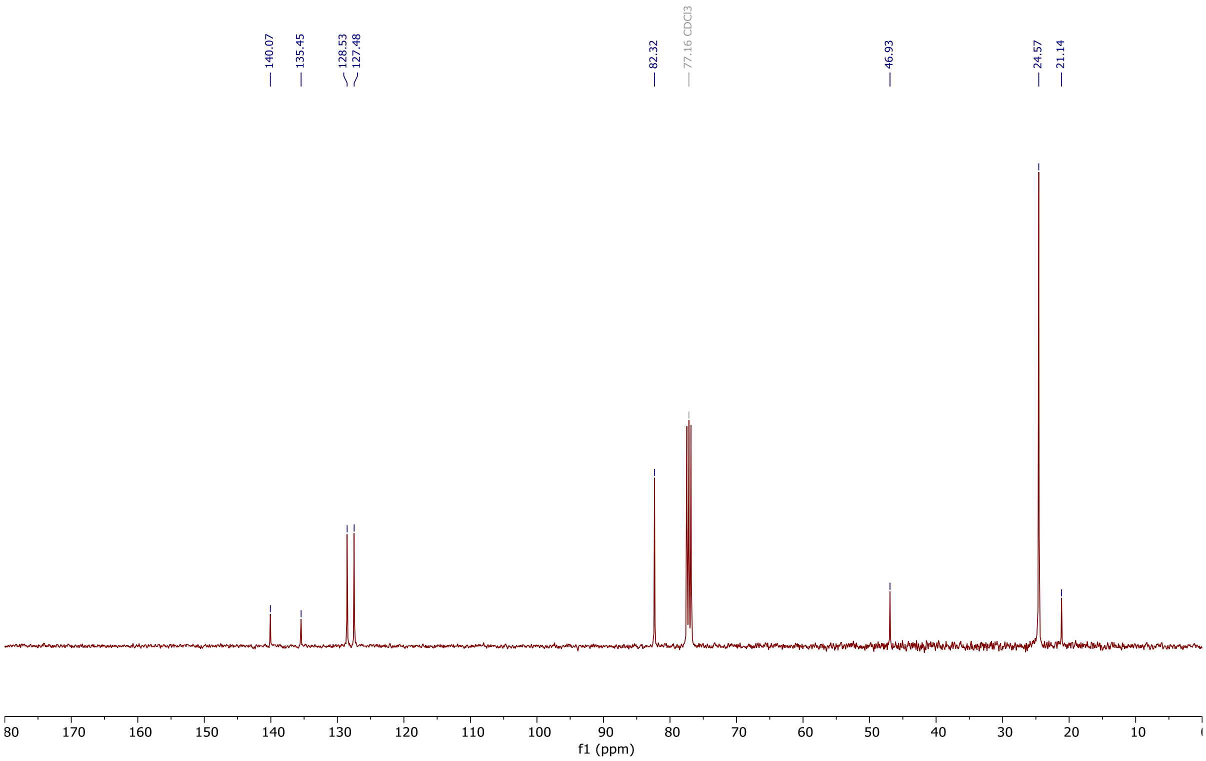

Figure S26: ^{13}C NMR spectrum ($CDCl_3$) of **3f**

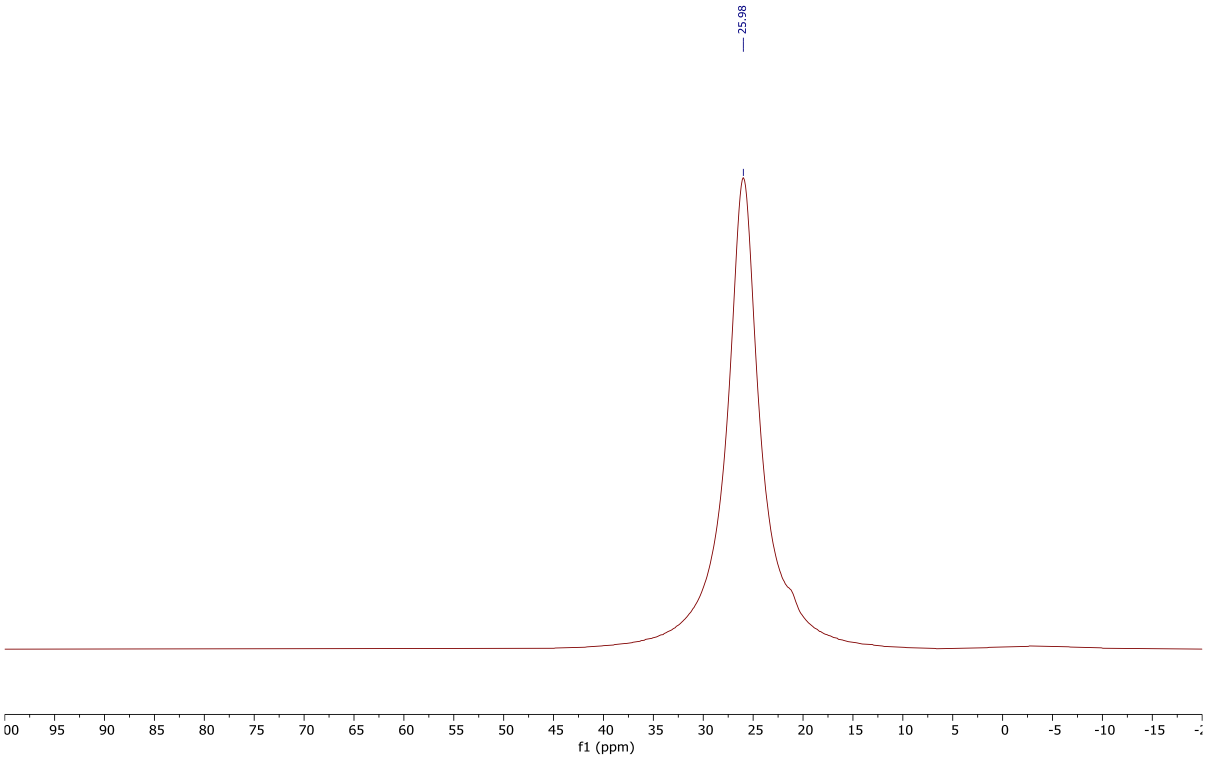

Figure S27: ^{11}B NMR spectrum ($CDCl_3$) of **3f**

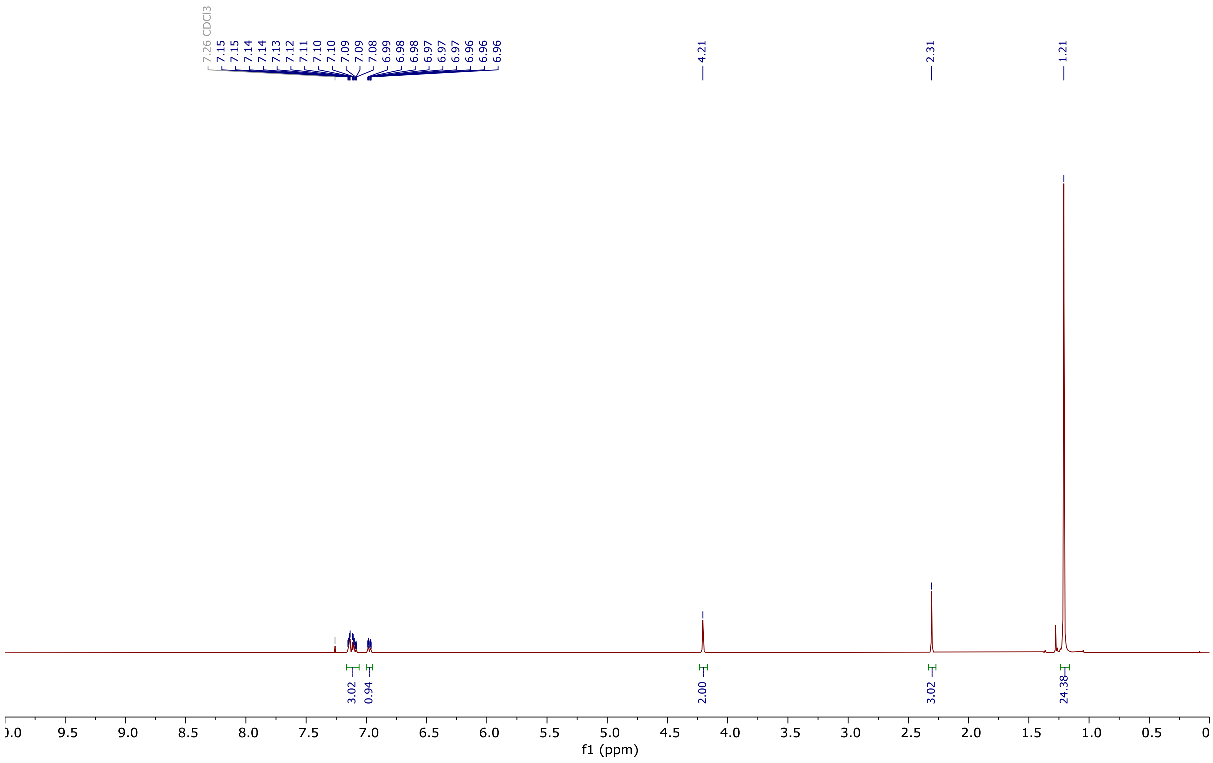

Figure S28: ^{1}H NMR spectrum ($CDCl_3$) of **3g**

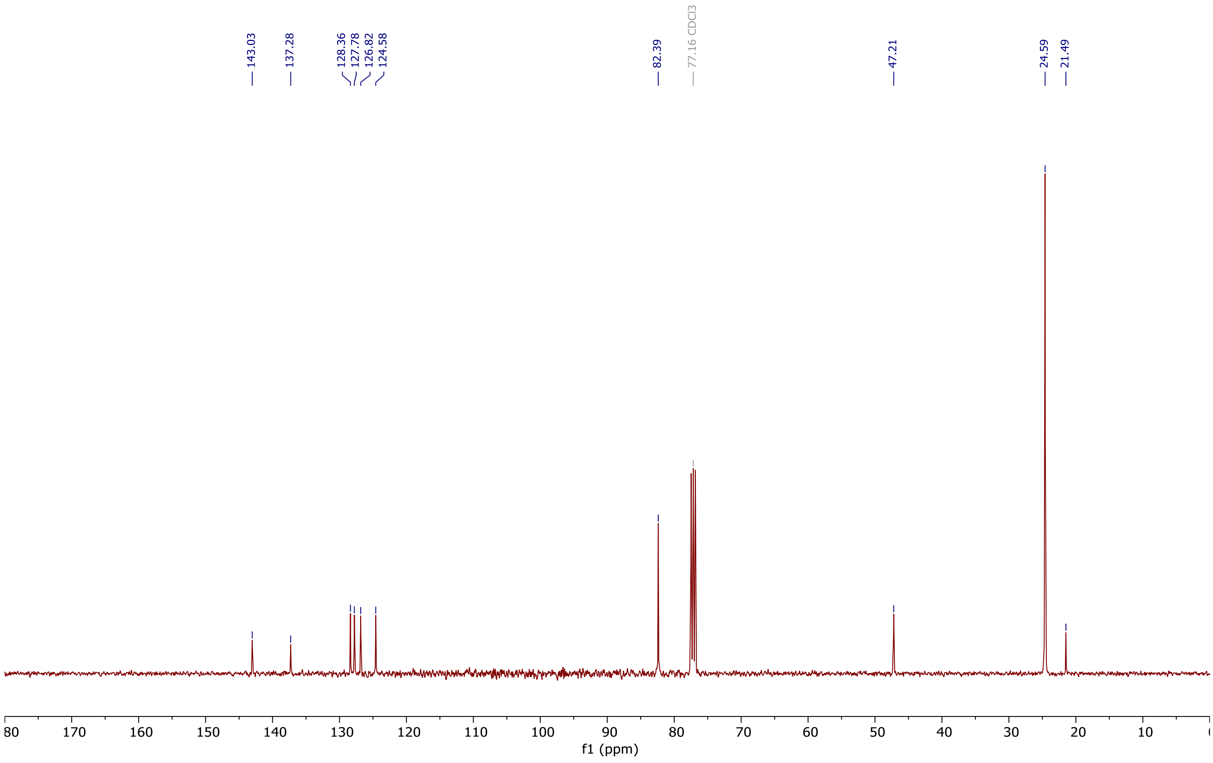

Figure S29: ^{13}C NMR spectrum ($CDCl_3$) of **3g**

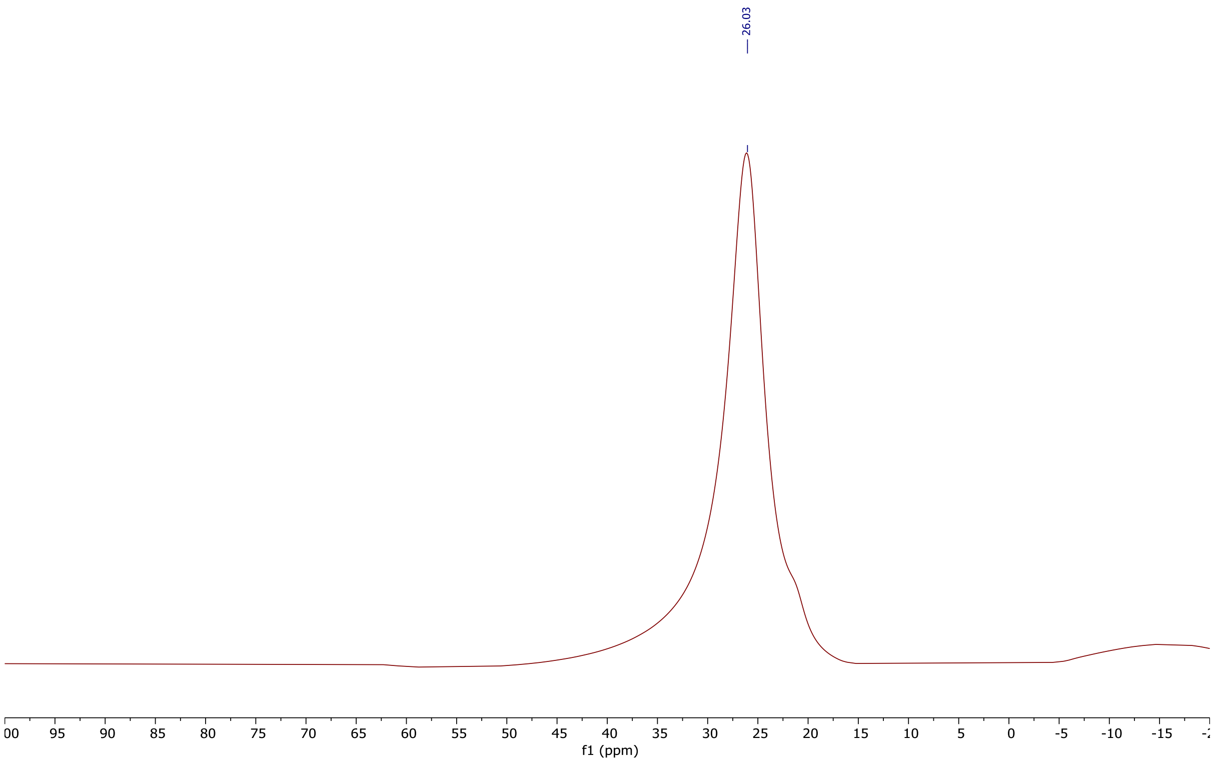

Figure S30: ^{11}B NMR spectrum ($CDCl_3$) of **3g**

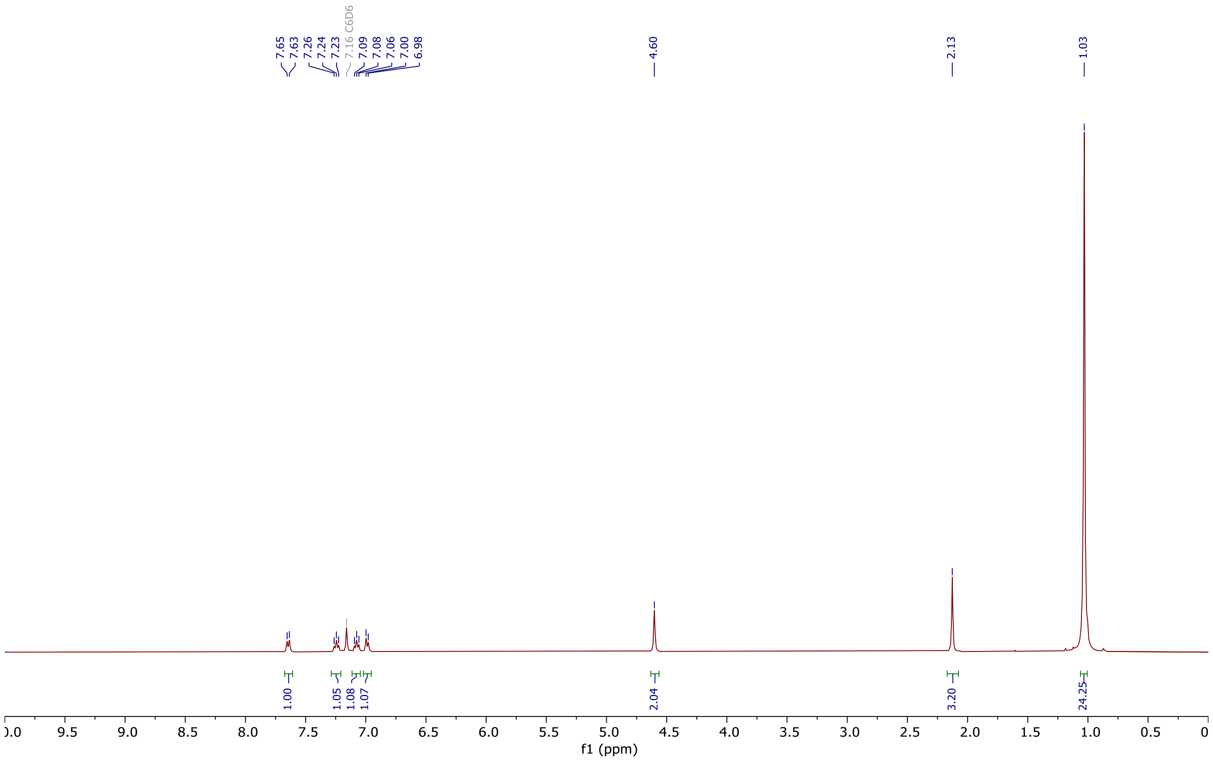

Figure S31: ^{1}H NMR spectrum (C_6D_6) of **3h**

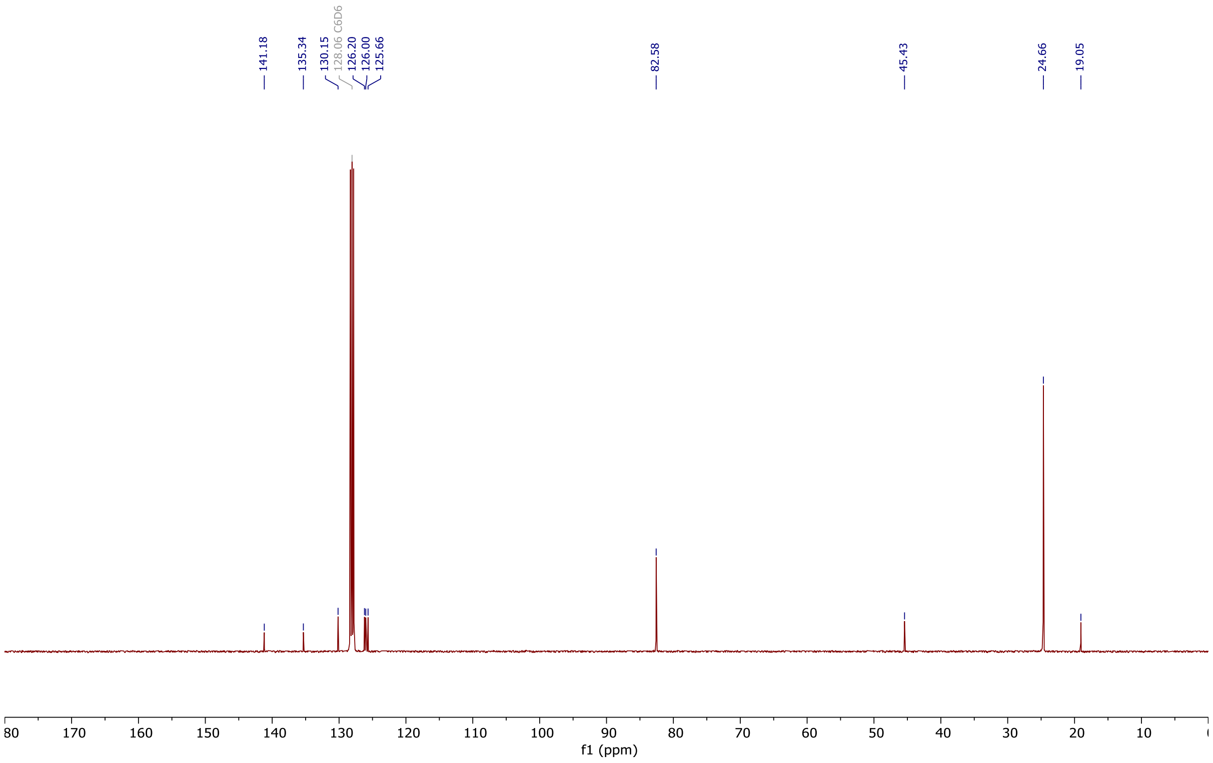

Figure S32: ^{13}C NMR spectrum (C_6D_6) of **3h**

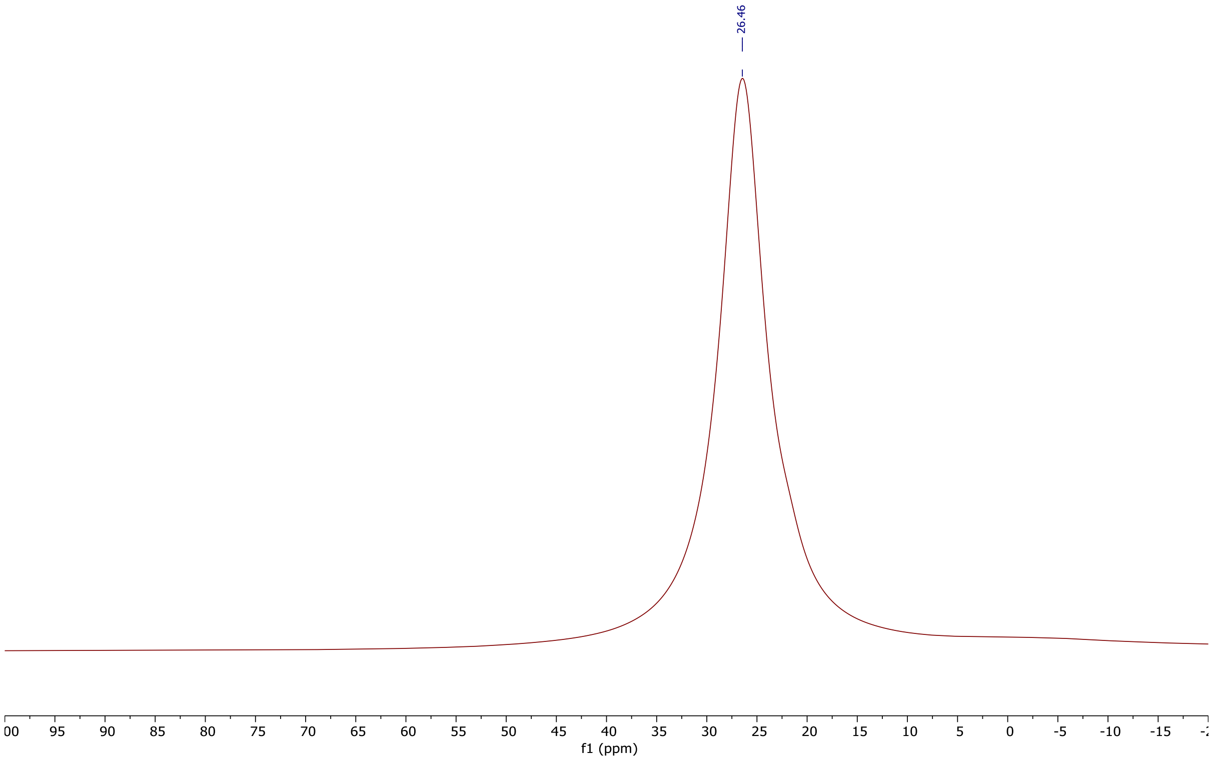

Figure S33: ^{11}B NMR spectrum (C_6D_6) of **3h**

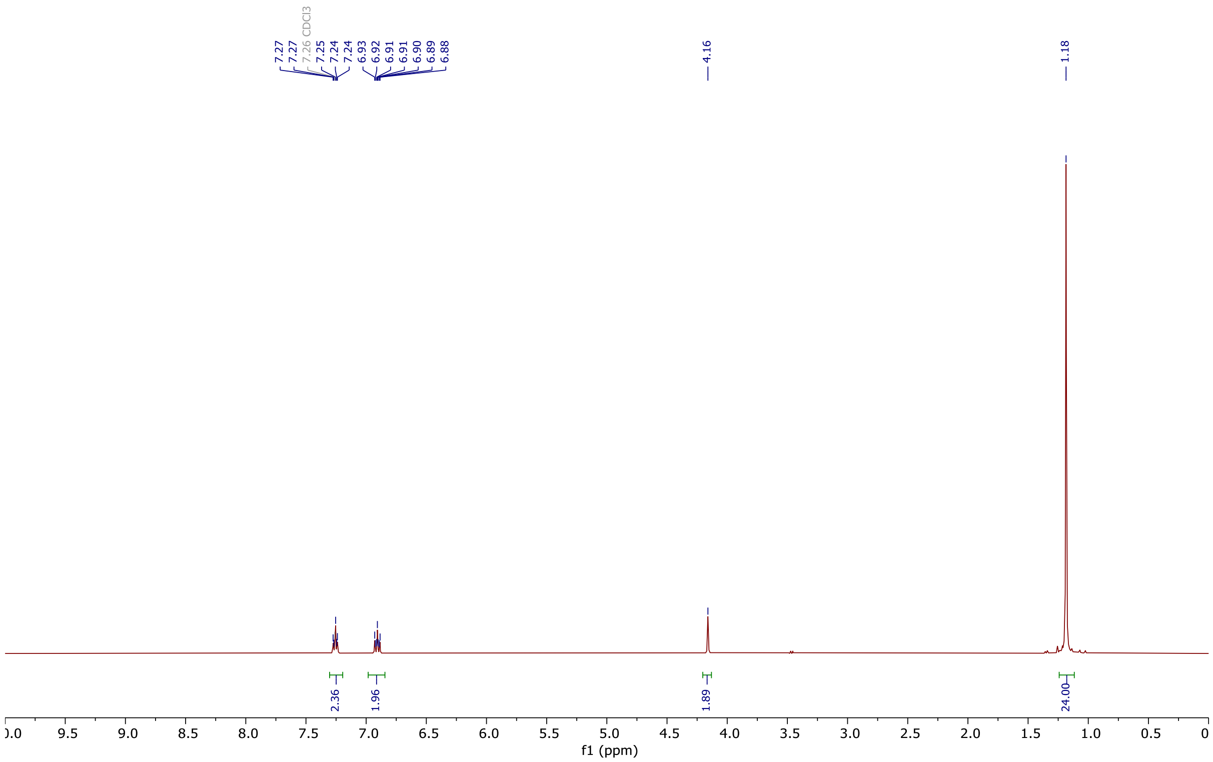

Figure S34: ^{1}H NMR spectrum ($CDCl_3$) of **3i**

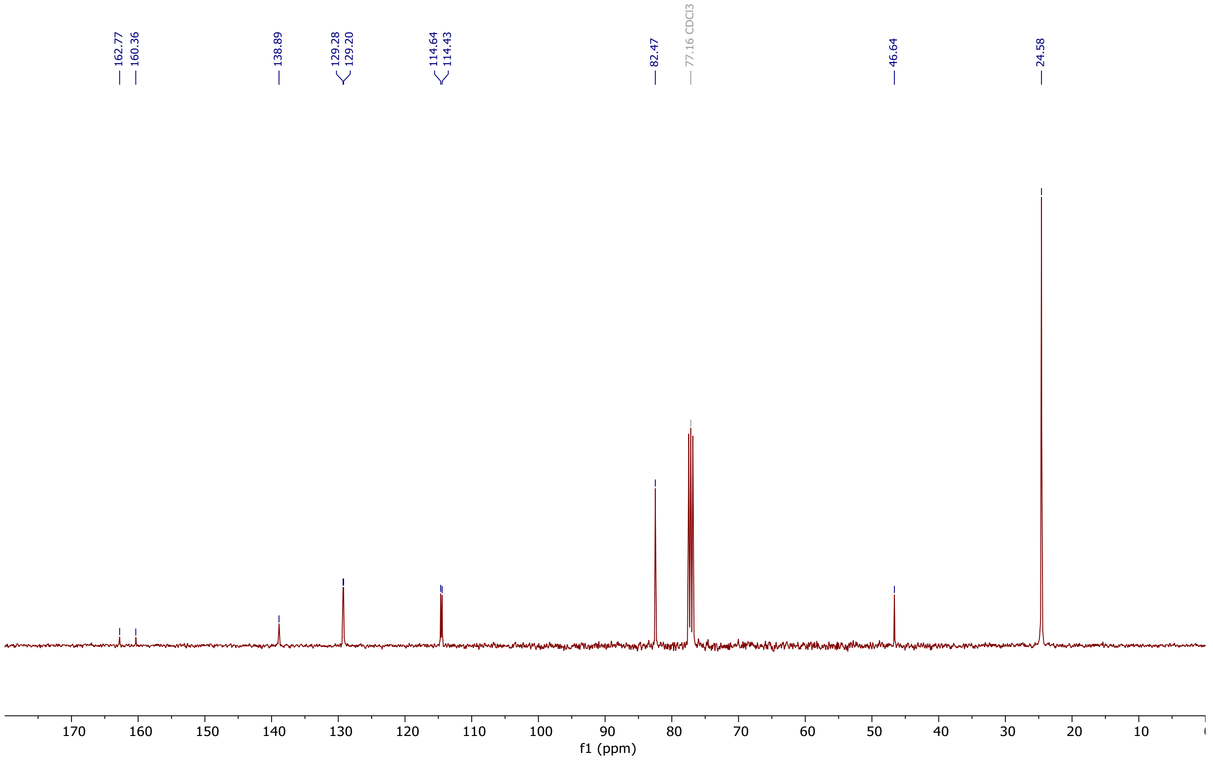

Figure S35: ^{13}C NMR spectrum ($CDCl_3$) of **3i**

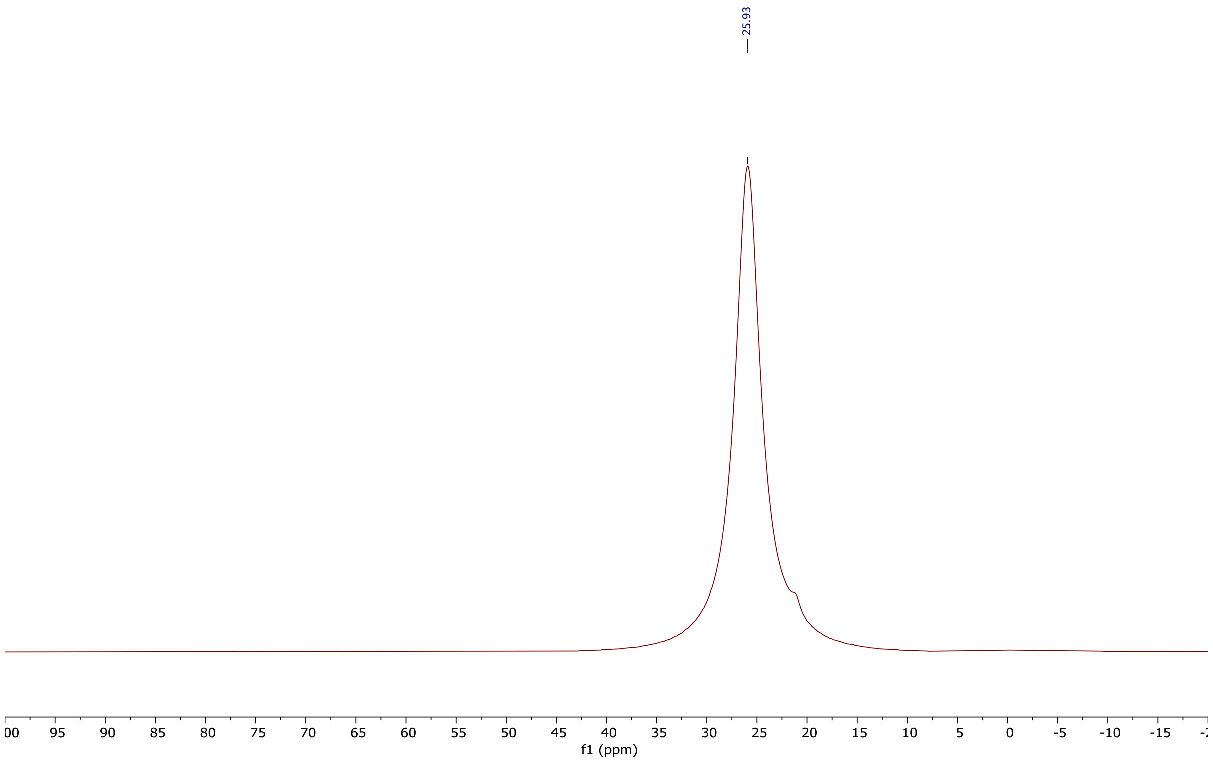

Figure S36: ^{11}B NMR spectrum ($CDCl_3$) of **3i**

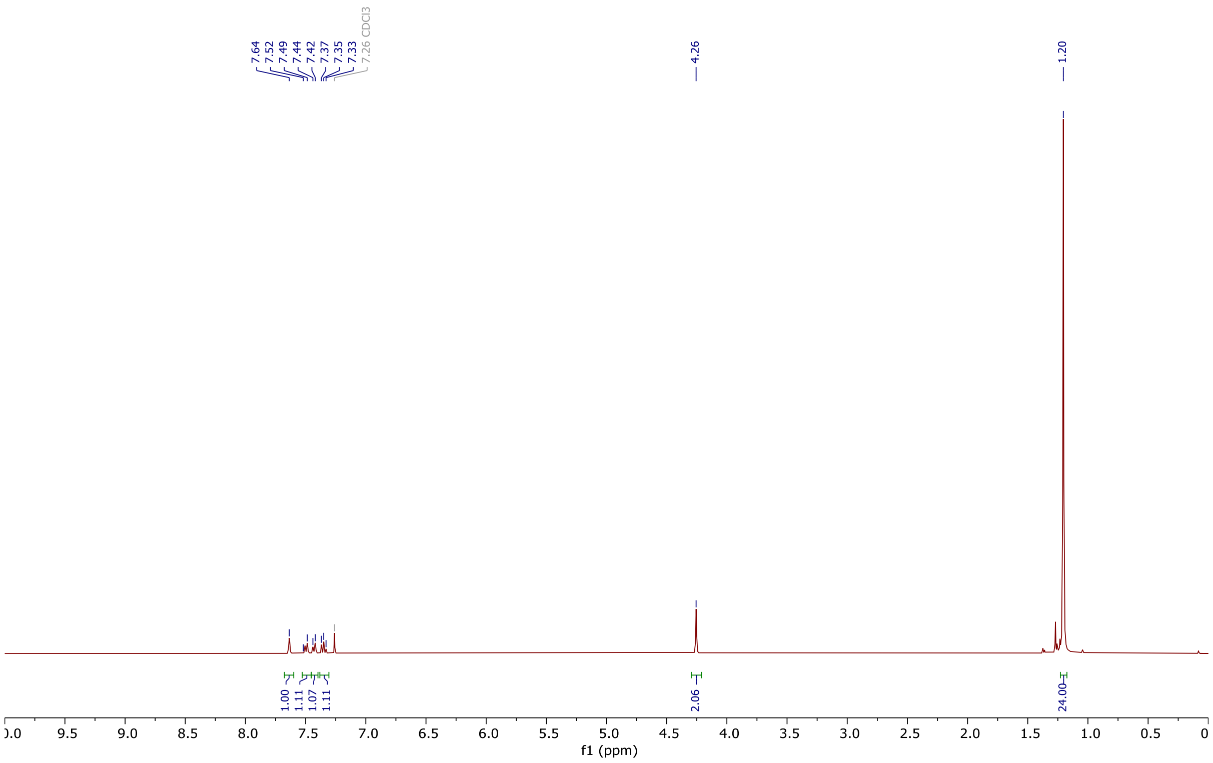

Figure S37: ^{1}H NMR spectrum ($CDCl_3$) of **3j**

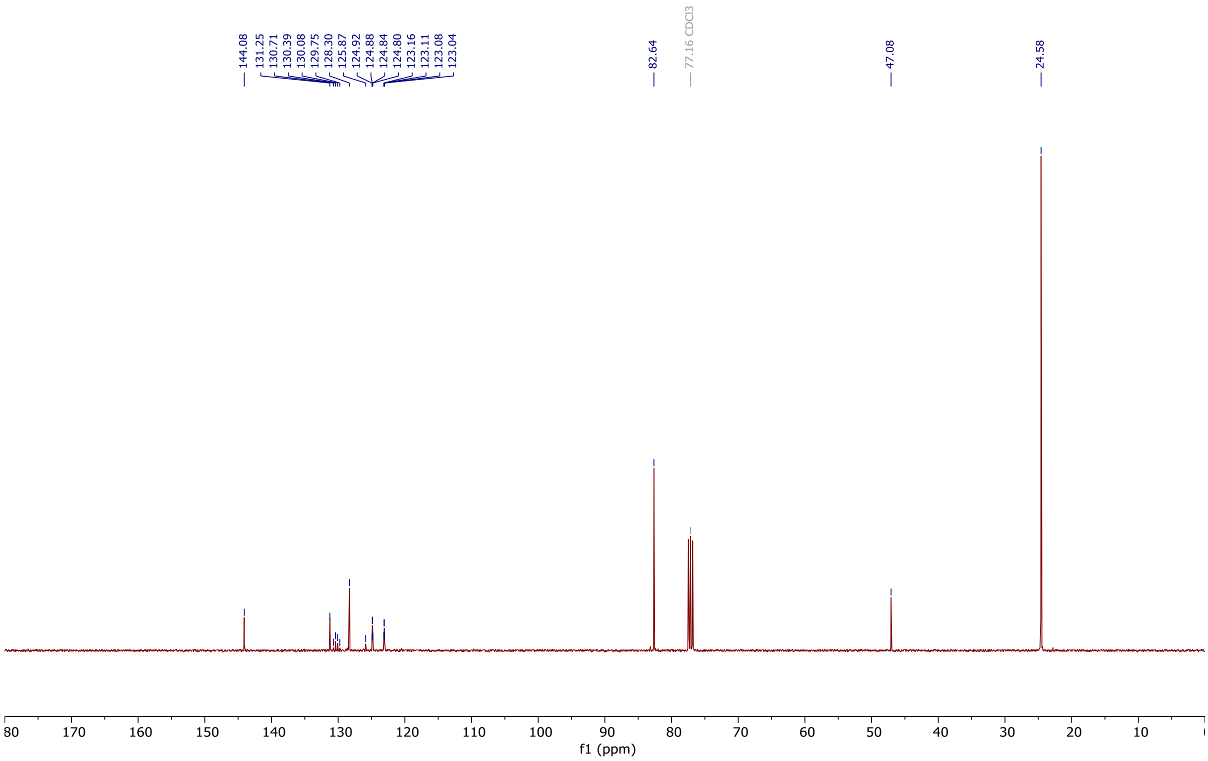

Figure S38: ^{13}C NMR spectrum ($CDCl_3$) of **3j**

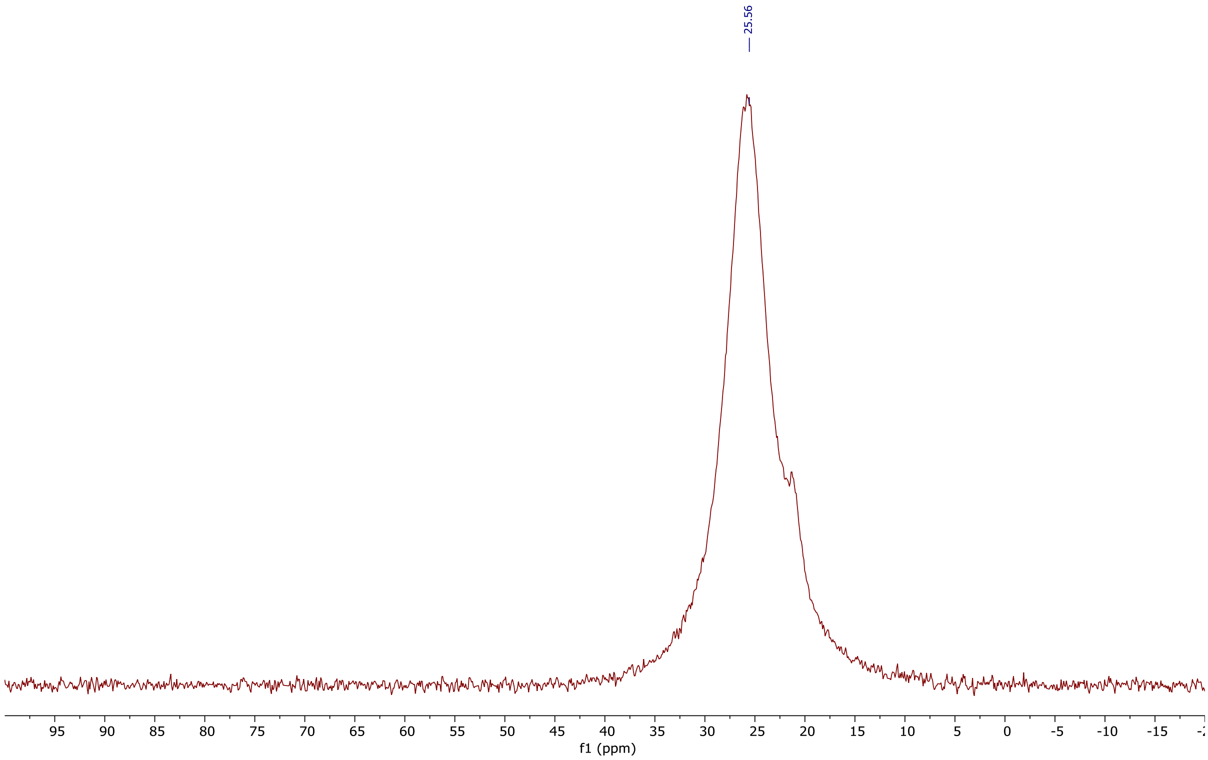

Figure S39: ^{11}B NMR spectrum ($CDCl_3$) of **3j**

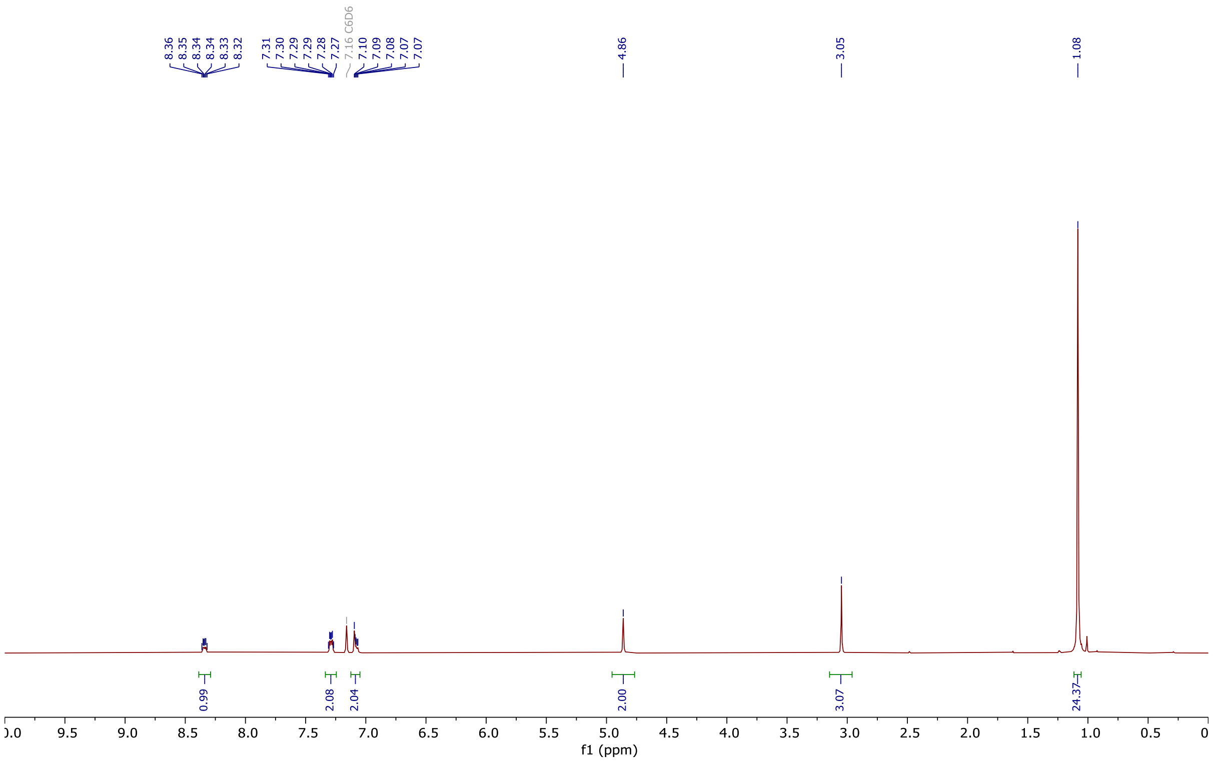

Figure S40: ^{1}H NMR spectrum (C_6D_6) of **3k**

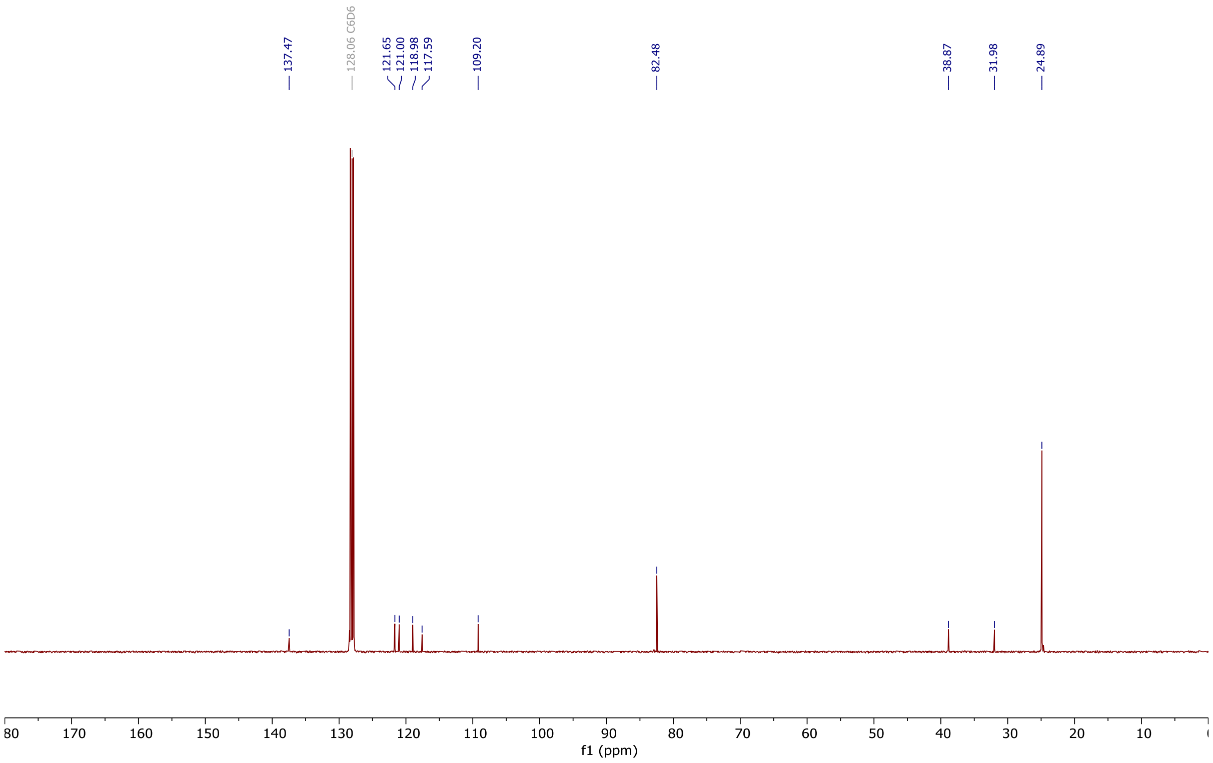

Figure S41: ^{13}C NMR spectrum (C_6D_6) of **3k**

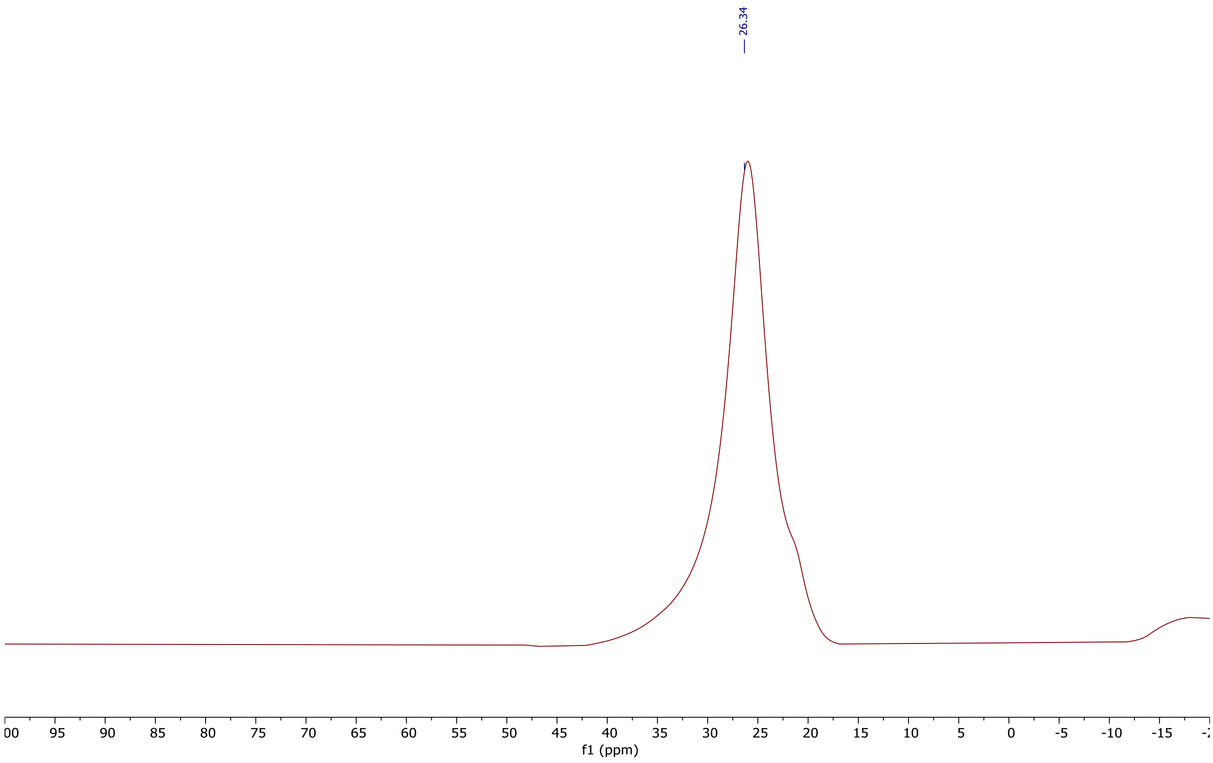

Figure S42: ^{11}B NMR spectrum (C_6D_6) of **3k**

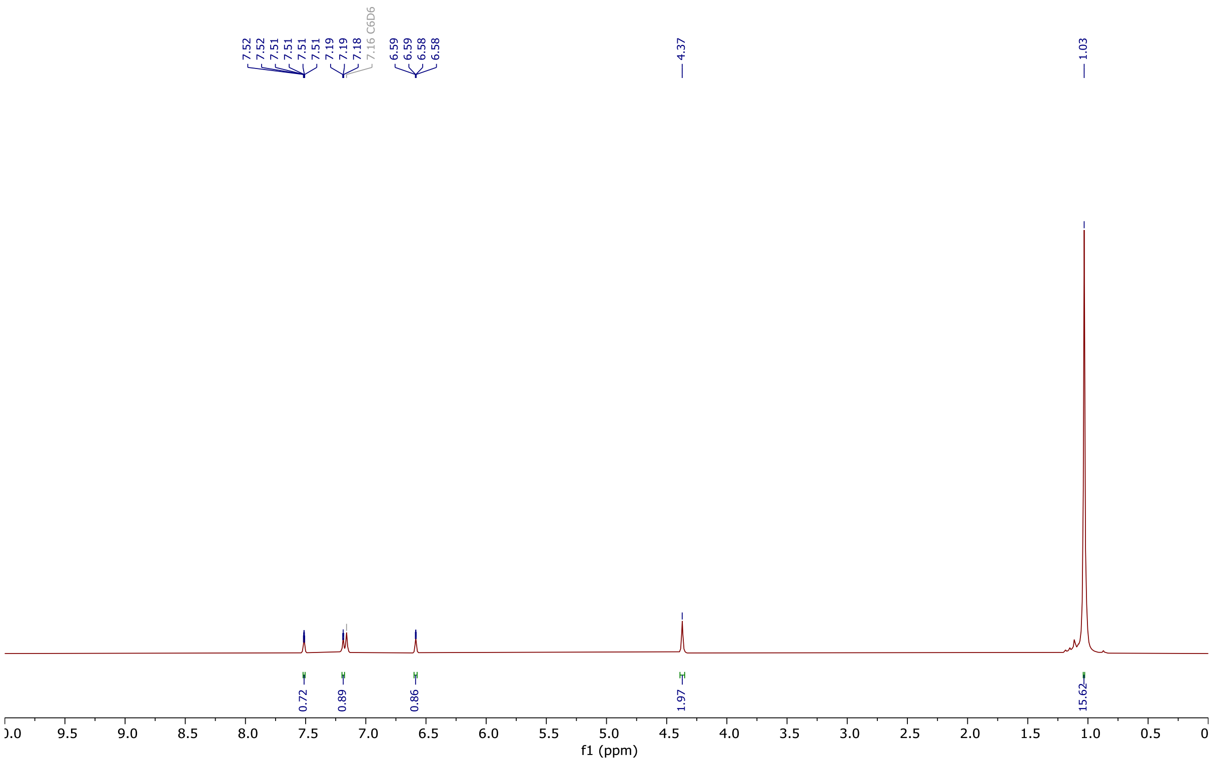

Figure S43: ^{1}H NMR spectrum (C_6D_6) of **3l**

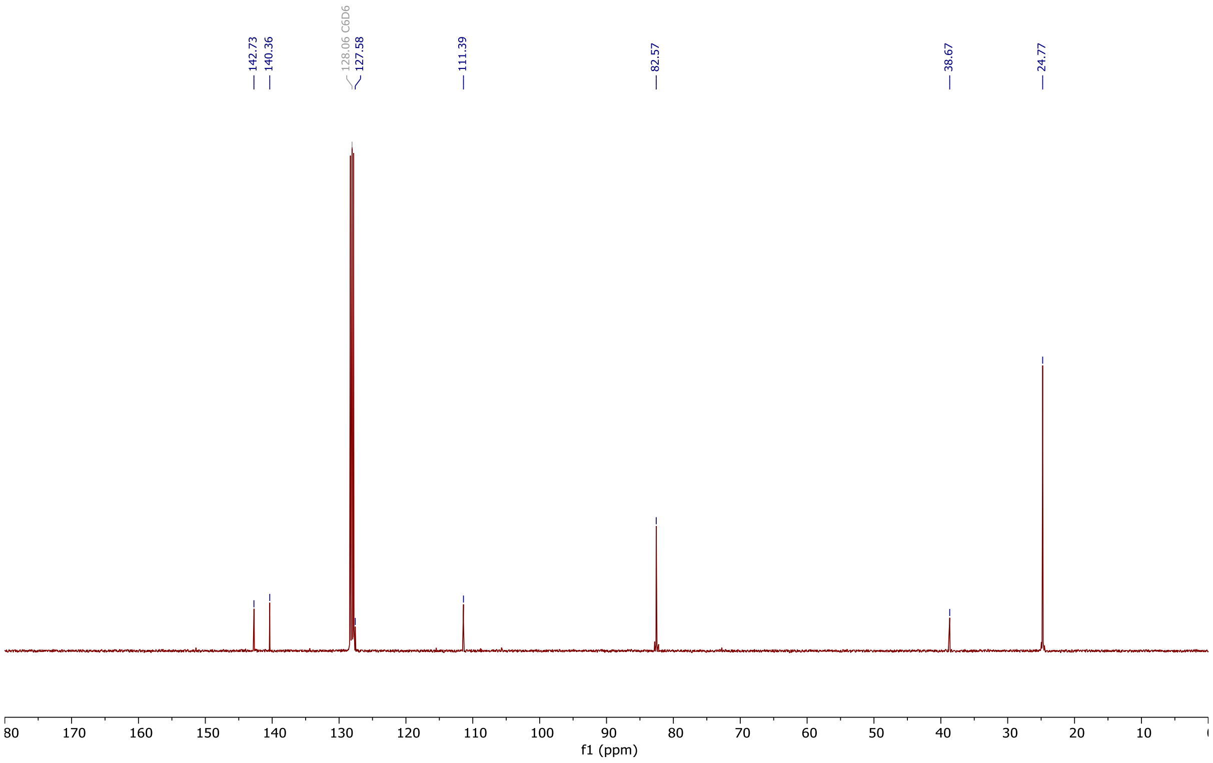

Figure S44: ^{13}C NMR spectrum (C_6D_6) of **3l**

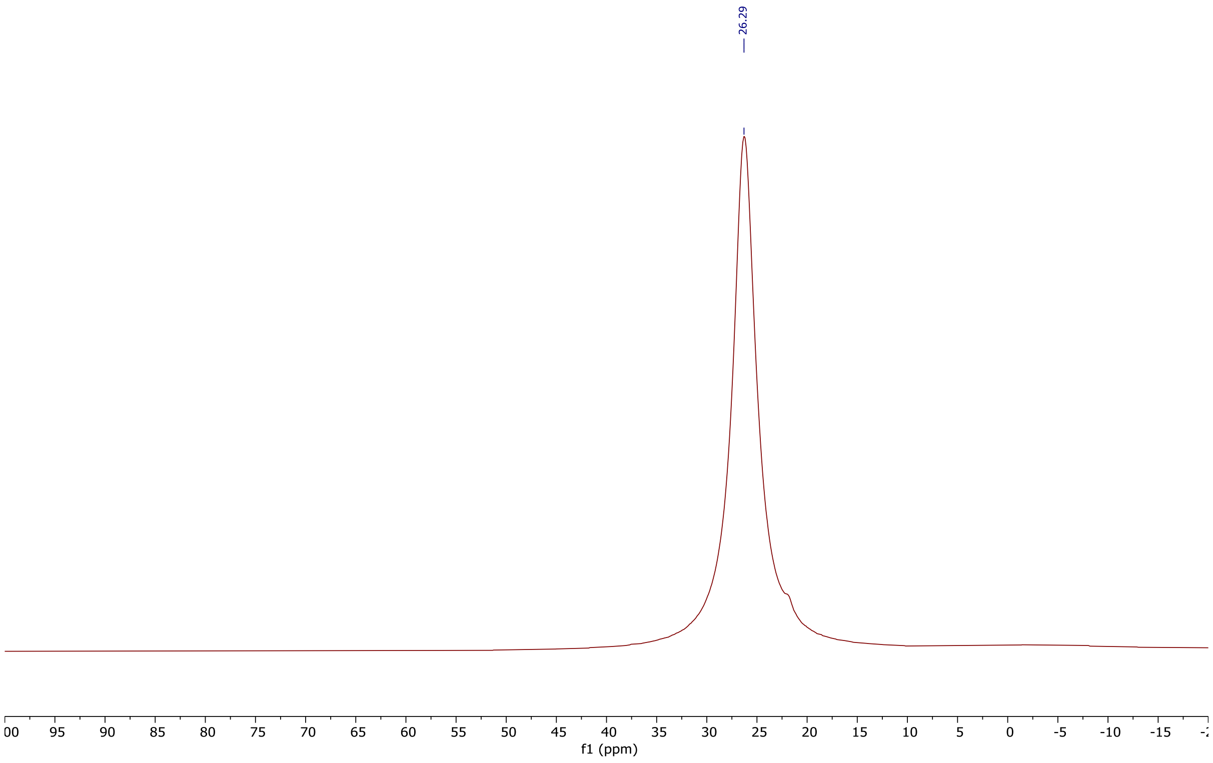

Figure S45: ^{11}B NMR spectrum (C_6D_6) of **3l**

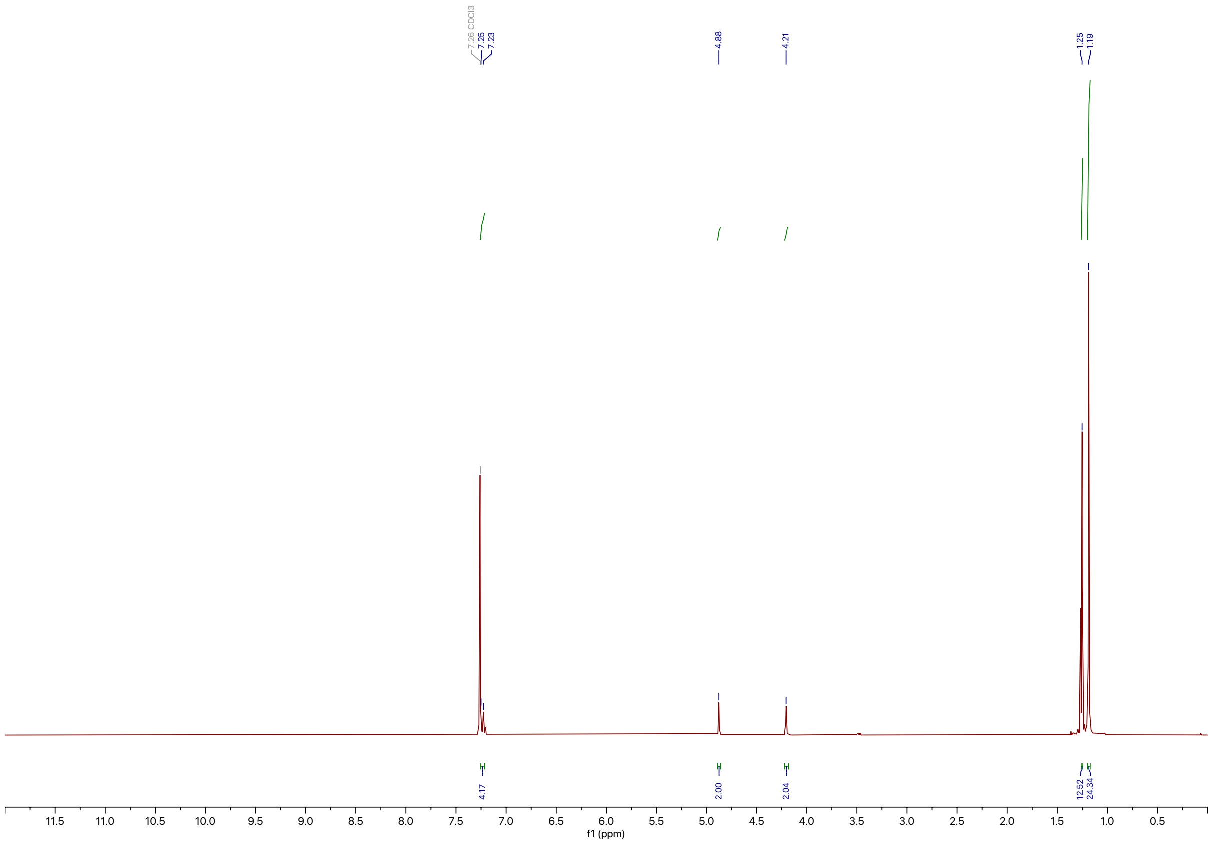

Figure S47: ^{1}H NMR spectrum ($CDCl_3$) of **3m**

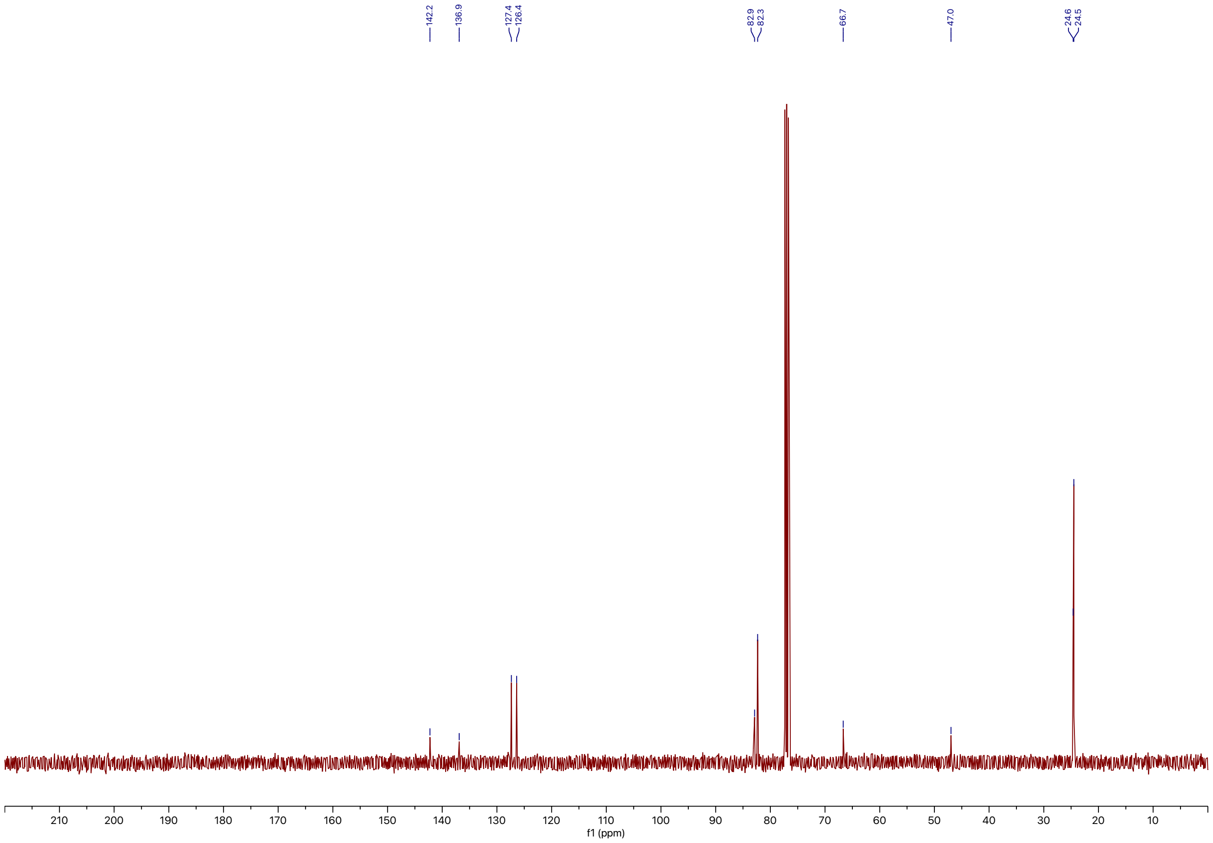

Figure S48: ^{13}C NMR spectrum ($CDCl_3$) of **3m**

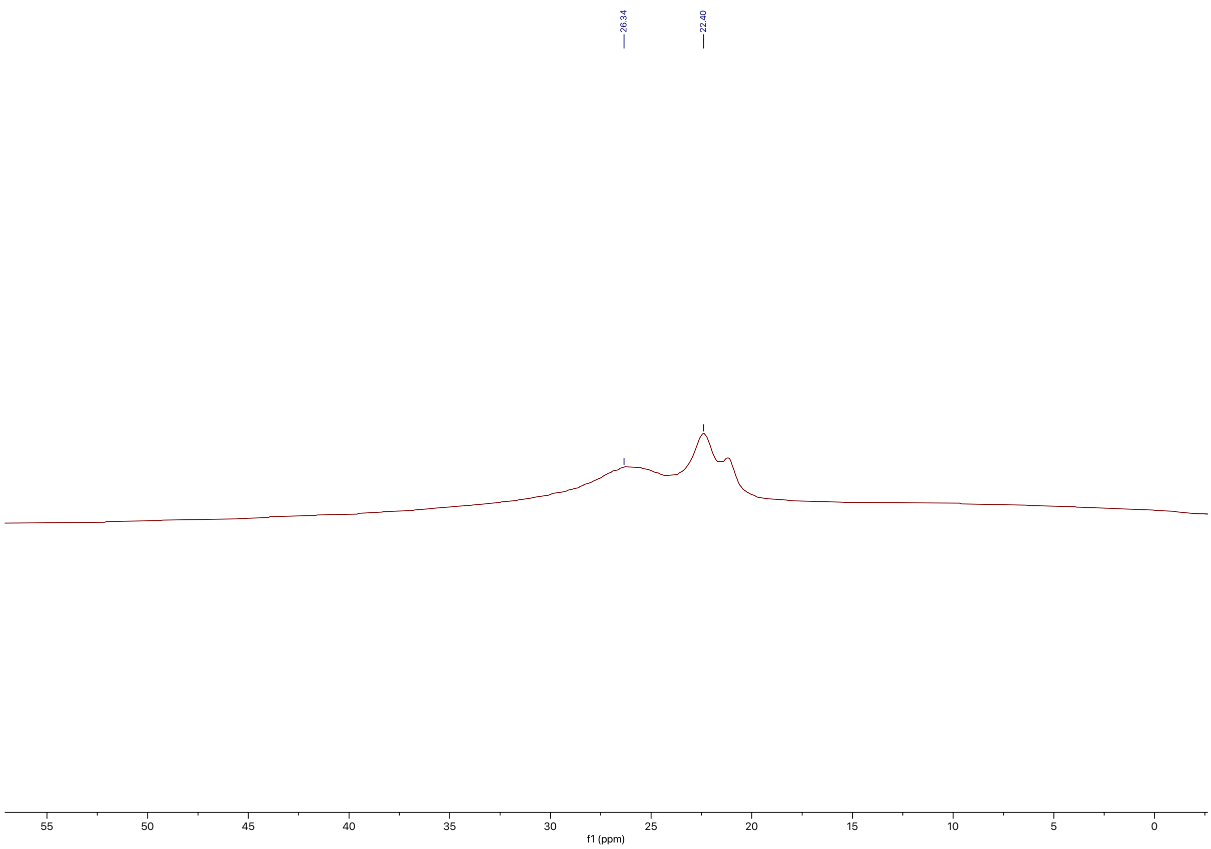

Figure S49: ^{11}B NMR spectrum ($CDCl_3$) of **3m**

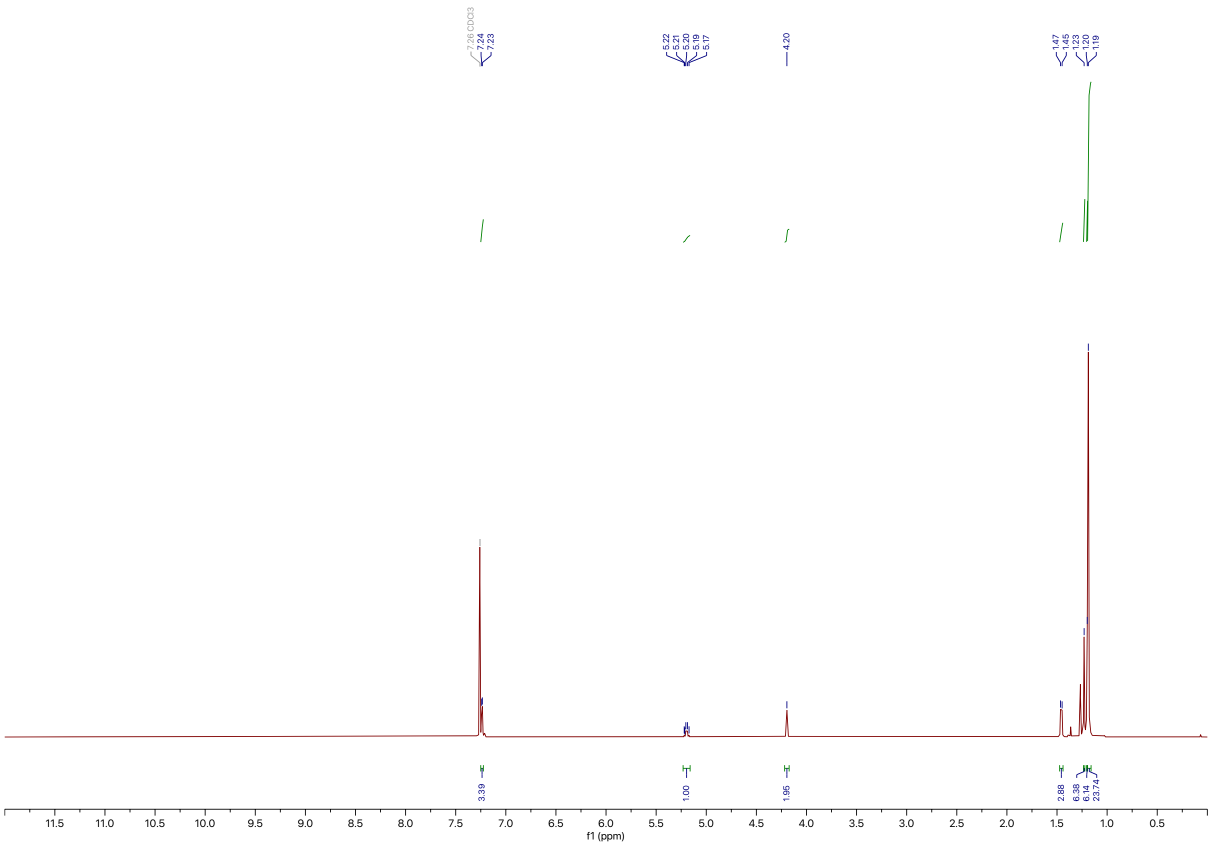

Figure S50: ^{1}H NMR spectrum ($CDCl_3$) of **3n**

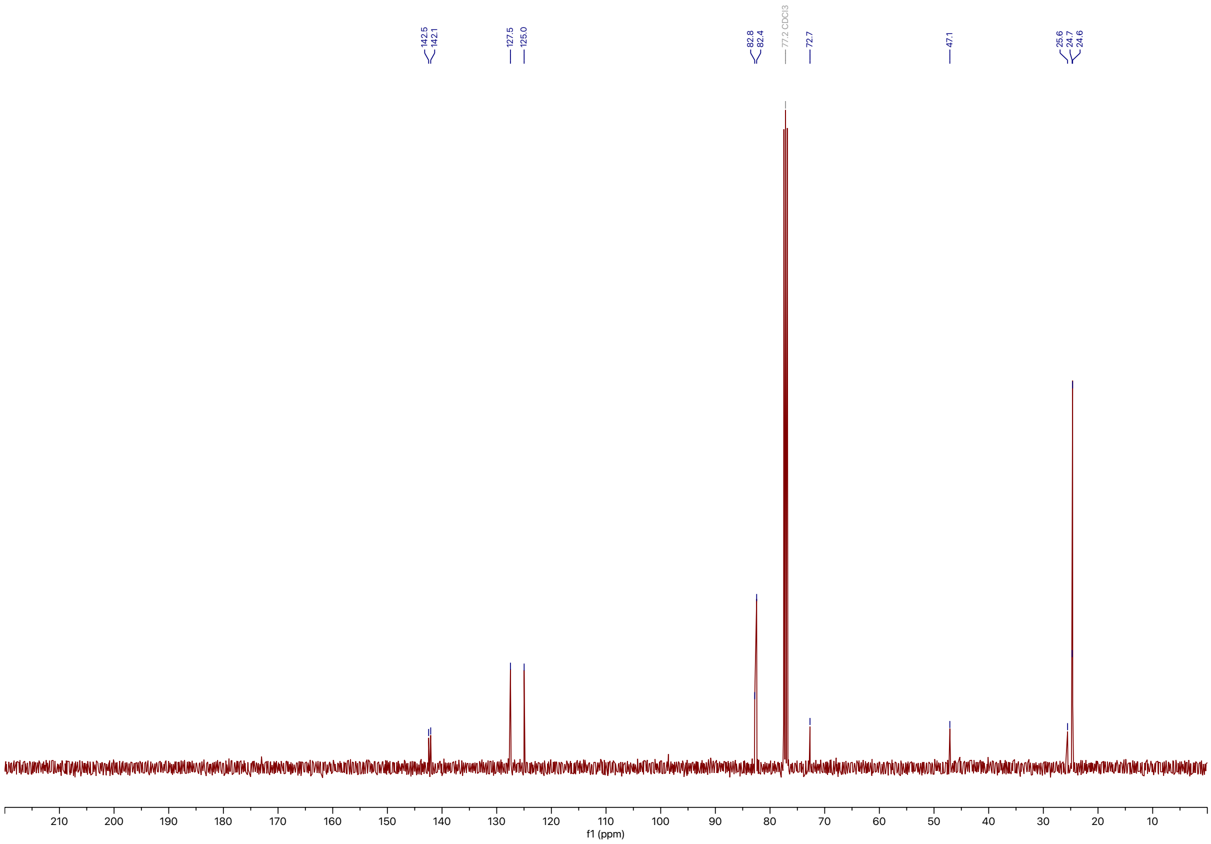

Figure S51: ^{13}C NMR spectrum ($CDCl_3$) of **3n**

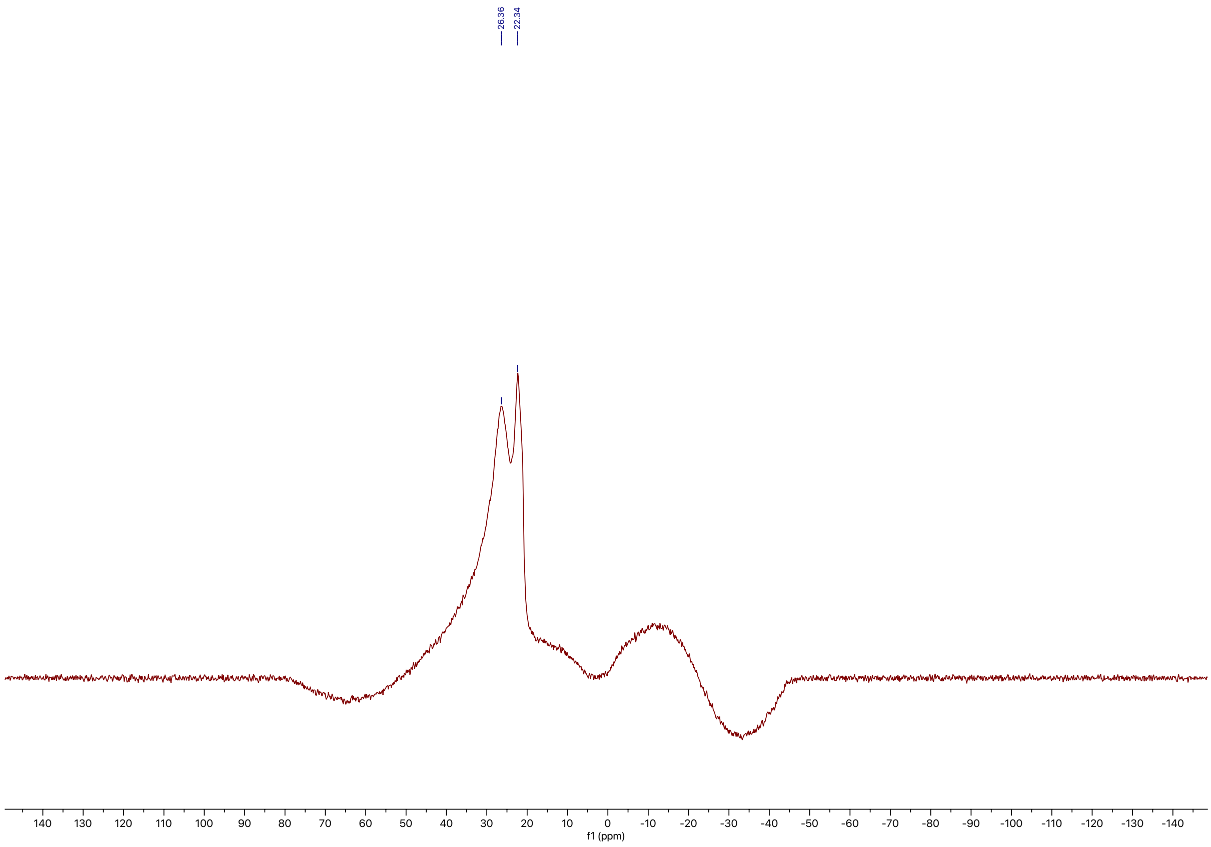

Figure S52: ^{11}B NMR spectrum ($CDCl_3$) of **3n**

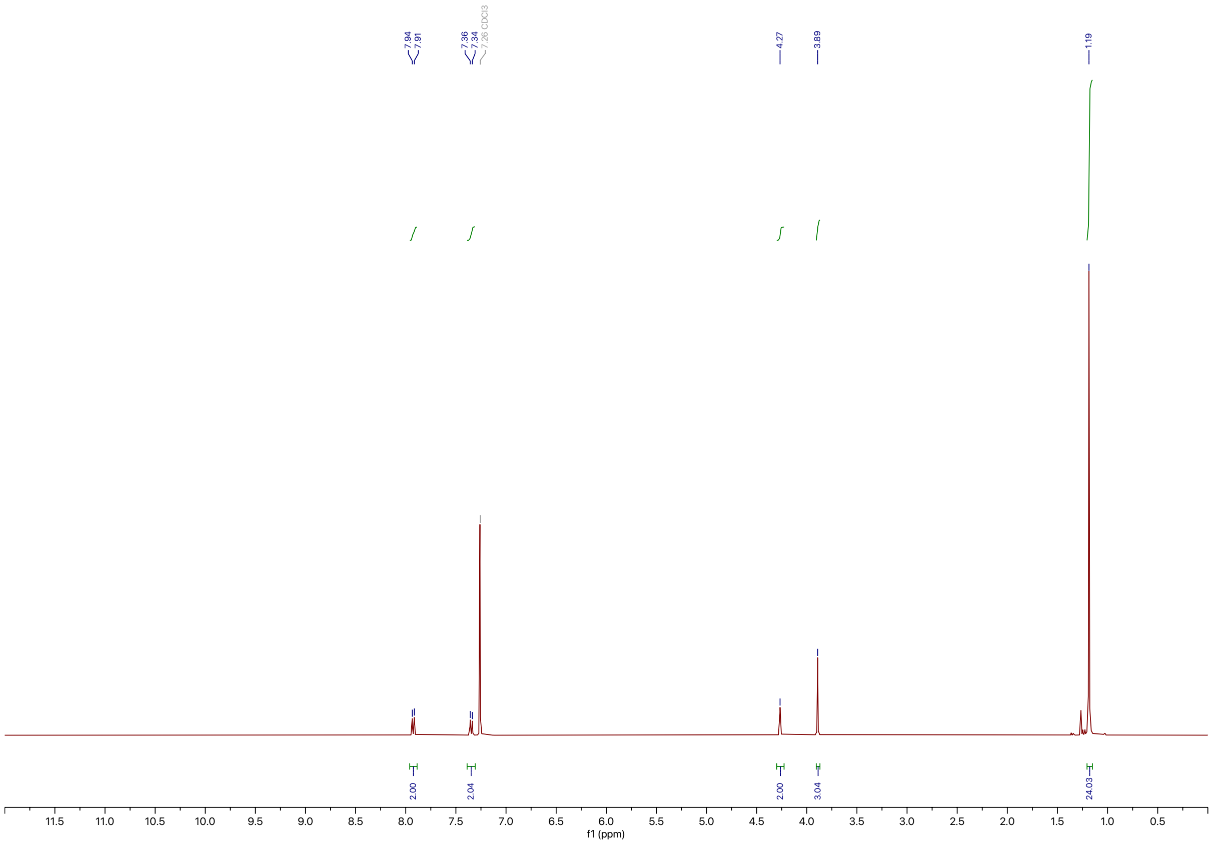

Figure S53: ^{1}H NMR spectrum ($CDCl_3$) of **3o**

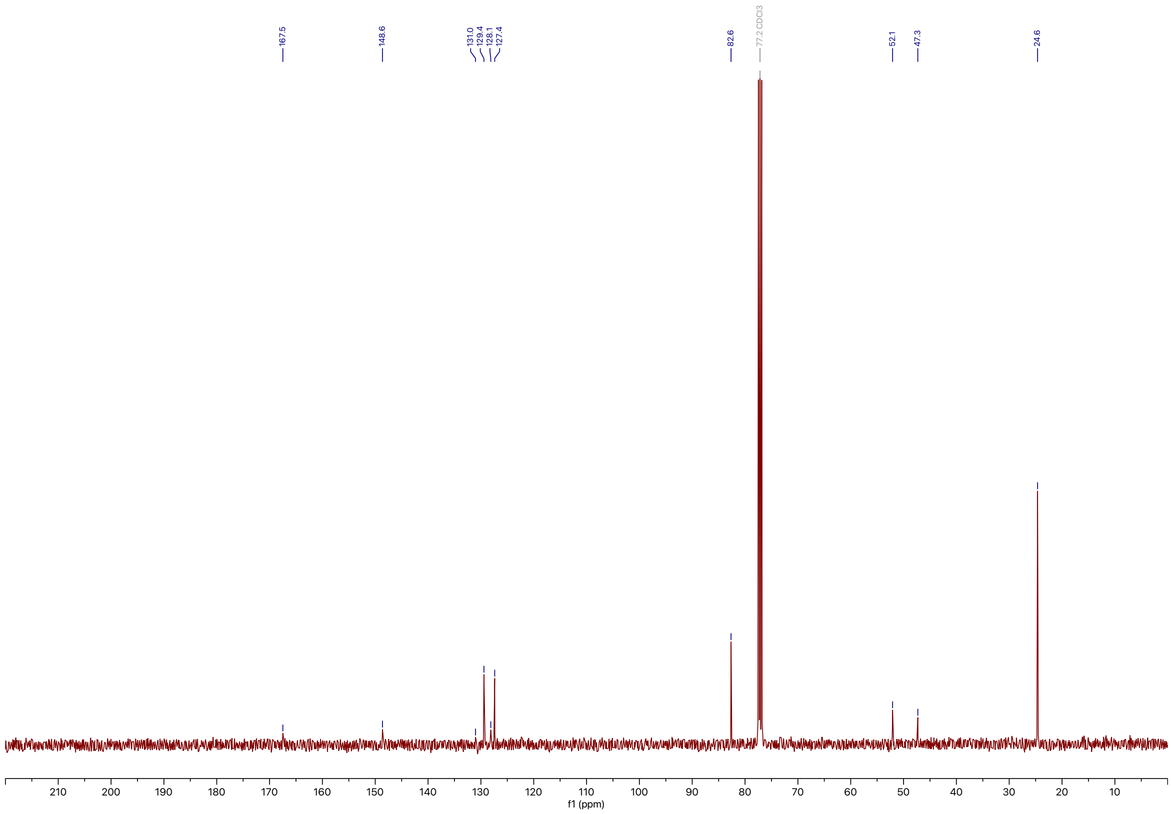

Figure S54: ^{13}C NMR spectrum ($CDCl_3$) of **3o**

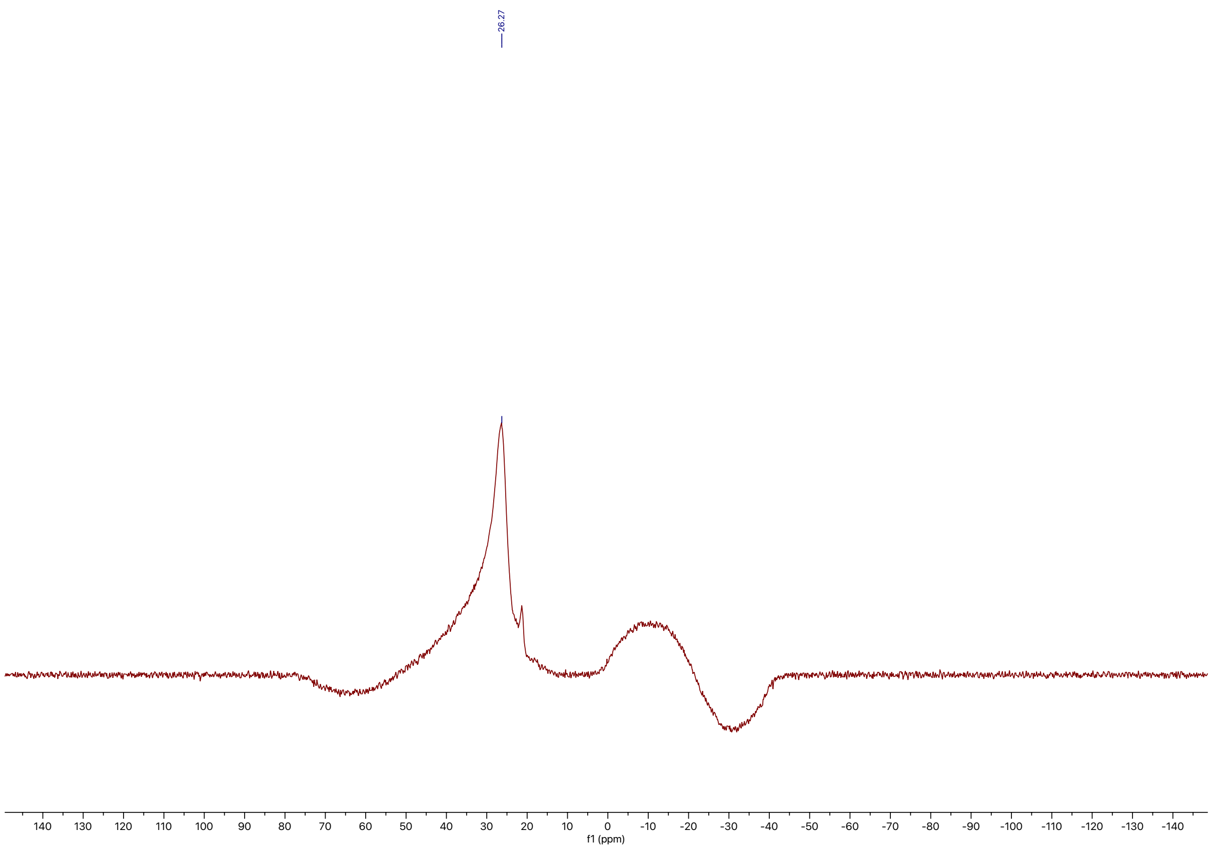

Figure S55: ^{11}B NMR spectrum ($CDCl_3$) of **3o**

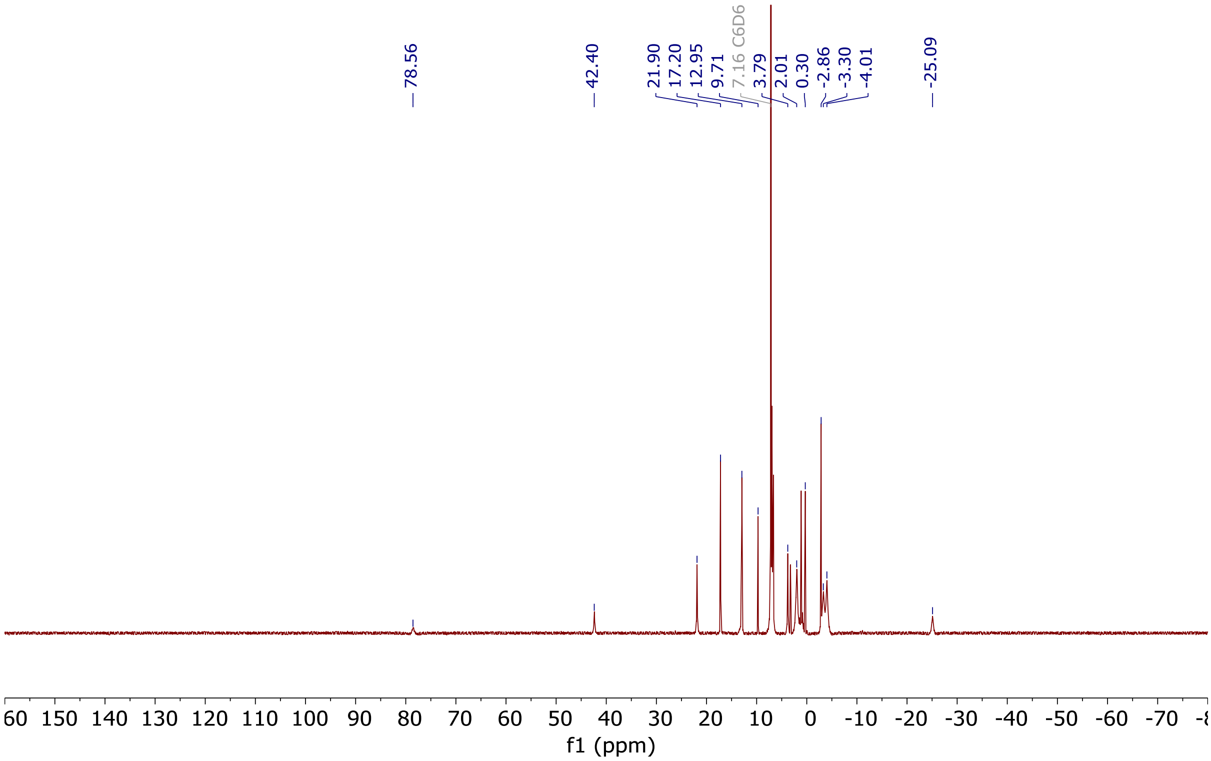

Figure S56: ^{1}H NMR spectrum (C_6D_6) of **C**

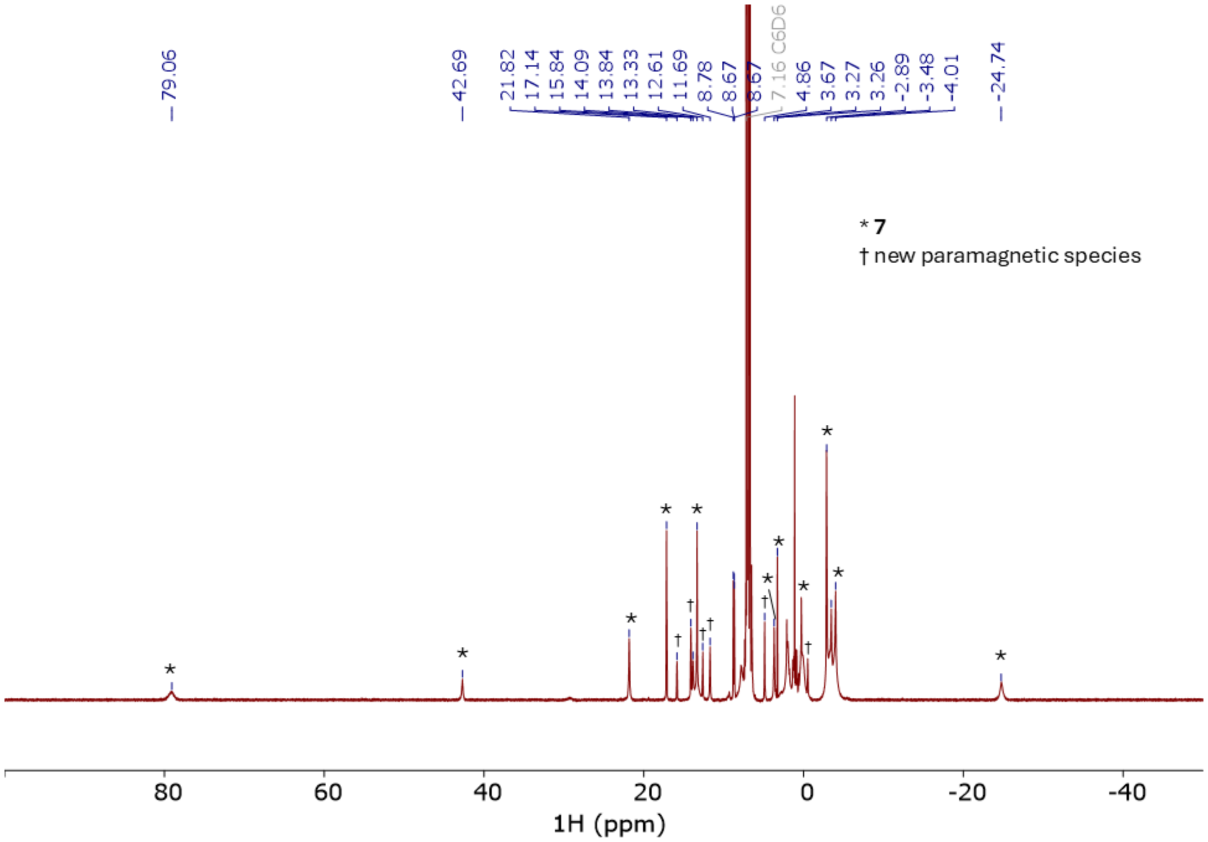

Figure S57: ^{1}H NMR spectrum (C_6D_6) of reaction of **C** and 10 equivalents of benzonitrile.

Computational Methods

All calculations were performed using the Gaussian-09 software package with the unrestricted B3LYP-GD3 hybrid density functional method.[6,7] Geometry optimizations, analytical frequency calculations, and constraint geometry scans were done with a modest basis set containing def2-SVP on all atoms (basis set BS1).[8] Single-point calculations with def2-TZVP basis set on all atoms were done on all optimized geometries to correct the energies (basis set BS2). Free energies were calculated at 323.15 K and use a solvent model (self-consistent reaction field) with a dielectric constant mimicking benzene.[9] These methods were applied previously for analogous transition metal systems and were shown to give good product distributions, rate constants, and regioselectivity predictions for inorganic reaction mechanisms.[10] To accommodate for the solvent cage around the catalytic complex the entropy contributions were scaled with Wertz's method.[11]

Computational data

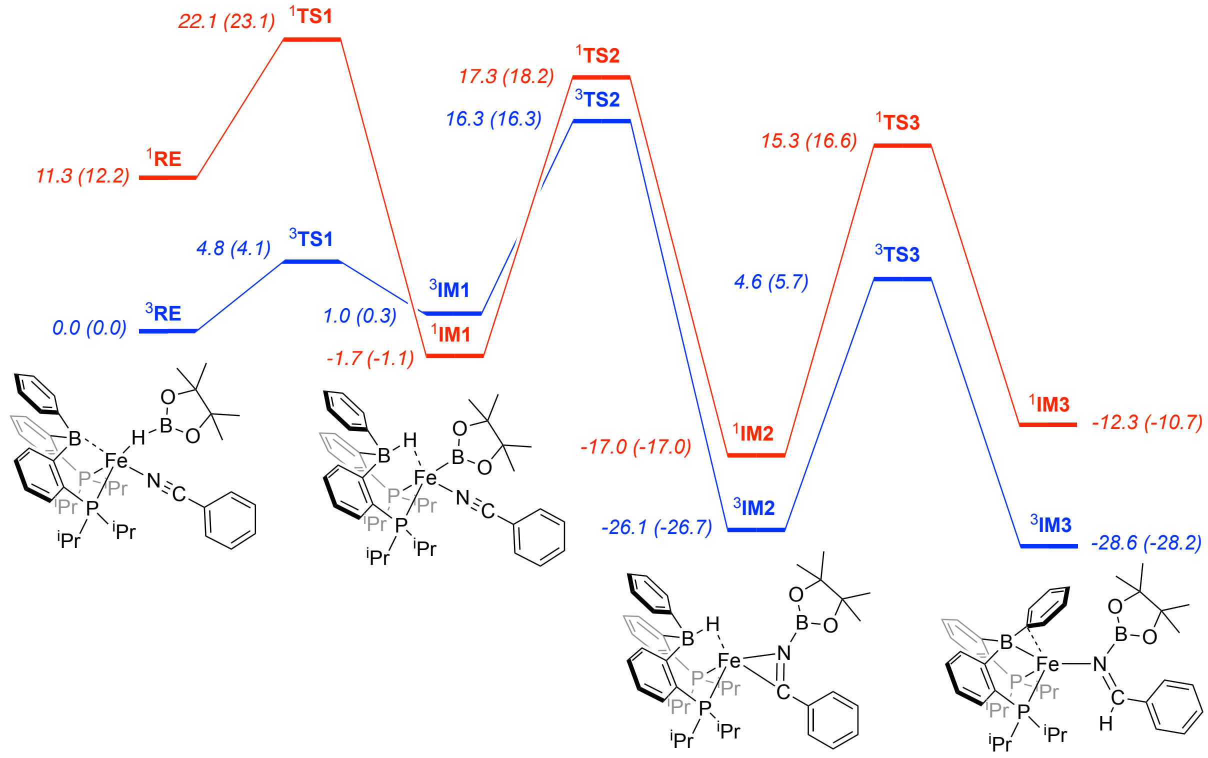

Figure S58: Energy landscape for the first reaction cycle of benzonitrile with HBPin on an iron-borane catalyst. Energies (in kcal mol^{-1}) are calculated at the UB3LYP/BS2//UB3LYP/BS1 level of theory and have been corrected with zero-point energies, while free energies with thermal corrections and Wertz corrected entropies are included at 323.15 K are in parenthesis. All calculations were done with the CPCM model with a dielectric constant mimicking benzene included.

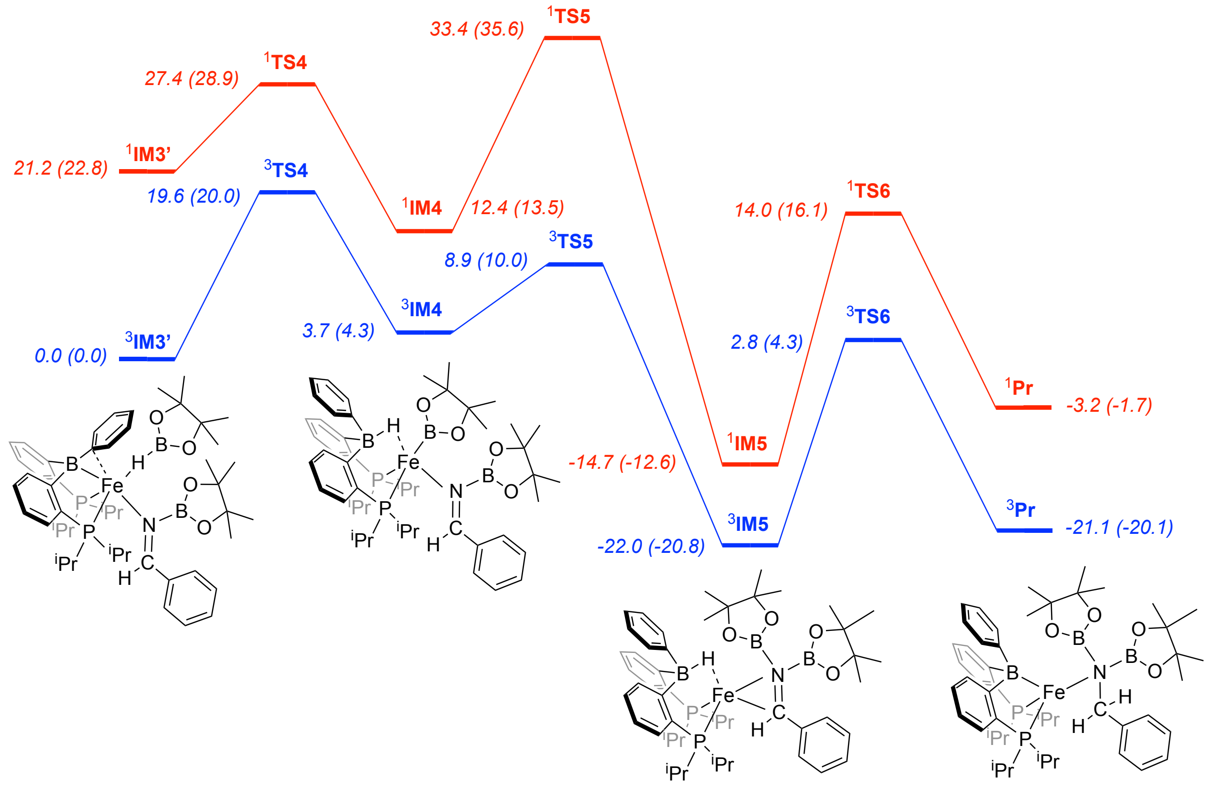

Figure S59: Energy landscape for the second reaction cycle of the reaction of the product of cycle one with HBPin on an iron-borane catalyst. Energies (in kcal mol^{-1}) are calculated at the UB3LYP/BS2//UB3LYP/BS1 level of theory and have been corrected with zero-point energies, while free energies with thermal corrections and Wertz corrected entropies are included at 323.15 K are in parenthesis. All calculations were done with the CPCM model with a dielectric constant mimicking benzene included.

Table S8: Absolute (in au) energies and free energies of optimized geometries for the first reaction cycle of benzonitrile with HBPin on an iron-borane catalyst. UB3LYP/BS1 optimized geometries calculated in the triplet and singlet spin states in Gaussian-09. Also given are the results from the UB3LYP/BS2 single-point calculations.

	E [BS1]	ZPE Correct	E+ZPE [BS1]	G [BS1]	E [BS2]	E+ZPE [BS2]	G [BS2]	S [BS1] (cal/mol K)
**^{3}Re**	-3874.211131	0.929825	-3873.281307	-3873.385364	-3876.707677	-3875.777852	-3875.881910	329.997
**^{3}TS1**	-3874.200138	0.926219	-3873.273919	-3873.379914	-3876.696496	-3875.770277	-3875.876272	333.407
**^{3}IM1**	-3874.208053	0.929475	-3873.278579	-3873.384779	-3876.705684	-3875.776209	-3875.882409	333.734
**^{3}TS2**	-3874.186578	0.928325	-3873.258254	-3873.361450	-3876.680229	-3875.751904	-3875.855100	326.951
**^{3}IM2**	-3874.256680	0.931004	-3873.325676	-3873.431110	-3876.750436	-3875.819432	-3875.924866	331.556
**^{3}TS3**	-3874.204915	0.927438	-3873.277478	-3873.374214	-3876.697910	-3875.770472	-3875.867208	310.041
**^{3}IM3**	-3874.268511	0.934385	-3873.334127	-3873.434847	-3876.757856	-3875.823471	-3875.924192	320.606
**^{1}Re**	-3874.194233	0.930870	-3873.263364	-3873.362481	-3876.690742	-3875.759872	-3875.858990	317.392
**^{1}TS1**	-3874.176921	0.930545	-3873.246376	-3873.343353	-3876.673216	-3875.742671	-3875.839648	310.340
**^{1}IM1**	-3874.218759	0.934194	-3873.284564	-3873.384906	-3876.714774	-3875.780580	-3875.880921	320.137
**^{1}TS2**	-3874.190347	0.934087	-3873.256260	-3873.355140	-3876.684390	-3875.750303	-3875.849183	316.450
**^{1}IM2**	-3874.242798	0.931550	-3873.311248	-3873.414425	-3876.736477	-3875.804927	-3875.908105	326.864
**^{1}TS3**	-3874.192706	0.931394	-3873.261312	-3873.357294	-3876.684926	-3875.753532	-3875.849513	308.412
**^{1}IM3**	-3874.246961	0.939596	-3873.307366	-3873.401673	-3876.737118	-3875.797522	-3875.891830	304.620

Table S9: Absolute (in au) energies and free energies of optimized geometries for the second reaction cycle of the product of cycle one with HBPin on an iron-borane catalyst. UB3LYP/BS1 optimized geometries calculated in the triplet and singlet spin states in Gaussian-09. Also given are the results from the UB3LYP/BS2 single-point calculations.

	E [BS1]	ZPE Correct	E+ZPE [BS1]	G [BS1]	E [BS2]	E+ZPE [BS2]	G [BS2]	S [BS1] (cal/mol K)
**^{3}IM3’**	-4285.886953	1.125171	-4284.761782	-4284.877916	-4288.832475	-4287.707304	-4287.823438	376.457
**^{3}TS4**	-4285.857089	1.122234	-4284.734855	-4284.848543	-4288.798271	-4287.676037	-4287.789725	369.873
**^{3}IM4**	-4285.885382	1.127735	-4284.757648	-4284.870354	-4288.829158	-4287.701423	-4287.814130	367.459
**^{3}TS5**	-4285.878408	1.124998	-4284.753410	-4284.864246	-4288.818060	-4287.693062	-4287.803898	363.257
**^{3}IM5**	-4285.932096	1.130520	-4284.801575	-4284.910617	-4288.872915	-4287.742395	-4287.851437	357.985
**^{3}TS6**	-4285.889241	1.126225	-4284.763016	-4284.871599	-4288.828995	-4287.702770	-4287.811353	357.826
**^{3}Pr**	-4285.928598	1.130577	-4284.798021	-4284.909166	-4288.871497	-4287.740920	-4287.852064	364.422
**^{1}IM3’**	-4285.858331	1.127853	-4284.730477	-4284.839350	-4288.801451	-4287.673598	-4287.782470	359.811
**^{1}TS4**	-4285.844559	1.124426	-4284.720133	-4284.827978	-4288.788126	-4287.663700	-4287.771545	355.453
**^{1}IM4**	-4285.875045	1.130141	-4284.744904	-4284.854769	-4288.817723	-4287.687582	-4287.797447	360.378
**^{1}TS5**	-4285.842945	1.129835	-4284.713111	-4284.818038	-4288.783930	-4287.654095	-4287.759023	348.962
**^{1}IM5**	-4285.920019	1.130724	-4284.789295	-4284.893761	-4288.861450	-4287.730726	-4287.835192	346.711
**^{1}TS6**	-4285.873692	1.129609	-4284.744083	-4284.849347	-4288.814664	-4287.685055	-4287.790319	349.330
**^{1}Pr**	-4285.900680	1.131208	-4284.769473	-4284.878835	-4288.843558	-4287.712350	-4287.821713	360.565

Table S3: Relative (in kcal mol^{-1}) energies and free energies of optimized geometries for the first reaction cycle of benzonitrile with HBPin on an iron-borane catalyst. UB3LYP/BS1 optimized geometries calculated in the triplet and singlet spin states in Gaussian-09. Also given are the results from the UB3LYP/BS2 single-point calculations.

	ΔE [BS1]	ΔE+ZPE [BS1]	ΔG [BS1]	ΔE [BS2]	ΔE+ZPE [BS2]	ΔG [BS2]	ΔG_{Wertz} [BS2]
**^{3}Re**	0.00	0.00	0.00	0.00	0.00	0.00	0.00
**^{3}TS1**	6.90	4.64	3.42	7.02	4.75	3.54	4.13
**^{3}IM1**	1.93	1.71	0.37	1.25	1.03	-0.31	0.34
**^{3}TS2**	15.41	14.47	15.01	17.22	16.28	16.82	16.29
**^{3}IM2**	-28.58	-27.84	-28.71	-26.83	-26.09	-26.96	-26.68
**^{3}TS3**	3.90	2.40	7.00	6.13	4.63	9.23	5.74
**^{3}IM3**	-36.01	-33.15	-31.05	-31.49	-28.63	-26.53	-28.17
**^{1}Re**	10.60	11.26	14.36	10.63	11.28	14.38	12.18
**^{1}TS1**	21.47	21.92	26.36	21.62	22.08	26.52	23.09
**^{1}IM1**	-4.79	-2.04	0.29	-4.45	-1.71	0.62	-1.10
**^{1}TS2**	13.04	15.72	18.97	14.61	17.29	20.54	18.17
**^{1}IM2**	-19.87	-18.79	-18.24	-18.07	-16.99	-16.44	-16.98
**^{1}TS3**	11.56	12.55	17.61	14.28	15.26	20.33	16.56
**^{1}IM3**	-22.48	-16.35	-10.23	-18.47	-12.34	-6.22	-10.65

Table S4: Relative (in kcal mol^{-1}) energies and free energies of optimized geometries for the second reaction cycle of the product of cycle one with HBPin on an iron-borane catalyst. UB3LYP/BS1 optimized geometries calculated in the triplet and singlet spin states in Gaussian-09. Also given are the results from the UB3LYP/BS2 single-point calculations.

	ΔE [BS1]	ΔE+ZPE [BS1]	ΔG [BS1]	ΔE [BS2]	ΔE+ZPE [BS2]	ΔG [BS2]	ΔG_{Wertz} [BS2]
**^{3}IM3'**	0.00	0.00	0.00	0.00	0.00	0.00	0.00
**^{3}TS4**	18.74	16.90	18.43	21.46	19.62	21.16	20.01
**^{3}IM4**	0.99	2.59	4.75	2.08	3.69	5.84	4.27
**^{3}TS5**	5.36	5.25	8.58	9.05	8.94	12.26	9.96
**^{3}IM5**	-28.33	-24.97	-20.52	-25.38	-22.02	-17.57	-20.79
**^{3}TS6**	-1.44	-0.77	3.96	2.18	2.84	7.58	4.33
**^{3}Pr**	-26.13	-22.74	-19.61	-24.49	-21.09	-17.96	-20.06
**^{1}IM3'**	17.96	19.64	24.20	19.47	21.15	25.71	22.80
**^{1}TS4**	26.60	26.14	31.34	27.83	27.36	32.56	28.90
**^{1}IM4**	7.47	10.59	14.52	9.26	12.38	16.31	13.50
**^{1}TS5**	27.62	30.54	37.57	30.46	33.39	40.42	35.62
**^{1}IM5**	-20.75	-17.26	-9.94	-18.18	-14.70	-7.38	-12.57
**^{1}TS6**	8.32	11.11	17.93	11.18	13.96	20.78	16.05
**^{1}Pr**	-8.61	-4.83	-0.58	-6.96	-3.17	1.08	-1.69

Table S5: Group charges (part a) and group spin densities (part b) of optimized geometries of the first reaction cycle calculated at UB3LYP/BS1 in Gaussian-09.

(a) Group charges.

	Fe	Iron ligand	PhCN	HBPin	Total
**^{3}Re**	0.00	-0.25	-0.02	0.26	0.00
**^{3}TS1**	-0.06	-0.17	0.04	0.19	0.00
**^{3}IM1**	-0.09	-0.12	0.04	0.17	0.00
**^{3}TS2**	-0.14	-0.06	-0.21	0.41	0.00
**^{3}IM2**	0.12	-0.04	-0.12	0.04	0.00
**^{3}TS3**	-0.03	-0.06	-0.09	0.18	0.00
**^{3}IM3**	0.26	0.03	-0.27	-0.02	0.00
**^{1}Re**	0.14	-0.20	-0.03	0.09	0.00
**^{1}TS1**	-0.14	-0.03	0.02	0.16	0.00
**^{1}IM1**	-0.24	0.12	0.03	0.10	0.00
**^{1}TS2**	-0.13	-0.08	-0.15	0.36	0.00
**^{1}IM2**	0.11	-0.04	-0.13	0.06	0.00
**^{1}TS3**	0.03	0.04	-0.08	0.01	0.00
**^{1}IM3**	0.07	0.29	-0.26	-0.09	0.00

(b) Group spin densities.

	Fe	Iron ligand	PhCN	HBPin	Total
**^{3}Re**	2.69	-0.56	-0.15	0.02	2.00
**^{3}TS1**	2.40	-0.30	-0.05	-0.06	2.00
**^{3}IM1**	2.11	0.03	-0.03	-0.10	2.00
**^{3}TS2**	2.57	0.08	-0.52	-0.13	2.00
**^{3}IM2**	2.19	0.00	-0.16	-0.03	2.00
**^{3}TS3**	2.28	-0.15	-0.12	0.00	2.00
**^{3}IM3**	2.92	-0.44	-0.48	0.00	2.00
**^{1}Re**	0.54	-0.46	-0.09	0.00	0.00
**^{1}TS1**	0.00	0.00	0.00	0.00	0.00
**^{1}IM1**	0.00	0.00	0.00	0.00	0.00
**^{1}TS2**	0.35	-0.01	-0.37	0.03	0.00
**^{1}IM2**	0.03	-0.02	-0.02	0.01	0.00
**^{1}TS3**	0.00	0.00	0.00	0.00	0.00
**^{1}IM3**	0.00	0.00	0.00	0.00	0.00

Table S6: Group charges (part a) and group spin densities (part b) of optimized geometries of the second reaction cycle calculated at UB3LYP/BS1 in Gaussian-09.

(a) Group charges.

	Fe	Iron ligand	PhCN+HBPin_1	HBPin_2	Total
**^{3}IM3'**	0.35	-0.01	-0.43	0.08	0.00
**^{3}TS4**	-0.01	0.03	-0.25	0.23	0.00
**^{3}IM4**	-0.17	-0.24	0.21	0.20	0.00
**^{3}TS5**	0.11	-0.06	-0.20	0.14	0.00
**^{3}IM5**	0.09	-0.17	-0.12	0.19	0.00
**^{3}TS6**	0.14	-0.26	-0.09	0.21	0.00
**^{3}Pr**	0.03	-0.31	-0.01	0.29	0.00
**^{1}IM3'**	0.28	0.09	-0.46	0.09	0.00
**^{1}TS4**	-0.02	0.13	-0.36	0.25	0.00
**^{1}IM4**	-0.15	-0.23	0.22	0.15	0.00
**^{1}TS5**	-0.12	-0.10	0.07	0.15	0.00
**^{1}IM5**	0.11	-0.15	-0.16	0.20	0.00
**^{1}TS6**	0.15	-0.26	-0.12	0.23	0.00
**^{1}Pr**	0.20	-0.34	-0.04	0.17	0.00

(b) Group spin densities.

	Fe	Iron ligand	PhCN+HBPin_1	HBPin_2	Total
**^{3}IM3'**	3.30	-0.47	-0.84	0.01	2.00
**^{3}TS4**	3.00	-0.14	-0.70	-0.15	2.00
**^{3}IM4**	2.29	0.02	-0.10	-0.21	2.00
**^{3}TS5**	3.15	0.06	-0.87	-0.34	2.00
**^{3}IM5**	2.26	-0.03	-0.20	-0.04	2.00
**^{3}TS6**	2.39	-0.18	-0.18	-0.03	2.00
**^{3}Pr**	2.53	-0.59	0.07	-0.01	2.00
**^{1}IM3'**	1.27	-0.36	-0.89	-0.02	0.00
**^{1}TS4**	1.17	-0.23	-0.88	-0.06	0.00
**^{1}IM4**	0.00	0.00	0.00	0.00	0.00
**^{1}TS5**	0.00	0.00	0.00	0.00	0.00
**^{1}IM5**	0.00	-0.04	0.01	0.03	0.00
**^{1}TS6**	0.00	0.00	0.00	0.00	0.00
**^{1}Pr**	0.50	-0.47	0.00	-0.03	0.00

Cartesian Coordinates of Optimized Geometries

Structures for Cycle 1:

^{3}Re

26	0.718214000	8.954402000	4.776370000
15	1.358596000	11.047672000	5.485088000
15	-0.022305000	7.790601000	2.907286000
7	2.450149000	8.063318000	4.868649000
6	-1.746642000	9.103134000	5.431410000
6	-1.657314000	9.987341000	2.843719000
6	-1.316580000	8.812041000	2.113716000
6	-0.074473000	12.156497000	5.676684000
6	1.387015000	7.631187000	1.679327000
1	2.102473000	7.005727000	2.238477000
6	-2.456615000	10.929884000	2.161282000
1	-2.705539000	11.871182000	2.653318000
6	-2.391335000	12.439337000	5.121584000
1	-3.322729000	12.071982000	4.683260000
6	-1.222710000	11.656050000	5.025201000
6	-2.948532000	10.703307000	0.874175000
1	-3.564532000	11.464147000	0.386407000
5	-1.198286000	10.175136000	4.373265000
6	2.385397000	11.263056000	7.035802000
1	2.602461000	12.339213000	7.136268000
6	0.542295000	5.078307000	3.383897000
1	0.181317000	4.121715000	3.794520000
1	1.280134000	5.478162000	4.092206000
1	1.056952000	4.860333000	2.435861000
6	-1.685186000	5.501913000	2.220762000
1	-1.281894000	5.355588000	1.207499000
1	-2.552022000	6.173460000	2.146362000
1	-2.049171000	4.522615000	2.574724000
6	-0.636848000	6.038702000	3.195883000
1	-1.127203000	6.145420000	4.174409000
6	-1.454581000	9.240511000	6.814747000
6	-1.846199000	8.562112000	0.836953000
1	-1.604504000	7.638967000	0.310830000
6	-2.550405000	7.983389000	5.084515000
6	2.372258000	11.900456000	4.141240000
1	3.127417000	11.146412000	3.857980000
6	-0.080778000	13.373928000	6.374597000
1	0.820946000	13.729224000	6.879400000
6	-2.667750000	9.503311000	0.213166000
1	-3.063950000	9.311164000	-0.786996000
6	1.097152000	6.949234000	0.340223000
1	0.459761000	7.577809000	-0.298061000
1	0.610271000	5.971407000	0.454899000
1	2.043446000	6.786916000	-0.202951000
6	-2.407752000	13.658796000	5.804208000
1	-3.334372000	14.236710000	5.863292000
6	-2.590762000	7.172253000	7.371873000
6	2.037289000	8.999481000	1.463007000
1	2.915263000	8.904102000	0.803228000
1	2.374137000	9.441180000	2.410809000
1	1.333813000	9.700493000	0.988402000
6	3.707306000	10.499507000	6.909611000
1	4.323454000	10.654886000	7.810577000
1	4.300705000	10.820132000	6.039923000
1	3.518901000	9.422611000	6.806087000
6	-1.250307000	14.133918000	6.432618000
1	-1.264503000	15.083880000	6.972947000
6	1.599101000	10.802660000	8.267132000
1	1.329951000	9.739198000	8.188067000
1	0.676143000	11.385786000	8.402076000
1	2.213460000	10.932632000	9.174002000
6	1.475676000	12.180857000	2.929253000
1	0.786996000	13.011424000	3.144914000
1	0.861249000	11.314101000	2.655756000
1	2.089757000	12.462240000	2.058505000
6	-2.957759000	7.039094000	6.022371000
6	-1.853110000	8.291375000	7.760797000
6	3.086109000	13.178222000	4.587056000
1	3.619958000	13.625900000	3.732039000
1	3.825212000	13.001708000	5.381218000
1	2.362696000	13.925824000	4.948934000
1	-2.862891000	7.861655000	4.045145000
1	-0.919797000	10.123485000	7.161396000
1	-1.579980000	8.430295000	8.809644000
1	-2.906383000	6.428712000	8.108260000
1	-3.573659000	6.192151000	5.705751000
6	6.559665000	4.608199000	4.412823000
6	6.768207000	5.952997000	4.076986000
6	5.299852000	4.190067000	4.863001000
6	5.736868000	6.880099000	4.190891000
6	4.251947000	5.097682000	4.978384000
6	4.463244000	6.459734000	4.644604000
1	7.748727000	6.281506000	3.724807000
1	5.132265000	3.142730000	5.124784000
1	5.898326000	7.928300000	3.933711000
1	3.268584000	4.781869000	5.328338000
6	3.392434000	7.370796000	4.770606000
1	7.376180000	3.888738000	4.322968000
6	1.979804000	6.486372000	8.811630000
6	1.643405000	5.051744000	8.254698000
8	1.134860000	7.334274000	7.987679000
8	1.252213000	5.344586000	6.879634000
5	0.821775000	6.642612000	6.859226000
6	3.426920000	6.908336000	8.556897000
1	3.529086000	7.980677000	8.773575000
1	4.124946000	6.355861000	9.202104000
1	3.716278000	6.745140000	7.509716000
6	1.609740000	6.710152000	10.271045000
1	0.534522000	6.572348000	10.441194000
1	2.163456000	6.017303000	10.922792000
1	1.871542000	7.737842000	10.563251000
6	2.809481000	4.074998000	8.252354000
1	3.140570000	3.875830000	9.282938000
1	2.492158000	3.120716000	7.806071000
1	3.664298000	4.452941000	7.679250000
6	0.420162000	4.421908000	8.922817000
1	-0.428893000	5.120825000	8.931557000
1	0.121997000	3.531769000	8.350065000
1	0.636033000	4.114642000	9.955961000
1	0.215575000	7.095865000	5.933688000

^{3}TS1

26	1.356748000	8.751153000	4.885383000
15	1.679011000	11.057408000	5.051696000
15	0.366601000	7.795314000	3.011206000
7	3.063382000	7.932247000	4.824442000
6	-2.050038000	8.569500000	5.852308000
6	-1.501465000	9.719065000	3.504647000
6	-1.001176000	8.883153000	2.477982000
6	0.209762000	11.816890000	5.834615000
6	1.591388000	7.569113000	1.603885000
1	2.372070000	6.979060000	2.115864000
6	-2.502850000	10.639764000	3.137693000
1	-2.913190000	11.307273000	3.898751000
6	-2.110502000	11.598787000	6.413477000

1	-3.038707000	11.022707000	6.426930000
6	-0.965632000	11.034265000	5.809147000
6	-2.976147000	10.743562000	1.828207000
1	-3.740042000	11.486359000	1.581806000
5	-1.079927000	9.599037000	5.066598000
6	3.095142000	11.520969000	6.190367000
1	3.073582000	12.615705000	6.313974000
6	0.721918000	5.048068000	3.489093000
1	0.287778000	4.113281000	3.880450000
1	1.485145000	5.377576000	4.206369000
1	1.204014000	4.810747000	2.528174000
6	-1.496271000	5.667030000	2.391449000
1	-1.131641000	5.472220000	1.372284000
1	-2.290730000	6.423240000	2.321704000
1	-1.953356000	4.734734000	2.764072000
6	-0.378385000	6.104573000	3.339979000
1	-0.821050000	6.252924000	4.337079000
6	-2.087026000	8.531297000	7.265216000
6	-1.495781000	8.961907000	1.165606000
1	-1.113271000	8.295615000	0.393452000
6	-2.920842000	7.685397000	5.186282000
6	2.011284000	12.094341000	3.515179000
1	2.850776000	11.566900000	3.029830000
6	0.234921000	13.084205000	6.443300000
1	1.161722000	13.660114000	6.478479000
6	-2.475967000	9.897860000	0.833143000
1	-2.844036000	9.967465000	-0.193521000
6	1.181097000	6.794750000	0.348239000
1	0.448425000	7.344878000	-0.259119000
1	0.757735000	5.809640000	0.581159000
1	2.067298000	6.632325000	-0.288468000
6	-2.096322000	12.862106000	7.006811000
1	-3.006470000	13.260478000	7.463870000
6	-3.781867000	6.798745000	7.272651000
6	2.208324000	8.925115000	1.247423000
1	3.047623000	8.792277000	0.545243000
1	2.589333000	9.437649000	2.142436000
1	1.469152000	9.585036000	0.768455000
6	4.441223000	11.126795000	5.574743000
1	5.264487000	11.403728000	6.253955000
1	4.626928000	11.622781000	4.610250000
1	4.491817000	10.040625000	5.409246000
6	-0.915347000	13.611642000	7.029813000
1	-0.888939000	14.593759000	7.508684000
6	2.897410000	10.874943000	7.563809000
1	2.894363000	9.778184000	7.495976000
1	1.941686000	11.173491000	8.018832000
1	3.709521000	11.177351000	8.245993000
6	0.802250000	12.039326000	2.579955000
1	-0.064188000	12.547590000	3.029360000
1	0.495631000	11.010387000	2.365178000
1	1.036082000	12.540692000	1.626567000
6	-3.765448000	6.807402000	5.876152000
6	-2.936966000	7.675096000	7.965634000
6	2.428885000	13.543439000	3.773971000
1	2.650072000	14.041541000	2.815179000
1	3.326404000	13.627858000	4.403054000
1	1.616430000	14.108410000	4.255337000
1	-2.954035000	7.692200000	4.095326000
1	-1.426204000	9.193663000	7.829528000
1	-2.938463000	7.685506000	9.059455000
1	-4.445865000	6.121786000	7.816469000
1	-4.423638000	6.135936000	5.317099000
6	7.129237000	4.452816000	5.396555000
6	7.335455000	5.798878000	5.721098000
6	5.890052000	4.031540000	4.899806000
6	6.311098000	6.727871000	5.553885000
6	4.854860000	4.946732000	4.725845000
6	5.061252000	6.304627000	5.054586000
1	8.302268000	6.126104000	6.109341000
1	5.729151000	2.981308000	4.647590000
1	6.461841000	7.777952000	5.809469000
1	3.883896000	4.628303000	4.343537000
6	3.991285000	7.227529000	4.905503000
1	7.936523000	3.729829000	5.531851000
6	1.633728000	6.741122000	8.610271000
6	1.038975000	5.608448000	7.675663000
8	1.258912000	7.959475000	7.921785000
8	0.884601000	6.294069000	6.409985000
5	0.973510000	7.654546000	6.611654000
6	3.162702000	6.729764000	8.681636000
1	3.500968000	7.639179000	9.199434000
1	3.538752000	5.856146000	9.233361000
1	3.609091000	6.727661000	7.677719000
6	1.040888000	6.782319000	10.012891000
1	-0.044594000	6.939729000	9.982283000
1	1.246848000	5.846085000	10.553972000
1	1.490720000	7.611949000	10.578378000
6	1.962665000	4.413660000	7.460790000
1	2.167729000	3.900715000	8.412849000
1	1.478218000	3.694786000	6.783321000
1	2.917736000	4.711852000	7.009956000
6	-0.349821000	5.128556000	8.093747000
1	-1.041543000	5.971509000	8.209084000
1	-0.751430000	4.477376000	7.303339000
1	-0.317852000	4.555842000	9.032228000
1	0.166942000	8.564767000	5.918618000

^{3}IM1

26	1.462028000	8.664327000	4.912338000
15	1.732850000	11.020565000	5.044548000
15	0.469725000	7.762625000	2.994351000
7	3.162721000	7.969910000	4.723823000
6	-2.144447000	8.529230000	5.940290000
6	-1.475994000	9.627751000	3.581281000
6	-0.907030000	8.868616000	2.528211000
6	0.251885000	11.726910000	5.870619000
6	1.632601000	7.494138000	1.551378000
1	2.435341000	6.926786000	2.055121000
6	-2.505987000	10.520355000	3.226280000
1	-2.970148000	11.125191000	4.008541000
6	-2.064995000	11.553046000	6.483733000
1	-3.001989000	10.992057000	6.511726000
6	-0.940574000	10.963526000	5.868758000
6	-2.942282000	10.681552000	1.910797000
1	-3.733999000	11.400161000	1.681847000
5	-1.101833000	9.506725000	5.160749000
6	3.156203000	11.477853000	6.172225000
1	3.098838000	12.562756000	6.355590000
6	0.750625000	5.035032000	3.597161000
1	0.286148000	4.121246000	4.003419000
1	1.519644000	5.356644000	4.312033000
1	1.230975000	4.762165000	2.644714000
6	-1.457130000	5.676140000	2.486191000
1	-1.099892000	5.415839000	1.479360000
1	-2.219751000	6.460177000	2.378742000
1	-1.949455000	4.782984000	2.906007000
6	-0.319029000	6.115855000	3.409388000
1	-0.755776000	6.329385000	4.398941000
6	-2.154041000	8.459315000	7.348700000
6	-1.354495000	9.016352000	1.203132000
1	-0.911653000	8.421084000	0.405960000
6	-3.044470000	7.686085000	5.263541000
6	2.009307000	12.095474000	3.525129000

1	2.864121000	11.605613000	3.026351000
6	0.302098000	12.991459000	6.486346000
1	1.236155000	13.555261000	6.509365000
6	-2.362680000	9.926076000	0.887214000
1	-2.693811000	10.041646000	-0.147801000
6	1.183549000	6.665689000	0.344089000
1	0.380401000	7.150539000	-0.228132000
1	0.834708000	5.665868000	0.633637000
1	2.034775000	6.530946000	-0.344438000
6	-2.023771000	12.815276000	7.077897000
1	-2.921761000	13.228559000	7.545458000
6	-3.898913000	6.775397000	7.340243000
6	2.229808000	8.844191000	1.137063000
1	3.051562000	8.694774000	0.418021000
1	2.631105000	9.383753000	2.007832000
1	1.476134000	9.488181000	0.659406000
6	4.501325000	11.169941000	5.506418000
1	5.327248000	11.428236000	6.189457000
1	4.651450000	11.738280000	4.576365000
1	4.586627000	10.099958000	5.267370000
6	-0.829094000	13.540332000	7.089606000
1	-0.776058000	14.521271000	7.568573000
6	3.015171000	10.751130000	7.512828000
1	3.079687000	9.661526000	7.383575000
1	2.049066000	10.962558000	7.993323000
1	3.817756000	11.065881000	8.200237000
6	0.797235000	12.009275000	2.596041000
1	-0.090221000	12.460672000	3.064730000
1	0.540624000	10.973447000	2.349657000
1	1.000326000	12.550358000	1.657548000
6	-3.904448000	6.815218000	5.944239000
6	-3.019269000	7.609330000	8.040891000
6	2.374761000	13.554406000	3.805942000
1	2.597548000	14.069593000	2.856670000
1	3.256856000	13.660252000	4.453590000
1	1.535164000	14.086245000	4.277894000
1	-3.084090000	7.714542000	4.172385000
1	-1.455909000	9.080676000	7.913153000
1	-3.006298000	7.590359000	9.134438000
1	-4.573292000	6.104353000	7.878855000
1	-4.587153000	6.172925000	5.380439000
6	7.279908000	4.524541000	4.806635000
6	7.476660000	5.736151000	4.134072000
6	6.052389000	4.252079000	5.422432000
6	6.452338000	6.679168000	4.069957000
6	5.019019000	5.184515000	5.370937000
6	5.217329000	6.406034000	4.690031000
1	8.435303000	5.946199000	3.654978000
1	5.900467000	3.306729000	5.947834000
1	6.596448000	7.624951000	3.544957000
1	4.055955000	4.994677000	5.847414000
6	4.136367000	7.330800000	4.652496000
1	8.086838000	3.789767000	4.851171000
6	1.326737000	6.842079000	8.793422000
6	1.191097000	5.605998000	7.833917000
8	1.076235000	7.952742000	7.894568000
8	1.577721000	6.179897000	6.560117000
5	1.337277000	7.548034000	6.603440000
6	2.745382000	7.026528000	9.338574000
1	2.816801000	8.017648000	9.810067000
1	2.997683000	6.265258000	10.091661000
1	3.489342000	6.982169000	8.529501000
6	0.312138000	6.885340000	9.927264000
1	-0.714687000	6.900774000	9.544058000
1	0.430484000	6.013359000	10.589486000
1	0.467082000	7.794301000	10.527827000
6	2.118536000	4.440883000	8.152574000

1	1.892998000	4.023871000	9.146410000
1	1.976038000	3.641895000	7.409106000
1	3.174131000	4.741187000	8.133664000
6	-0.249325000	5.113746000	7.678406000
1	-0.929431000	5.946605000	7.455826000
1	-0.289848000	4.404336000	6.838438000
1	-0.607312000	4.599834000	8.582888000
1	-0.040209000	8.787046000	5.451360000

^{3}TS2

26	1.365955000	8.726783000	4.726474000
15	1.761774000	11.069160000	4.848341000
15	0.343301000	7.708543000	2.858378000
7	3.020932000	7.964332000	4.896612000
6	-2.097086000	8.576603000	6.126970000
6	-1.549047000	9.595729000	3.686996000
6	-1.073190000	8.838225000	2.582176000
6	0.332792000	11.764340000	5.773337000
6	1.371421000	7.517577000	1.307041000
1	2.191864000	6.897790000	1.710359000
6	-2.601907000	10.494908000	3.417840000
1	-2.995010000	11.099510000	4.237702000
6	-1.920316000	11.603392000	6.590252000
1	-2.849306000	11.043024000	6.716411000
6	-0.851831000	10.993391000	5.899020000
6	-3.154439000	10.661020000	2.148994000
1	-3.963063000	11.380629000	1.994896000
5	-1.088524000	9.508894000	5.253993000
6	3.261791000	11.537991000	5.859730000
1	3.233179000	12.629254000	6.010712000
6	0.646313000	4.930192000	3.174251000
1	0.250013000	4.007708000	3.629417000
1	1.557829000	5.198571000	3.723540000
1	0.908783000	4.695642000	2.131514000
6	-1.682783000	5.694224000	2.484019000
1	-1.491516000	5.562656000	1.408321000
1	-2.450967000	6.471868000	2.594040000
1	-2.102508000	4.748360000	2.865488000
6	-0.408600000	6.039181000	3.258073000
1	-0.678359000	6.166362000	4.319355000
6	-2.013760000	8.549392000	7.534618000
6	-1.640834000	8.995177000	1.303264000
1	-1.275822000	8.397178000	0.470584000
6	-3.015347000	7.691441000	5.535151000
6	1.907283000	12.114616000	3.292688000
1	2.711023000	11.606456000	2.732398000
6	0.425391000	13.048672000	6.341957000
1	1.354669000	13.615735000	6.266047000
6	-2.670870000	9.905161000	1.077350000
1	-3.093337000	10.019666000	0.076067000
6	0.811912000	6.788392000	0.081194000
1	0.053845000	7.381071000	-0.449849000
1	0.367654000	5.816866000	0.333637000
1	1.629426000	6.601043000	-0.635010000
6	-1.837614000	12.884841000	7.136964000
1	-2.695793000	13.309411000	7.665069000
6	-3.705053000	6.821618000	7.690978000
6	1.972034000	8.878845000	0.937375000
1	2.745687000	8.758036000	0.162101000
1	2.432644000	9.357464000	1.814407000
1	1.203860000	9.561757000	0.543283000
6	4.543597000	11.182490000	5.098197000
1	5.426120000	11.420514000	5.714599000
1	4.638284000	11.741758000	4.155435000
1	4.576878000	10.109597000	4.860007000
6	-0.652198000	13.614923000	7.021371000
1	-0.564237000	14.612150000	7.459652000

6	3.202335000	10.859050000	7.230864000
1	3.212392000	9.763904000	7.145543000
1	2.290433000	11.137016000	7.780040000
1	4.070414000	11.163984000	7.838481000
6	0.615235000	12.021535000	2.478796000
1	-0.222871000	12.488055000	3.018165000
1	0.330053000	10.984557000	2.270342000
1	0.736550000	12.546328000	1.517293000
6	-3.805151000	6.821876000	6.297477000
6	-2.804428000	7.697209000	8.308733000
6	2.308477000	13.575084000	3.510242000
1	2.448301000	14.066573000	2.533185000
1	3.247665000	13.685124000	4.071241000
1	1.520486000	14.127085000	4.043682000
1	-3.120894000	7.682010000	4.447839000
1	-1.302944000	9.212931000	8.034773000
1	-2.712119000	7.707115000	9.398551000
1	-4.322389000	6.148100000	8.291148000
1	-4.505414000	6.145173000	5.799386000
6	6.991554000	4.911161000	6.747328000
6	7.094341000	6.297732000	6.923801000
6	5.945514000	4.386660000	5.976423000
6	6.167331000	7.157344000	6.342126000
6	5.004321000	5.228624000	5.391411000
6	5.096743000	6.631606000	5.577560000
1	7.907671000	6.712087000	7.524308000
1	5.858470000	3.306367000	5.836886000
1	6.243343000	8.237015000	6.482908000
1	4.176462000	4.822964000	4.809199000
6	4.118515000	7.480639000	5.011110000
1	7.724050000	4.243550000	7.205825000
6	1.896164000	6.514920000	8.276331000
6	1.326297000	5.427136000	7.275571000
8	1.969220000	7.707048000	7.443104000
8	1.685866000	5.971342000	5.979642000
5	1.871722000	7.323757000	6.130726000
6	3.315155000	6.220141000	8.762322000
1	3.699644000	7.106545000	9.287911000
1	3.329787000	5.371715000	9.461926000
1	3.993877000	6.000273000	7.929387000
6	0.986108000	6.817253000	9.459588000
1	0.003288000	7.175052000	9.130279000
1	0.846607000	5.918530000	10.079559000
1	1.444815000	7.596776000	10.085903000
6	1.960917000	4.049050000	7.411620000
1	1.755781000	3.626362000	8.406911000
1	1.529489000	3.372837000	6.658542000
1	3.046254000	4.083904000	7.258389000
6	-0.198042000	5.313907000	7.295087000
1	-0.677568000	6.292619000	7.169153000
1	-0.514533000	4.670658000	6.460995000
1	-0.561870000	4.866517000	8.230869000
1	-0.066062000	8.766065000	5.504620000

^{3}IM2

26	1.525994000	8.772259000	4.733559000
15	1.891217000	11.043682000	5.107120000
15	0.477171000	7.721393000	2.954258000
7	2.354325000	7.480532000	6.040627000
6	-2.126126000	8.757471000	5.622006000
6	-1.285397000	9.816167000	3.289580000
6	-0.738376000	8.933147000	2.323354000
6	0.350044000	11.872808000	5.676918000
6	1.587370000	7.109507000	1.581661000
1	2.317439000	6.524175000	2.168024000
6	-2.173523000	10.797673000	2.810857000
1	-2.609937000	11.503692000	3.521247000
6	-2.020729000	11.815477000	6.088136000
1	-2.976922000	11.286339000	6.054834000
6	-0.874488000	11.167475000	5.585482000
6	-2.503778000	10.918938000	1.458772000
1	-3.188336000	11.706553000	1.132110000
5	-1.023837000	9.706140000	4.892937000
6	3.107815000	11.399641000	6.481295000
1	3.131517000	12.492305000	6.623550000
6	0.374244000	5.085323000	3.937201000
1	-0.216656000	4.311114000	4.452768000
1	1.197938000	5.366348000	4.608499000
1	0.803680000	4.622052000	3.035609000
6	-1.741338000	5.901026000	2.752992000
1	-1.459405000	5.494784000	1.770264000
1	-2.395216000	6.769148000	2.590693000
1	-2.331150000	5.131107000	3.277634000
6	-0.525961000	6.278656000	3.600930000
1	-0.904250000	6.689972000	4.550444000
6	-1.828421000	8.160824000	6.865289000
6	-1.077257000	9.042930000	0.964624000
1	-0.654285000	8.354233000	0.234888000
6	-3.386719000	8.471233000	5.069046000
6	2.508919000	12.011753000	3.627029000
1	3.425456000	11.464394000	3.344558000
6	0.398954000	13.160710000	6.243260000
1	1.350717000	13.687000000	6.334160000
6	-1.954134000	10.036288000	0.526058000
1	-2.202628000	10.118836000	-0.534890000
6	1.012889000	6.182455000	0.505974000
1	0.257609000	6.676708000	-0.120820000
1	0.552319000	5.284832000	0.938972000
1	1.824008000	5.848749000	-0.162576000
6	-1.976421000	13.097293000	6.638731000
1	-2.890499000	13.562694000	7.017832000
6	-3.988342000	7.061878000	6.947958000
6	2.345847000	8.300785000	0.983058000
1	3.167803000	7.945786000	0.340815000
1	2.778833000	8.932084000	1.773066000
1	1.687237000	8.935797000	0.371914000
6	4.517613000	10.929426000	6.114120000
1	5.219786000	11.175787000	6.927254000
1	4.893814000	11.405527000	5.195884000
1	4.549525000	9.840615000	5.964734000
6	-0.757943000	13.778387000	6.718200000
1	-0.705670000	14.777414000	7.158236000
6	2.608361000	10.748060000	7.776475000
1	2.515974000	9.657125000	7.668252000
1	1.622622000	11.138117000	8.069309000
1	3.313194000	10.951780000	8.599283000
6	1.499926000	11.879709000	2.481797000
1	0.545243000	12.364258000	2.737431000
1	1.276187000	10.830567000	2.249688000
1	1.892901000	12.359356000	1.570614000
6	-4.307978000	7.637413000	5.716166000
6	-2.740046000	7.331503000	7.522727000
6	2.867770000	13.474983000	3.888636000
1	3.310431000	13.918312000	2.981185000
1	3.595846000	13.596092000	4.704643000
1	1.971407000	14.062432000	4.137596000
1	-3.654152000	8.902833000	4.100890000
1	-0.849027000	8.341465000	7.320427000
1	-2.479643000	6.888465000	8.488015000
1	-4.701591000	6.407821000	7.456454000
1	-5.277607000	7.433285000	5.252773000
6	6.908623000	6.402937000	3.808927000
6	6.359692000	7.552939000	3.230543000
6	6.201178000	5.703884000	4.795287000

6	5.102652000	8.000100000	3.636635000
6	4.949817000	6.156127000	5.211760000
6	4.389952000	7.312494000	4.633878000
1	6.912371000	8.096000000	2.460535000
1	6.630222000	4.802190000	5.238622000
1	4.653955000	8.891678000	3.194677000
1	4.385215000	5.609618000	5.969874000
6	3.068256000	7.804636000	5.021775000
1	7.890503000	6.046768000	3.487797000
6	1.543607000	5.989152000	9.255276000
6	1.444667000	4.763998000	8.265798000
8	1.626518000	7.111821000	8.341293000
8	2.164568000	5.258582000	7.102435000
5	2.090136000	6.630056000	7.144267000
6	2.830470000	5.988849000	10.082312000
1	2.932378000	6.964540000	10.579273000
1	2.815568000	5.205693000	10.853857000
1	3.714190000	5.836190000	9.445341000
6	0.331942000	6.182812000	10.155406000
1	-0.581355000	6.341390000	9.569360000
1	0.187112000	5.308055000	10.807462000
1	0.483685000	7.065531000	10.793824000
6	2.116648000	3.489487000	8.753761000
1	1.647898000	3.138201000	9.685536000
1	2.004572000	2.699455000	7.996414000
1	3.188722000	3.639515000	8.934232000
6	0.012555000	4.478095000	7.810602000
1	-0.486131000	5.390521000	7.453282000
1	0.040735000	3.758082000	6.980220000
1	-0.590293000	4.046988000	8.622785000
1	0.007511000	9.041714000	5.240743000

^{3}TS3

26	1.625041000	8.757314000	3.961551000
15	1.958116000	11.053190000	4.040718000
15	0.164316000	7.983701000	2.364976000
7	3.122935000	7.705003000	4.705462000
6	-1.235041000	8.372615000	6.104600000
6	-1.486022000	9.646153000	3.760025000
6	-1.318402000	9.041098000	2.491608000
6	0.862094000	11.689208000	5.359230000
6	0.814552000	7.939785000	0.605148000
1	1.751723000	7.374514000	0.758160000
6	-2.603097000	10.493274000	3.906915000
1	-2.777937000	10.976963000	4.870202000
6	-1.020138000	11.345099000	6.806549000
1	-1.843106000	10.726138000	7.169380000
6	-0.188268000	10.842716000	5.781408000
6	-3.482810000	10.763089000	2.857096000
1	-4.321578000	11.447513000	3.011360000
5	-0.549232000	9.418512000	5.078444000
6	3.688399000	11.458766000	4.642923000
1	3.756192000	12.553854000	4.739548000
6	0.630832000	5.227956000	2.600581000
1	0.322104000	4.264277000	3.037886000
1	1.551942000	5.549700000	3.110635000
1	0.876259000	5.046222000	1.542085000
6	-1.794968000	5.845273000	2.084105000
1	-1.681423000	5.657122000	1.007602000
1	-2.573923000	6.610663000	2.212178000
1	-2.161819000	4.912492000	2.545041000
6	-0.484248000	6.266197000	2.750390000
1	-0.700919000	6.383202000	3.823062000
6	-0.819207000	8.284918000	7.451094000
6	-2.213583000	9.290226000	1.437362000
1	-2.069863000	8.809905000	0.469675000
6	-2.286916000	7.518334000	5.719498000
6	1.725982000	12.218900000	2.578793000
1	2.267005000	11.709606000	1.763220000
6	1.070771000	12.950349000	5.946029000
1	1.908951000	13.572704000	5.627059000
6	-3.288906000	10.162056000	1.609935000
1	-3.969768000	10.367453000	0.780131000
6	0.025804000	7.219475000	-0.492071000
1	-0.953164000	7.681534000	-0.682878000
1	-0.140041000	6.160126000	-0.257110000
1	0.593173000	7.260404000	-1.437218000
6	-0.828910000	12.602430000	7.381227000
1	-1.501377000	12.948143000	8.171178000
6	-2.461664000	6.578876000	7.947397000
6	1.209028000	9.359518000	0.186544000
1	1.772656000	9.340453000	-0.760280000
1	1.840095000	9.830817000	0.953082000
1	0.323769000	9.997474000	0.041156000
6	4.712878000	10.987808000	3.605173000
1	5.736041000	11.233078000	3.934516000
1	4.560576000	11.461129000	2.623049000
1	4.654346000	9.897247000	3.466043000
6	0.227844000	13.413145000	6.955493000
1	0.398209000	14.392134000	7.410432000
6	3.946776000	10.842060000	6.020055000
1	3.897504000	9.745789000	5.980847000
1	3.215242000	11.189118000	6.763675000
1	4.952992000	11.118374000	6.375510000
6	0.242704000	12.278300000	2.205181000
1	-0.338070000	12.787788000	2.989235000
1	-0.189415000	11.279797000	2.077903000
1	0.110598000	12.837824000	1.264798000
6	-2.888359000	6.629652000	6.618184000
6	-1.421098000	7.417843000	8.361588000
6	2.308722000	13.625944000	2.729677000
1	2.157255000	14.186857000	1.792371000
1	3.387723000	13.624059000	2.940444000
1	1.801501000	14.187148000	3.528523000
1	-2.659860000	7.560493000	4.693742000
1	0.005946000	8.916038000	7.790743000
1	-1.065429000	7.379288000	9.394594000
1	-2.932144000	5.890557000	8.654347000
1	-3.703240000	5.982942000	6.280201000
6	1.235493000	4.769256000	8.471935000
6	0.408385000	4.783033000	7.345661000
6	2.302779000	5.669259000	8.562729000
6	0.632994000	5.703288000	6.324085000
6	2.551693000	6.570684000	7.527610000
6	1.709109000	6.600052000	6.400823000
1	-0.433114000	4.091902000	7.270782000
1	2.951390000	5.663097000	9.442041000
1	-0.032877000	5.734589000	5.465666000
1	3.399089000	7.253861000	7.583003000
6	1.981646000	7.542508000	5.303075000
1	1.047072000	4.060332000	9.281778000
6	6.572166000	6.715304000	4.105136000
6	6.613787000	7.173807000	5.615445000
8	5.278592000	7.204459000	3.671468000
8	5.204246000	7.208829000	5.961035000
5	4.498041000	7.347621000	4.791144000
6	6.552767000	5.194178000	3.942610000
1	6.313009000	4.952133000	2.896967000
1	7.527491000	4.748540000	4.187720000
1	5.786512000	4.734165000	4.583684000
6	7.650497000	7.330099000	3.223495000
1	7.575748000	8.424663000	3.197460000
1	8.652064000	7.053016000	3.586157000
1	7.544201000	6.956730000	2.194184000

6	7.325634000	6.212821000	6.556814000
1	8.377409000	6.086432000	6.258181000
1	7.307388000	6.615680000	7.580351000
1	6.841663000	5.228118000	6.570402000
6	7.150253000	8.594611000	5.795701000
1	6.656903000	9.295982000	5.109415000
1	6.943749000	8.924741000	6.824080000
1	8.235577000	8.641521000	5.626671000
1	0.785570000	8.433969000	5.251180000

[3]IM3

26	1.168841000	8.815138000	5.020282000
15	1.947798000	11.063782000	5.002429000
15	0.089834000	7.472889000	3.295116000
7	2.919945000	8.112452000	5.585240000
6	-1.171304000	9.239791000	5.798894000
6	-1.417463000	9.785662000	3.127374000
6	-1.219390000	8.510909000	2.534041000
6	0.588580000	12.262855000	5.235884000
6	1.266427000	7.063649000	1.890353000
1	2.037641000	6.464989000	2.403272000
6	-2.245262000	10.688704000	2.426074000
1	-2.385977000	11.696309000	2.819605000
6	-1.799154000	12.534211000	5.135159000
1	-2.800308000	12.126816000	4.971480000
6	-0.673181000	11.705863000	4.951961000
6	-2.898692000	10.337399000	1.244156000
1	-3.531620000	11.068349000	0.733648000
5	-0.805849000	10.156341000	4.553656000
6	3.179698000	11.566409000	6.324700000
1	3.305199000	12.653049000	6.193427000
6	0.322439000	4.678546000	3.744298000
1	-0.035234000	3.833485000	4.351896000
1	1.310628000	4.950030000	4.131220000
1	0.440746000	4.321521000	2.711527000
6	-2.019746000	5.464659000	3.214814000
1	-1.923303000	5.207707000	2.149053000
1	-2.762900000	6.269310000	3.299922000
1	-2.423804000	4.577115000	3.729924000
6	-0.674624000	5.836029000	3.840470000
1	-0.846576000	6.029305000	4.910420000
6	-0.637505000	9.522875000	7.082824000
6	-1.910786000	8.143319000	1.367773000
1	-1.783940000	7.149691000	0.943222000
6	-2.103798000	8.169890000	5.733370000
6	2.743348000	11.591126000	3.384215000
1	3.417882000	10.752436000	3.146760000
6	0.726727000	13.592822000	5.665682000
1	1.711864000	14.006492000	5.891995000
6	-2.753369000	9.049372000	0.721529000
1	-3.273412000	8.757068000	-0.193972000
6	0.722594000	6.256801000	0.705102000
1	0.125139000	6.893869000	0.037858000
1	0.104824000	5.399172000	1.000864000
1	1.567489000	5.869278000	0.111572000
6	-1.670753000	13.859411000	5.556842000
1	-2.562816000	14.476013000	5.696471000
6	-1.926347000	7.748759000	8.113127000
6	1.923650000	8.351995000	1.390412000
1	2.636885000	8.119291000	0.582942000
1	2.479820000	8.859337000	2.187216000
1	1.170554000	9.038406000	0.976212000
6	4.552645000	10.907064000	6.179185000
1	5.279113000	11.410592000	6.837755000
1	4.938480000	10.952221000	5.152148000
1	4.502443000	9.850873000	6.469382000
6	-0.404208000	14.395718000	5.818818000
1	-0.301609000	15.431182000	6.152834000
6	2.599179000	11.316927000	7.719551000
1	2.425763000	10.242613000	7.884653000
1	1.649249000	11.849475000	7.873672000
1	3.306612000	11.662939000	8.490667000
6	1.662607000	11.700303000	2.302512000
1	1.042389000	12.594973000	2.463827000
1	0.990184000	10.835059000	2.293084000
1	2.130464000	11.783546000	1.308313000
6	-2.483509000	7.447916000	6.859159000
6	-0.997952000	8.783174000	8.217363000
6	3.550674000	12.889306000	3.461361000
1	3.951188000	13.133285000	2.463271000
1	4.403172000	12.824709000	4.150950000
1	2.916060000	13.733710000	3.773402000
1	-2.566451000	7.934547000	4.773716000
1	0.018941000	10.382787000	7.209109000
1	-0.563106000	9.035220000	9.188005000
1	-2.223165000	7.180474000	8.998013000
1	-3.222926000	6.647857000	6.768248000
6	1.489190000	3.276131000	6.934132000
6	0.566362000	4.196929000	7.447345000
6	2.618246000	3.745423000	6.256186000
6	0.776118000	5.565181000	7.288473000
6	2.820171000	5.115032000	6.074556000
6	1.899653000	6.050820000	6.589425000
1	-0.321647000	3.844002000	7.978441000
1	3.351487000	3.036677000	5.862174000
1	0.058222000	6.279570000	7.691583000
1	3.714460000	5.469348000	5.563870000
6	2.107829000	7.503962000	6.472488000
1	1.330982000	2.203275000	7.067923000
6	5.589309000	7.942979000	3.138488000
6	6.300712000	7.399450000	4.438664000
8	4.382092000	8.527059000	3.667805000
8	5.182282000	7.140901000	5.311704000
5	4.106322000	7.907769000	4.883576000
6	5.177284000	6.820106000	2.182342000
1	4.541284000	7.238815000	1.391168000
1	6.049745000	6.346788000	1.709828000
1	4.601387000	6.044327000	2.708405000
6	6.364763000	9.016626000	2.386628000
1	6.543783000	9.897699000	3.015950000
1	7.334373000	8.628086000	2.039395000
1	5.789827000	9.338841000	1.505427000
6	7.080415000	6.103644000	4.247407000
1	7.890900000	6.239656000	3.514901000
1	7.532584000	5.802413000	5.204181000
1	6.431912000	5.286045000	3.907647000
6	7.190123000	8.443034000	5.119299000
1	6.667036000	9.400416000	5.238427000
1	7.460478000	8.076391000	6.120358000
1	8.116021000	8.618126000	4.552130000
1	1.820195000	8.066320000	7.375868000

[1]Re

26	0.532771000	8.868808000	4.955425000
15	1.311705000	10.875357000	5.662653000
15	-0.162058000	7.566398000	3.203553000
7	2.240399000	8.188085000	4.849971000
6	-1.827682000	9.321041000	5.492655000
6	-1.629627000	9.890644000	2.829949000
6	-1.379934000	8.596012000	2.282920000
6	0.019000000	12.165483000	5.582337000
6	1.218757000	7.285899000	1.951411000
1	1.942070000	6.698026000	2.538813000
6	-2.285620000	10.809871000	1.979884000

1	-2.444264000	11.831826000	2.325477000
6	-2.140814000	12.743009000	4.702114000
1	-3.063779000	12.481050000	4.179529000
6	-1.117004000	11.782348000	4.829912000
6	-2.753354000	10.459893000	0.712663000
1	-3.261253000	11.205982000	0.095128000
5	-1.272828000	10.258488000	4.339864000
6	2.203255000	11.142138000	7.285877000
1	2.547826000	12.188659000	7.307756000
6	0.285814000	4.811695000	3.657874000
1	-0.104547000	3.887990000	4.115925000
1	1.095032000	5.171752000	4.305941000
1	0.700343000	4.542710000	2.675521000
6	-1.994192000	5.348792000	2.667729000
1	-1.673233000	5.149732000	1.634152000
1	-2.823811000	6.068981000	2.629149000
1	-2.390614000	4.403125000	3.074922000
6	-0.846204000	5.842108000	3.549697000
1	-1.245926000	5.978332000	4.564985000
6	-1.705539000	9.679391000	6.871276000
6	-1.890984000	8.233648000	1.026384000
1	-1.729059000	7.229663000	0.638844000
6	-2.573372000	8.127903000	5.247100000
6	2.542581000	11.495992000	4.367821000
1	3.203852000	10.630763000	4.192997000
6	0.124777000	13.436431000	6.166519000
1	1.005209000	13.703076000	6.755457000
6	-2.588330000	9.154332000	0.241078000
1	-2.972548000	8.861296000	-0.739048000
6	0.887780000	6.499168000	0.679060000
1	0.310118000	7.112021000	-0.026992000
1	0.324562000	5.576391000	0.868895000
1	1.824952000	6.216276000	0.170589000
6	-2.036286000	14.016974000	5.267968000
1	-2.854532000	14.732803000	5.150270000
6	-2.802961000	7.649878000	7.613418000
6	1.883032000	8.613898000	1.582032000
1	2.742045000	8.433353000	0.914702000
1	2.246662000	9.149144000	2.465608000
1	1.177483000	9.271271000	1.050697000
6	3.419423000	10.214011000	7.359470000
1	3.961636000	10.368216000	8.307078000
1	4.129683000	10.385032000	6.536796000
1	3.097225000	9.166582000	7.320274000
6	-0.900661000	14.370288000	6.003959000
1	-0.819147000	15.362168000	6.455615000
6	1.264010000	10.908552000	8.472808000
1	0.893493000	9.873589000	8.482241000
1	0.401699000	11.591029000	8.452632000
1	1.806460000	11.081270000	9.417426000
6	1.797832000	11.815201000	3.064886000
1	1.240970000	12.759208000	3.159781000
1	1.072993000	11.036537000	2.795385000
1	2.514386000	11.923118000	2.234634000
6	-3.034450000	7.308379000	6.270901000
6	-2.160181000	8.860776000	7.901354000
6	3.394265000	12.692340000	4.796139000
1	4.045702000	13.003062000	3.961986000
1	4.043581000	12.472289000	5.655245000
1	2.761374000	13.555902000	5.054448000
1	-2.795468000	7.851443000	4.216363000
1	-1.232509000	10.624222000	7.131488000
1	-2.007741000	9.166379000	8.939974000
1	-3.152592000	6.999516000	8.419172000
1	-3.585243000	6.395632000	6.026448000
6	7.035267000	5.764400000	4.389533000
6	6.982608000	7.097819000	4.815663000
6	5.852156000	5.088383000	4.064436000
6	5.761518000	7.758011000	4.920386000
6	4.622401000	5.733479000	4.161296000
6	4.563774000	7.080504000	4.593708000
1	7.903908000	7.626590000	5.070113000
1	5.889701000	4.048474000	3.732341000
1	5.715379000	8.794647000	5.257678000
1	3.697404000	5.211106000	3.911571000
6	3.309223000	7.723166000	4.719368000
1	7.996861000	5.252711000	4.311500000
6	2.105574000	6.279782000	8.576210000
6	1.490458000	5.098349000	7.719611000
8	1.328225000	7.427364000	8.132135000
8	0.895074000	5.798217000	6.597711000
5	0.720487000	7.100082000	6.957795000
6	3.571134000	6.561602000	8.248399000
1	3.883731000	7.485512000	8.753987000
1	4.220068000	5.743908000	8.592776000
1	3.722716000	6.695219000	7.170162000
6	1.925056000	6.137915000	10.081881000
1	0.865278000	6.081212000	10.360426000
1	2.437217000	5.236655000	10.451402000
1	2.363151000	7.011491000	10.586822000
6	2.508227000	4.100435000	7.182770000
1	3.027000000	3.590859000	8.008744000
1	1.991967000	3.337895000	6.581162000
1	3.256886000	4.586618000	6.544590000
6	0.348744000	4.365957000	8.426134000
1	-0.408999000	5.072218000	8.794884000
1	-0.138978000	3.692812000	7.706076000
1	0.713763000	3.765637000	9.271678000
1	-0.033093000	7.829315000	6.378980000

[1]TS1

26	1.124262000	8.569634000	4.990508000
15	1.599029000	10.692251000	5.250613000
15	0.459487000	8.078734000	2.894906000
7	2.862285000	7.909555000	4.844562000
6	-2.220427000	8.496039000	5.850083000
6	-1.731503000	9.555266000	3.413879000
6	-0.999384000	9.013289000	2.335833000
6	0.094805000	11.657202000	5.643258000
6	1.650599000	7.788847000	1.467087000
1	2.486845000	7.294804000	1.992291000
6	-2.932380000	10.216435000	3.083457000
1	-3.548414000	10.633953000	3.882351000
6	-2.275541000	11.670060000	6.016549000
1	-3.237650000	11.152373000	6.042254000
6	-1.129710000	10.961977000	5.589964000
6	-3.354110000	10.382455000	1.763087000
1	-4.280793000	10.922855000	1.550992000
5	-1.286709000	9.471106000	4.981711000
6	2.775061000	11.183405000	6.636104000
1	3.017202000	12.240055000	6.445540000
6	0.872419000	5.314769000	3.514205000
1	0.426206000	4.379787000	3.890857000
1	1.609481000	5.641932000	4.257671000
1	1.396573000	5.075798000	2.577025000
6	-1.349490000	5.865463000	2.394645000
1	-0.980689000	5.597255000	1.395034000
1	-2.139114000	6.618298000	2.266823000
1	-1.811178000	4.965070000	2.833475000
6	-0.231952000	6.359879000	3.317488000
1	-0.686637000	6.536236000	4.305498000
6	-2.311925000	8.621541000	7.254824000
6	-1.416118000	9.169644000	1.003265000
1	-0.830643000	8.748328000	0.187168000

6	-3.004461000	7.480995000	5.266404000
6	2.338020000	11.532770000	3.732252000
1	2.964897000	10.735198000	3.301441000
6	0.158012000	13.005729000	6.040155000
1	1.117984000	13.524991000	6.079466000
6	-2.587654000	9.867665000	0.712074000
1	-2.904550000	10.001246000	-0.325224000
6	1.212028000	6.858219000	0.329060000
1	0.343433000	7.243038000	-0.222783000
1	0.962413000	5.851081000	0.682876000
1	2.038490000	6.756454000	-0.394266000
6	-2.221078000	13.004761000	6.416437000
1	-3.132900000	13.517643000	6.734986000
6	-3.887466000	6.786907000	7.414889000
6	2.181388000	9.115596000	0.915427000
1	2.951848000	8.923402000	0.151122000
1	2.637061000	9.727051000	1.703114000
1	1.384106000	9.710528000	0.445769000
6	4.069908000	10.367839000	6.567561000
1	4.814186000	10.777495000	7.270162000
1	4.518712000	10.370916000	5.561654000
1	3.876350000	9.323322000	6.847047000
6	-0.998147000	13.687036000	6.415111000
1	-0.945009000	14.733603000	6.725402000
6	2.138786000	11.093248000	8.026234000
1	1.857305000	10.062356000	8.274319000
1	1.248205000	11.730505000	8.110707000
1	2.868011000	11.433645000	8.781229000
6	1.218675000	11.873772000	2.743274000
1	0.616921000	12.714727000	3.119010000
1	0.535291000	11.032271000	2.577742000
1	1.645134000	12.164926000	1.769975000
6	-3.816096000	6.632431000	6.028419000
6	-3.130935000	7.796041000	8.024789000
6	3.229725000	12.753426000	3.974514000
1	3.590421000	13.137692000	3.005794000
1	4.113935000	12.523977000	4.585305000
1	2.676832000	13.572118000	4.460359000
1	-2.993509000	7.359993000	4.181677000
1	-1.719881000	9.393037000	7.755812000
1	-3.178613000	7.933838000	9.108937000
1	-4.526156000	6.133327000	8.014744000
1	-4.405808000	5.855310000	5.533373000
6	7.519895000	5.205492000	4.973353000
6	7.516085000	6.585451000	5.208136000
6	6.323664000	4.543048000	4.673807000
6	6.326177000	7.307481000	5.148285000
6	5.125499000	5.250195000	4.605979000
6	5.119066000	6.641975000	4.845740000
1	8.448685000	7.102776000	5.443524000
1	6.324882000	3.466295000	4.490981000
1	6.315193000	8.381711000	5.338467000
1	4.188656000	4.741373000	4.372584000
6	3.891466000	7.356024000	4.807414000
1	8.456057000	4.645430000	5.024863000
6	1.800354000	6.637894000	8.663579000
6	0.920355000	5.671965000	7.774067000
8	1.479260000	7.947090000	8.123294000
8	0.735733000	6.452315000	6.567106000
5	1.037479000	7.781099000	6.832265000
6	3.304507000	6.437428000	8.462798000
1	3.842408000	7.261506000	8.953732000
1	3.649775000	5.489260000	8.899290000
1	3.569080000	6.449107000	7.396656000
6	1.457476000	6.627554000	10.146589000
1	0.416712000	6.928575000	10.319741000
1	1.610465000	5.624827000	10.574020000
1	2.111370000	7.331379000	10.682782000
6	1.598771000	4.359614000	7.399793000
1	1.833271000	3.771112000	8.300037000
1	0.922217000	3.764157000	6.769055000
1	2.526450000	4.527980000	6.837807000
6	-0.467034000	5.407422000	8.353864000
1	-0.972992000	6.344587000	8.616310000
1	-1.082659000	4.908020000	7.591879000
1	-0.418472000	4.761814000	9.243087000
1	0.174746000	8.754608000	6.236655000

[1]IM1

26	1.235883000	8.668937000	4.759632000
15	1.748993000	10.697781000	5.617007000
15	0.195244000	7.276489000	3.302062000
7	2.962339000	8.209247000	4.387172000
6	-2.382717000	8.698627000	5.654097000
6	-1.280169000	9.630940000	3.382290000
6	-0.813630000	8.602811000	2.517576000
6	0.217734000	11.639137000	5.980863000
6	1.265053000	6.479109000	1.977673000
1	1.944485000	5.878012000	2.608184000
6	-1.961838000	10.706980000	2.780686000
1	-2.319868000	11.521427000	3.414272000
6	-2.184556000	11.753562000	6.024014000
1	-3.159972000	11.291611000	5.849143000
6	-1.023463000	11.021594000	5.701757000
6	-2.193152000	10.774230000	1.405922000
1	-2.725955000	11.629614000	0.981644000
5	-1.172024000	9.563290000	5.001346000
6	2.929153000	11.090504000	7.017183000
1	3.223623000	12.145740000	6.891561000
6	-0.330738000	4.651700000	4.203468000
1	-1.013535000	4.010294000	4.783922000
1	0.576202000	4.812241000	4.799607000
1	-0.063717000	4.096530000	3.293077000
6	-2.254504000	5.813797000	2.993354000
1	-1.997788000	5.405266000	2.004813000
1	-2.775470000	6.768341000	2.843189000
1	-2.963930000	5.117136000	3.470340000
6	-1.025118000	5.980873000	3.887567000
1	-1.360824000	6.420230000	4.839545000
6	-2.235769000	8.154013000	6.946336000
6	-1.057502000	8.666747000	1.134604000
1	-0.714374000	7.868741000	0.479023000
6	-3.605110000	8.465938000	5.001809000
6	2.539786000	11.574530000	4.150839000
1	3.527627000	11.086777000	4.079757000
6	0.274987000	12.931229000	6.536889000
1	1.239730000	13.391344000	6.758703000
6	-1.743775000	9.744417000	0.574946000
1	-1.924154000	9.778288000	-0.502373000
6	0.611714000	5.529764000	0.964667000
1	-0.088021000	6.047391000	0.293732000
1	0.066186000	4.707939000	1.441783000
1	1.394739000	5.080524000	0.330954000
6	-2.131700000	13.036601000	6.569803000
1	-3.056715000	13.570257000	6.805095000
6	-4.453340000	7.178957000	6.873954000
6	2.124736000	7.524795000	1.251851000
1	2.912889000	7.017358000	0.671129000
1	2.612021000	8.219938000	1.945104000
1	1.528721000	8.120373000	0.545942000
6	4.182999000	10.220725000	6.873504000
1	4.905165000	10.457641000	7.671744000
1	4.689936000	10.374113000	5.908971000
1	3.923422000	9.156336000	6.951548000

6	-0.893255000	13.634609000	6.827084000
1	-0.838427000	14.636084000	7.261180000
6	2.275034000	10.927926000	8.392976000
1	1.847184000	9.923649000	8.501245000
1	1.470302000	11.659128000	8.549119000
1	3.029520000	11.080536000	9.182865000
6	1.746435000	11.229558000	2.887162000
1	0.731190000	11.650360000	2.924532000
1	1.639129000	10.142458000	2.747401000
1	2.250072000	11.626694000	1.990710000
6	-4.629724000	7.719073000	5.597770000
6	-3.247639000	7.404332000	7.549947000
6	2.740411000	13.083315000	4.279334000
1	3.286180000	13.464553000	3.400016000
1	3.321226000	13.360602000	5.171714000
1	1.774215000	13.607969000	4.323486000
1	-3.760655000	8.870625000	3.997969000
1	-1.294473000	8.312892000	7.479350000
1	-3.096776000	6.989090000	8.550895000
1	-5.247105000	6.589155000	7.340050000
1	-5.567068000	7.552955000	5.058970000
6	8.070947000	6.624389000	3.709480000
6	7.643851000	7.118455000	4.947316000
6	7.172608000	6.543836000	2.639204000
6	6.325407000	7.533537000	5.121958000
6	5.850642000	6.953463000	2.798693000
6	5.416614000	7.453081000	4.044983000
1	8.343992000	7.180464000	5.783202000
1	7.505140000	6.157720000	1.673233000
1	5.985223000	7.919305000	6.083831000
1	5.143685000	6.893316000	1.969994000
6	4.068163000	7.872170000	4.218164000
1	9.105849000	6.300670000	3.578792000
6	1.604914000	6.923048000	8.546865000
6	1.422924000	5.642969000	7.653166000
8	1.207626000	7.978082000	7.643420000
8	1.617193000	6.177991000	6.321968000
5	1.350323000	7.544840000	6.327675000
6	3.065827000	7.168567000	8.935611000
1	3.161094000	8.170709000	9.375938000
1	3.416319000	6.432123000	9.673398000
1	3.722351000	7.119116000	8.055118000
6	0.718708000	6.981162000	9.784628000
1	-0.345859000	6.959536000	9.519491000
1	0.931203000	6.137812000	10.459724000
1	0.912338000	7.914749000	10.334085000
6	2.448082000	4.540568000	7.889615000
1	2.394555000	4.170514000	8.925337000
1	2.241759000	3.695139000	7.216141000
1	3.470461000	4.887825000	7.692635000
6	0.007332000	5.065972000	7.732859000
1	-0.749639000	5.833550000	7.522116000
1	-0.103333000	4.266172000	6.989326000
1	-0.193695000	4.637010000	8.725361000
1	-0.208027000	8.826781000	5.456510000

[1]TS2

26	1.101365000	8.830762000	4.696190000
15	1.580828000	10.909170000	5.503948000
15	0.105091000	7.370404000	3.298801000
7	2.784043000	8.268417000	4.558117000
6	-2.506940000	8.705223000	5.675406000
6	-1.365480000	9.717631000	3.442687000
6	-0.877360000	8.726601000	2.543859000
6	0.015937000	11.768315000	5.943948000
6	1.195140000	6.579593000	1.996708000
1	1.823830000	5.933039000	2.635299000
6	-2.030724000	10.819964000	2.867429000
1	-2.407865000	11.605652000	3.525307000
6	-2.380022000	11.731912000	6.172548000
1	-3.334908000	11.210221000	6.067832000
6	-1.202875000	11.070982000	5.769425000
6	-2.222521000	10.947293000	1.491898000
1	-2.748090000	11.818300000	1.091355000
5	-1.303759000	9.606803000	5.070616000
6	2.732298000	11.322279000	6.920779000
1	2.874438000	12.414825000	6.880648000
6	-0.471322000	4.724936000	4.147376000
1	-1.156201000	4.101964000	4.745676000
1	0.463300000	4.846992000	4.707558000
1	-0.256557000	4.170345000	3.222253000
6	-2.363559000	5.958381000	2.967309000
1	-2.111318000	5.572369000	1.968335000
1	-2.869456000	6.924223000	2.842391000
1	-3.084218000	5.260871000	3.425469000
6	-1.130918000	6.080094000	3.865325000
1	-1.455149000	6.504212000	4.827925000
6	-2.357680000	8.087394000	6.933008000
6	-1.067677000	8.859521000	1.156709000
1	-0.695936000	8.095031000	0.477165000
6	-3.731883000	8.511023000	5.015637000
6	2.334384000	11.848471000	4.059190000
1	3.330635000	11.380366000	3.971337000
6	0.032545000	13.064405000	6.492525000
1	0.978641000	13.585355000	6.648468000
6	-1.738202000	9.960815000	0.627506000
1	-1.881530000	10.047285000	-0.452380000
6	0.540632000	5.685596000	0.936154000
1	-0.108712000	6.249968000	0.252221000
1	-0.059455000	4.879438000	1.373696000
1	1.326476000	5.215398000	0.321778000
6	-2.367220000	13.021607000	6.706132000
1	-3.303719000	13.499624000	7.006442000
6	-4.571832000	7.115794000	6.812610000
6	2.120021000	7.618663000	1.347661000
1	2.908963000	7.106519000	0.773521000
1	2.604873000	8.258403000	2.094676000
1	1.571105000	8.269221000	0.651397000
6	4.098449000	10.651973000	6.730315000
1	4.790405000	10.985457000	7.521065000
1	4.557013000	10.904466000	5.762043000
1	4.002187000	9.561057000	6.798555000
6	-1.152885000	13.696007000	6.867874000
1	-1.127308000	14.701737000	7.294740000
6	2.104669000	10.964673000	8.272161000
1	1.866177000	9.894289000	8.310819000
1	1.187758000	11.540266000	8.460341000
1	2.817851000	11.189761000	9.082817000
6	1.555187000	11.540088000	2.777498000
1	0.522014000	11.912236000	2.836894000
1	1.503063000	10.458930000	2.580482000
1	2.040813000	12.017101000	1.910610000
6	-4.753520000	7.730141000	5.570984000
6	-3.364762000	7.302153000	7.497561000
6	2.511042000	13.354705000	4.247217000
1	3.053871000	13.776654000	3.385121000
1	3.084109000	13.607492000	5.151558000
1	1.536340000	13.862184000	4.305194000
1	-3.890627000	8.974096000	4.037740000
1	-1.417963000	8.224625000	7.477507000
1	-3.209538000	6.829999000	8.471920000
1	-5.363204000	6.498294000	7.245556000
1	-5.692853000	7.595043000	5.027207000
6	7.406657000	5.563565000	4.988207000

6	7.430179000	6.940755000	5.247202000
6	6.221814000	4.959020000	4.546140000
6	6.287602000	7.714498000	5.069982000
6	5.067227000	5.713550000	4.365835000
6	5.083954000	7.107311000	4.633149000
1	8.351744000	7.414763000	5.592931000
1	6.198263000	3.885112000	4.346411000
1	6.301502000	8.785619000	5.277221000
1	4.135664000	5.246817000	4.043911000
6	3.901763000	7.853607000	4.475443000
1	8.308185000	4.963827000	5.129652000
6	2.616923000	6.794804000	8.020362000
6	1.901945000	5.644539000	7.210517000
8	2.293792000	7.962527000	7.235419000
8	1.847508000	6.191712000	5.875198000
5	1.883607000	7.585534000	5.964154000
6	4.140534000	6.659191000	8.057995000
1	4.571446000	7.592638000	8.449020000
1	4.453501000	5.834672000	8.715428000
1	4.558595000	6.484949000	7.060046000
6	2.086599000	7.003823000	9.435099000
1	1.023001000	7.273742000	9.433082000
1	2.219981000	6.094214000	10.040519000
1	2.642505000	7.819942000	9.920231000
6	2.664497000	4.326236000	7.169153000
1	2.790790000	3.917346000	8.183403000
1	2.100579000	3.591481000	6.574928000
1	3.654551000	4.447194000	6.711921000
6	0.459657000	5.408084000	7.668013000
1	-0.114178000	6.344568000	7.664183000
1	-0.031995000	4.713107000	6.974357000
1	0.420235000	4.972201000	8.676465000
1	-0.318073000	8.904943000	5.539664000

^{1}IM2

26	1.476295000	8.787122000	4.741051000
15	1.889982000	11.028073000	5.137866000
15	0.478578000	7.691678000	2.988075000
7	2.354466000	7.500739000	6.013424000
6	-2.147263000	8.769931000	5.617024000
6	-1.264327000	9.809931000	3.292468000
6	-0.720270000	8.910485000	2.338940000
6	0.348829000	11.873868000	5.683160000
6	1.594991000	7.064889000	1.626870000
1	2.325795000	6.489316000	2.222059000
6	-2.132529000	10.800537000	2.795658000
1	-2.564534000	11.520117000	3.494965000
6	-2.026146000	11.832971000	6.071208000
1	-2.984978000	11.308947000	6.032576000
6	-0.878894000	11.174667000	5.585129000
6	-2.449922000	10.912408000	1.439883000
1	-3.120471000	11.706260000	1.099594000
5	-1.026649000	9.708300000	4.900895000
6	3.084057000	11.364675000	6.535241000
1	3.121899000	12.457761000	6.671384000
6	0.357421000	5.062953000	3.982781000
1	-0.238608000	4.294977000	4.501544000
1	1.182111000	5.342754000	4.653310000
1	0.784925000	4.592562000	3.084148000
6	-1.750773000	5.888229000	2.789270000
1	-1.468377000	5.484136000	1.805850000
1	-2.401318000	6.759160000	2.628695000
1	-2.343828000	5.118856000	3.311004000
6	-0.535532000	6.259766000	3.639982000
1	-0.913336000	6.677052000	4.587152000
6	-1.859423000	8.151198000	6.851886000
6	-1.046338000	9.011647000	0.976254000
1	-0.626341000	8.310214000	0.257009000
6	-3.410807000	8.509147000	5.058674000
6	2.542285000	11.986235000	3.667197000
1	3.453622000	11.423744000	3.399005000
6	0.399939000	13.166103000	6.239107000
1	1.353928000	13.687498000	6.335134000
6	-1.906086000	10.011940000	0.520690000
1	-2.145482000	10.086628000	-0.542904000
6	1.021675000	6.122957000	0.563210000
1	0.265923000	6.608883000	-0.069649000
1	0.562135000	5.229747000	1.006233000
1	1.833446000	5.782556000	-0.101105000
6	-1.979608000	13.119026000	6.611842000
1	-2.894549000	13.592726000	6.978359000
6	-4.033072000	7.079204000	6.915578000
6	2.350390000	8.248700000	1.010025000
1	3.182530000	7.885253000	0.385963000
1	2.768675000	8.901303000	1.789710000
1	1.693546000	8.863379000	0.376734000
6	4.493651000	10.868876000	6.203036000
1	5.185295000	11.132166000	7.019887000
1	4.889929000	11.312580000	5.277058000
1	4.515684000	9.775747000	6.088451000
6	-0.758151000	13.794089000	6.697424000
1	-0.704743000	14.796418000	7.129722000
6	2.545602000	10.728759000	7.822472000
1	2.439284000	9.638877000	7.718321000
1	1.560244000	11.136387000	8.091432000
1	3.235699000	10.926622000	8.659074000
6	1.547094000	11.868459000	2.508160000
1	0.594606000	12.363011000	2.752659000
1	1.315253000	10.822366000	2.268951000
1	1.957775000	12.346472000	1.604007000
6	-4.343850000	7.678295000	5.692669000
6	-2.782029000	7.323308000	7.495699000
6	2.920067000	13.443953000	3.933044000
1	3.384227000	13.878331000	3.032068000
1	3.637132000	13.554371000	4.760269000
1	2.029486000	14.046238000	4.166635000
1	-3.670891000	8.957455000	4.096043000
1	-0.878097000	8.311703000	7.310655000
1	-2.527096000	6.860822000	8.453450000
1	-4.755352000	6.426321000	7.412698000
1	-5.315604000	7.494042000	5.225408000
6	6.862830000	6.473312000	3.687108000
6	6.319215000	7.649423000	3.158347000
6	6.156840000	5.741780000	4.650562000
6	5.067608000	8.088798000	3.589232000
6	4.912384000	6.187836000	5.093998000
6	4.356505000	7.368682000	4.563989000
1	6.871202000	8.218320000	2.406708000
1	6.582016000	4.820346000	5.055427000
1	4.622426000	8.999317000	3.184464000
1	4.349820000	5.618527000	5.836844000
6	3.037666000	7.850469000	4.979089000
1	7.839595000	6.122460000	3.345094000
6	1.561542000	6.012660000	9.232445000
6	1.474813000	4.782294000	8.248397000
8	1.633174000	7.130669000	8.314514000
8	2.189863000	5.277702000	7.083902000
5	2.102865000	6.650544000	7.116867000
6	2.847930000	6.027501000	10.060386000
1	2.940815000	7.006220000	10.553191000
1	2.839933000	5.247598000	10.835350000
1	3.733183000	5.880135000	9.424275000
6	0.347448000	6.198301000	10.131576000
1	-0.566882000	6.347816000	9.544435000

1	0.209331000	5.324328000	10.786153000
1	0.491202000	7.084147000	10.767575000
6	2.158283000	3.516071000	8.742393000
1	1.692829000	3.164251000	9.675639000
1	2.054050000	2.721681000	7.988451000
1	3.228818000	3.677273000	8.922297000
6	0.044561000	4.480839000	7.796643000
1	-0.462255000	5.386651000	7.433564000
1	0.077695000	3.755159000	6.971544000
1	-0.553621000	4.050389000	8.612606000
1	-0.016571000	9.032621000	5.284376000

^{1}TS3

26	0.971124000	9.401856000	4.714583000
15	1.199114000	11.517248000	5.504858000
15	0.440848000	7.751275000	3.234496000
7	3.261483000	8.891710000	5.409976000
6	-2.060564000	8.553797000	5.558272000
6	-1.324559000	9.859512000	3.285863000
6	-0.733316000	8.895040000	2.413274000
6	-0.480813000	12.079114000	5.948740000
6	1.922675000	7.526271000	2.101687000
1	2.631933000	7.008986000	2.769588000
6	-2.054956000	10.902044000	2.668845000
1	-2.501786000	11.673095000	3.300504000
6	-2.814323000	11.513589000	6.027006000
1	-3.630172000	10.812594000	5.830500000
6	-1.502264000	11.149103000	5.657427000
6	-2.244691000	10.964088000	1.290991000
1	-2.832188000	11.777632000	0.856456000
5	-1.218563000	9.741831000	4.897158000
6	2.207276000	11.891577000	7.037456000
1	1.883723000	12.900587000	7.343305000
6	0.702499000	4.966755000	3.696117000
1	0.197080000	4.062209000	4.070099000
1	1.449581000	5.258039000	4.443351000
1	1.233644000	4.688737000	2.774678000
6	-1.318168000	5.688093000	2.335038000
1	-0.805784000	5.517267000	1.376419000
1	-2.077597000	6.465351000	2.175885000
1	-1.841542000	4.755304000	2.603462000
6	-0.339498000	6.063906000	3.450083000
1	-0.916049000	6.197640000	4.376508000
6	-2.059044000	8.375539000	6.961066000
6	-0.932441000	8.957111000	1.020832000
1	-0.491676000	8.202451000	0.370466000
6	-2.883048000	7.674967000	4.825881000
6	1.862286000	12.704619000	4.212747000
1	2.890651000	12.335019000	4.053412000
6	-0.756712000	13.302498000	6.585228000
1	0.050311000	13.995529000	6.830943000
6	-1.690906000	9.980801000	0.458674000
1	-1.844489000	10.019394000	-0.622595000
6	1.780163000	6.694501000	0.823013000
1	1.159165000	7.196084000	0.067148000
1	1.356037000	5.699051000	1.002774000
1	2.777393000	6.550100000	0.374036000
6	-3.100077000	12.733361000	6.642297000
1	-4.130374000	12.982011000	6.912323000
6	-3.559501000	6.477239000	6.823237000
6	2.533168000	8.899236000	1.791538000
1	3.495197000	8.772419000	1.271458000
1	2.731479000	9.473226000	2.709474000
1	1.872448000	9.498803000	1.146956000
6	3.717416000	11.920875000	6.785023000
1	4.244578000	12.125166000	7.731765000
1	4.004169000	12.710412000	6.075127000
1	4.078400000	10.962528000	6.391289000
6	-2.067187000	13.634965000	6.927974000
1	-2.281730000	14.585261000	7.423341000
6	1.831127000	10.918623000	8.159901000
1	2.215407000	9.909337000	7.953946000
1	0.740992000	10.850629000	8.295262000
1	2.271060000	11.254710000	9.112779000
6	1.085326000	12.548387000	2.901339000
1	0.027000000	12.818087000	3.033442000
1	1.112932000	11.521813000	2.510327000
1	1.509989000	13.211775000	2.130211000
6	-3.614144000	6.650180000	5.437377000
6	-2.781773000	7.358692000	7.586250000
6	1.916735000	14.173534000	4.638267000
1	2.444661000	14.765645000	3.872360000
1	2.440447000	14.324865000	5.593168000
1	0.902826000	14.589928000	4.734605000
1	-2.957166000	7.799131000	3.743138000
1	-1.458509000	9.054550000	7.573594000
1	-2.740814000	7.249282000	8.673898000
1	-4.126851000	5.677565000	7.306444000
1	-4.234481000	5.986726000	4.827576000
6	1.691916000	4.397152000	7.393858000
6	0.602741000	5.271653000	7.327000000
6	2.944796000	4.807490000	6.921461000
6	0.766282000	6.545906000	6.785706000
6	3.109876000	6.085232000	6.387086000
6	2.016675000	6.964646000	6.308905000
1	-0.381039000	4.964255000	7.689022000
1	3.797261000	4.125829000	6.968981000
1	-0.083899000	7.221411000	6.714154000
1	4.081258000	6.393717000	6.003224000
6	2.154140000	8.309172000	5.693390000
1	1.567500000	3.395523000	7.812474000
6	6.663167000	9.017157000	4.195715000
6	6.179598000	7.624131000	3.639908000
8	5.677972000	9.293257000	5.218184000
8	4.753480000	7.692732000	3.861396000
5	4.527471000	8.611338000	4.874592000
6	6.552959000	10.137323000	3.157294000
1	6.685073000	11.104203000	3.664397000
1	7.321034000	10.046280000	2.375658000
1	5.562758000	10.136014000	2.677878000
6	8.048524000	9.011780000	4.826015000
1	8.100957000	8.328591000	5.683476000
1	8.809824000	8.713276000	4.089112000
1	8.296706000	10.022654000	5.182460000
6	6.438283000	7.398200000	2.156448000
1	7.517414000	7.418651000	1.940187000
1	6.049745000	6.412340000	1.859794000
1	5.941992000	8.157747000	1.539366000
6	6.708062000	6.442536000	4.457087000
1	6.552699000	6.603676000	5.533981000
1	6.160831000	5.534545000	4.163811000
1	7.780293000	6.272938000	4.282060000
1	1.076293000	9.031024000	6.202590000

^{1}IM3

26	0.769837000	8.833610000	5.089188000
15	1.105395000	11.021468000	5.347489000
15	0.641634000	8.249299000	2.851948000
7	2.510485000	8.181159000	5.327137000
6	-1.498845000	8.646622000	5.425740000
6	-1.786220000	9.550038000	2.874237000
6	-0.922602000	8.790222000	2.050139000
6	-0.468384000	11.936923000	5.558674000
6	2.013107000	8.338672000	1.563773000

1	2.846400000	7.940002000	2.154758000
6	-2.981267000	10.023023000	2.296855000
1	-3.670165000	10.621535000	2.893833000
6	-2.833426000	11.933302000	5.106428000
1	-3.736047000	11.441400000	4.737835000
6	-1.588885000	11.280752000	5.010245000
6	-3.322485000	9.745192000	0.972160000
1	-4.258497000	10.129409000	0.558436000
5	-1.443778000	9.813659000	4.404921000
6	2.117574000	11.602725000	6.816718000
1	2.106722000	12.698502000	6.718560000
6	1.814417000	5.642295000	3.081798000
1	1.686013000	4.621203000	3.475907000
1	2.553977000	6.154612000	3.710332000
1	2.224576000	5.549309000	2.068034000
6	-0.580508000	5.658073000	2.240130000
1	-0.291135000	5.611410000	1.180162000
1	-1.564082000	6.143033000	2.290753000
1	-0.696918000	4.620742000	2.595824000
6	0.464495000	6.368793000	3.103984000
1	0.089941000	6.321737000	4.138269000
6	-1.116663000	8.852269000	6.785212000
6	-1.274725000	8.496582000	0.722214000
1	-0.620707000	7.890402000	0.100786000
6	-1.979443000	7.339228000	5.088678000
6	1.890422000	11.955582000	3.912498000
1	2.699876000	11.281855000	3.590660000
6	-0.587595000	13.200530000	6.159236000
1	0.285416000	13.700237000	6.583192000
6	-2.468298000	8.974057000	0.179765000
1	-2.726453000	8.747694000	-0.857602000
6	1.854775000	7.483767000	0.295387000
1	1.289911000	8.022540000	-0.478590000
1	1.367517000	6.517045000	0.458867000
1	2.854822000	7.282628000	-0.124032000
6	-2.956861000	13.190444000	5.701911000
1	-3.938094000	13.667807000	5.766878000
6	-1.706830000	6.576202000	7.373638000
6	2.403700000	9.755487000	1.154189000
1	3.255906000	9.704989000	0.455925000
1	2.720137000	10.351038000	2.012273000
1	1.580981000	10.269448000	0.634974000
6	3.582944000	11.161421000	6.733350000
1	4.187613000	11.729219000	7.459009000
1	4.007455000	11.336505000	5.735482000
1	3.714849000	10.096567000	6.959713000
6	-1.830431000	13.831516000	6.227545000
1	-1.921229000	14.814344000	6.696301000
6	1.462173000	11.268340000	8.161356000
1	1.331557000	10.187821000	8.299684000
1	0.472700000	11.738108000	8.255035000
1	2.090737000	11.642600000	8.986021000
6	0.867007000	12.118379000	2.783748000
1	0.093860000	12.846133000	3.070388000
1	0.357882000	11.181577000	2.531018000
1	1.364875000	12.490369000	1.875402000
6	-2.082001000	6.336002000	6.029867000
6	-1.249643000	7.825373000	7.746257000
6	2.488967000	13.322689000	4.256446000
1	2.873802000	13.789561000	3.334833000
1	3.324588000	13.266146000	4.966053000
1	1.726143000	14.001376000	4.668553000
1	-2.304251000	7.152565000	4.063971000
1	-0.893403000	9.859605000	7.133487000
1	-0.986883000	8.024151000	8.785609000
1	-1.783280000	5.776811000	8.112878000
1	-2.465303000	5.353458000	5.742925000
6	1.026360000	4.476564000	8.684853000
6	1.224810000	5.675203000	9.379775000
6	1.160709000	4.470162000	7.292555000
6	1.544347000	6.843478000	8.689260000
6	1.484923000	5.640820000	6.605845000
6	1.675632000	6.856462000	7.285851000
1	1.132857000	5.697531000	10.469042000
1	1.015917000	3.541343000	6.733951000
1	1.700647000	7.773568000	9.242868000
1	1.604170000	5.619859000	5.527026000
6	1.997035000	8.122572000	6.605996000
1	0.775797000	3.559032000	9.222638000
6	6.129751000	8.289730000	4.904702000
6	5.587586000	8.656419000	3.463537000
8	4.935668000	7.865029000	5.589107000
8	4.208772000	8.983357000	3.724350000
5	3.832831000	8.350574000	4.903144000
6	6.696736000	9.495680000	5.658137000
1	6.839268000	9.217058000	6.712530000
1	7.667443000	9.810255000	5.247751000
1	6.010604000	10.351295000	5.623992000
6	7.139095000	7.146877000	4.932689000
1	6.704549000	6.215826000	4.547158000
1	8.031207000	7.395063000	4.337345000
1	7.459817000	6.965580000	5.969493000
6	6.260972000	9.858153000	2.812647000
1	7.336142000	9.673861000	2.665989000
1	5.807962000	10.044581000	1.827334000
1	6.139696000	10.765378000	3.418116000
6	5.602578000	7.465011000	2.500179000
1	5.091181000	6.594726000	2.936456000
1	5.073954000	7.743042000	1.576747000
1	6.626671000	7.169779000	2.230847000
1	2.331692000	8.880198000	7.321438000

Structures for cycle 2:

^{3}IM3’

26	-0.504758000	10.457124000	3.201824000
15	0.831593000	12.481843000	3.790076000
15	-2.258791000	11.353912000	1.816621000
6	-2.237755000	9.936923000	5.144798000
6	-2.756936000	12.473564000	4.296390000
6	-3.203340000	12.449537000	2.947324000
6	0.608719000	12.412402000	5.607499000
6	-1.463992000	12.529439000	0.574560000
1	-1.180186000	13.365869000	1.226543000
6	-3.340778000	13.435402000	5.145185000
1	-3.001598000	13.498859000	6.182674000
6	-0.934851000	11.860308000	7.371075000
1	-1.894407000	11.453223000	7.701243000
6	-0.652424000	11.912390000	5.992322000
6	-4.350080000	14.295099000	4.706323000
1	-4.783957000	15.024609000	5.395504000
5	-1.720613000	11.411488000	4.904837000
6	2.684587000	12.625756000	3.509426000
1	2.983437000	13.588917000	3.959741000
6	-4.550592000	9.666646000	1.603763000
1	-5.283701000	9.199930000	0.925669000
1	-5.097223000	10.336754000	2.281918000
1	-4.094483000	8.868634000	2.204824000
6	-2.774764000	9.461948000	-0.178946000
1	-2.152240000	8.743035000	0.374604000
1	-2.125226000	9.995091000	-0.886745000
1	-3.509390000	8.892068000	-0.770802000
6	-3.505090000	10.414799000	0.777485000
1	-4.031621000	11.178237000	0.181944000

6	-1.426009000	8.974926000	5.796119000
6	-4.239712000	13.292285000	2.514268000
1	-4.599117000	13.239247000	1.484382000
6	-3.537405000	9.520974000	4.776241000
6	0.115109000	14.207299000	3.416527000
1	-0.944039000	13.973496000	3.233834000
6	1.538413000	12.859763000	6.557687000
1	2.503795000	13.263680000	6.247922000
6	-4.817152000	14.213607000	3.390134000
1	-5.618014000	14.871709000	3.044344000
6	-0.175413000	11.921610000	0.007800000
1	-0.380397000	11.091751000	-0.683651000
1	0.472981000	11.529211000	0.802612000
1	0.394167000	12.681861000	-0.552007000
6	-0.011038000	12.294876000	8.324203000
1	-0.254568000	12.237032000	9.388415000
6	-3.153627000	7.289831000	5.632612000
6	-2.361640000	13.085745000	-0.530758000
1	-1.806927000	13.841522000	-1.111415000
1	-3.260978000	13.575391000	-0.132388000
1	-2.678904000	12.303506000	-1.237122000
6	3.038578000	12.627222000	2.016359000
1	4.134196000	12.664699000	1.901786000
1	2.621932000	13.475002000	1.464654000
1	2.684074000	11.697293000	1.554537000
6	1.230498000	12.796436000	7.917631000
1	1.958294000	13.135478000	8.658990000
6	3.457122000	11.484829000	4.186143000
1	3.252542000	10.537150000	3.672402000
1	3.215307000	11.366216000	5.249831000
1	4.538413000	11.682847000	4.105631000
6	0.172838000	15.163715000	4.610788000
1	1.208767000	15.350963000	4.936168000
1	-0.389929000	14.784375000	5.472373000
1	-0.264969000	16.132589000	4.318529000
6	-3.993085000	8.225938000	5.017453000
6	-1.868021000	7.672087000	6.023418000
6	0.681029000	14.895553000	2.170905000
1	0.060231000	15.774608000	1.933361000
1	0.692403000	14.251636000	1.283124000
1	1.705909000	15.257256000	2.343384000
1	-4.206561000	10.244495000	4.311404000
1	-0.435959000	9.259303000	6.149671000
1	-1.204132000	6.949194000	6.501352000
1	-3.497340000	6.267228000	5.801405000
1	-5.007553000	7.941400000	4.725063000
6	2.714709000	7.669111000	7.064880000
6	3.679300000	7.418339000	5.841191000
8	1.926504000	8.802990000	6.605327000
8	2.908939000	7.956168000	4.729686000
5	1.992349000	8.822382000	5.246563000
6	3.410687000	8.055162000	8.361621000
1	2.658482000	8.223833000	9.146234000
1	4.080916000	7.249139000	8.696827000
1	3.995489000	8.976955000	8.248647000
6	1.733316000	6.520043000	7.299715000
1	1.226151000	6.229479000	6.368395000
1	2.240226000	5.636231000	7.712629000
1	0.969015000	6.848873000	8.018713000
6	4.970493000	8.234646000	5.910271000
1	5.645392000	7.856078000	6.691166000
1	5.486461000	8.165030000	4.941515000
1	4.763051000	9.295765000	6.109611000
6	3.989427000	5.954992000	5.562209000
1	3.078549000	5.382143000	5.351586000
1	4.652317000	5.875000000	4.688246000
1	4.501347000	5.499321000	6.423374000
7	-0.146242000	8.646916000	2.470320000
6	-0.549997000	3.636408000	3.948679000
6	0.541286000	4.518520000	3.929671000
6	-1.809756000	4.108158000	3.540412000
6	0.392127000	5.836606000	3.511166000
6	-1.969586000	5.422352000	3.122394000
6	-0.872887000	6.331083000	3.084871000
1	1.525474000	4.166778000	4.252532000
1	-2.672558000	3.436069000	3.553074000
1	1.246824000	6.509173000	3.535188000
1	-2.956145000	5.781639000	2.818049000
6	-1.093327000	7.681500000	2.682247000
1	-0.423670000	2.602502000	4.277974000
6	2.688805000	7.362451000	0.565040000
6	3.007771000	8.903712000	0.658949000
8	1.302106000	7.318762000	0.944403000
8	2.084076000	9.343765000	1.680409000
5	1.040359000	8.421577000	1.737723000
6	3.475171000	6.534671000	1.587131000
1	3.041362000	5.525144000	1.630563000
1	4.535476000	6.446924000	1.306919000
1	3.406946000	6.979644000	2.588609000
6	2.837269000	6.759506000	-0.826314000
1	2.160125000	7.234489000	-1.547979000
1	3.871436000	6.861139000	-1.190029000
1	2.592771000	5.687442000	-0.787663000
6	4.432618000	9.222797000	1.095536000
1	5.157528000	8.785027000	0.392102000
1	4.589575000	10.310011000	1.112850000
1	4.637296000	8.833449000	2.100263000
6	2.664211000	9.653946000	-0.630758000
1	1.642970000	9.418696000	-0.962775000
1	2.723155000	10.736400000	-0.449955000
1	3.362842000	9.403351000	-1.442102000
1	-2.140815000	8.001597000	2.645346000
1	1.315052000	9.531712000	4.576030000

^{3}TS4

26	-0.017711000	10.157751000	3.206135000
15	0.887091000	12.371071000	4.065030000
15	-1.848345000	11.015488000	1.961500000
6	-2.565253000	9.633922000	5.671066000
6	-2.492804000	12.019233000	4.449928000
6	-2.792955000	12.117216000	3.073984000
6	0.498283000	12.276654000	5.871838000
6	-1.215312000	12.139754000	0.580870000
1	-1.045516000	13.085170000	1.114941000
6	-3.097750000	12.969079000	5.300876000
1	-2.875535000	12.937693000	6.369914000
6	-0.873194000	11.397470000	7.655593000
1	-1.723339000	10.796815000	7.988353000
6	-0.622250000	11.507346000	6.276923000
6	-3.971288000	13.947052000	4.824094000
1	-4.413767000	14.667370000	5.517187000
5	-1.646135000	10.868839000	5.218377000
6	2.726198000	12.784554000	3.921209000
1	2.818559000	13.848267000	4.201514000
6	-4.031206000	9.227521000	2.110011000
1	-4.773014000	8.615726000	1.571128000
1	-4.573563000	9.928064000	2.757855000
1	-3.456833000	8.555378000	2.763783000
6	-2.513839000	9.004230000	0.086039000
1	-1.887497000	8.247562000	0.577678000
1	-1.887819000	9.524367000	-0.651693000
1	-3.310148000	8.478500000	-0.465744000
6	-3.141892000	9.962298000	1.105724000
1	-3.776744000	10.683672000	0.565449000

6	-2.041283000	8.484135000	6.299283000
6	-3.689228000	13.086911000	2.592703000
1	-3.919274000	13.140792000	1.528014000
6	-3.962040000	9.654014000	5.480288000
6	0.071145000	14.030670000	3.594856000
1	-0.959802000	13.732915000	3.367223000
6	1.284781000	12.934537000	6.827435000
1	2.125693000	13.555087000	6.516197000
6	-4.279885000	14.003737000	3.461387000
1	-4.966310000	14.762062000	3.077199000
6	0.138000000	11.635691000	0.066742000
1	0.045926000	10.655646000	-0.425177000
1	0.868124000	11.525395000	0.878996000
1	0.555533000	12.342345000	-0.669338000
6	-0.073035000	12.034866000	8.607709000
1	-0.295793000	11.926078000	9.672524000
6	-4.227661000	7.455528000	6.463589000
6	-2.169096000	12.429891000	-0.583089000
1	-1.718889000	13.194213000	-1.237652000
1	-3.144590000	12.815699000	-0.257759000
1	-2.348505000	11.538813000	-1.201224000
6	3.168263000	12.595424000	2.461845000
1	4.232454000	12.866087000	2.358903000
1	2.604887000	13.200337000	1.744010000
1	3.057256000	11.539457000	2.180139000
6	1.006765000	12.816102000	8.191553000
1	1.633912000	13.332177000	8.922915000
6	3.669482000	11.957232000	4.800251000
1	3.697340000	10.917455000	4.455245000
1	3.383489000	11.938874000	5.856474000
1	4.686109000	12.377232000	4.718448000
6	0.009306000	15.043746000	4.740069000
1	1.013680000	15.331122000	5.091096000
1	-0.554654000	14.659334000	5.598566000
1	-0.491713000	15.960926000	4.387650000
6	-4.782850000	8.589710000	5.866379000
6	-2.846378000	7.411404000	6.683464000
6	0.655751000	14.694997000	2.343941000
1	0.002296000	15.529712000	2.041864000
1	0.733950000	14.013130000	1.488013000
1	1.654529000	15.116186000	2.534551000
1	-4.424844000	10.526566000	5.016684000
1	-0.969584000	8.404553000	6.467065000
1	-2.391627000	6.532955000	7.149179000
1	-4.863001000	6.616014000	6.757639000
1	-5.861304000	8.646116000	5.694606000
6	2.476335000	8.640449000	6.434851000
6	2.003295000	7.336432000	5.693087000
8	1.946886000	9.677343000	5.567512000
8	0.798899000	7.792110000	5.030962000
5	0.903430000	9.149431000	4.849029000
6	3.986447000	8.801543000	6.542095000
1	4.224665000	9.742692000	7.058612000
1	4.418534000	7.973018000	7.124123000
1	4.466432000	8.823937000	5.555817000
6	1.827892000	8.832608000	7.806257000
1	0.738068000	8.705746000	7.758484000
1	2.233173000	8.125197000	8.544550000
1	2.023088000	9.857183000	8.152075000
6	2.978405000	6.891755000	4.601326000
1	3.901785000	6.475107000	5.029066000
1	2.493437000	6.117822000	3.992459000
1	3.240118000	7.732078000	3.942048000
6	1.641614000	6.167028000	6.596913000
1	0.836876000	6.431114000	7.295324000
1	1.295716000	5.323079000	5.981866000
1	2.517559000	5.836653000	7.176306000
7	0.469073000	8.567208000	2.141539000
6	-0.390116000	3.448709000	3.213709000
6	0.688667000	4.045982000	2.547228000
6	-1.483571000	4.244940000	3.589119000
6	0.691724000	5.410154000	2.268113000
6	-1.491401000	5.605892000	3.309078000
6	-0.398412000	6.233512000	2.650222000
1	1.542583000	3.435742000	2.239714000
1	-2.332414000	3.796687000	4.112343000
1	1.525846000	5.850195000	1.730062000
1	-2.336945000	6.220427000	3.629413000
6	-0.476487000	7.653227000	2.451344000
1	-0.382390000	2.378627000	3.433568000
6	3.315827000	7.686223000	0.007651000
6	3.684786000	9.128952000	0.531101000
8	1.916586000	7.585384000	0.355056000
8	2.740669000	9.309859000	1.611601000
5	1.669116000	8.457796000	1.396816000
6	4.059029000	6.573937000	0.752964000
1	3.623597000	5.602951000	0.475373000
1	5.126740000	6.558039000	0.491353000
1	3.968137000	6.690189000	1.841622000
6	3.460872000	7.491433000	-1.495898000
1	2.813106000	8.177074000	-2.056418000
1	4.503176000	7.650856000	-1.811866000
1	3.176533000	6.462598000	-1.762048000
6	5.094682000	9.267885000	1.090789000
1	5.845642000	9.037806000	0.319695000
1	5.257518000	10.301701000	1.429318000
1	5.254484000	8.601743000	1.948185000
6	3.419972000	10.216801000	-0.512715000
1	2.409973000	10.126021000	-0.934899000
1	3.504094000	11.204327000	-0.039500000
1	4.147717000	10.165456000	-1.335099000
1	-1.436173000	8.080682000	2.770667000
1	-0.322221000	9.830959000	4.791103000

^{3}IM4

26	0.453232000	10.438301000	3.236840000
15	1.018449000	12.434463000	4.205275000
15	-1.387175000	11.158754000	1.883570000
6	-2.482748000	9.649654000	5.819835000
6	-2.547370000	11.937856000	4.349053000
6	-2.554927000	12.124697000	2.942962000
6	0.450120000	12.390919000	5.958116000
6	-0.942266000	12.322915000	0.458713000
1	-0.956429000	13.310559000	0.944003000
6	-3.418994000	12.755728000	5.098410000
1	-3.425373000	12.644448000	6.186140000
6	-1.137893000	11.703484000	7.625359000
1	-2.030825000	11.142398000	7.911337000
6	-0.719319000	11.658119000	6.280140000
6	-4.259793000	13.703060000	4.516504000
1	-4.912627000	14.317963000	5.142303000
5	-1.632216000	10.878869000	5.180592000
6	2.841551000	12.921672000	4.223985000
1	2.864856000	14.001319000	4.448435000
6	-3.367899000	9.151886000	2.122373000
1	-4.023914000	8.421807000	1.620285000
1	-3.997699000	9.832309000	2.708586000
1	-2.738760000	8.602155000	2.833516000
6	-1.811343000	8.959274000	0.128131000
1	-1.212252000	8.228032000	0.682750000
1	-1.135603000	9.480991000	-0.563222000
1	-2.544071000	8.396579000	-0.473093000
6	-2.542054000	9.910963000	1.080895000
1	-3.242307000	10.522097000	0.489069000

6	-1.812467000	8.680609000	6.598936000
6	-3.412595000	13.074053000	2.352745000
1	-3.419333000	13.215187000	1.272450000
6	-3.855697000	9.435716000	5.605479000
6	0.187298000	14.049935000	3.608753000
1	-0.789128000	13.681450000	3.271495000
6	1.119808000	13.144220000	6.938099000
1	1.986531000	13.749701000	6.672603000
6	-4.260440000	13.863643000	3.127517000
1	-4.912427000	14.599552000	2.650102000
6	0.487923000	12.046535000	-0.020465000
1	0.557172000	11.079574000	-0.535852000
1	1.204225000	12.008460000	0.811170000
1	0.812568000	12.834244000	-0.720673000
6	-0.457570000	12.426475000	8.607163000
1	-0.819738000	12.425274000	9.639227000
6	-3.834675000	7.373603000	6.884465000
6	-1.889635000	12.371904000	-0.745832000
1	-1.569948000	13.176438000	-1.428678000
1	-2.936140000	12.567805000	-0.476926000
1	-1.864670000	11.433142000	-1.318262000
6	3.433622000	12.676346000	2.828936000
1	4.499278000	12.958422000	2.814333000
1	2.929975000	13.238536000	2.034814000
1	3.364765000	11.606541000	2.584718000
6	0.680070000	13.159726000	8.261853000
1	1.218127000	13.745308000	9.011726000
6	3.714438000	12.183783000	5.245130000
1	3.827868000	11.131283000	4.959469000
1	3.322001000	12.222381000	6.266913000
1	4.720255000	12.635837000	5.251592000
6	-0.079443000	15.078656000	4.709975000
1	0.852406000	15.445167000	5.170579000
1	-0.715935000	14.673120000	5.505777000
1	-0.595631000	15.950275000	4.273067000
6	-4.525399000	8.317875000	6.120987000
6	-2.468005000	7.565431000	7.125914000
6	0.876087000	14.725476000	2.419785000
1	0.229395000	15.531675000	2.035869000
1	1.070040000	14.037673000	1.589123000
1	1.832200000	15.187853000	2.710380000
1	-4.422983000	10.162849000	5.018827000
1	-0.743288000	8.810236000	6.772974000
1	-1.910519000	6.838012000	7.724233000
1	-4.353630000	6.500863000	7.290049000
1	-5.594055000	8.186422000	5.926657000
6	2.370491000	8.361670000	6.398402000
6	3.389839000	8.053596000	5.229841000
8	1.349393000	9.130801000	5.728018000
8	3.062547000	9.069815000	4.239683000
5	1.806552000	9.595035000	4.515056000
6	2.941010000	9.244481000	7.509593000
1	2.115854000	9.567972000	8.160744000
1	3.677874000	8.702566000	8.120670000
1	3.415685000	10.144616000	7.102006000
6	1.713552000	7.127764000	7.011678000
1	1.138655000	6.563172000	6.266814000
1	2.467186000	6.460847000	7.457392000
1	1.021254000	7.438773000	7.807926000
6	4.858454000	8.205495000	5.608069000
1	5.133500000	7.488981000	6.397184000
1	5.488344000	8.001418000	4.728976000
1	5.087584000	9.218228000	5.962938000
6	3.160076000	6.690343000	4.578888000
1	2.116018000	6.560969000	4.267588000
1	3.780762000	6.609059000	3.677530000
1	3.428568000	5.872834000	5.263594000
7	0.288240000	8.513834000	2.704985000
6	-1.658676000	3.633550000	2.765173000
6	-1.031940000	4.312266000	1.711756000
6	-1.886354000	4.291010000	3.978239000
6	-0.617732000	5.631443000	1.871727000
6	-1.482760000	5.615256000	4.138187000
6	-0.821035000	6.299899000	3.095905000
1	-0.872181000	3.808439000	0.755669000
1	-2.390569000	3.774681000	4.798098000
1	-0.142308000	6.145256000	1.041401000
1	-1.683600000	6.141751000	5.073569000
6	-0.468017000	7.691372000	3.363774000
1	-1.980855000	2.597959000	2.633174000
6	2.827995000	7.169950000	0.385785000
6	2.857683000	8.726586000	0.120844000
8	2.025495000	7.079048000	1.592645000
8	1.628556000	9.165777000	0.761680000
5	1.288024000	8.220563000	1.686458000
6	4.187418000	6.542441000	0.656249000
1	4.064555000	5.467883000	0.856666000
1	4.845788000	6.655357000	-0.218198000
1	4.676493000	6.998673000	1.525447000
6	2.087071000	6.388368000	-0.700583000
1	1.107882000	6.837822000	-0.922072000
1	2.666999000	6.347583000	-1.633405000
1	1.921949000	5.359053000	-0.350767000
6	4.007112000	9.435106000	0.835960000
1	4.978116000	9.162866000	0.398491000
1	3.875587000	10.519942000	0.723132000
1	4.010514000	9.202691000	1.910356000
6	2.812480000	9.124249000	-1.347494000
1	1.897080000	8.768968000	-1.837242000
1	2.842626000	10.219906000	-1.434789000
1	3.681705000	8.715868000	-1.884834000
1	-0.957117000	8.102296000	4.254339000
1	-0.890056000	10.271563000	4.382120000

^{3}TS5

26	0.205328000	10.184252000	3.179177000
15	0.666864000	12.356607000	4.153514000
15	-1.714681000	10.688928000	1.741946000
6	-2.738375000	9.322429000	5.713863000
6	-2.805860000	11.564754000	4.196511000
6	-2.878836000	11.665966000	2.782411000
6	0.039310000	12.276195000	5.878269000
6	-1.437715000	11.710692000	0.183835000
1	-1.461542000	12.744456000	0.561730000
6	-3.634336000	12.439763000	4.931173000
1	-3.587291000	12.403343000	6.022446000
6	-1.458988000	11.376022000	7.526657000
1	-2.271803000	10.699829000	7.800758000
6	-1.033402000	11.396627000	6.183594000
6	-4.494107000	13.357954000	4.330773000
1	-5.108990000	14.019067000	4.947527000
5	-1.881137000	10.548869000	5.074772000
6	2.428337000	13.026403000	4.106732000
1	2.320538000	14.123168000	4.143875000
6	-3.266196000	8.440778000	2.361670000
1	-3.821377000	7.556498000	2.009927000
1	-3.943450000	9.057760000	2.964765000
1	-2.463896000	8.089566000	3.023277000
6	-1.851743000	8.326040000	0.258616000
1	-0.926941000	8.025795000	0.765744000
1	-1.576956000	8.824940000	-0.681115000
1	-2.393146000	7.401300000	0.002996000
6	-2.709504000	9.212502000	1.165314000
1	-3.557429000	9.618140000	0.588690000

6	-2.059986000	8.240534000	6.314953000
6	-3.754377000	12.587954000	2.174620000
1	-3.804802000	12.668000000	1.089670000
6	-4.141534000	9.239915000	5.682597000
6	-0.310317000	13.812397000	3.435590000
1	-1.297502000	13.369735000	3.261141000
6	0.581050000	13.120999000	6.862831000
1	1.356187000	13.841894000	6.603991000
6	-4.559868000	13.431880000	2.936670000
1	-5.226830000	14.144249000	2.444840000
6	-0.030560000	11.435012000	-0.361124000
1	0.042783000	10.416599000	-0.770114000
1	0.740664000	11.520623000	0.416945000
1	0.213248000	12.139764000	-1.173134000
6	-0.891863000	12.182143000	8.515712000
1	-1.256204000	12.123610000	9.545138000
6	-4.136052000	7.078976000	6.785175000
6	-2.481101000	11.581514000	-0.932340000
1	-2.279744000	12.331395000	-1.715021000
1	-3.511958000	11.733750000	-0.585578000
1	-2.438839000	10.593328000	-1.412220000
6	3.040635000	12.618660000	2.757561000
1	4.048643000	13.053685000	2.657351000
1	2.450304000	12.949145000	1.894425000
1	3.127702000	11.525815000	2.696494000
6	0.127555000	13.075458000	8.180773000
1	0.565928000	13.734483000	8.934205000
6	3.376937000	12.585864000	5.223946000
1	3.544816000	11.505599000	5.167988000
1	3.019225000	12.834220000	6.228839000
1	4.347307000	13.089266000	5.080367000
6	-0.490971000	14.976028000	4.411942000
1	0.472392000	15.415631000	4.718921000
1	-1.033101000	14.672297000	5.316163000
1	-1.072918000	15.773469000	3.920663000
6	-4.834954000	8.141388000	6.207731000
6	-2.737877000	7.136759000	6.838553000
6	0.236881000	14.309237000	2.094952000
1	-0.496276000	14.988755000	1.631147000
1	0.428474000	13.493313000	1.387348000
1	1.172360000	14.875859000	2.220925000
1	-4.716976000	10.050020000	5.227414000
1	-0.970978000	8.276474000	6.364074000
1	-2.173253000	6.315485000	7.290601000
1	-4.672475000	6.216934000	7.190903000
1	-5.927593000	8.114614000	6.159408000
6	3.377791000	8.605283000	5.746772000
6	2.112282000	8.695076000	6.678458000
8	2.995395000	9.461955000	4.628283000
8	1.056993000	8.922564000	5.718178000
5	1.603625000	9.419736000	4.553028000
6	3.599130000	7.191826000	5.205309000
1	4.372184000	7.216531000	4.429179000
1	3.930863000	6.511374000	6.002653000
1	2.684715000	6.783982000	4.754561000
6	4.664499000	9.139493000	6.359119000
1	4.568592000	10.186601000	6.669873000
1	4.946584000	8.540601000	7.238690000
1	5.481533000	9.071024000	5.625062000
6	1.790632000	7.419051000	7.448398000
1	2.622684000	7.143885000	8.114777000
1	0.897107000	7.581734000	8.069189000
1	1.585625000	6.579786000	6.772021000
6	2.136111000	9.888526000	7.631950000
1	2.379523000	10.816176000	7.103002000
1	1.137092000	10.015524000	8.070670000
1	2.862366000	9.738672000	8.444577000
7	1.093223000	8.394822000	3.029786000
6	-1.231610000	3.975172000	1.295356000
6	-0.210712000	4.745860000	0.722118000
6	-1.738447000	4.339573000	2.554006000
6	0.297201000	5.862928000	1.377369000
6	-1.238594000	5.450003000	3.218994000
6	-0.209278000	6.256262000	2.647623000
1	0.196321000	4.468107000	-0.253874000
1	-2.535180000	3.748983000	3.013533000
1	1.101085000	6.430687000	0.914686000
1	-1.648515000	5.736151000	4.191274000
6	0.201644000	7.402522000	3.386130000
1	-1.625410000	3.100990000	0.772103000
6	3.527407000	9.201145000	0.441045000
6	4.141464000	7.878271000	1.043643000
8	2.521193000	9.541161000	1.427346000
8	3.032313000	7.345425000	1.816486000
5	2.195244000	8.388094000	2.107172000
6	2.800435000	8.973104000	-0.885511000
1	2.267188000	9.891645000	-1.164897000
1	3.505004000	8.725529000	-1.691978000
1	2.061572000	8.162066000	-0.805060000
6	4.504865000	10.360855000	0.310056000
1	4.944905000	10.634654000	1.276105000
1	5.315630000	10.105233000	-0.388885000
1	3.978007000	11.242005000	-0.084545000
6	4.557250000	6.836115000	0.015489000
1	5.346075000	7.231389000	-0.642524000
1	4.954756000	5.949954000	0.531840000
1	3.709566000	6.515731000	-0.603369000
6	5.284902000	8.156816000	2.019562000
1	4.977429000	8.886824000	2.781044000
1	5.558810000	7.221001000	2.527164000
1	6.174329000	8.534138000	1.495260000
1	-0.260593000	7.540657000	4.364173000
1	-1.075246000	9.911595000	4.348126000

^{3}IM5

26	-0.176117000	10.011026000	2.860490000
15	0.509897000	12.225166000	3.469994000
15	-2.200663000	10.522527000	1.906480000
6	-2.565318000	9.581522000	6.064148000
6	-2.697372000	11.699630000	4.415643000
6	-3.068287000	11.681948000	3.048106000
6	0.451934000	12.211298000	5.322684000
6	-2.133663000	11.446845000	0.260837000
1	-2.050363000	12.492564000	0.592458000
6	-3.234884000	12.743901000	5.197861000
1	-2.920410000	12.829382000	6.240835000
6	-0.600478000	11.543123000	7.382547000
1	-1.371500000	10.979283000	7.913105000
6	-0.562946000	11.463807000	5.977098000
6	-4.121406000	13.693499000	4.690661000
1	-4.502247000	14.489280000	5.336683000
5	-1.710759000	10.638480000	5.163541000
6	2.276787000	12.699500000	3.018410000
1	2.407082000	13.754256000	3.316737000
6	-3.846309000	8.529939000	2.988899000
1	-4.639298000	7.778310000	2.841818000
1	-4.193154000	9.254082000	3.737740000
1	-2.970143000	8.019568000	3.412492000
6	-3.112890000	8.141268000	0.611664000
1	-2.338069000	7.471660000	1.001141000
1	-2.732844000	8.572764000	-0.323900000
1	-3.992517000	7.525195000	0.361701000
6	-3.500800000	9.191734000	1.654522000
1	-4.386687000	9.744926000	1.298968000

6	-2.203932000	8.221090000	6.100723000
6	-3.981180000	12.624656000	2.538933000
1	-4.270876000	12.601698000	1.488970000
6	-3.699313000	9.938220000	6.820137000
6	-0.466130000	13.805976000	3.077734000
1	-1.499853000	13.458907000	3.170567000
6	1.349968000	12.998189000	6.064097000
1	2.104728000	13.599868000	5.556522000
6	-4.512631000	13.626408000	3.350793000
1	-5.211197000	14.356660000	2.934706000
6	-0.844157000	11.092541000	-0.488771000
1	-0.876963000	10.062465000	-0.874825000
1	0.034895000	11.175357000	0.167492000
1	-0.700078000	11.768257000	-1.347902000
6	0.304690000	12.309690000	8.120643000
1	0.240240000	12.334434000	9.212055000
6	-4.036192000	7.661339000	7.585421000
6	-3.346613000	11.344011000	-0.671129000
1	-3.231153000	12.059089000	-1.502388000
1	-4.299129000	11.569659000	-0.173058000
1	-3.436770000	10.342384000	-1.114264000
6	2.496058000	12.579225000	1.502090000
1	3.534980000	12.857223000	1.259160000
1	1.837112000	13.224414000	0.913403000
1	2.350051000	11.540548000	1.176512000
6	1.287903000	13.046844000	7.457470000
1	2.000161000	13.659061000	8.016287000
6	3.318771000	11.840070000	3.736949000
1	3.201173000	10.788821000	3.457720000
1	3.249382000	11.886136000	4.828330000
1	4.330742000	12.159705000	3.436959000
6	-0.270463000	14.940808000	4.083893000
1	0.775958000	15.285514000	4.124710000
1	-0.574450000	14.648134000	5.096410000
1	-0.888525000	15.802809000	3.781531000
6	-4.422489000	9.004467000	7.571518000
6	-2.917623000	7.274009000	6.841144000
6	-0.278550000	14.322923000	1.648412000
1	-1.091146000	15.028333000	1.409871000
1	-0.302677000	13.522874000	0.897717000
1	0.670703000	14.868202000	1.534052000
1	-4.043238000	10.974679000	6.817436000
1	-1.328037000	7.892583000	5.541005000
1	-2.599045000	6.227154000	6.834863000
1	-4.601742000	6.926258000	8.164466000
1	-5.297356000	9.327634000	8.143527000
6	2.947778000	8.571417000	6.558703000
6	1.707665000	7.609299000	6.833317000
8	2.603825000	9.195047000	5.298004000
8	1.081575000	7.504978000	5.521258000
5	1.627302000	8.450675000	4.711324000
6	4.262697000	7.824333000	6.331570000
1	5.030934000	8.548041000	6.023315000
1	4.606441000	7.325438000	7.248611000
1	4.164284000	7.078082000	5.531450000
6	3.135589000	9.673414000	7.592820000
1	2.251023000	10.317209000	7.663078000
1	3.347844000	9.247168000	8.584645000
1	3.989604000	10.302945000	7.301374000
6	2.085713000	6.199421000	7.272353000
1	2.620166000	6.222698000	8.233736000
1	1.170594000	5.603852000	7.404996000
1	2.716563000	5.694544000	6.529826000
6	0.687268000	8.199547000	7.798923000
1	0.344858000	9.185611000	7.466971000
1	-0.190024000	7.542973000	7.854632000
1	1.115043000	8.294847000	8.807075000
7	1.309478000	8.606668000	3.290749000
6	-0.133219000	5.532520000	-0.698876000
6	0.281662000	6.862635000	-0.822125000
6	-0.448122000	5.028535000	0.566675000
6	0.369305000	7.681807000	0.303158000
6	-0.350113000	5.849847000	1.692102000
6	0.049883000	7.195080000	1.583041000
1	0.527865000	7.269814000	-1.806134000
1	-0.772344000	3.990804000	0.678332000
1	0.671022000	8.720886000	0.188791000
1	-0.603063000	5.453009000	2.678569000
6	0.032193000	8.055277000	2.798681000
1	-0.213781000	4.895067000	-1.582593000
6	4.044580000	8.782803000	0.785245000
6	4.675374000	7.935837000	1.970590000
8	2.796581000	9.258544000	1.368012000
8	3.515618000	7.625710000	2.786285000
5	2.522453000	8.503916000	2.466862000
6	3.688102000	7.941813000	-0.437927000
1	3.094067000	8.552990000	-1.132491000
1	4.594949000	7.608015000	-0.961689000
1	3.089242000	7.062959000	-0.168766000
6	4.869650000	9.991759000	0.365776000
1	5.018633000	10.691825000	1.195893000
1	5.853467000	9.671945000	-0.008802000
1	4.353908000	10.528518000	-0.443677000
6	5.325080000	6.627396000	1.541342000
1	6.167586000	6.817073000	0.859556000
1	5.714136000	6.104943000	2.427557000
1	4.608954000	5.963346000	1.041864000
6	5.636869000	8.738084000	2.847451000
1	5.174169000	9.666248000	3.207078000
1	5.907987000	8.134050000	3.724191000
1	6.557404000	8.989714000	2.302324000
1	-0.483140000	7.542090000	3.616238000
1	-1.119329000	9.867363000	4.366560000

^{3}TS6

26	-0.064641000	10.135888000	3.238459000
15	0.563947000	12.294176000	3.855063000
15	-1.752559000	10.554821000	1.552461000
6	-2.473141000	9.137855000	5.540605000
6	-2.683449000	11.384106000	4.088397000
6	-2.864787000	11.496403000	2.686553000
6	0.164753000	12.188172000	5.648068000
6	-1.268621000	11.819170000	0.224897000
1	-1.255059000	12.760843000	0.792483000
6	-3.498761000	12.209185000	4.894284000
1	-3.378957000	12.157479000	5.979216000
6	-1.174853000	11.202374000	7.379154000
1	-1.949035000	10.504938000	7.703843000
6	-0.829176000	11.245901000	6.011774000
6	-4.446717000	13.085459000	4.369327000
1	-5.049192000	13.706307000	5.038237000
5	-1.666756000	10.392764000	4.898227000
6	2.373813000	12.809233000	3.774361000
1	2.435645000	13.879215000	4.036954000
6	-3.841229000	8.660163000	1.609481000
1	-4.490794000	7.964132000	1.052707000
1	-4.482488000	9.331609000	2.195846000
1	-3.242337000	8.065754000	2.310739000
6	-2.214384000	8.527443000	-0.334780000
1	-1.554772000	7.838278000	0.207155000
1	-1.597380000	9.089654000	-1.051700000
1	-2.929908000	7.921663000	-0.915480000
6	-2.960643000	9.443897000	0.637284000
1	-3.613884000	10.117824000	0.059598000

6	-1.856911000	8.258188000	6.460436000
6	-3.836479000	12.367347000	2.156306000
1	-3.978717000	12.434242000	1.077634000
6	-3.796605000	8.822971000	5.175641000
6	-0.391775000	13.838660000	3.293448000
1	-1.345666000	13.406907000	2.963046000
6	0.762918000	13.026620000	6.602317000
1	1.509161000	13.762637000	6.297168000
6	-4.625434000	13.163010000	2.984346000
1	-5.369686000	13.837391000	2.553237000
6	0.159243000	11.525004000	-0.243896000
1	0.197104000	10.601096000	-0.842738000
1	0.839466000	11.384090000	0.603971000
1	0.542788000	12.345834000	-0.873444000
6	-0.571044000	12.021933000	8.334522000
1	-0.869690000	11.947494000	9.383970000
6	-3.821449000	6.843047000	6.576836000
6	-2.195289000	12.012550000	-0.979155000
1	-1.816050000	12.839526000	-1.602852000
1	-3.227233000	12.264583000	-0.700591000
1	-2.230021000	11.117419000	-1.617800000
6	2.898278000	12.591713000	2.350307000
1	3.969539000	12.845892000	2.294794000
1	2.374430000	13.184751000	1.592924000
1	2.783697000	11.533419000	2.079637000
6	0.409861000	12.940146000	7.948951000
1	0.889648000	13.587959000	8.686920000
6	3.235621000	11.993002000	4.739890000
1	3.117503000	10.916453000	4.560987000
1	2.996824000	12.182324000	5.792787000
1	4.300268000	12.234152000	4.585584000
6	-0.706798000	14.831595000	4.413357000
1	0.205964000	15.273391000	4.844622000
1	-1.277932000	14.364606000	5.225378000
1	-1.312641000	15.657177000	4.003738000
6	-4.462299000	7.698707000	5.676293000
6	-2.510475000	7.134466000	6.971344000
6	0.242435000	14.561156000	2.101682000
1	-0.449360000	15.339296000	1.739769000
1	0.451022000	13.891205000	1.258009000
1	1.181703000	15.063236000	2.380560000
1	-4.322257000	9.474773000	4.476479000
1	-0.827764000	8.449172000	6.770493000
1	-1.990873000	6.476445000	7.673783000
1	-4.336095000	5.961620000	6.968106000
1	-5.488318000	7.489785000	5.359903000
6	3.858558000	8.294620000	6.375520000
6	2.482346000	8.238134000	7.145959000
8	3.448706000	8.706147000	5.047000000
8	1.546429000	7.959612000	6.069908000
5	2.135112000	8.351076000	4.897899000
6	4.522198000	6.924528000	6.225132000
1	5.347931000	7.008214000	5.503826000
1	4.928688000	6.563351000	7.180720000
1	3.811229000	6.178467000	5.841480000
6	4.852596000	9.310377000	6.920095000
1	4.446435000	10.328738000	6.897010000
1	5.125971000	9.063396000	7.957150000
1	5.768947000	9.293239000	6.311433000
6	2.373440000	7.126340000	8.179732000
1	3.137189000	7.250616000	8.962481000
1	1.384084000	7.165852000	8.658870000
1	2.492306000	6.134948000	7.724511000
6	2.078812000	9.577678000	7.757628000
1	2.125556000	10.389528000	7.020679000
1	1.040779000	9.518509000	8.109485000
1	2.719322000	9.838446000	8.612229000
7	1.428367000	8.404833000	3.637719000
6	-1.740822000	4.887707000	1.439420000
6	-0.573092000	5.502651000	0.982396000
6	-2.327872000	5.345759000	2.623976000
6	-0.013082000	6.567990000	1.691146000
6	-1.756330000	6.398715000	3.337084000
6	-0.588540000	7.040532000	2.878339000
1	-0.094356000	5.160463000	0.061146000
1	-3.236955000	4.873776000	3.005038000
1	0.881191000	7.036818000	1.295957000
1	-2.217488000	6.728908000	4.266879000
6	-0.023404000	8.139514000	3.719146000
1	-2.184205000	4.057638000	0.884577000
6	3.028154000	8.862810000	0.327740000
6	4.162646000	8.235222000	1.226763000
8	2.010173000	9.191334000	1.323306000
8	3.407215000	7.704046000	2.341295000
5	2.249004000	8.421732000	2.436019000
6	2.414741000	7.867064000	-0.657920000
1	1.468655000	8.277983000	-1.038211000
1	3.088250000	7.695553000	-1.509377000
1	2.204621000	6.895681000	-0.192454000
6	3.445469000	10.123456000	-0.416302000
1	3.736337000	10.928486000	0.266457000
1	4.297319000	9.900419000	-1.076228000
1	2.618373000	10.485396000	-1.041302000
6	4.940453000	7.103725000	0.571009000
1	5.450288000	7.460396000	-0.336682000
1	5.704999000	6.729562000	1.267662000
1	4.286583000	6.264673000	0.301988000
6	5.121228000	9.278093000	1.806319000
1	4.572872000	10.104742000	2.277949000
1	5.733719000	8.797896000	2.582385000
1	5.789385000	9.689545000	1.036545000
1	-0.205879000	7.833321000	4.753691000
1	-0.903465000	9.216292000	4.158948000

^{3}Pr

26	-0.345367000	10.504471000	3.756207000
15	0.651394000	12.531271000	4.458679000
15	-1.904077000	10.900271000	2.015948000
6	-2.209037000	9.919780000	5.397456000
6	-3.044657000	12.200881000	4.188286000
6	-3.231046000	11.904114000	2.810806000
6	-0.022183000	12.816369000	6.143887000
6	-1.119195000	12.139024000	0.824233000
1	-1.027963000	13.027036000	1.462749000
6	-3.927014000	13.144376000	4.757985000
1	-3.794945000	13.421923000	5.806889000
6	-1.943038000	12.426340000	7.544118000
1	-2.923892000	11.969601000	7.706753000
6	-1.302436000	12.250474000	6.301844000
6	-4.976960000	13.714271000	4.035495000
1	-5.642560000	14.436678000	4.516587000
5	-1.959827000	11.443062000	5.080797000
6	2.515287000	12.784765000	4.595091000
1	2.703023000	13.867192000	4.704755000
6	-3.815853000	8.831532000	1.564246000
1	-4.356333000	8.209319000	0.831389000
1	-4.563449000	9.379753000	2.153986000
1	-3.281840000	8.156666000	2.248918000
6	-1.941204000	9.007281000	-0.116246000
1	-1.181362000	8.426734000	0.421706000
1	-1.403762000	9.670677000	-0.806725000
1	-2.537152000	8.311202000	-0.730131000
6	-2.864129000	9.779456000	0.831838000
1	-3.481943000	10.460777000	0.224828000

6	-1.386025000	9.218046000	6.318431000
6	-4.314913000	12.441020000	2.097583000
1	-4.481567000	12.165119000	1.054397000
6	-3.286913000	9.177907000	4.842455000
6	0.059779000	14.129163000	3.601003000
1	-0.940415000	13.836095000	3.247050000
6	0.567133000	13.580725000	7.164596000
1	1.534045000	14.064155000	7.014625000
6	-5.192150000	13.343709000	2.703708000
1	-6.025932000	13.764748000	2.136206000
6	0.289530000	11.666861000	0.465241000
1	0.268011000	10.726762000	-0.097026000
1	0.885434000	11.488289000	1.371790000
1	0.816006000	12.418402000	-0.146002000
6	-1.352018000	13.161956000	8.574237000
1	-1.869967000	13.283040000	9.529870000
6	-2.690615000	7.184897000	6.085874000
6	-1.914245000	12.547299000	-0.416071000
1	-1.379297000	13.353287000	-0.946563000
1	-2.911274000	12.930996000	-0.161686000
1	-2.034802000	11.716841000	-1.127731000
6	3.194378000	12.267558000	3.318426000
1	4.281830000	12.445250000	3.369167000
1	2.824606000	12.747991000	2.404499000
1	3.041954000	11.182718000	3.217601000
6	-0.095375000	13.749801000	8.381428000
1	0.366212000	14.339042000	9.178125000
6	3.096580000	12.033818000	5.795109000
1	2.820463000	10.974722000	5.735283000
1	2.762182000	12.425633000	6.762068000
1	4.197861000	12.083304000	5.769866000
6	-0.121113000	15.321255000	4.543759000
1	0.824131000	15.601136000	5.036309000
1	-0.864308000	15.123821000	5.325848000
1	-0.462016000	16.195116000	3.963133000
6	-3.525429000	7.848331000	5.171643000
6	-1.623649000	7.881804000	6.657208000
6	0.911679000	14.548805000	2.399615000
1	0.400302000	15.354690000	1.847914000
1	1.096433000	13.731776000	1.694557000
1	1.887535000	14.943285000	2.722923000
1	-3.967297000	9.686891000	4.161591000
1	-0.564320000	9.749112000	6.801984000
1	-0.963473000	7.374662000	7.362671000
1	-2.874443000	6.140191000	6.348079000
1	-4.374422000	7.321588000	4.726461000
6	3.581391000	8.093897000	5.905084000
6	2.356070000	7.543234000	6.723256000
8	2.915752000	8.892201000	4.891412000
8	1.332424000	7.463758000	5.696454000
5	1.692189000	8.312052000	4.683243000
6	4.351904000	6.999738000	5.159381000
1	5.027786000	7.477911000	4.436629000
1	4.947946000	6.382496000	5.846466000
1	3.674891000	6.340908000	4.596596000
6	4.550358000	8.956907000	6.698923000
1	4.055769000	9.802403000	7.188008000
1	5.047251000	8.348883000	7.469926000
1	5.323808000	9.356242000	6.026643000
6	2.550262000	6.155719000	7.317105000
1	3.378076000	6.158040000	8.042439000
1	1.635090000	5.847979000	7.844115000
1	2.763782000	5.407511000	6.543006000
6	1.876610000	8.523781000	7.793520000
1	1.741864000	9.533887000	7.382146000
1	0.911608000	8.185323000	8.193429000
1	2.590546000	8.578967000	8.627372000
7	0.903565000	8.462304000	3.471351000
6	0.235287000	3.642445000	1.736273000
6	-0.777384000	4.477425000	1.256383000
6	1.067776000	4.095373000	2.764416000
6	-0.943021000	5.757541000	1.791120000
6	0.892044000	5.372414000	3.302686000
6	-0.109054000	6.226277000	2.815281000
1	-1.436526000	4.135585000	0.454523000
1	1.856020000	3.447580000	3.156360000
1	-1.724425000	6.409959000	1.397830000
1	1.527697000	5.705821000	4.121794000
6	-0.324624000	7.612848000	3.383111000
1	0.372721000	2.643695000	1.315019000
6	2.133036000	8.045739000	0.067588000
6	3.388782000	8.720208000	0.752749000
8	1.066905000	8.363822000	1.001885000
8	2.998483000	8.758950000	2.147059000
5	1.646549000	8.575257000	2.223481000
6	2.223875000	6.519676000	0.008866000
1	1.247725000	6.115553000	-0.291546000
1	2.979630000	6.190782000	-0.718478000
1	2.466466000	6.090759000	0.990299000
6	1.782440000	8.594236000	-1.308146000
1	1.605200000	9.675313000	-1.292105000
1	2.597594000	8.386028000	-2.017469000
1	0.871865000	8.102277000	-1.680023000
6	4.676649000	7.911953000	0.646294000
1	4.962996000	7.773261000	-0.407181000
1	5.490289000	8.450562000	1.154214000
1	4.575913000	6.926018000	1.117008000
6	3.639116000	10.157936000	0.300096000
1	2.730492000	10.766167000	0.371895000
1	4.400792000	10.608987000	0.951739000
1	4.004889000	10.191688000	-0.736138000
1	-0.743967000	7.533606000	4.390991000
1	-1.063323000	8.139441000	2.769069000

[1]IM3'

26	-0.196701000	10.435190000	3.211686000
15	0.758026000	12.564980000	3.832251000
15	-1.802382000	11.241569000	1.878184000
6	-2.210988000	9.728928000	5.214444000
6	-2.504446000	12.234347000	4.345115000
6	-2.852752000	12.276188000	2.973296000
6	0.665769000	12.370046000	5.649389000
6	-1.031896000	12.451777000	0.655339000
1	-0.532538000	13.141424000	1.344043000
6	-3.178133000	13.116470000	5.210124000
1	-2.916407000	13.116053000	6.272172000
6	-0.638430000	11.423010000	7.434222000
1	-1.499632000	10.846938000	7.782047000
6	-0.465900000	11.630481000	6.053519000
6	-4.183033000	13.972141000	4.753282000
1	-4.689421000	14.644562000	5.451104000
5	-1.503281000	11.123682000	4.940328000
6	2.584294000	12.767010000	3.416445000
1	2.861527000	13.765279000	3.795814000
6	-4.177026000	9.666843000	1.558476000
1	-4.871675000	9.200736000	0.840346000
1	-4.733895000	10.426067000	2.123911000
1	-3.856182000	8.888908000	2.263221000
6	-2.285808000	9.185754000	-0.036133000
1	-1.901300000	8.381716000	0.604045000
1	-1.443439000	9.576530000	-0.619713000
1	-3.004699000	8.743698000	-0.745433000
6	-2.992607000	10.264115000	0.796002000
1	-3.395892000	11.023240000	0.105135000

6	-1.541782000	8.673858000	5.874943000
6	-3.892752000	13.103684000	2.518601000
1	-4.193930000	13.088162000	1.470121000
6	-3.562253000	9.498598000	4.877144000
6	0.000847000	14.301916000	3.555763000
1	-1.022409000	14.070006000	3.229215000
6	1.578880000	12.888556000	6.575667000
1	2.442258000	13.470039000	6.244362000
6	-4.558379000	13.952650000	3.405183000
1	-5.363009000	14.598483000	3.045120000
6	0.058544000	11.765103000	-0.170812000
1	-0.366167000	11.213443000	-1.021926000
1	0.643741000	11.060384000	0.431550000
1	0.750589000	12.515509000	-0.585620000
6	0.274212000	11.923939000	8.366538000
1	0.120273000	11.741123000	9.433658000
6	-3.505902000	7.261725000	5.804688000
6	-1.961620000	13.280101000	-0.230720000
1	-1.356162000	13.938542000	-0.876105000
1	-2.632885000	13.922735000	0.352780000
1	-2.571846000	12.650331000	-0.897148000
6	2.809100000	12.706962000	1.900069000
1	3.880521000	12.839533000	1.677450000
1	2.259868000	13.472955000	1.342733000
1	2.504656000	11.721825000	1.524273000
6	1.387215000	12.657722000	7.940136000
1	2.102585000	13.048989000	8.667878000
6	3.486166000	11.719921000	4.082323000
1	3.369566000	10.744740000	3.592523000
1	3.291447000	11.600129000	5.155335000
1	4.539216000	12.021238000	3.961265000
6	-0.113173000	15.107728000	4.853326000
1	0.875209000	15.344592000	5.277215000
1	-0.692528000	14.578165000	5.618416000
1	-0.623857000	16.060711000	4.638180000
6	-4.201934000	8.290198000	5.162528000
6	-2.169611000	7.462856000	6.162160000
6	0.668145000	15.155958000	2.473696000
1	0.110214000	16.100954000	2.374887000
1	0.670825000	14.680353000	1.484673000
1	1.705700000	15.415990000	2.732152000
1	-4.132369000	10.291787000	4.393436000
1	-0.505622000	8.819489000	6.171118000
1	-1.612273000	6.661799000	6.653229000
1	-3.996986000	6.309595000	6.019052000
1	-5.250908000	8.151780000	4.885714000
6	2.436931000	8.066145000	6.770852000
6	3.421494000	7.723787000	5.585991000
8	1.703957000	9.207315000	6.236036000
8	2.700847000	8.245020000	4.425628000
5	1.824713000	9.173046000	4.889975000
6	3.111868000	8.503554000	8.062294000
1	2.343468000	8.743645000	8.811438000
1	3.743273000	7.696052000	8.462560000
1	3.730131000	9.397926000	7.914393000
6	1.417733000	6.960708000	7.043184000
1	0.913878000	6.636550000	6.121856000
1	1.898189000	6.085916000	7.503646000
1	0.655654000	7.339577000	7.738702000
6	4.741797000	8.490542000	5.654428000
1	5.379488000	8.114083000	6.466732000
1	5.278890000	8.361142000	4.703656000
1	4.576466000	9.565448000	5.809801000
6	3.681900000	6.238252000	5.389009000
1	2.757661000	5.687159000	5.183464000
1	4.369656000	6.086772000	4.544815000
1	4.151295000	5.814609000	6.289724000
7	-0.176352000	8.575079000	2.576135000
6	-0.600474000	3.555061000	4.012167000
6	0.516659000	4.394161000	3.874148000
6	-1.879487000	4.085834000	3.767283000
6	0.372472000	5.723127000	3.492007000
6	-2.034722000	5.412089000	3.388531000
6	-0.912776000	6.277569000	3.226431000
1	1.517548000	3.999259000	4.073510000
1	-2.762363000	3.449807000	3.878897000
1	1.246574000	6.364262000	3.417550000
1	-3.034469000	5.816145000	3.213440000
6	-1.126915000	7.641245000	2.876035000
1	-0.479180000	2.510887000	4.309443000
6	2.564710000	7.278140000	0.542618000
6	2.909600000	8.821761000	0.632037000
8	1.190435000	7.236489000	0.965339000
8	1.953909000	9.302128000	1.604425000
5	0.943955000	8.346865000	1.750860000
6	3.376877000	6.431798000	1.527574000
1	2.947681000	5.420309000	1.563147000
1	4.430638000	6.354019000	1.222002000
1	3.330054000	6.860197000	2.535580000
6	2.658923000	6.677530000	-0.855291000
1	1.972325000	7.169187000	-1.556264000
1	3.683789000	6.753775000	-1.249661000
1	2.389233000	5.611927000	-0.809368000
6	4.313459000	9.125006000	1.145628000
1	5.075164000	8.673886000	0.491630000
1	4.477872000	10.212041000	1.160226000
1	4.449504000	8.748070000	2.166192000
6	2.667016000	9.555852000	-0.687472000
1	1.655094000	9.365448000	-1.069655000
1	2.777626000	10.637646000	-0.532312000
1	3.393820000	9.246516000	-1.452253000
1	-2.156004000	7.996996000	2.970775000
1	1.312362000	9.996335000	4.184136000

^{1}TS4

26	0.174846000	10.346837000	3.351460000
15	0.876694000	12.397328000	4.211334000
15	-1.543205000	11.027230000	1.969600000
6	-2.476518000	9.575855000	5.613405000
6	-2.492545000	11.976801000	4.364899000
6	-2.616648000	12.125039000	2.967212000
6	0.350296000	12.407239000	5.978594000
6	-1.043429000	12.055893000	0.459675000
1	-0.944380000	13.065826000	0.883938000
6	-3.228085000	12.878147000	5.162997000
1	-3.139948000	12.813081000	6.250042000
6	-1.132102000	11.501923000	7.652540000
1	-1.966179000	10.850362000	7.927209000
6	-0.749343000	11.575397000	6.302497000
6	-4.049195000	13.864784000	4.613628000
1	-4.592167000	14.550681000	5.269666000
5	-1.609640000	10.853693000	5.143415000
6	2.721888000	12.836173000	4.095415000
1	2.739818000	13.937101000	4.062815000
6	-3.679180000	9.184142000	2.305133000
1	-4.402148000	8.497184000	1.834316000
1	-4.242404000	9.926230000	2.885004000
1	-3.081823000	8.606980000	3.022088000
6	-2.212560000	8.806753000	0.273423000
1	-1.714858000	7.993576000	0.814122000
1	-1.469323000	9.233905000	-0.411949000
1	-3.014266000	8.359317000	-0.337332000
6	-2.813801000	9.842185000	1.230150000
1	-3.471775000	10.506348000	0.646777000

6	-1.873083000	8.360675000	6.002467000
6	-3.453716000	13.104240000	2.407386000
1	-3.536818000	13.204202000	1.324779000
6	-3.882494000	9.622016000	5.698982000
6	0.130521000	14.025062000	3.562358000
1	-0.898955000	13.732857000	3.326005000
6	0.981199000	13.171167000	6.969661000
1	1.796045000	13.847718000	6.710501000
6	-4.169510000	13.979093000	3.225328000
1	-4.806545000	14.749156000	2.783062000
6	0.341176000	11.598645000	-0.013122000
1	0.315229000	10.566534000	-0.392846000
1	1.072840000	11.628832000	0.806421000
1	0.716054000	12.246807000	-0.822759000
6	-0.481303000	12.236855000	8.647676000
1	-0.801729000	12.153548000	9.690016000
6	-4.012729000	7.334922000	6.496336000
6	-2.013665000	12.136487000	-0.723211000
1	-1.635706000	12.866197000	-1.458735000
1	-3.023409000	12.458136000	-0.434108000
1	-2.107821000	11.171580000	-1.242117000
6	3.235364000	12.286512000	2.757151000
1	4.298455000	12.545393000	2.617452000
1	2.686784000	12.673217000	1.889189000
1	3.147126000	11.189347000	2.747089000
6	0.572659000	13.087253000	8.303847000
1	1.078289000	13.682679000	9.068554000
6	3.685395000	12.392424000	5.200325000
1	3.898149000	11.322760000	5.119961000
1	3.307205000	12.569101000	6.212302000
1	4.631116000	12.948848000	5.082104000
6	0.059947000	15.152333000	4.594117000
1	1.059734000	15.443552000	4.956963000
1	-0.549426000	14.874846000	5.463316000
1	-0.393509000	16.045838000	4.132403000
6	-4.640994000	8.528791000	6.131673000
6	-2.617114000	7.258696000	6.431558000
6	0.787266000	14.534235000	2.274102000
1	0.162374000	15.327978000	1.832762000
1	0.910993000	13.752845000	1.515162000
1	1.778686000	14.972805000	2.466338000
1	-4.404291000	10.537837000	5.412762000
1	-0.790090000	8.259087000	5.936973000
1	-2.103249000	6.333592000	6.708748000
1	-4.601937000	6.475384000	6.826901000
1	-5.730961000	8.608881000	6.178349000
6	2.530352000	9.031923000	6.709984000
6	2.385077000	7.706075000	5.879186000
8	2.117102000	10.024035000	5.747991000
8	1.360875000	8.055082000	4.934734000
5	1.306304000	9.448740000	4.781294000
6	3.947267000	9.341144000	7.178924000
1	3.956919000	10.297057000	7.722984000
1	4.306748000	8.556085000	7.862328000
1	4.650042000	9.421061000	6.340101000
6	1.566567000	9.116278000	7.895890000
1	0.536201000	8.887559000	7.594709000
1	1.860033000	8.429873000	8.704291000
1	1.570206000	10.143558000	8.285721000
6	3.642132000	7.361933000	5.073563000
1	4.472850000	7.046752000	5.721933000
1	3.406608000	6.534966000	4.388141000
1	3.963901000	8.219006000	4.465426000
6	1.922474000	6.491586000	6.675600000
1	0.949988000	6.669054000	7.152885000
1	1.816770000	5.627998000	6.001497000
1	2.654022000	6.230972000	7.456256000
7	0.269601000	8.522084000	2.524208000
6	0.130690000	3.348974000	2.518437000
6	1.232751000	4.209711000	2.449804000
6	-1.147827000	3.869162000	2.748741000
6	1.056009000	5.584894000	2.590512000
6	-1.324667000	5.244391000	2.900205000
6	-0.228575000	6.120546000	2.794134000
1	2.235974000	3.803053000	2.301505000
1	-2.007027000	3.198357000	2.819607000
1	1.919586000	6.250004000	2.594768000
1	-2.318190000	5.654232000	3.098127000
6	-0.453667000	7.555259000	2.972257000
1	0.271460000	2.270989000	2.410482000
6	2.350585000	7.856584000	-0.443794000
6	3.395828000	8.529329000	0.556544000
8	1.084264000	8.016623000	0.246693000
8	2.641897000	8.653000000	1.792468000
5	1.331956000	8.372749000	1.539228000
6	2.560038000	6.355802000	-0.646698000
1	1.710598000	5.954174000	-1.217857000
1	3.483096000	6.154100000	-1.208246000
1	2.603944000	5.815875000	0.306554000
6	2.241830000	8.541835000	-1.801537000
1	1.979271000	9.602157000	-1.708087000
1	3.190165000	8.462524000	-2.353072000
1	1.458822000	8.047305000	-2.394769000
6	4.627406000	7.679072000	0.849426000
1	5.214283000	7.513243000	-0.065938000
1	5.265263000	8.203051000	1.576046000
1	4.359517000	6.704385000	1.275991000
6	3.828385000	9.934031000	0.141242000
1	2.965818000	10.595052000	-0.006897000
1	4.454251000	10.367569000	0.932638000
1	4.415622000	9.908668000	-0.787361000
1	-1.313053000	7.813846000	3.599725000
1	-0.203493000	9.907888000	4.768303000

[1]IM4

26	0.409572000	10.385118000	3.241089000
15	0.987373000	12.418313000	4.053793000
15	-1.335891000	11.063737000	1.856447000
6	-2.318140000	9.603659000	5.792163000
6	-2.479415000	11.844890000	4.303259000
6	-2.547693000	11.987493000	2.897824000
6	0.535919000	12.414289000	5.840261000
6	-0.937632000	12.262833000	0.439850000
1	-0.982275000	13.241978000	0.939266000
6	-3.368383000	12.630456000	5.066846000
1	-3.329902000	12.550341000	6.156385000
6	-0.995540000	11.746256000	7.568326000
1	-1.877527000	11.189213000	7.893242000
6	-0.610674000	11.677907000	6.216588000
6	-4.285208000	13.507967000	4.491148000
1	-4.949001000	14.102869000	5.124486000
5	-1.505929000	10.855649000	5.143096000
6	2.809410000	12.895658000	3.986662000
1	2.870758000	13.952372000	4.297264000
6	-3.346465000	9.049868000	1.974234000
1	-3.968153000	8.322422000	1.425934000
1	-4.015955000	9.734315000	2.510544000
1	-2.774738000	8.496144000	2.728273000
6	-1.653513000	8.862659000	0.089376000
1	-1.078462000	8.134437000	0.675398000
1	-0.949794000	9.397787000	-0.564618000
1	-2.339891000	8.290925000	-0.556491000
6	-2.448518000	9.809227000	0.995590000
1	-3.111062000	10.413045000	0.357010000

6	-1.674554000	8.727530000	6.692018000
6	-3.484400000	12.862717000	2.314150000
1	-3.540402000	12.967424000	1.231258000
6	-3.659791000	9.312421000	5.486155000
6	0.113659000	14.036114000	3.503462000
1	-0.852959000	13.655110000	3.151650000
6	1.248679000	13.169046000	6.784323000
1	2.117058000	13.756407000	6.482010000
6	-4.348981000	13.623547000	3.098815000
1	-5.062778000	14.303703000	2.627165000
6	0.496403000	12.036172000	-0.044635000
1	0.572766000	11.100402000	-0.614510000
1	1.204577000	11.960389000	0.787723000
1	0.816679000	12.857248000	-0.707534000
6	-0.281065000	12.485561000	8.514910000
1	-0.609509000	12.503451000	9.557936000
6	-3.664869000	7.364569000	6.930759000
6	-1.880006000	12.303102000	-0.768956000
1	-1.579000000	13.127402000	-1.436883000
1	-2.933881000	12.464763000	-0.506948000
1	-1.822152000	11.375102000	-1.357387000
6	3.331900000	12.754945000	2.550433000
1	4.427462000	12.876241000	2.531409000
1	2.904854000	13.484653000	1.855821000
1	3.098131000	11.751319000	2.169885000
6	0.853128000	13.200249000	8.123062000
1	1.422554000	13.785509000	8.849776000
6	3.693683000	12.052425000	4.910722000
1	3.756817000	11.019324000	4.545663000
1	3.327127000	12.023614000	5.942586000
1	4.714695000	12.469127000	4.922200000
6	-0.185306000	15.024338000	4.633903000
1	0.735087000	15.403868000	5.106037000
1	-0.807752000	14.577581000	5.418718000
1	-0.728185000	15.892079000	4.221891000
6	-4.327219000	8.211886000	6.038776000
6	-2.328665000	7.630043000	7.255860000
6	0.778739000	14.774948000	2.338570000
1	0.108429000	15.577574000	1.988445000
1	0.987792000	14.127522000	1.479469000
1	1.722051000	15.253470000	2.645036000
1	-4.204563000	9.965977000	4.801994000
1	-0.628501000	8.914793000	6.929435000
1	-1.792571000	6.973340000	7.947298000
1	-4.182127000	6.505652000	7.366753000
1	-5.370710000	8.018909000	5.773747000
6	2.375262000	8.413232000	6.490201000
6	3.163897000	7.877162000	5.231427000
8	1.369898000	9.257652000	5.895790000
8	2.823768000	8.844823000	4.212158000
5	1.672230000	9.554795000	4.579462000
6	3.213791000	9.290454000	7.421933000
1	2.539256000	9.788893000	8.133684000
1	3.945910000	8.696835000	7.989340000
1	3.749789000	10.069478000	6.866925000
6	1.677639000	7.327452000	7.306286000
1	0.925933000	6.794748000	6.710611000
1	2.406034000	6.598119000	7.692561000
1	1.164777000	7.782905000	8.166543000
6	4.680227000	7.850844000	5.391758000
1	4.977093000	7.186217000	6.217665000
1	5.143989000	7.471087000	4.468474000
1	5.083981000	8.852505000	5.587087000
6	2.673490000	6.512175000	4.742299000
1	1.583577000	6.495720000	4.619423000
1	3.119008000	6.300485000	3.760525000
1	2.955895000	5.707594000	5.436757000
7	0.220003000	8.462552000	2.827267000
6	-1.784984000	3.553381000	2.986323000
6	-0.884244000	4.082293000	2.053558000
6	-2.254335000	4.359193000	4.028376000
6	-0.445373000	5.400549000	2.156641000
6	-1.824850000	5.679908000	4.133081000
6	-0.906320000	6.224114000	3.205714000
1	-0.519195000	3.457457000	1.234842000
1	-2.961957000	3.960581000	4.758895000
1	0.265533000	5.790776000	1.434598000
1	-2.202486000	6.310866000	4.939627000
6	-0.583833000	7.633286000	3.418178000
1	-2.121932000	2.517831000	2.897125000
6	2.740523000	7.456973000	0.302419000
6	3.025891000	8.992305000	0.548501000
8	1.865932000	7.117083000	1.413295000
8	1.835818000	9.396193000	1.298778000
5	1.292565000	8.266648000	1.860009000
6	3.967618000	6.559902000	0.368792000
1	3.667904000	5.514788000	0.202476000
1	4.692070000	6.836914000	-0.411514000
1	4.460553000	6.624615000	1.346581000
6	1.945272000	7.186324000	-0.975451000
1	1.050062000	7.821670000	-1.030846000
1	2.555268000	7.360430000	-1.873059000
1	1.619574000	6.135915000	-0.979453000
6	4.253829000	9.230889000	1.422807000
1	5.168937000	8.936928000	0.890062000
1	4.329007000	10.298349000	1.661877000
1	4.177638000	8.688355000	2.370732000
6	3.119868000	9.824765000	-0.721616000
1	2.208295000	9.765234000	-1.327204000
1	3.289850000	10.878776000	-0.461207000
1	3.969788000	9.483451000	-1.331715000
1	-1.170744000	8.073262000	4.230730000
1	-0.686166000	10.197119000	4.396747000

[1]TS5

26	0.265058000	10.380152000	3.073385000
15	0.980341000	12.324864000	3.993365000
15	-1.421782000	11.159960000	1.772364000
6	-2.619890000	9.738001000	5.601903000
6	-2.434915000	12.040751000	4.231422000
6	-2.515500000	12.203241000	2.828003000
6	0.563106000	12.211495000	5.784497000
6	-1.043788000	12.259274000	0.281500000
1	-1.020535000	13.265985000	0.725664000
6	-3.155548000	12.956600000	5.023352000
1	-3.094250000	12.866196000	6.111159000
6	-0.961225000	11.477705000	7.493240000
1	-1.866283000	10.944963000	7.793907000
6	-0.618723000	11.511777000	6.128953000
6	-3.930297000	13.978126000	4.474492000
1	-4.464328000	14.674032000	5.127228000
5	-1.616725000	10.880952000	5.014392000
6	2.827506000	12.684335000	3.892987000
1	2.971529000	13.720776000	4.243775000
6	-3.387927000	9.174623000	2.174129000
1	-4.047385000	8.405018000	1.740065000
1	-4.010069000	9.879743000	2.737524000
1	-2.722627000	8.680817000	2.892158000
6	-1.857778000	8.869816000	0.184817000
1	-1.154345000	8.260679000	0.769973000
1	-1.292596000	9.358363000	-0.620855000
1	-2.572730000	8.176681000	-0.288217000
6	-2.601585000	9.873653000	1.067228000
1	-3.316928000	10.431674000	0.442371000

6	-2.112266000	8.618920000	6.294365000
6	-3.312362000	13.221496000	2.271975000
1	-3.375073000	13.347589000	1.191506000
6	-4.019803000	9.804969000	5.470985000
6	0.224145000	14.022708000	3.566961000
1	-0.820145000	13.760880000	3.362188000
6	1.336564000	12.846465000	6.768226000
1	2.224671000	13.415143000	6.488727000
6	-4.018204000	14.107818000	3.085305000
1	-4.623907000	14.899131000	2.636518000
6	0.354407000	11.943942000	-0.251099000
1	0.373628000	10.954979000	-0.729930000
1	1.105718000	11.931220000	0.547250000
1	0.661687000	12.685066000	-1.007359000
6	-0.181973000	12.092000000	8.477693000
1	-0.480799000	12.033019000	9.528082000
6	-4.331844000	7.712575000	6.656623000
6	-2.057326000	12.265448000	-0.868620000
1	-1.777417000	13.040513000	-1.601245000
1	-3.086044000	12.474088000	-0.544987000
1	-2.068253000	11.303601000	-1.402662000
6	3.304457000	12.563426000	2.438768000
1	4.394695000	12.720220000	2.386866000
1	2.834310000	13.279768000	1.757818000
1	3.088932000	11.553231000	2.069336000
6	0.976232000	12.782879000	8.115384000
1	1.593109000	13.274125000	8.872265000
6	3.671243000	11.734188000	4.742870000
1	3.559260000	10.705216000	4.380724000
1	3.400917000	11.750973000	5.804501000
1	4.735817000	12.010000000	4.661333000
6	0.223430000	15.029514000	4.718737000
1	1.243339000	15.271575000	5.060156000
1	-0.346180000	14.665144000	5.582376000
1	-0.240994000	15.970839000	4.379232000
6	-4.866011000	8.815184000	5.984759000
6	-2.944074000	7.621882000	6.811139000
6	0.795389000	14.671247000	2.303455000
1	0.143836000	15.505561000	1.995322000
1	0.859453000	13.974837000	1.459359000
1	1.798778000	15.089792000	2.477567000
1	-4.469637000	10.654800000	4.953186000
1	-1.033880000	8.531823000	6.408558000
1	-2.505474000	6.767925000	7.336011000
1	-4.988319000	6.935837000	7.058206000
1	-5.948686000	8.906549000	5.858060000
6	1.935531000	8.230624000	6.432874000
6	3.111203000	7.893853000	5.413427000
8	0.916501000	8.793909000	5.592390000
8	2.703056000	8.575305000	4.201328000
5	1.383024000	9.004514000	4.312095000
6	2.293915000	9.290875000	7.473671000
1	1.385200000	9.567619000	8.025346000
1	3.044614000	8.922519000	8.188272000
1	2.668275000	10.203272000	6.997446000
6	1.345167000	7.004348000	7.130061000
1	0.915097000	6.299363000	6.407475000
1	2.107856000	6.481642000	7.726546000
1	0.542194000	7.324040000	7.810403000
6	4.484363000	8.413203000	5.833814000
1	4.824398000	7.897668000	6.744190000
1	5.211232000	8.207993000	5.033527000
1	4.484455000	9.490140000	6.027067000
6	3.235677000	6.406529000	5.079306000
1	2.277585000	5.972357000	4.778230000
1	3.940200000	6.285950000	4.242579000
1	3.622447000	5.836967000	5.936768000
7	0.419253000	8.438261000	2.999178000
6	-0.257037000	3.227030000	3.827250000
6	0.771030000	3.879854000	3.135643000
6	-1.297866000	3.985166000	4.379362000
6	0.769661000	5.264122000	2.987624000
6	-1.306643000	5.368284000	4.240669000
6	-0.274905000	6.050900000	3.537477000
1	1.590405000	3.298601000	2.704852000
1	-2.104650000	3.492242000	4.927443000
1	1.575642000	5.742182000	2.439907000
1	-2.112575000	5.955084000	4.686934000
6	-0.412349000	7.479676000	3.470192000
1	-0.245918000	2.140199000	3.936399000
6	2.333538000	7.756317000	-0.107428000
6	3.042900000	9.011193000	0.541548000
8	1.430506000	7.322369000	0.951311000
8	2.023158000	9.459711000	1.493545000
5	1.280281000	8.350069000	1.822546000
6	3.267911000	6.600092000	-0.433353000
1	2.688610000	5.769852000	-0.863074000
1	4.021724000	6.910572000	-1.172313000
1	3.783186000	6.230905000	0.462035000
6	1.471514000	8.104530000	-1.317390000
1	0.811876000	8.953605000	-1.102165000
1	2.089532000	8.351124000	-2.192007000
1	0.841053000	7.239379000	-1.567201000
6	4.278693000	8.648090000	1.364246000
1	5.106220000	8.333510000	0.712986000
1	4.597338000	9.531138000	1.934516000
1	4.059872000	7.853128000	2.087853000
6	3.379561000	10.116409000	-0.446151000
1	2.496111000	10.494562000	-0.968060000
1	3.858196000	10.956210000	0.075038000
1	4.090007000	9.733968000	-1.194345000
1	-1.254955000	7.868982000	4.042863000
1	-0.911579000	10.177882000	4.177078000

^{1}IM5

26	-0.204202000	10.045450000	2.867625000
15	0.507454000	12.227161000	3.465313000
15	-2.181041000	10.531504000	1.908054000
6	-2.536553000	9.551965000	5.987219000
6	-2.684345000	11.706762000	4.397969000
6	-3.064872000	11.691020000	3.034900000
6	0.459658000	12.209308000	5.316076000
6	-2.090858000	11.460053000	0.266991000
1	-2.004258000	12.503933000	0.603331000
6	-3.222044000	12.740172000	5.192793000
1	-2.904040000	12.818707000	6.235375000
6	-0.599832000	11.505140000	7.361656000
1	-1.370809000	10.929010000	7.879076000
6	-0.549451000	11.445595000	5.956476000
6	-4.116683000	13.687488000	4.694266000
1	-4.498750000	14.477713000	5.346300000
5	-1.688852000	10.640897000	5.119637000
6	2.273821000	12.705743000	3.013683000
1	2.400835000	13.760316000	3.314321000
6	-3.851737000	8.541097000	2.958040000
1	-4.645350000	7.793041000	2.796460000
1	-4.205253000	9.261017000	3.707806000
1	-2.981302000	8.025913000	3.387486000
6	-3.105445000	8.164559000	0.584010000
1	-2.352002000	7.474313000	0.978652000
1	-2.700755000	8.597316000	-0.340553000
1	-3.994105000	7.570664000	0.312979000
6	-3.488290000	9.211202000	1.632811000
1	-4.366003000	9.775836000	1.274879000

6	-2.119799000	8.210326000	6.072838000
6	-3.983830000	12.630956000	2.534539000
1	-4.277702000	12.612099000	1.485408000
6	-3.723288000	9.874633000	6.674307000
6	-0.461490000	13.816116000	3.082033000
1	-1.496869000	13.470162000	3.158629000
6	1.347951000	12.993526000	6.070518000
1	2.100777000	13.606950000	5.574017000
6	-4.514156000	13.626272000	3.355875000
1	-5.217255000	14.356955000	2.948249000
6	-0.796288000	11.099367000	-0.470594000
1	-0.826296000	10.065850000	-0.846671000
1	0.082254000	11.193104000	0.184965000
1	-0.646921000	11.766434000	-1.335655000
6	0.295287000	12.270658000	8.113512000
1	0.225096000	12.282316000	9.204718000
6	-4.006979000	7.599848000	7.466521000
6	-3.293208000	11.371226000	-0.679880000
1	-3.163293000	12.089989000	-1.505920000
1	-4.249397000	11.601447000	-0.191211000
1	-3.386153000	10.372759000	-1.129493000
6	2.495718000	12.589289000	1.497624000
1	3.535681000	12.865214000	1.256873000
1	1.839725000	13.237497000	0.909105000
1	2.348008000	11.551734000	1.169910000
6	1.276739000	13.022875000	7.464320000
1	1.981193000	13.632690000	8.035555000
6	3.317979000	11.849352000	3.732451000
1	3.205392000	10.798391000	3.450470000
1	3.245230000	11.891443000	4.823733000
1	4.329396000	12.174070000	3.435927000
6	-0.275845000	14.939234000	4.102977000
1	0.770345000	15.282601000	4.158572000
1	-0.590108000	14.635052000	5.108955000
1	-0.890237000	15.805313000	3.804707000
6	-4.446557000	8.925021000	7.405253000
6	-2.833746000	7.247735000	6.792540000
6	-0.258696000	14.350788000	1.661344000
1	-1.063385000	15.066420000	1.426291000
1	-0.285075000	13.561315000	0.899591000
1	0.696187000	14.888947000	1.560624000
1	-4.109106000	10.895171000	6.631636000
1	-1.201259000	7.906279000	5.573164000
1	-2.471215000	6.215820000	6.825039000
1	-4.572469000	6.851876000	8.028802000
1	-5.363380000	9.221775000	7.923150000
6	2.968623000	8.592099000	6.571427000
6	1.743496000	7.614881000	6.852518000
8	2.595138000	9.232104000	5.327806000
8	1.105538000	7.514092000	5.546933000
5	1.621789000	8.481580000	4.740373000
6	4.285228000	7.859421000	6.309787000
1	5.041266000	8.593379000	5.995776000
1	4.650677000	7.353000000	7.214255000
1	4.177252000	7.121988000	5.502947000
6	3.166662000	9.678507000	7.620059000
1	2.278578000	10.314023000	7.716337000
1	3.400148000	9.237818000	8.600810000
1	4.010457000	10.319513000	7.323863000
6	2.140926000	6.206001000	7.277437000
1	2.685223000	6.227593000	8.233383000
1	1.233543000	5.599783000	7.415164000
1	2.769028000	5.713325000	6.524444000
6	0.727457000	8.187684000	7.833842000
1	0.371179000	9.172311000	7.511850000
1	-0.142131000	7.521224000	7.895245000
1	1.165180000	8.280705000	8.837922000
7	1.282598000	8.649183000	3.328516000
6	-0.183130000	5.484323000	-0.588523000
6	0.237408000	6.809025000	-0.746237000
6	-0.497955000	5.014666000	0.690294000
6	0.329835000	7.656275000	0.357722000
6	-0.394054000	5.864350000	1.793907000
6	0.010612000	7.205115000	1.650462000
1	0.483939000	7.189733000	-1.740770000
1	-0.826557000	3.981557000	0.829262000
1	0.635486000	8.690720000	0.215428000
1	-0.646499000	5.494380000	2.790969000
6	-0.009800000	8.100397000	2.841616000
1	-0.267831000	4.824709000	-1.455449000
6	3.990398000	8.785567000	0.789070000
6	4.630203000	7.944309000	1.974572000
8	2.749605000	9.270104000	1.379821000
8	3.481477000	7.656050000	2.812051000
5	2.485448000	8.532778000	2.494291000
6	3.621882000	7.938278000	-0.426046000
1	3.024148000	8.546896000	-1.119785000
1	4.524023000	7.599538000	-0.954760000
1	3.023402000	7.062563000	-0.147200000
6	4.817052000	9.987815000	0.352893000
1	4.981498000	10.692072000	1.176410000
1	5.794054000	9.659721000	-0.032187000
1	4.294659000	10.522875000	-0.453418000
6	5.257613000	6.623157000	1.550145000
1	6.092815000	6.794629000	0.854717000
1	5.652388000	6.105546000	2.436689000
1	4.526190000	5.962812000	1.068318000
6	5.616688000	8.743781000	2.825924000
1	5.171729000	9.680666000	3.185135000
1	5.897244000	8.144924000	3.703117000
1	6.529805000	8.979583000	2.261600000
1	-0.514647000	7.593767000	3.669218000
1	-1.094443000	9.873553000	4.294175000

[1]TS6

26	-0.045429000	10.133563000	3.019781000
15	0.592348000	12.225557000	3.690988000
15	-1.724346000	10.708488000	1.676757000
6	-2.473940000	9.210228000	5.446983000
6	-2.732184000	11.498462000	4.116148000
6	-2.931808000	11.610425000	2.722864000
6	0.303857000	12.102639000	5.511423000
6	-1.277526000	11.942453000	0.313464000
1	-1.297905000	12.900553000	0.851102000
6	-3.558406000	12.287549000	4.942186000
1	-3.422555000	12.233904000	6.025662000
6	-1.086288000	11.259842000	7.281986000
1	-1.913371000	10.635070000	7.625668000
6	-0.760032000	11.261131000	5.909579000
6	-4.539066000	13.134234000	4.425773000
1	-5.152950000	13.737549000	5.100384000
5	-1.676320000	10.483774000	4.820819000
6	2.405064000	12.753229000	3.588161000
1	2.472570000	13.769297000	4.013042000
6	-3.754040000	8.738678000	1.647238000
1	-4.258966000	7.927250000	1.097081000
1	-4.520510000	9.435745000	2.012305000
1	-3.266049000	8.294373000	2.519425000
6	-1.839767000	8.480436000	-0.018629000
1	-1.204551000	7.910072000	0.671217000
1	-1.180676000	9.008615000	-0.724505000
1	-2.439420000	7.760527000	-0.599300000
6	-2.754558000	9.448801000	0.737463000
1	-3.334837000	10.026393000	0.002458000

6	-1.855234000	8.313961000	6.347474000
6	-3.937161000	12.441306000	2.196889000
1	-4.093443000	12.505128000	1.119627000
6	-3.804484000	8.907015000	5.094587000
6	-0.366593000	13.828310000	3.277454000
1	-1.333839000	13.431817000	2.943945000
6	1.006061000	12.878861000	6.448457000
1	1.816201000	13.536607000	6.128757000
6	-4.740307000	13.205948000	3.042240000
1	-5.513458000	13.856562000	2.625733000
6	0.150491000	11.687690000	-0.164532000
1	0.205511000	10.753283000	-0.741188000
1	0.843924000	11.591307000	0.677053000
1	0.500633000	12.504513000	-0.817562000
6	-0.393048000	12.030557000	8.216511000
1	-0.677370000	11.997389000	9.271983000
6	-3.816086000	6.893137000	6.446308000
6	-2.216650000	12.058014000	-0.890675000
1	-1.888977000	12.896843000	-1.527435000
1	-3.262861000	12.247922000	-0.616587000
1	-2.190947000	11.151405000	-1.514338000
6	2.907615000	12.773403000	2.141857000
1	3.980421000	13.026556000	2.118390000
1	2.383015000	13.484183000	1.496235000
1	2.789998000	11.774201000	1.705168000
6	0.669086000	12.839346000	7.801692000
1	1.226426000	13.441165000	8.524070000
6	3.299185000	11.803657000	4.387822000
1	3.254269000	10.786334000	3.981652000
1	3.026443000	11.733554000	5.446192000
1	4.349517000	12.133133000	4.326920000
6	-0.648810000	14.731576000	4.479364000
1	0.276989000	15.123530000	4.928919000
1	-1.214017000	14.210882000	5.261674000
1	-1.248132000	15.595556000	4.145997000
6	-4.466994000	7.772949000	5.576952000
6	-2.502686000	7.176265000	6.835571000
6	0.231181000	14.652597000	2.134479000
1	-0.478892000	15.447272000	1.852007000
1	0.432061000	14.059373000	1.233938000
1	1.168087000	15.145920000	2.435611000
1	-4.341351000	9.576190000	4.421292000
1	-0.828911000	8.501348000	6.663349000
1	-1.975877000	6.504391000	7.519322000
1	-4.326910000	6.001986000	6.820298000
1	-5.498191000	7.575594000	5.270152000
6	3.723628000	8.261054000	6.529235000
6	2.319296000	8.117892000	7.236501000
8	3.357736000	8.767690000	5.221894000
8	1.429503000	7.918474000	6.104117000
5	2.054610000	8.424532000	4.999749000
6	4.425034000	6.921091000	6.302753000
1	5.282657000	7.078815000	5.632707000
1	4.792591000	6.491556000	7.245786000
1	3.750942000	6.195180000	5.825303000
6	4.669694000	9.254493000	7.188238000
1	4.236625000	10.261948000	7.220290000
1	4.908524000	8.939512000	8.215357000
1	5.609121000	9.303394000	6.617802000
6	2.189835000	6.921068000	8.167501000
1	2.916988000	6.992691000	8.990458000
1	1.180326000	6.902650000	8.604171000
1	2.348608000	5.973801000	7.636739000
6	1.867663000	9.395012000	7.939243000
1	1.914018000	10.264013000	7.269582000
1	0.822370000	9.283804000	8.254748000
1	2.476225000	9.601649000	8.831139000
7	1.402556000	8.570577000	3.714748000
6	-1.294484000	4.634234000	1.573801000
6	0.015639000	5.089442000	1.415205000
6	-2.148959000	5.321304000	2.445035000
6	0.452513000	6.236564000	2.085495000
6	-1.710082000	6.463908000	3.109553000
6	-0.411707000	6.973272000	2.907410000
1	0.713697000	4.542695000	0.775471000
1	-3.165827000	4.959031000	2.616337000
1	1.496356000	6.527264000	1.988653000
1	-2.375871000	6.970242000	3.808411000
6	-0.038818000	8.223624000	3.640246000
1	-1.638662000	3.740408000	1.048363000
6	2.803282000	8.908362000	0.316265000
6	4.037411000	8.374981000	1.154120000
8	1.853305000	9.269542000	1.375308000
8	3.430672000	7.977223000	2.407324000
5	2.227887000	8.604113000	2.517288000
6	2.130320000	7.848034000	-0.554263000
1	1.173043000	8.247370000	-0.916971000
1	2.756284000	7.602253000	-1.423613000
1	1.917235000	6.924744000	-0.005983000
6	3.144344000	10.112265000	-0.551744000
1	3.455950000	10.981924000	0.034441000
1	3.966386000	9.842424000	-1.231366000
1	2.284371000	10.399573000	-1.168086000
6	4.728593000	7.163389000	0.544329000
1	5.138493000	7.409486000	-0.446584000
1	5.561298000	6.850826000	1.191227000
1	4.041176000	6.314574000	0.438822000
6	5.062089000	9.459156000	1.491322000
1	4.580951000	10.342664000	1.930611000
1	5.766424000	9.056013000	2.232992000
1	5.630788000	9.772163000	0.604619000
1	-0.346119000	8.028852000	4.672985000
1	-0.934317000	9.336053000	3.936849000

[1]Pr

26	-0.370223000	10.539911000	3.783503000
15	0.644257000	12.508317000	4.449923000
15	-1.880567000	10.919067000	2.051792000
6	-2.190441000	9.927521000	5.378271000
6	-3.045346000	12.202816000	4.181508000
6	-3.234105000	11.912422000	2.807528000
6	-0.000803000	12.798352000	6.142169000
6	-1.091525000	12.143797000	0.850935000
1	-0.990580000	13.033044000	1.485191000
6	-3.955930000	13.100894000	4.774195000
1	-3.825802000	13.371885000	5.824960000
6	-1.950278000	12.421514000	7.502835000
1	-2.938663000	11.974879000	7.647191000
6	-1.288064000	12.247803000	6.272367000
6	-5.030790000	13.641634000	4.064360000
1	-5.719603000	14.333103000	4.558141000
5	-1.892937000	11.452056000	5.008066000
6	2.511588000	12.757635000	4.555582000
1	2.695538000	13.841724000	4.653554000
6	-3.779847000	8.832704000	1.586091000
1	-4.316565000	8.213869000	0.847551000
1	-4.531182000	9.377641000	2.173681000
1	-3.250428000	8.153789000	2.270040000
6	-1.907921000	9.016710000	-0.095209000
1	-1.140015000	8.438010000	0.431817000
1	-1.381189000	9.682702000	-0.791299000
1	-2.508029000	8.319464000	-0.703835000
6	-2.825047000	9.784134000	0.861815000
1	-3.443389000	10.467877000	0.258472000

6	-1.374127000	9.231413000	6.302758000
6	-4.337830000	12.418668000	2.102987000
1	-4.503240000	12.148325000	1.058111000
6	-3.285975000	9.202501000	4.847854000
6	0.066230000	14.123344000	3.606325000
1	-0.938629000	13.848273000	3.252373000
6	0.585553000	13.545868000	7.176135000
1	1.560634000	14.018334000	7.045981000
6	-5.239148000	13.284221000	2.727719000
1	-6.089853000	13.685965000	2.171300000
6	0.311919000	11.663356000	0.483159000
1	0.282431000	10.720997000	-0.074540000
1	0.914490000	11.486849000	1.385364000
1	0.836414000	12.410281000	-0.135447000
6	-1.364924000	13.139981000	8.548594000
1	-1.896003000	13.259512000	9.497262000
6	-2.717884000	7.221609000	6.119147000
6	-1.895193000	12.554540000	-0.383035000
1	-1.360928000	13.357863000	-0.918257000
1	-2.887921000	12.943230000	-0.119113000
1	-2.026784000	11.724944000	-1.093686000
6	3.176355000	12.234257000	3.274737000
1	4.263237000	12.419981000	3.307874000
1	2.790305000	12.705142000	2.362684000
1	3.031248000	11.147944000	3.184603000
6	-0.096474000	13.711843000	8.382907000
1	0.358809000	14.286631000	9.193703000
6	3.112418000	12.023930000	5.755991000
1	2.830095000	10.966100000	5.719274000
1	2.799420000	12.434047000	6.722354000
1	4.213374000	12.067169000	5.708983000
6	-0.097963000	15.305836000	4.564620000
1	0.851420000	15.567981000	5.058958000
1	-0.842100000	15.108175000	5.345661000
1	-0.428765000	16.191160000	3.995496000
6	-3.550470000	7.882778000	5.204281000
6	-1.631359000	7.904750000	6.667776000
6	0.918498000	14.552673000	2.408310000
1	0.411943000	15.370319000	1.869524000
1	1.095936000	13.745373000	1.690531000
1	1.898196000	14.935300000	2.733857000
1	-3.961602000	9.712757000	4.163427000
1	-0.540633000	9.757446000	6.770803000
1	-0.973993000	7.396686000	7.374906000
1	-2.919550000	6.185549000	6.402485000
1	-4.414167000	7.364900000	4.777834000
6	3.560262000	8.098253000	5.886908000
6	2.330492000	7.565664000	6.710042000
8	2.902556000	8.903690000	4.874363000
8	1.300821000	7.502256000	5.688486000
5	1.668036000	8.344487000	4.671819000
6	4.315150000	6.993309000	5.140891000
1	4.995303000	7.462120000	4.416026000
1	4.905308000	6.369870000	5.827508000
1	3.629445000	6.341750000	4.580432000
6	4.544228000	8.948584000	6.676577000
1	4.064416000	9.800880000	7.168614000
1	5.036556000	8.333503000	7.444914000
1	5.319745000	9.337487000	6.000606000
6	2.506423000	6.175042000	7.302465000
1	3.336590000	6.165018000	8.024993000
1	1.588737000	5.880221000	7.832484000
1	2.706676000	5.424065000	6.527518000
6	1.871533000	8.552930000	7.783283000
1	1.749612000	9.564910000	7.372524000
1	0.904090000	8.228677000	8.188953000
1	2.590644000	8.597631000	8.613271000
7	0.880420000	8.495418000	3.457964000
6	0.256659000	3.652755000	1.751425000
6	-0.755871000	4.479508000	1.257204000
6	1.077853000	4.116810000	2.783610000
6	-0.932231000	5.762537000	1.781406000
6	0.890817000	5.396585000	3.311493000
6	-0.109535000	6.242652000	2.809577000
1	-1.406362000	4.128948000	0.452045000
1	1.865714000	3.475698000	3.187154000
1	-1.713228000	6.408141000	1.376365000
1	1.516443000	5.738639000	4.134296000
6	-0.339145000	7.632341000	3.365972000
1	0.402554000	2.651831000	1.338256000
6	2.138115000	8.046973000	0.065289000
6	3.388091000	8.730730000	0.753408000
8	1.065548000	8.364360000	0.991534000
8	2.984661000	8.791683000	2.143193000
5	1.634010000	8.597973000	2.213966000
6	2.235484000	6.521169000	0.013026000
1	1.263711000	6.112109000	-0.294704000
1	2.998967000	6.192613000	-0.706358000
1	2.470802000	6.096729000	0.998105000
6	1.790856000	8.588448000	-1.314366000
1	1.607539000	9.668647000	-1.302691000
1	2.609853000	8.382078000	-2.019745000
1	0.884380000	8.090281000	-1.687978000
6	4.675664000	7.918421000	0.673790000
1	4.974932000	7.764714000	-0.373973000
1	5.483759000	8.462686000	1.184461000
1	4.567260000	6.939356000	1.156877000
6	3.648111000	10.159901000	0.279790000
1	2.742384000	10.773866000	0.334217000
1	4.407137000	10.618296000	0.929279000
1	4.022033000	10.174310000	-0.753988000
1	-0.765430000	7.555924000	4.371102000
1	-1.077670000	8.148677000	2.745628000

Raw Kinetic Data

Table S14. Raw kinetic data used to determine the order in iron (0.002475M in complex A, 0.666 in HBpin and 0.333 in Benzonitrile)

Run 1		Run 2	
Time (s)	[3d] (M)	Time (s)	[3d] (M)
60	0.00861	60	0.01026
120	0.01053	120	0.01211
180	0.01266	180	0.01379
240	0.01519	240	0.01738
300	0.0173	300	0.01936
360	0.02039	360	0.02197
420	0.02293	420	0.02454
480	0.02594	480	0.0284
540	0.02868	540	0.03171
600	0.03164	600	0.03484
660	0.03463	660	0.03841
720	0.03793	720	0.04187
780	0.04202	780	0.04541
840	0.04562	840	0.0491
900	0.04939	900	0.05317
960	0.05438	960	0.05789
1020	0.0578	1020	0.06276
1080	0.06144	1080	0.0672
1140	0.06615	1140	0.0719
1200	0.0701	1200	0.07616

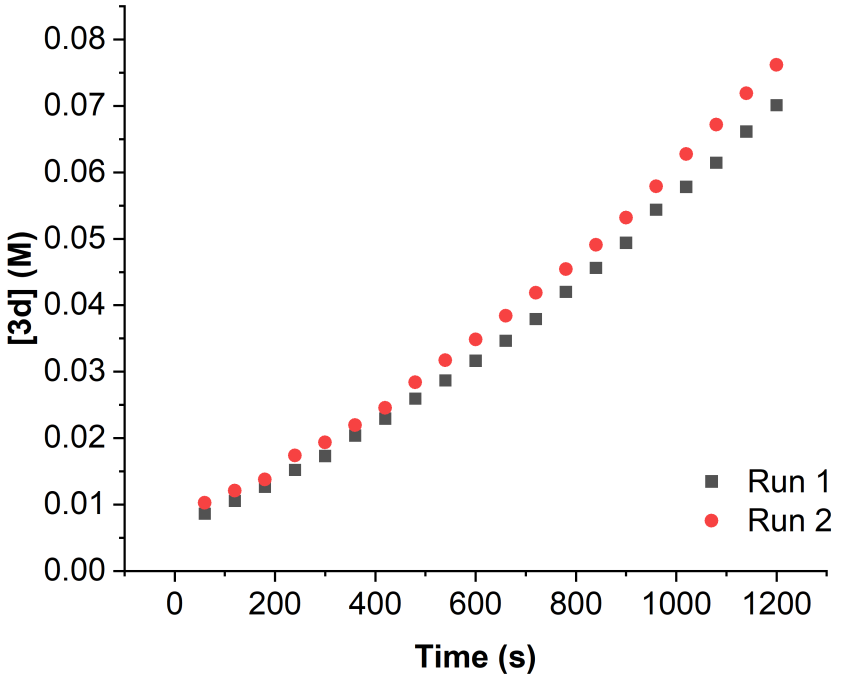

Table S15. Raw kinetic data used to determine the order in iron (0.003211M in complex A, 0.666 in HBpin and 0.333 in Benzonitrile)

Run 1		Run 2	
Time (s)	[3d] (M)	Time (s)	[3d] (M)
60	0.00903	60	0.00725
120	0.01132	120	0.01044
180	0.01458	180	0.01384
240	0.01707	240	0.01743
300	0.0206	300	0.02039
360	0.02424	360	0.02468
420	0.03747	420	0.02833
480	0.03191	480	0.03267
540	0.03648	540	0.03726
600	0.04097	600	0.04132
660	0.04624	660	0.04584
720	0.05147	720	0.05025
780	0.05509	780	0.05522
840	0.06156	840	0.05984
900	0.06578	900	0.06492
960	0.07121	960	0.06978
1020	0.07856	1020	0.07531
1080	0.08366	1080	0.08032
1140	0.08955	1140	0.08616
1200	0.09581	1200	0.09129

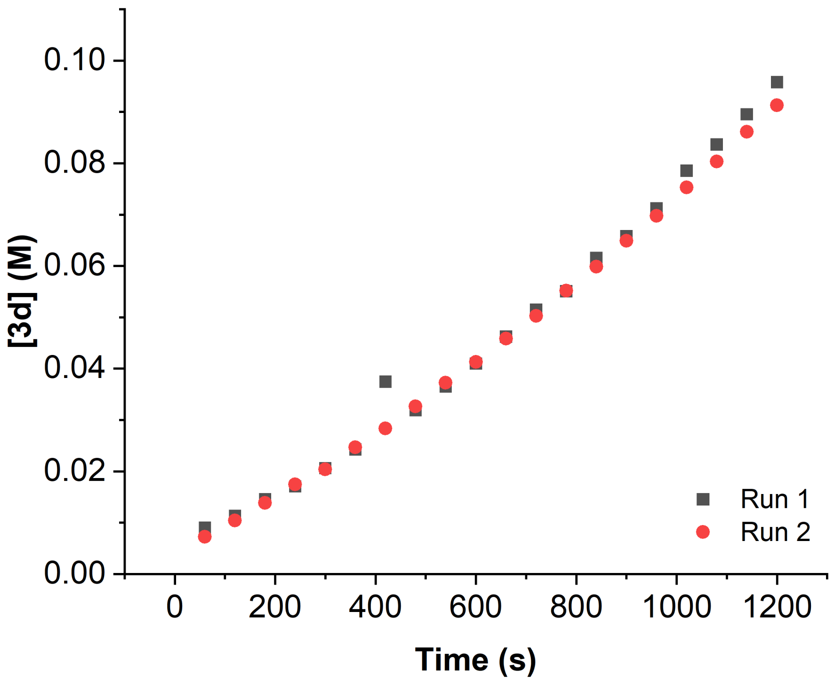

Table S16. Raw kinetic data used to determine the order in iron (0.004067M in complex A, 0.666 in HBpin and 0.333 in Benzonitrile)

Run 1		Run 2	
Time (s)	[3d] (M)	Time (s)	[3d] (M)
60	0.01422	60	0.01153
120	0.01729	120	0.01627
180	0.02283	180	0.02053
240	0.02726	240	0.02537
300	0.03183	300	0.03114
360	0.03678	360	0.03605
420	0.04192	420	0.04172
480	0.05054	480	0.04823
540	0.06279	540	0.05446
600	0.05857	600	0.06152
660	0.06382	660	0.06854
720	0.06993	720	0.07509
780	0.07626	780	0.08256
840	0.08238	840	0.08972
900	0.0891	900	0.0982
960	0.09447	960	0.10618
1020	0.09985	1020	0.11397
1080	0.10872	1080	0.12247
1140	0.11587	1140	0.12912
1200	0.12395	1200	0.14074

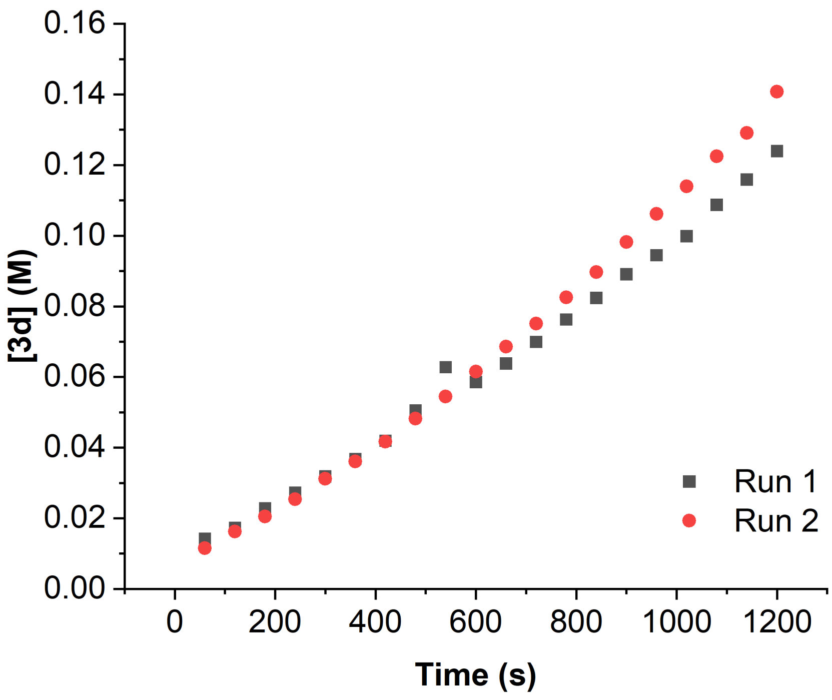

Table S17. Raw kinetic data used to determine the order in iron (0.004864M in complex A, 0.666 in HBpin and 0.333 in Benzonitrile)

Run 1		Run 2	
Time (s)	[3d] (M)	Time (s)	[3d] (M)
60	0.02162	60	0.02372
120	0.02729	120	0.02431
180	0.03222	180	0.02845
240	0.03771	240	0.03344
300	0.04314	300	0.05023
360	0.04969	360	0.04427
420	0.05683	420	0.05078
480	0.06261	480	0.0561
540	0.06945	540	0.06386
600	0.07581	600	0.07066
660	0.08505	660	0.07776
720	0.08983	720	0.08387
780	0.09627	780	0.09248
840	0.10598	840	0.09936
900	0.11355	900	0.10724
960	0.12135	960	0.116
1020	0.13039	1020	0.12358
1080	0.1393	1080	0.1338
1140	0.14935	1140	0.14326
1200	0.15876	1200	0.15179

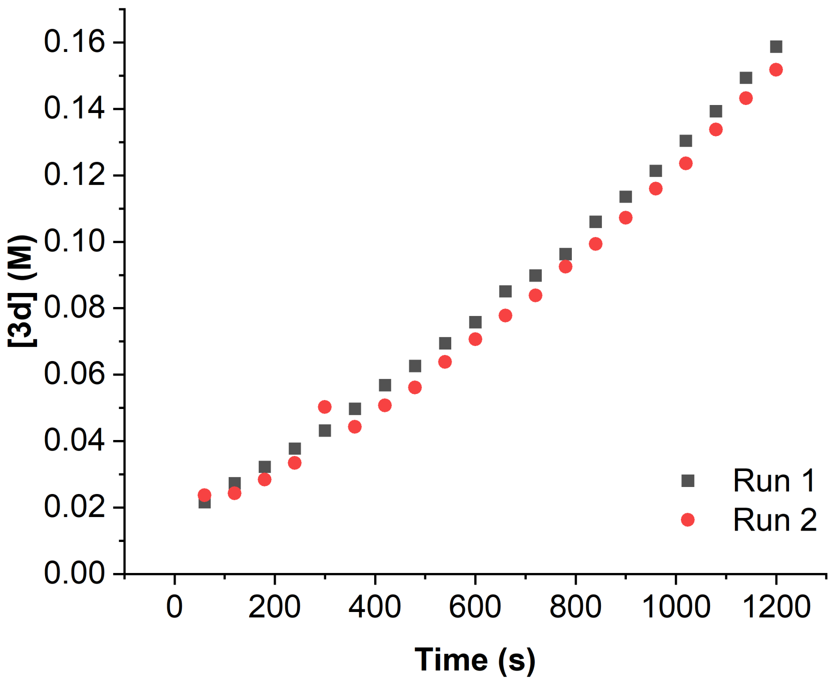

Table S18. Raw kinetic data used to determine the order in HBpin (0.00333M complex A, 0.333M benzonitrile and 0.354M in HBpin)

Run 1		Run 2	
Time (s)	[3d] (M)	Time (s)	[3d] (M)
60	0.00893	60	0.00114
120	0.01145	120	0.00276
180	0.00611	180	0.0043
240	0.00644	240	0.00671
300	0.00987	300	0.00851
360	0.01094	360	0.01074
420	0.0152	420	0.01154
480	0.01568	480	0.01489
540	0.01702	540	0.01808
600	0.01976	600	0.01983
660	0.02194	660	0.02275
720	0.02477	720	0.02514
780	0.02727	780	0.02715
840	0.0308	840	0.03112
900	0.03324	900	0.03335
960	0.03778	960	0.0367
1020	0.04029	1020	0.0401
1080	0.04335	1080	0.04285
1140	0.04632	1140	0.04561
1200	0.04923	1200	0.04841

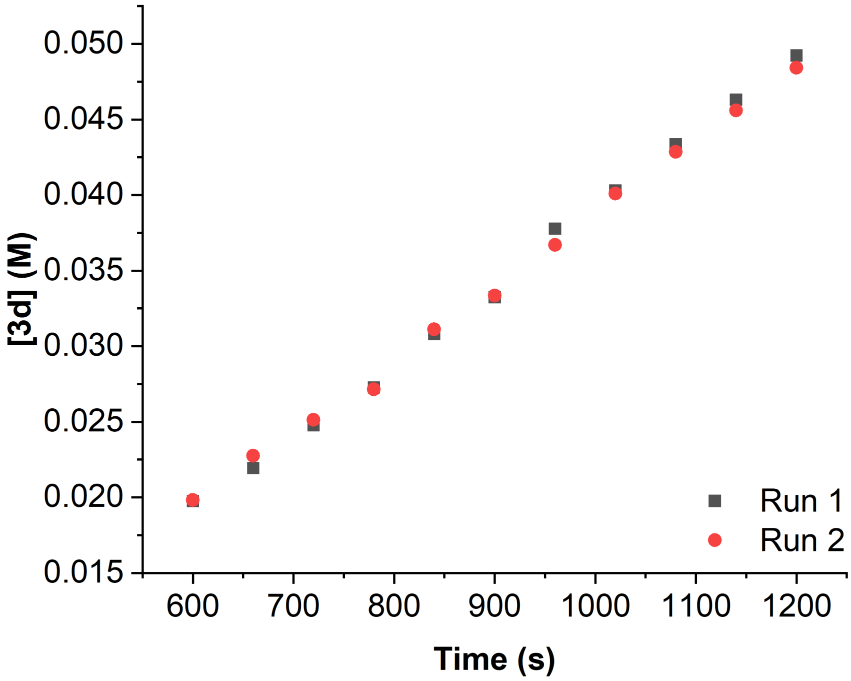

Table S19. Raw kinetic data used to determine the order in HBpin (0.00333M complex A, 0.333M benzonitrile and 0.389M in HBpin)

Run 1		Run 2	
Time (s)	[3d] (M)	Time (s)	[3d] (M)
60	0.00163	60	0.00192
120	0.00177	120	0.0029
180	0.00335	180	0.00456
240	0.00638	240	0.00752
300	0.0091	300	0.00876
360	0.01033	360	0.012
420	0.01405	420	0.0134
480	0.01633	480	0.01637
540	0.01911	540	0.01821
600	0.02364	600	0.02169
660	0.026	660	0.02475
720	0.03084	720	0.02711
780	0.03385	780	0.0311
840	0.0379	840	0.0345
900	0.04064	900	0.03736
960	0.04432	960	0.04034
1020	0.04693	1020	0.04467
1080	0.05357	1080	0.04698
1140	0.05795	1140	0.05188
1200	0.06011	1200	0.05421

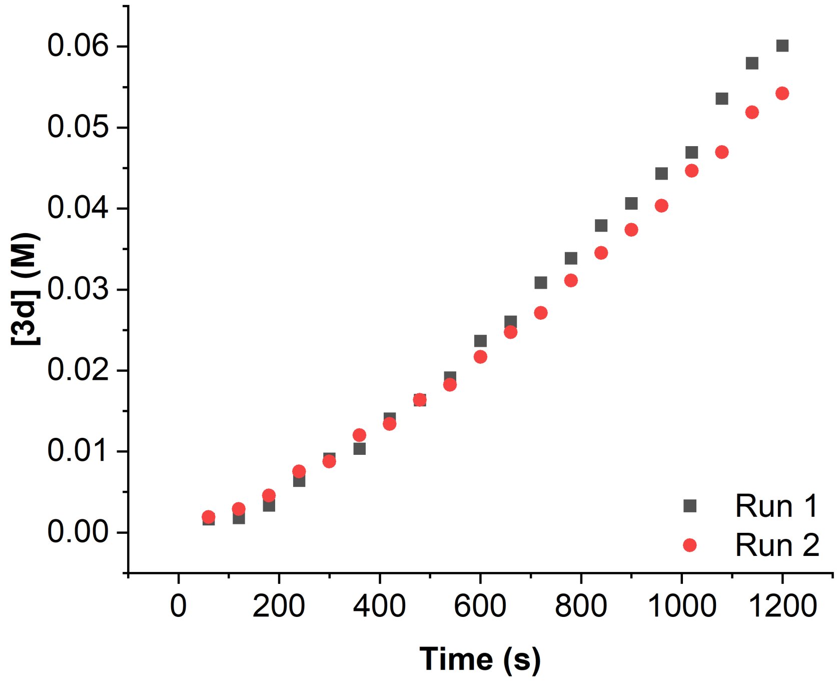

Table S20. Raw kinetic data used to determine the order in HBpin (0.00333M complex A, 0.333M benzonitrile and 0.439M in HBpin)

Run 1		Run 2	
Time (s)	[3d] (M)	Time (s)	[3d] (M)
60	0.00894	60	0.00399
120	0.01112	120	0.00456
180	0.0136	180	0.00755
240	0.01697	240	0.00931
300	0.01927	300	0.01051
360	0.02211	360	0.01501
420	0.0254	420	0.01614
480	0.02957	480	0.01991
540	0.03222	540	0.02308
600	0.03524	600	0.02763
660	0.03989	660	0.03068
720	0.04332	720	0.03408
780	0.04698	780	0.03706
840	0.05096	840	0.04101
900	0.05788	900	0.04535
960	0.06	960	0.04942
1020	0.0633	1020	0.05358
1080	0.06753	1080	0.05756
1140	0.07122	1140	0.06356
1200	0.07556	1200	0.06683

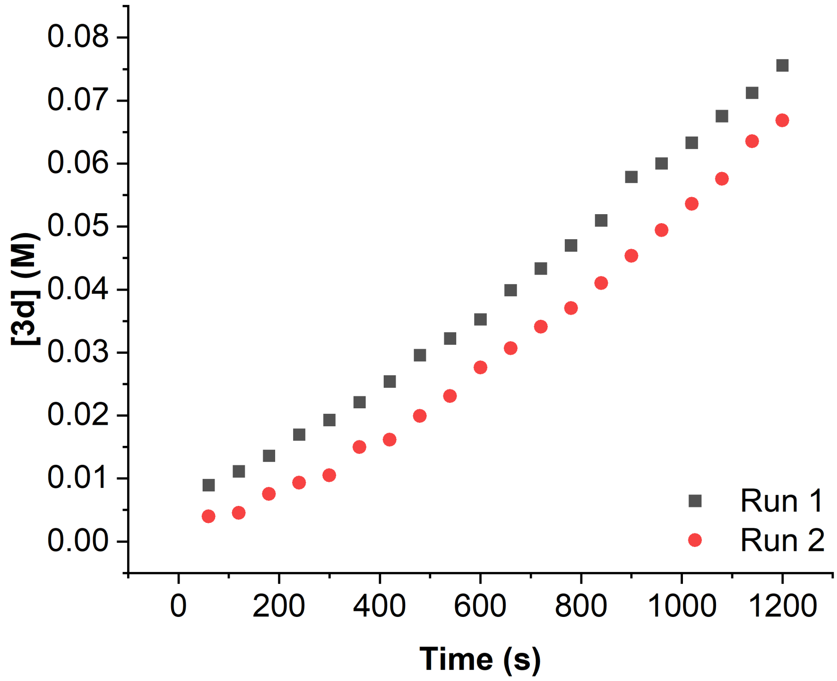

Table S21. Raw kinetic data used to determine the order in HBpin (0.00333M complex A, 0.333M benzonitrile and 0.483M in HBpin)

Run 1		Run 2	
Time (s)	[3d] (M)	Time (s)	[3d] (M)
60	0.00361	60	0.01017
120	0.00484	120	0.01264
180	0.00656	180	0.01647
240	0.00965	240	0.01945
300	0.01202	300	0.02322
360	0.01456	360	0.02698
420	0.01619	420	0.03117
480	0.02081	480	0.035
540	0.0238	540	0.04168
600	0.02694	600	0.04177
660	0.03138	660	0.04588
720	0.03513	720	0.05096
780	0.03855	780	0.05533
840	0.04273	840	0.05909
900	0.04648	900	0.06285
960	0.05131	960	0.06701
1020	0.0553	1020	0.07191
1080	0.06	1080	0.07582
1140	0.06422	1140	0.08099
1200	0.06783	1200	0.08473

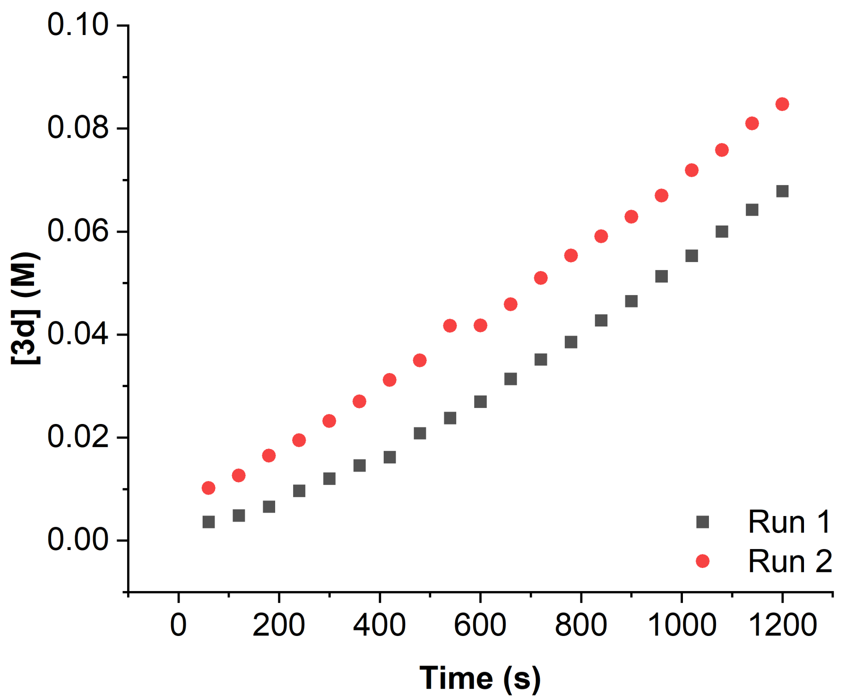

Table S22. Raw kinetic data used to determine the order in HBpin (0.00333M complex A, 0.333M benzonitrile and 0.531M in HBpin)

Run 1		Run 2	
Time (s)	[3d] (M)	Time (s)	[3d] (M)
60	0.00766	60	0.00581
120	0.00914	120	0.00936
180	0.01147	180	0.01311
240	0.01329	240	0.01594
300	0.01554	300	0.01971
360	0.01895	360	0.02348
420	0.02179	420	0.02778
480	0.02519	480	0.03513
540	0.02802	540	0.03652
600	0.03074	600	0.04067
660	0.03382	660	0.04501
720	0.0385	720	0.04941
780	0.0411	780	0.05704
840	0.04585	840	0.05902
900	0.0492	900	0.06335
960	0.05487	960	0.06872
1020	0.05894	1020	0.07148
1080	0.06412	1080	0.07441
1140	0.06817	1140	0.07802
1200	0.07172	1200	0.08377

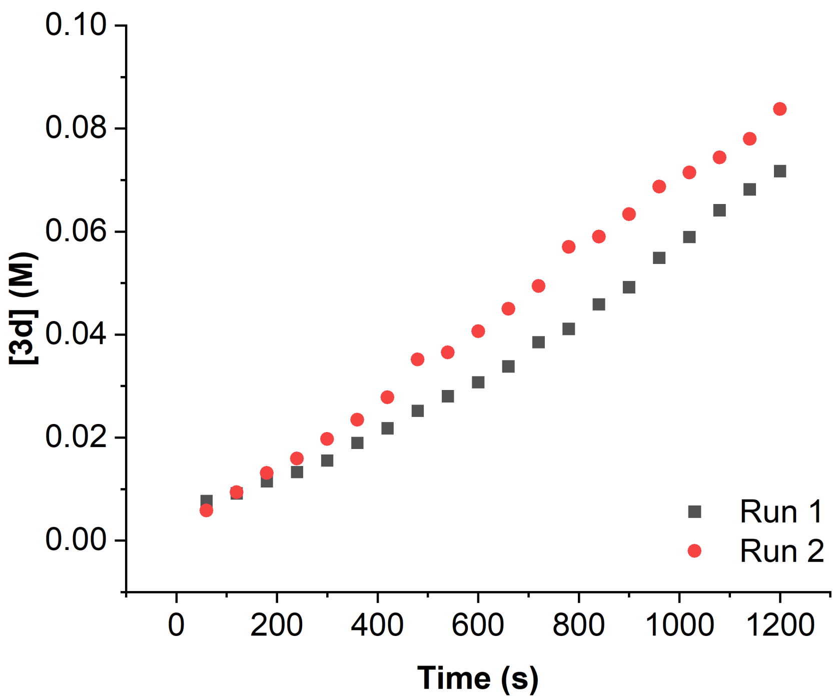

Table S23. Raw kinetic data used to determine the order in HBpin (0.00333M complex A, 0.333M benzonitrile and 0.571M in HBpin)

Run 1		Run 2	
Time (s)	[3d] (M)	Time (s)	[3d] (M)
60	0.00728	60	0.00782
120	0.00866	120	0.00968
180	0.01228	180	0.01145
240	0.01478	240	0.01447
300	0.01789	300	0.01825
360	0.02064	360	0.02212
420	0.03196	420	0.02547
480	0.02888	480	0.02844
540	0.03233	540	0.03236
600	0.03675	600	0.03357
660	0.04168	660	0.03802
720	0.04585	720	0.04197
780	0.04973	780	0.04597
840	0.05449	840	0.04994
900	0.05961	900	0.05372
960	0.06259	960	0.059
1020	0.06736	1020	0.06261
1080	0.07147	1080	0.06702
1140	0.07459	1140	0.0713
1200	0.07974	1200	0.07526

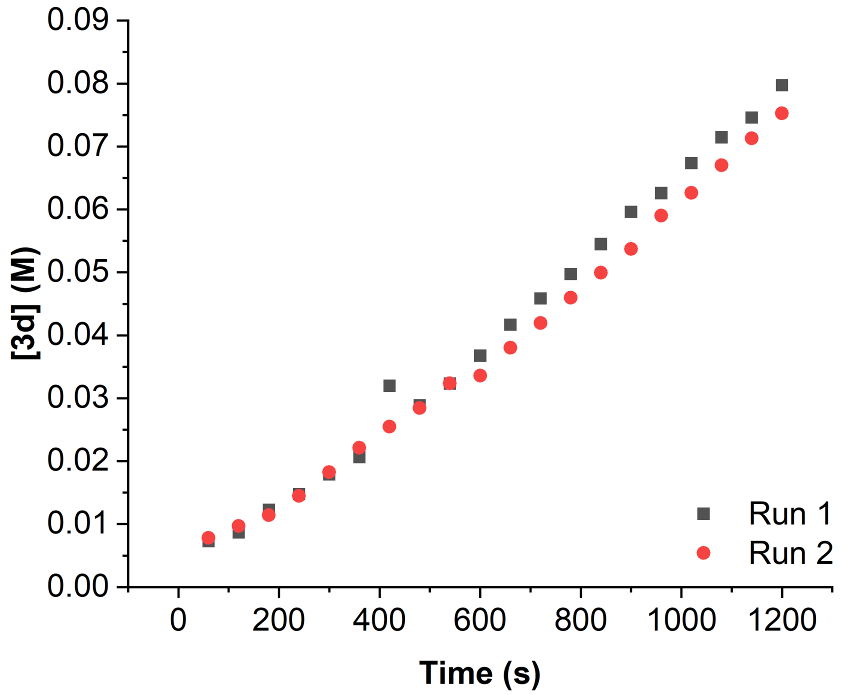

Table S24. Raw kinetic data used to determine the order in HBpin (0.00333M complex A, 0.333M benzonitrile and 0.612M in HBpin)

Run 1		Run 2	
Time (s)	[3d] (M)	Time (s)	[3d] (M)
60	0.00665	60	0.00936
120	0.00878	120	0.0116
180	0.01048	180	0.01416
240	0.01296	240	0.0171
300	0.0167	300	0.02138
360	0.01867	360	0.0255
420	0.0227	420	0.03833
480	0.0258	480	0.03661
540	0.02905	540	0.03709
600	0.03295	600	0.03971
660	0.0383	660	0.04457
720	0.04311	720	0.04906
780	0.04856	780	0.05387
840	0.05186	840	0.05754
900	0.05552	900	0.0626
960	0.05765	960	0.06654
1020	0.06255	1020	0.07031
1080	0.06784	1080	0.07486
1140	0.07199	1140	0.07896
1200	0.07541	1200	0.08355

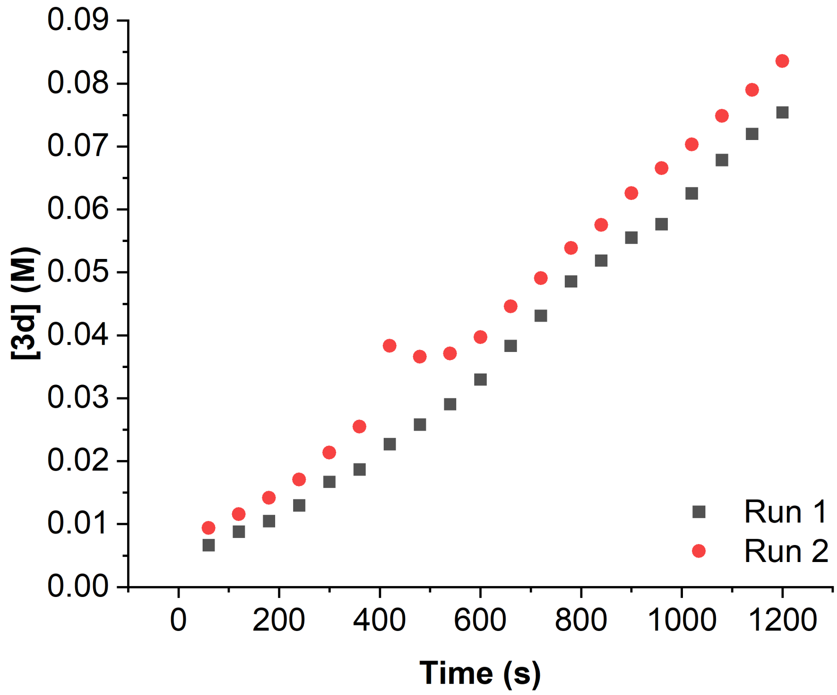

Table S25. Raw kinetic data used to determine the order in nitrile (0.00333M complex A, 0.333M benzonitrile and 0.06182M in HBpin)

Run 1		Run 2	
Time (s)	[3d] (M)	Time (s)	[3d] (M)
60	0.036	60	0.03038
120	0.05213	120	0.04821
180	0.07233	180	0.06921
240	0.09691	240	0.09167
300	0.12899	300	0.11529
360	0.15052	360	0.14129
420	0.18484	420	0.17107
480	0.21168	480	0.19948
540	0.24029	540	0.22993
600	0.27226	600	0.2557
660	0.30257	660	0.2872
720	0.33803	720	0.31758
780	0.36029	780	0.34945
840	0.39388	840	0.38537
900	0.4159	900	0.41622
960	0.45261	960	0.44123
1020	0.48952	1020	0.47395
1080	0.52848	1080	0.50558
1140	0.55835	1140	0.54571
1200	0.58893	1200	0.5705

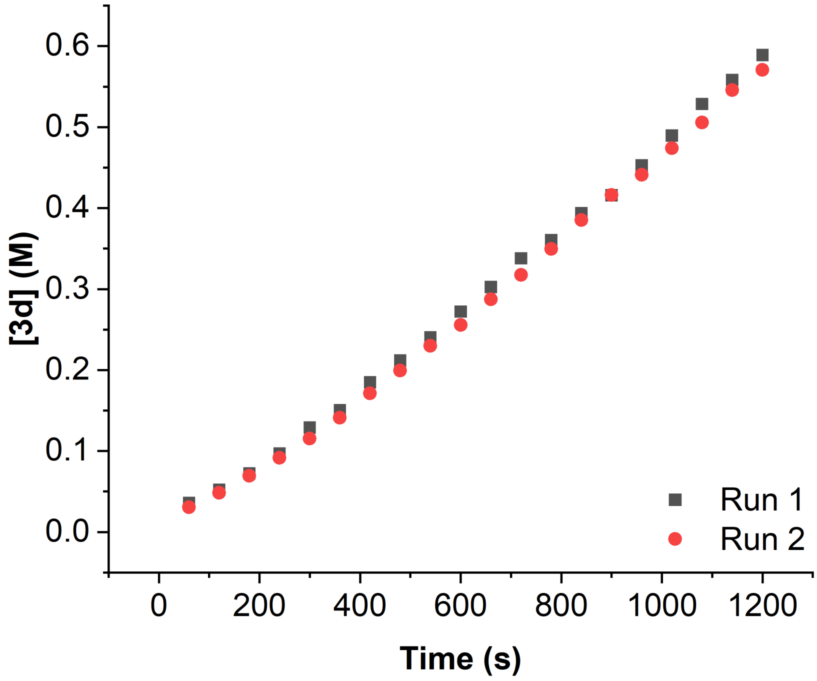

Table S26. Raw kinetic data used to determine the order in nitrile (0.00333M complex A, 0.333M benzonitrile and 0.1237M in HBpin)

Run 1		Run 2	
Time (s)	[3d] (M)	Time (s)	[3d] (M)
60	0.00917	60	0.01586
120	0.01505	120	0.0233
180	0.02038	180	0.03186
240	0.02906	240	0.04381
300	0.03632	300	0.04996
360	0.04755	360	0.06188
420	0.05581	420	0.07213
480	0.06722	480	0.08311
540	0.08103	540	0.10276
600	0.08832	600	0.10383
660	0.09971	660	0.11969
720	0.11245	720	0.13405
780	0.12353	780	0.15311
840	0.13838	840	0.16281
900	0.15155	900	0.17659
960	0.17019	960	0.19789
1020	0.18299	1020	0.20412
1080	0.19973	1080	0.2209
1140	0.21392	1140	0.23505
1200	0.22589	1200	0.25193

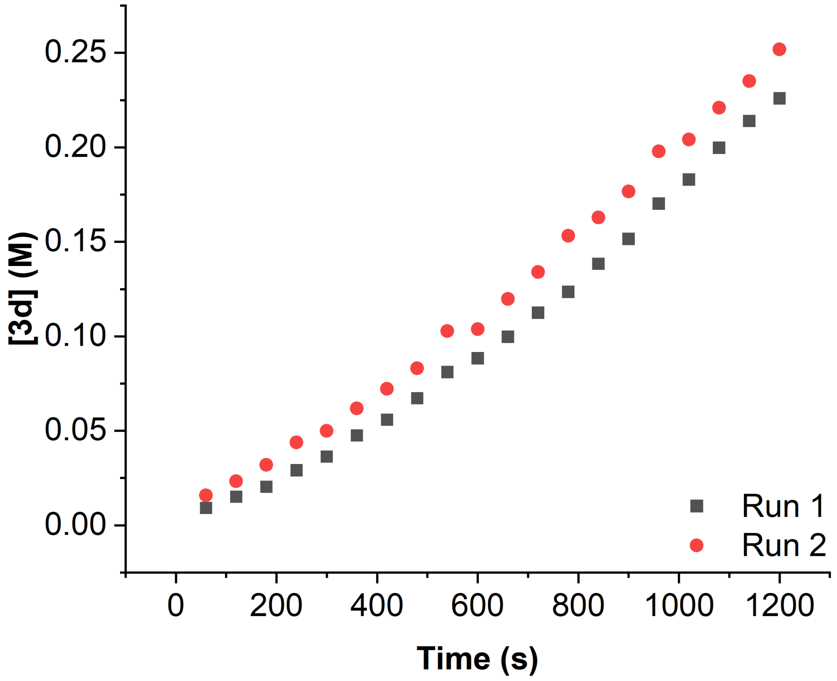

Table S27. Raw kinetic data used to determine the order in nitrile (0.00333M complex A, 0.333M benzonitrile and 0.1586M in HBpin)

Run 1		Run 2	
Time (s)	[3d] (M)	Time (s)	[3d] (M)
60	0.00603	60	0.01121
120	0.01031	120	0.01582
180	0.01705	180	0.02243
240	0.02233	240	0.03008
300	0.03744	300	0.03698
360	0.03618	360	0.04649
420	0.04288	420	0.05486
480	0.05267	480	0.06341
540	0.07742	540	0.07199
600	0.06688	600	0.08142
660	0.07879	660	0.09053
720	0.08157	720	0.09975
780	0.09357	780	0.10942
840	0.10212	840	0.11871
900	0.11006	900	0.12577
960	0.12046	960	0.13875
1020	0.13457	1020	0.14879
1080	0.14679	1080	0.15802
1140	0.15745	1140	0.16963
1200	0.164	1200	0.17761

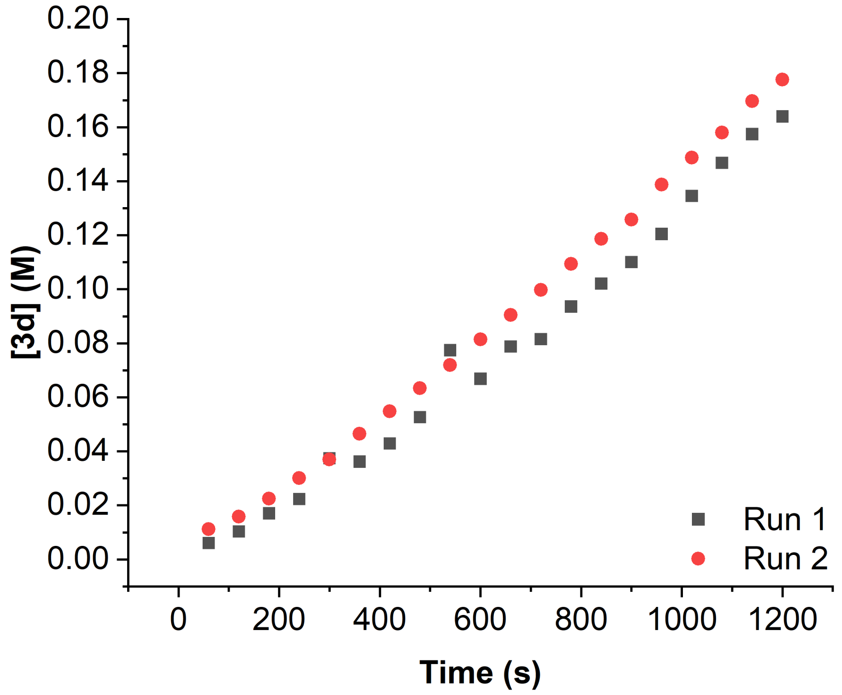

Table S28. Raw kinetic data used to determine the order in nitrile (0.00333M complex A, 0.333M benzonitrile and 0.2472M in HBpin)

Run 1		Run 2	
Time (s)	[3d] (M)	Time (s)	[3d] (M)
60	0.00603	60	0.01121
120	0.01031	120	0.01582
180	0.01705	180	0.02243
240	0.02233	240	0.03008
300	0.03744	300	0.03698
360	0.03618	360	0.04649
420	0.04288	420	0.05486
480	0.05267	480	0.06341
540	0.07742	540	0.07199
600	0.06688	600	0.08142
660	0.07879	660	0.09053
720	0.08157	720	0.09975
780	0.09357	780	0.10942
840	0.10212	840	0.11871
900	0.11006	900	0.12577
960	0.12046	960	0.13875
1020	0.13457	1020	0.14879
1080	0.14679	1080	0.15802
1140	0.15745	1140	0.16963
1200	0.164	1200	0.17761

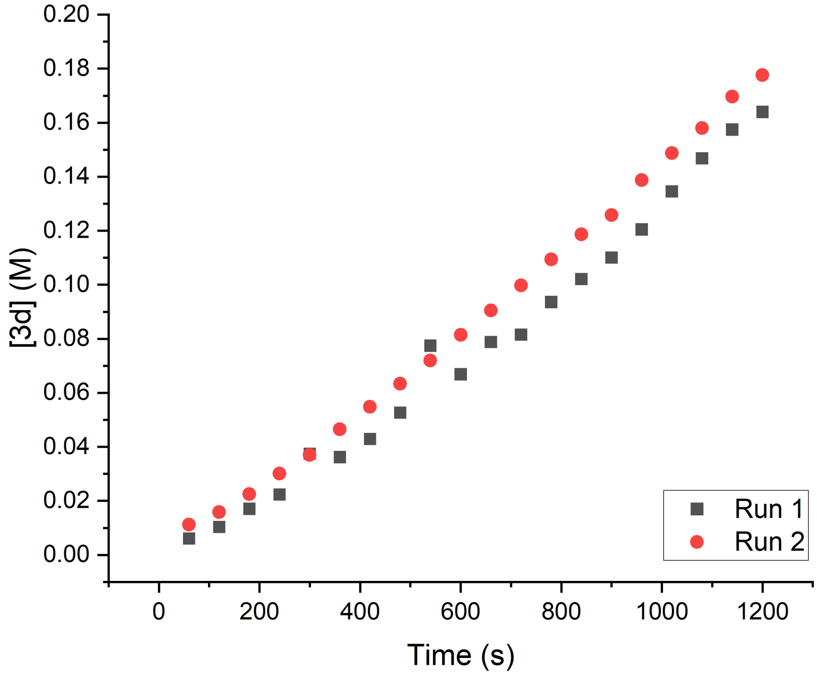

Table S29. Raw kinetic data used to determine the temperature dependence (313 K)

Run 1		Run 2	
Time (s)	[3d] (M)	Time (s)	[3d] (M)
60	9.98877E-4	60	7.44801E-4
120	0.0012	120	0.00202
180	0.00177	180	0.00157
240	0.0033	240	0.00314
300	0.00397	300	0.00403
360	0.00528	360	0.00519
420	0.00639	420	0.00627
480	0.00741	480	0.00715
540	0.0085	540	0.00836
600	0.00981	600	0.00982
660	0.0113	660	0.01136
720	0.01247	720	0.01233
780	0.01418	780	0.0142
840	0.01526	840	0.01467
900	0.0167	900	0.01667
960	0.01798	960	0.01754
1020	0.01957	1020	0.0194
1080	0.02103	1080	0.02076
1140	0.0228	1140	0.02239
1200	0.02457	1200	0.02475

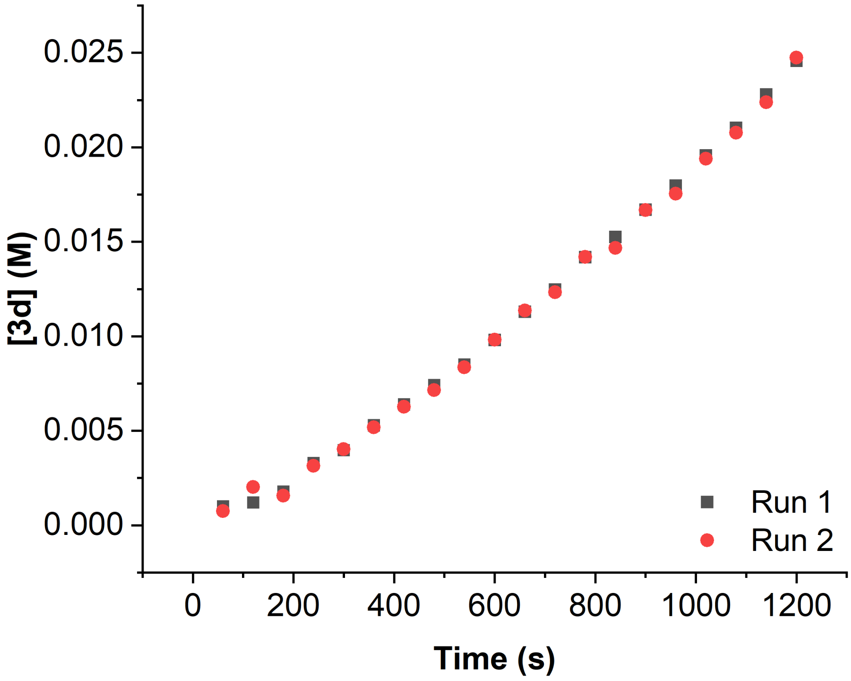

Table S30. Raw kinetic data used to determine the temperature dependence (323 K)

Run 1		Run 2	
Time (s)	[3d] (M)	Time (s)	[3d] (M)
60	0.00883	60	0.0087
120	0.01124	120	0.01154
180	0.01528	180	0.01511
240	0.01911	240	0.01765
300	0.02306	300	0.02228
360	0.02682	360	0.02577
420	0.03188	420	0.02977
480	0.0353	480	0.03438
540	0.04003	540	0.03812
600	0.04455	600	0.04253
660	0.04961	660	0.04732
720	0.05519	720	0.05199
780	0.05971	780	0.05705
840	0.06929	840	0.06218
900	0.0713	900	0.06735
960	0.07653	960	0.07215
1020	0.08181	1020	0.07779
1080	0.08801	1080	0.08302
1140	0.09415	1140	0.08905
1200	0.09929	1200	0.09596

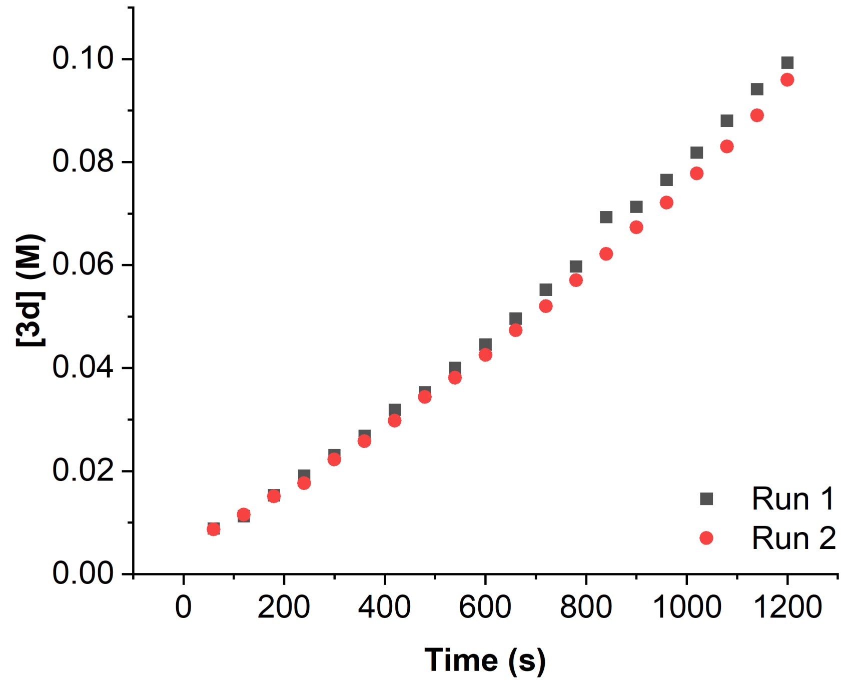

Table S31. Raw kinetic data used to determine the temperature dependence (333 K)

Run 1		Run 2	
Time (s)	[3d] (M)	Time (s)	[3d] (M)
60	0.02429	60	0.01201
120	0.03755	120	0.02231
180	0.05159	180	0.03481
240	0.07536	240	0.05181
300	0.09073	300	0.06367
360	0.1114	360	0.08022
420	0.14055	420	0.09717
480	0.1425	480	0.12266
540	0.16448	540	0.14365
600	0.18705	600	0.17334
660	0.21147	660	0.18867
720	0.2599	720	0.22572
780	0.26076	780	0.23898
840	0.31222	840	0.26383
900	0.34085	900	0.29915
960	0.37265	960	0.31862
1020	0.3998	1020	0.36155
1080	0.43114	1080	0.37251
1140	0.45552	1140	0.40857
1200	0.48235	1200	0.4479

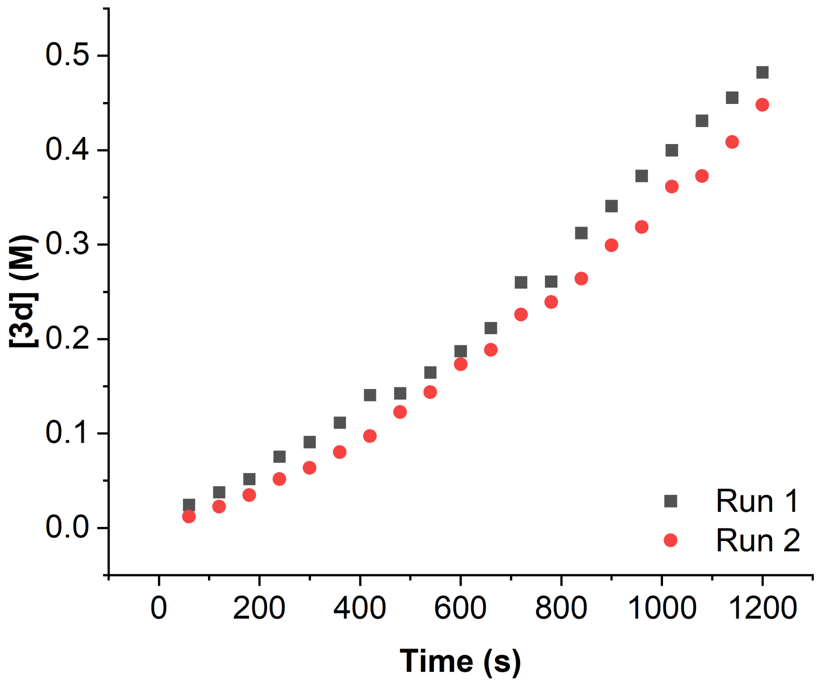

Table S32. Raw kinetic data used to determine the temperature dependence (343 K)

Run 1		Run 2	
Time (s)	[3d] (M)	Time (s)	[3d] (M)
60	0.05011	60	0.05808
120	0.07536	120	0.0908
180	0.10912	180	0.13163
240	0.15009	240	0.17525
300	0.19897	300	0.22275
360	0.24827	360	0.30363
420	0.31402	420	0.36587
480	0.35925	480	0.43989
540	0.43161	540	0.50658
600	0.49281	600	0.57481
660	0.55713	660	0.65923
720	0.6052	720	0.71733
780	0.68763	780	0.79172
840	0.75348	840	0.87405
900	0.8158	900	0.9482
960	0.87849	960	0.99838
1020	0.94716	1020	1.0762
1080	1.01325	1080	1.13776
1140	1.07192	1140	1.19658
1200	1.14933	1200	1.26323

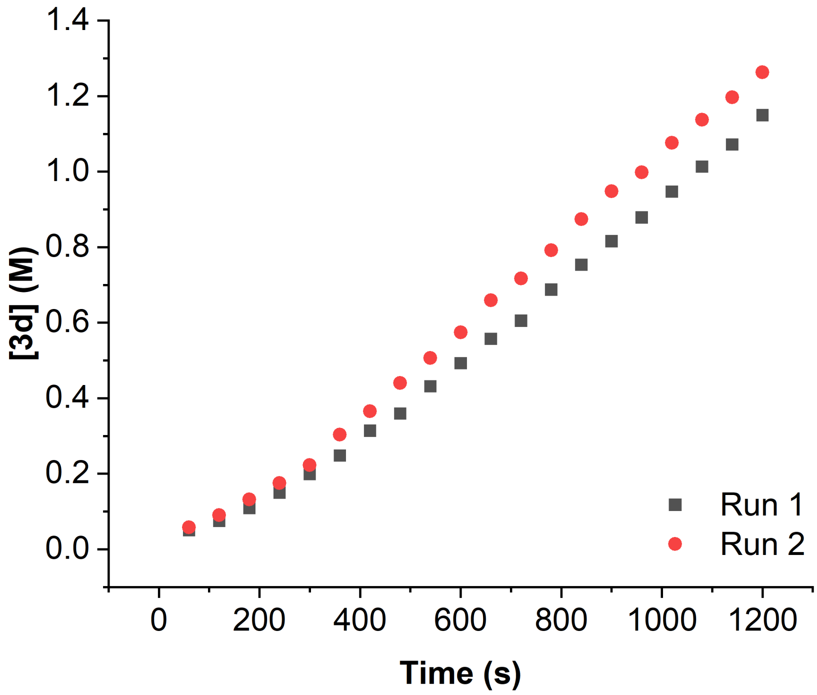

Table S33. Raw kinetic data used to determine the KIE (DBPin rate data)

Run 1		Run 2	
Time (s)	[3d] (M)	Time (s)	[3d] (M)
60	0.05924	60	0.03774
120	0.07244	120	0.0568
180	0.09071	180	0.07029
240	0.1133	240	0.08088
300	0.12529	300	0.10125
360	0.14342	360	0.12204
420	0.16331	420	0.13559
480	0.18006	480	0.15766
540	0.20066	540	0.1708
600	0.21485	600	0.21005
660	0.23712	660	0.21466
720	0.25849	720	0.23581
780	0.27062	780	0.2622
840	0.2973	840	0.27596
900	0.31033	900	0.30079
960	0.33478	960	0.31742
1020	0.35756	1020	0.33806
1080	0.3725	1080	0.36274
1140	0.39789	1140	0.37315
1200	0.41096	1200	0.4023

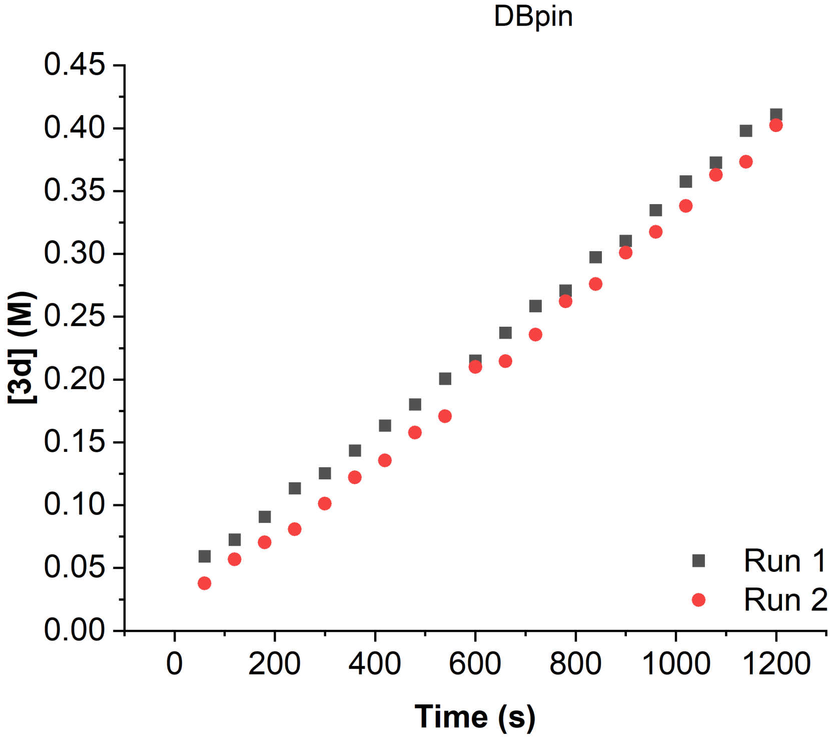

Table S34. Raw kinetic data used to determine the KIE (HBPin rate data)

Run 1		Run 2	
Time (s)	[3d] (M)	Time (s)	[3d] (M)
60	0.00734	60	0.005
120	0.01192	120	0.00927
180	0.017	180	0.01357
240	0.02052	240	0.01837
300	0.0256	300	0.02333
360	0.03258	360	0.02944
420	0.03681	420	0.03422
480	0.04224	480	0.04117
540	0.05086	540	0.04737
600	0.05409	600	0.05321
660	0.05998	660	0.05995
720	0.06574	720	0.06647
780	0.07376	780	0.07328
840	0.08	840	0.07954
900	0.08649	900	0.08602
960	0.0939	960	0.09286
1020	0.09976	1020	0.10002
1080	0.11026	1080	0.10709
1140	0.11305	1140	0.11344
1200	0.12175	1200	0.12118

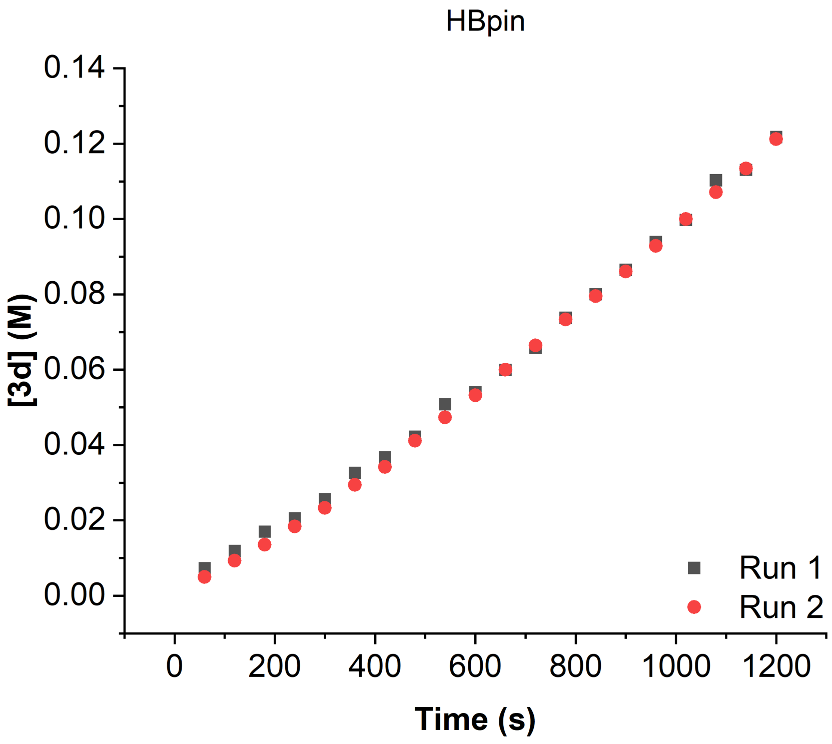

References

[1] A. Kaithal, B. Chatterjee, C. Gunanathan, *J. Org. Chem*. **2016**, *81*, 11153–11161.

[2] CrysAlisPro, Agilent Technologies, Yarton, UK, 2017.

[3] L. J. Bourhis, O. V. Dolomanov, R. J. Gildea, J. A. K. Howard, H. Puschmann, *Acta Cryst*. **2015**, *A71*, 59–75.

[4] O. V. Dolomanov, L. J. Bourhis, R. J. Gildea, J. A. K. Howard, H. Puschmann, *J. Appl. Crystallogr*. **2009**, *42*, 339–341.

[5] L. J. Farrugia, *J. Appl. Crystallogr*. **2012**, *45*, 849–854.

[6] Gaussian-09, Revision D.01, M. J. Frisch, G. W. Trucks, H. B. Schlegel, G. E. Scuseria, M. A. Robb, J. R. Cheeseman, G. Scalmani, V. Barone, B. Mennucci, G. A. Petersson, H. Nakatsuji, M. Caricato, X. Li, H. P. Hratchian, A. F. Izmaylov, J. Bloino, G. Zheng, J. L. Sonnenberg, M. Hada, M. Ehara, K. Toyota, R. Fukuda, J. Hasegawa, M. Ishida, T. Nakajima, Y. Honda, O. Kitao, H. Nakai, T. Vreven, J. A. Montgomery, Jr., J. E. Peralta, F. Ogliaro, M. Bearpark, J. J. Heyd, E. Brothers, K. N. Kudin, V. N. Staroverov, T. Keith, R. Kobayashi, J. Normand, K. Raghavachari, A. Rendell, J. C. Burant, S. S. Iyengar, J. Tomasi, M. Cossi, N. Rega, J. M. Millam, M. Klene, J. E. Knox, J B Cross, V. Bakken, C. Adamo, J. Jaramillo, R. Gomperts, R. E. Stratmann, O. Yazyev, A. J. Austin, R. Cammi, C. Pomelli, J. W. Ochterski, R. L. Martin, K. Morokuma, V. G. Zakrzewski, G. A. Voth, P. Salvador, J. J. Dannenberg, S. Dapprich, A. D. Daniels, O. Farkas, J. B. Foresman, J. V. Ortiz, J. Cioslowski, D. J. Fox, Gaussian, Inc., Wallingford CT, 2010.

[7] (a) A. D. Becke, *J. Chem. Phys*. **1993**, *98*, 5648−5652. (b) C. Lee, W. Yang, R. G. Parr, *Phys. Rev. B*. **1988**, *37*, 785−789. (c) S. Grimme, J. Antony, S. Ehrlich, H. Krieg, *J. Chem. Phys*. **2010**, *132*, 154104.

[8] A. Schaefer, H. Horn, R. Ahlrichs, *J. Chem. Phys*. **1992**, *97*, 2571–2577.

[9] J. Tomasi, B. Mennucci, R. Cammi, *Chem. Rev*. **2005**, *105*, 2999–3009.

[10] (a) A. Hernández-Ortega, M. G. Quesne, S. Bui, D. J. Heyes, R. A. Steiner, N. S. Scrutton, S. P. de Visser, *J. Am. Chem. Soc*. **2015**, *137*, 7474−7487. (b) F. Himo, S. P. de Visser, *Commun. Chem*. **2022**, *5*, 29. (c) C. -C. G. Yeh, T. Mokkawes, J. M. Bradley, N. E. Le Brun, S. P. de Visser, *ChemBioChem*. **2022**, *2022*, e202200257.

[11] (a) D. H. Wertz, *J. Am. Chem. Soc*. **1980**, *102*, 5316−5322. (b) F. G.Cantú Reinhard, A. S. Faponle, S. P. de Visser, *J. Phys. Chem. A*, **2016**, *120*, 9805–9814.
